# Spatial, temporal, and demographic patterns in prevalence of chewing tobacco use in 204 countries and territories, 1990–2019: a systematic analysis from the Global Burden of Disease Study 2019

**DOI:** 10.1016/S2468-2667(21)00065-7

**Published:** 2021-05-28

**Authors:** GBD 2019 Chewing Tobacco Collaborators, Parkes J Kendrick, Marissa B Reitsma, Mohsen Abbasi-Kangevari, Amir Abdoli, Mohammad Abdollahi, Aidin Abedi, E S Abhilash, Victor Aboyans, Oladimeji M Adebayo, Shailesh M Advani, Bright Opoku Ahinkorah, Sohail Ahmad, Keivan Ahmadi, Haroon Ahmed, Budi Aji, Yonas Akalu, Chisom Joyqueenet Akunna, Fares Alahdab, Ziyad Al-Aly, Fahad Mashhour Alanezi, Turki M Alanzi, Khalid F Alhabib, Tilahun Ali, Sheikh Mohammad Alif, Vahid Alipour, Syed Mohamed Aljunid, Mahmoud A Alomari, Tarek Tawfik Amin, Saeed Amini, Hubert Amu, Robert Ancuceanu, Jason A Anderson, Catalina Liliana Andrei, Tudorel Andrei, Alireza Ansari-Moghaddam, Benny Antony, Davood Anvari, Jalal Arabloo, Nicholas D Arian, Monika Arora, Kurnia Dwi Artanti, Wondwossen Niguse Asmare, Desta Debalkie Atnafu, Marcel Ausloos, Asma Tahir Awan, Getinet Ayano, Getie Lake Aynalem, Samad Azari, Darshan B B, Ashish D Badiye, Atif Amin Baig, Maciej Banach, Srikanta K Banerjee, Suzanne Lyn Barker-Collo, Till Winfried Bärnighausen, Hiba Jawdat Barqawi, Sanjay Basu, Mohsen Bayati, Shahrzad Bazargan-Hejazi, Tariku Tesfaye Bekuma, Derrick A Bennett, Isabela M Bensenor, Habib Benzian, Catherine P Benziger, Adam E Berman, Akshaya Srikanth Bhagavathula, Neeraj Bhala, Nikha Bhardwaj, Pankaj Bhardwaj, Krittika Bhattacharyya, Sadia Bibi, Ali Bijani, Antonio Biondi, Dejana Braithwaite, Hermann Brenner, Andre R Brunoni, Katrin Burkart, Sharath Burugina Nagaraja, Zahid A Butt, Florentino Luciano Caetano dos Santos, Josip Car, Giulia Carreras, Joao Mauricio Castaldelli-Maia, Maria Sofia Sofia Cattaruzza, Jung-Chen Chang, Pankaj Chaturvedi, Simiao Chen, Onyema greg Chido-Amajuoyi, Dinh-Toi Chu, Sheng-Chia Chung, Liliana G Ciobanu, Vera Marisa Costa, Rosa A S Couto, Baye Dagnew, Xiaochen Dai, Albertino Antonio Moura Damasceno, Giovanni Damiani, Lalit Dandona, Rakhi Dandona, Parnaz Daneshpajouhnejad, Jiregna Darega Gela, Meseret Derbew Molla, Abebaw Alemayehu Desta, Samath Dhamminda Dharmaratne, Meghnath Dhimal, Arielle Wilder Eagan, Mohammad Ebrahimi Kalan, Kristina Edvardsson, Andem Effiong, Maha El Tantawi, Iffat Elbarazi, Saman Esmaeilnejad, Ibtihal Fadhil, Emerito Jose A Faraon, Medhat Farwati, Farshad Farzadfar, Mehdi Fazlzadeh, Valery L Feigin, Rachel Feldman, Irina Filip, Filippos Filippidis, Florian Fischer, Luisa Sorio Flor, Nataliya A Foigt, Morenike Oluwatoyin Folayan, Masoud Foroutan, Mohamed M Gad, Silvano Gallus, Biniyam Sahiledengle Geberemariyam, Birhan Gebresillassie Gebregiorgis, Lemma Getacher, Abera Getachew Obsa, Mansour Ghafourifard, Reza Ghanei Gheshlagh, Ahmad Ghashghaee, Nermin Ghith, Gabriela Fernanda Gil, Paramjit Singh Gill, Ibrahim Abdelmageed Ginawi, Salime Goharinezhad, Mahaveer Golechha, Sameer Vali Gopalani, Giuseppe Gorini, Michal Grivna, Avirup Guha, Rafael Alves Guimarães, Yuming Guo, Rajat Das Gupta, Rajeev Gupta, Tarun Gupta, Vin Gupta, Nima Hafezi-Nejad, Mohammad Rifat Haider, Randah R Hamadeh, Graeme J Hankey, Arief Hargono, Simon I Hay, Golnaz Heidari, Claudiu Herteliu, Kamal Hezam, Thomas R Hird, Ramesh Holla, Mehdi Hosseinzadeh, Mihaela Hostiuc, Sorin Hostiuc, Mowafa Househ, Thomas Hsiao, Junjie Huang, Charles Ugochukwu Ibeneme, Segun Emmanuel Ibitoye, Irena M Ilic, Milena D Ilic, Leeberk Raja Inbaraj, Seyed Sina Naghibi Irvani, Jessica Y Islam, Rakibul M Islam, Sheikh Mohammed Shariful Islam, Farhad Islami, Hiroyasu Iso, Ramaiah Itumalla, Jalil Jaafari, Vardhmaan Jain, Mihajlo Jakovljevic, Sung-In Jang, Shubha Jayaram, Panniyammakal Jeemon, Ravi Prakash Jha, Jost B Jonas, Mikk Jürisson, Ali Kabir, Zubair Kabir, Leila R Kalankesh, Tanuj Kanchan, Himal Kandel, Neeti Kapoor, André Karch, Salah Eddin Karimi, Kindie Mitiku Kebede, Bayew Kelkay, Ryan David Kennedy, Yousef Saleh Khader, Ejaz Ahmad Khan, Maryam Khayamzadeh, Gyu Ri Kim, Ruth W Kimokoti, Mika Kivimäki, Soewarta Kosen, Sindhura Lakshmi Koulmane Laxminarayana, Ai Koyanagi, Kewal Krishan, Nuworza Kugbey, G Anil Kumar, Nithin Kumar, Om P Kurmi, Dian Kusuma, Ben Lacey, Iván Landires, Savita Lasrado, Paolo Lauriola, Doo Woong Lee, Yo Han Lee, Janni Leung, Shanshan Li, Hualiang Lin, Wei Liu, Alessandra Lugo, Shilpashree Madhava Kunjathur, Azeem Majeed, Afshin Maleki, Reza Malekzadeh, Deborah Carvalho Malta, Abdullah A Mamun, Narayana Manjunatha, Borhan Mansouri, Mohammad Ali Mansournia, Santi Martini, Manu Raj Mathur, Prashant Mathur, Mohsen Mazidi, Martin McKee, Carlo Eduardo Medina-Solís, Suresh Mehata, Walter Mendoza, Ritesh G Menezes, Bartosz Miazgowski, Irmina Maria Michalek, Ted R Miller, GK Mini, Andreea Mirica, Erkin M Mirrakhimov, Hamed Mirzaei, Sanjeev Misra, Yousef Mohammad, Abdollah Mohammadian-Hafshejani, Shafiu Mohammed, Ali H Mokdad, Mariam Molokhia, Lorenzo Monasta, Mohammad Ali Moni, Rahmatollah Moradzadeh, Shane Douglas Morrison, Tilahun Belete Mossie, Sumaira Mubarik, Erin C Mullany, Christopher J L Murray, Shankar Prasad Nagaraju, Mohsen Naghavi, Nitish Naik, Mahdi Nalini, Vinay Nangia, Atta Abbas Naqvi, Sreenivas Narasimha Swamy, Muhammad Naveed, Javad Nazari, Sabina O Nduaguba, Ruxandra Irina Negoi, Sandhya Neupane Kandel, Huong Lan Thi Nguyen, Yeshambel T Nigatu, Molly R Nixon, Chukwudi A Nnaji, Jean Jacques Noubiap, Christoph Nowak, Virginia Nuñez-Samudio, Felix Akpojene Ogbo, Ayodipupo Sikiru Oguntade, In-Hwan Oh, Andrew T Olagunju, Mayowa O Owolabi, Mahesh P A, Keyvan Pakshir, Adrian Pana, Demosthenes Panagiotakos, Songhomitra Panda-Jonas, Ashok Pandey, Utsav Parekh, Eun-Cheol Park, Eun-Kee Park, Fatemeh Pashazadeh Kan, Mona Pathak, Shrikant Pawar, Richard G Pestell, Hai Quang Pham, Marina Pinheiro, Khem Narayan Pokhrel, Akram Pourshams, Akila Prashant, Amir Radfar, Vafa Rahimi-Movaghar, Mohammad Hifz Ur Rahman, Muhammad Aziz Rahman, Amir Masoud Rahmani, Pradhum Ram, Juwel Rana, Chhabi Lal Ranabhat, Priya Rathi, David Laith Rawaf, Salman Rawaf, Reza Rawassizadeh, Andre M N Renzaho, Aziz Rezapour, Mavra A Riaz, Leonardo Roever, Luca Ronfani, Gholamreza Roshandel, Ambuj Roy, Bedanta Roy, Basema Saddik, Amirhossein Sahebkar, Sana Salehi, Hamideh Salimzadeh, Abdallah M Samy, Juan Sanabria, Milena M Santric-Milicevic, Bruno Piassi Sao Jose, Brijesh Sathian, Monika Sawhney, Ganesh Kumar Saya, Falk Schwendicke, Abdul-Aziz Seidu, Nachimuthu Senthil Kumar, Sadaf G Sepanlou, Omid Shafaat, Syed Mahboob Shah, Masood Ali Shaikh, Mohammed Shannawaz, Kiomars Sharafi, Aziz Sheikh, Sara Sheikhbahaei, Mika Shigematsu, Rahman Shiri, Kawkab Shishani, K M Shivakumar, Siddharudha Shivalli, Roman Shrestha, Soraya Siabani, Negussie Boti Sidemo, Inga Dora Sigfusdottir, Rannveig Sigurvinsdottir, João Pedro Silva, Ambrish Singh, Jasvinder A Singh, Virendra Singh, Dhirendra Narain Sinha, Valentin Yurievich Skryabin, Anna Aleksandrovna Skryabina, Ali Soroush, Ireneous N Soyiri, Chandrashekhar T Sreeramareddy, Dan J Stein, Paschalis Steiropoulos, Stefan Stortecky, Kurt Straif, Rizwan Suliankatchi Abdulkader, Gerhard Sulo, Johan Sundström, Takahiro Tabuchi, Eyayou Girma Tadesse, Animut Tagele Tamiru, Minale Tareke, Md Ismail Tareque, Ingan Ukur Tarigan, Bhaskar Thakur, Kavumpurathu Raman Thankappan, Rekha Thapar, Musliu Adetola Tolani, Marcos Roberto Tovani-Palone, Bach Xuan Tran, Jaya Prasad Tripathy, Gebiyaw Wudie Tsegaye, Hayley D Tymeson, Saif Ullah, Brigid Unim, Rachel L Updike, Olalekan A Uthman, Marco Vacante, Constantine Vardavas, Narayanaswamy Venketasubramanian, Madhur Verma, Simone Vidale, Bay Vo, Giang Thu Vu, Yasir Waheed, Yanzhong Wang, Kevin Welding, Andrea Werdecker, Joanna L Whisnant, Nuwan Darshana Wickramasinghe, Befikadu Legesse Wubishet, Kazumasa Yamagishi, Yuichiro Yano, Vahid Yazdi-Feyzabadi, Yigizie Yeshaw, Mohammed Zewdu Yimmer, Naohiro Yonemoto, Zabihollah Yousefi, Chuanhua Yu, Ismaeel Yunusa, Hasan Yusefzadeh, Muhammed Shahriar Zaman, Mohammad Zamani, Maryam Zamanian, Mikhail Sergeevich Zastrozhin, Anasthasia Zastrozhina, Jianrong Zhang, Zhi-Jiang Zhang, Chenwen Zhong, Yves Miel H Zuniga, Emmanuela Gakidou

## Abstract

**Background:**

Chewing tobacco and other types of smokeless tobacco use have had less attention from the global health community than smoked tobacco use. However, the practice is popular in many parts of the world and has been linked to several adverse health outcomes. Understanding trends in prevalence with age, over time, and by location and sex is important for policy setting and in relation to monitoring and assessing commitment to the WHO Framework Convention on Tobacco Control.

**Methods:**

We estimated prevalence of chewing tobacco use as part of the Global Burden of Diseases, Injuries, and Risk Factors Study 2019 using a modelling strategy that used information on multiple types of smokeless tobacco products. We generated a time series of prevalence of chewing tobacco use among individuals aged 15 years and older from 1990 to 2019 in 204 countries and territories, including age-sex specific estimates. We also compared these trends to those of smoked tobacco over the same time period.

**Findings:**

In 2019, 273·9 million (95% uncertainty interval 258·5 to 290·9) people aged 15 years and older used chewing tobacco, and the global age-standardised prevalence of chewing tobacco use was 4·72% (4·46 to 5·01). 228·2 million (213·6 to 244·7; 83·29% [82·15 to 84·42]) chewing tobacco users lived in the south Asia region. Prevalence among young people aged 15–19 years was over 10% in seven locations in 2019. Although global age-standardised prevalence of smoking tobacco use decreased significantly between 1990 and 2019 (annualised rate of change: –1·21% [–1·26 to –1·16]), similar progress was not observed for chewing tobacco (0·46% [0·13 to 0·79]). Among the 12 highest prevalence countries (Bangladesh, Bhutan, Cambodia, India, Madagascar, Marshall Islands, Myanmar, Nepal, Pakistan, Palau, Sri Lanka, and Yemen), only Yemen had a significant decrease in the prevalence of chewing tobacco use, which was among males between 1990 and 2019 (−0·94% [–1·72 to –0·14]), compared with nine of 12 countries that had significant decreases in the prevalence of smoking tobacco. Among females, none of these 12 countries had significant decreases in prevalence of chewing tobacco use, whereas seven of 12 countries had a significant decrease in the prevalence of tobacco smoking use for the period.

**Interpretation:**

Chewing tobacco remains a substantial public health problem in several regions of the world, and predominantly in south Asia. We found little change in the prevalence of chewing tobacco use between 1990 and 2019, and that control efforts have had much larger effects on the prevalence of smoking tobacco use than on chewing tobacco use in some countries. Mitigating the health effects of chewing tobacco requires stronger regulations and policies that specifically target use of chewing tobacco, especially in countries with high prevalence.

**Funding:**

Bloomberg Philanthropies and the Bill & Melinda Gates Foundation.

## Introduction

Effective design of tobacco-control policies and appropriate allocation of resources requires understanding patterns and trends in all types of tobacco use.^1^ Although 138 (77%) of the 180 countries committed to the aims of the WHO Framework Convention on Tobacco Control (FCTC) include smokeless tobacco in their statutes,^2^ smokeless tobacco use has been monitored in far fewer countries than has smoking tobacco use, even in places with high prevalences of smokeless tobacco use.^2^ Only 55 (31%) FCTC countries have data on adult smokeless tobacco use from the past 10 years, and only 70 (39%) have data on smokeless tobacco use among young people.^2^ Additionally, smoked and smokeless tobacco use patterns differ by demographic, socioeconomic, and cultural characteristics,^3^, ^4^, ^5^, ^6^ so detailed information on smokeless tobacco use patterns and trends are needed to tailor interventions that best meet the needs of these different subgroups.

Monitoring of smokeless tobacco use alongside smoked tobacco use should be done for a variety of reasons, including beliefs that it is a safe alternative to smoking, beliefs about a variety of benefits (eg, for morning sickness), and local distribution and production.^6^, ^7^, ^8^ Moreover, smokeless tobacco is less regulated than smoked tobacco. Tobacco manufacturers can sell smokeless tobacco products that are sweeter or flavoured and aimed at new users,^9^ and these products are usually cheaper than cigarettes.^10^ A wide array of products is available in the market, but data on smokeless tobacco use are often not collected by specific products or subtypes, further complicating monitoring and regulation. Although all smokeless tobacco products are consumed through the mouth or nose without burning, the wide variety of products are used in different ways^11^ and are associated with varying degrees and types of harm.^12^, ^13^, ^14^ This study focuses on chewing tobacco use, because the associated health risks are well documented.^12^, ^15^, ^16^ Many studies have found strong evidence for the increased risk of oral cancer due to chewing tobacco.^11^, ^14^, ^17^

**Research in context**

**Evidence before this study**

Previous studies of smokeless tobacco use have found that both prevalence and the type of product used vary widely across countries. Studies on the health effects of smokeless tobacco products have found differences in toxicity by type of product, with chewing tobacco products being the most harmful. Limitations of available survey data have posed a challenge to estimating internally consistent and comparable estimates of product-specific prevalence, disaggregated by location, age, sex, and time period. These limitations have made it difficult to form a comprehensive, global picture of where chewing tobacco is used most, among which age groups and sexes, and how this has changed over time.

**Added value of this study**

This study, based on results from the Global Burden of Diseases, Injuries, and Risk Factors Study 2019, is the first global analysis of prevalence of chewing tobacco use by age, sex, and time period that incorporates information from available nationally representative surveys with questions about smokeless tobacco use. To address the challenge of heterogeneous survey data, of which little were available, we developed and implemented a new approach to combining different definitions and sources of smokeless tobacco prevalence data across locations to mitigate the effects of compositional bias in the available data. These methods improved estimates, particularly in locations that have less chewing tobacco-specific data but do have data on other smokeless tobacco products. This modelling approach allowed for the use 752 data sources, integral to producing improved estimates by age, sex, and location, across which prevalence of chewing tobacco use varies widely. Finally, we compared trends in chewing tobacco with trends in smoking prevalence. The difference in trends over time between prevalences of chewing and smoking tobacco indicates that tobacco control efforts and policies have had a much larger effect on the prevalence of smoking tobacco use than on the prevalence of chewing tobacco use.

**Implications of all the available evidence**

Monitoring of prevalence of chewing tobacco use would benefit greatly from concerted efforts to add questions about its use in surveys that clearly distinguish the types of products, in a similar way to what is done for smoking tobacco. We found that the prevalence of chewing tobacco use has remained fairly stable over time and is high in many regions and demographic groups, including those with historically lower prevalence of smoking tobacco. Increased commitment to control of smokeless tobacco through both local interventions and expansion of the policies outlined in the WHO Framework Convention on Tobacco Control articles to smokeless tobacco products is urgently needed.

In this context, we aimed to provide an improved understanding of chewing tobacco use, which is essential for targeted policy, assessment of the effectiveness of these policies, and, ultimately, mitigation of the associated harms.^1^ Studies have been done previously that estimated prevalence for a particular country,^18^, ^19^, ^20^ region,^3^, ^4^, ^21^ or source,^3^, ^18^, ^19^, ^20^, ^21^ or a restricted time period^11^, ^20^, ^21^ or age group,^5^, ^22^ but to our knowledge no attempt has been made to synthesise multiple data sources to understand these trends globally over time and across age groups. For the first time, as part of the Global Burden of Diseases, Injuries, and Risk Factors Study (GBD) 2019, we comprehensively estimated the prevalence of chewing tobacco use using all available data sources to estimate age-sex-specific prevalence of chewing tobacco use from 1990 to 2019 in 204 countries and territories. We also compared these trends with those of smoked tobacco over the same time period.^23^ This manuscript was produced as part of the GBD Collaborator Network and in accordance with the GBD Protocol.

## Methods

### Overview and definitions

We modelled prevalence of current chewing tobacco use by using data on multiple types of smokeless tobacco use. We defined current chewing tobacco use as use of chewing tobacco products within the past 30 days on either a daily or occasional basis, or current use as defined by the survey. We produced estimates for males and females separately, and for each 5-year age group between the ages of 15 and 94 years with a terminal age group of individuals aged 95 years and older. We produced estimates for every year between 1990 and 2019 and for 204 countries and territories included in GBD 2019. This study adheres to the Guidelines for Accurate and Transparent Health Estimates Reporting.^24^

Because data on chewing tobacco alone are sparse, we systematically reviewed, extracted, and included in our estimations data on all types of smokeless tobacco. We classified data into three categories: chewing tobacco products only, non-chewing tobacco products only, and general smokeless tobacco with products not specified; we refer to this third category as unspecified smokeless tobacco. The first and second categories are distinct and do not overlap. Available data in these two categories were used to adjust data reported as general smokeless tobacco, which comprises the majority of data sources. As a result, in our modelling process we used information from all three categories to produce our final estimates of prevalence of chewing tobacco use for all countries.

### Data sources

We searched the Global Health Data Exchange for representative surveys with data on use of any smokeless tobacco product among individuals aged 10 years and older collected between 1980 and 2019. Although we report data for individuals aged 15 years and older and from 1990 onwards, we included this additional age group and decade to inform time trends and age patterns of the model. We included individual-level survey data, tabulated survey report data, and data from scientific literature. We identified and extracted data from 752 surveys that were location and year specific that met our inclusion criteria. Of 204 countries and territories, 185 (91%) had at least one data source and 58 (28%) had at least five data sources. 57 countries (28%) had their most recent data source from either 2017 or 2018. Full details on inclusion criteria, search strings, and extraction methods are included in the appendix (pp 12–15). A list of all included surveys can be accessed through the GBD 2019 Data Input Sources Tool.

### Modelling strategy and overview of spatiotemporal Gaussian process regression

A key challenge in modelling the prevalence of chewing tobacco use is that 562 (75%) of 752 sources with information on smokeless tobacco did not distinguish between specific smokeless tobacco products. Because this large proportion of sources reported on unspecified smokeless tobacco use, we used a modelling strategy that maximised the use of available information, rather than constraining our analysis to only focus on sources reporting the prevalence of chewing tobacco use alone. An overview of the modelling strategy, from data processing to final prevalence estimates, is shown in the appendix (p 23).

In three different parts of the estimation process, we used spatiotemporal Gaussian process regression (ST-GPR) to model location-age-sex-specific trends over time. Details on ST-GPR are described in full elsewhere.^25^ Briefly, the model is implemented in three steps: first, linear regression; second, spatiotemporal smoothing, which adjusts the linear regression estimate on the basis of residuals weighted by distance in time, age, and location; and finally, Gaussian process regression, which incorporates uncertainty in the data and quantifies the uncertainty of the estimates. For all ST-GPR models, we used an agnostic first-stage linear model that only includes a global intercept and age fixed effects. As a result, variations in final estimates by time, location, and age were entirely data driven and were incorporated in the second and third stages of the model. The information sharing across similar locations in ST-GPR is particularly useful in this context because the proportion of chewing tobacco use versus non-chewing tobacco use appears to be very similar within geographical regions.^11^ Additionally, we do not believe that these trends over age and time are substantially affected by different survey methods (appendix p 20). The end result of ST-GPR is 1000 draws from the posterior distribution of the Gaussian process, from which we calculated the mean and 2·5th and 97·5th percentiles to characterise the 95% uncertainty interval (UI).

### Smokeless tobacco product mapping and generation of the prevalence model

Case definitions varied substantially across data sources. Surveys reported on 262 unique combinations of smokeless tobacco products, which we mapped to one of two mutually exclusive and collectively exhaustive categories: either chewing tobacco products or non-chewing tobacco products. Non-chewing tobacco products refers to smokeless tobacco products that are not chewing tobacco. In some cases, surveys did not specify a product, or specified a wide array of products that spanned both categories. We mapped these sources to a third category of unspecified smokeless tobacco. The product map is in the appendix (p 15). After product mapping, 170 sources reported on the prevalence of chewing tobacco use, 137 reported on the prevalence of non-chewing tobacco use, and 690 reported on the prevalence of unspecified smokeless tobacco use.

After product mapping, 141 (19%) of 752 sources reported data only in aggregated age groups or as both sexes combined. We split these data into our standard 5-year age groups by sex. To do so, we ran separate ST-GPR models for each of the product categories (chewing tobacco, non-chewing tobacco, and unspecified smokeless tobacco), using only data originally available in our standard 5-year age groups and separately by sex. In these models, we purposefully tuned the parameter controlling the decay function for age weights in the spatiotemporal smoothing step to ensure that age patterns were data driven rather than model driven. We then used the modelled estimates to generate age and sex ratios that included uncertainty and varied by location and year. We applied these ratios to the data originally reported in aggregated age groups or as both sexes combined to split the aggregated data into our target demographic groups. Additional details on these methods are in the appendix (pp 16–17).

The proportion of unspecified smokeless tobacco that is chewing tobacco varies widely across countries. For example, in Sweden, snus (pulverised tobacco for sublabial administration, which we classify as non-chewing tobacco)^26^ is the predominant product used, while in India, most users of smokeless tobacco use chewing tobacco.^6^ To include data sources that report the prevalence of unspecified smokeless tobacco use, we needed an estimate of the proportion of unspecified smokeless tobacco that is chewing tobacco in each country.

To arrive at that proportion, first we ran separate models for chewing tobacco and non-chewing tobacco, using all available data for each indicator. Then, based on the results of these models, we estimated an age-sex-location-year-specific ratio of chewing tobacco as a proportion of chewing and non-chewing tobacco. Finally, we used this estimated ratio to adjust data reported as prevalence of unspecified smokeless tobacco use. We added the variance of the estimated ratio to the original variance of the data to reflect the uncertainty in this adjustment.

The final step in our modelling process was a ST-GPR model that included all data reported as prevalence of chewing tobacco use, and data reported as unspecified smokeless tobacco that have been adjusted on the basis of the estimated product type ratio. Because data variance is an input to ST-GPR, datapoints with higher variance had a lower influence on final estimates than did datapoints with a lower variance. As a result, the adjusted datapoints added information to the final model, but were weighted less in the final estimation than datapoints that were reported directly as prevalence of chewing tobacco use. Additional details of these methods are in the appendix (pp 18–20).

### Statistical analysis

We report the prevalence of chewing tobacco use and the number of people that currently use chewing tobacco, by location, year, age, and sex, as well as age-standardised estimates for individuals aged 15 years and older, all with their respective 95% UIs. Similarly we report prevalences by sex among individuals aged 15–19 years. We calculated annualised rates of change to assess time trends and compare changes across time with those observed for the prevalence of smoking tobacco use. We calculated all results (including annualised rates of change) that are reported as geographical aggregations using population-weighted aggregation. We determined annualised rates of change to be significant if the 95% UI did not include zero. We considered prevalence results to be significantly different if their 95% UIs did not intersect.

Details on modelling the prevalence of smoking tobacco use have been published separately.^23^ Additionally, we did a sensitivity analysis comparing the results of this main method (using both chewing tobacco and adjusted unspecified smokeless tobacco data) versus the results of using only the data on chewing tobacco (appendix pp 28–29).

We did all analyses using R (version 3.6.3).

### Role of the funding source

The funders of the study had no role in study design, data collection, data analysis, data interpretation, or writing of the report.

## Results

**Figure 1:**
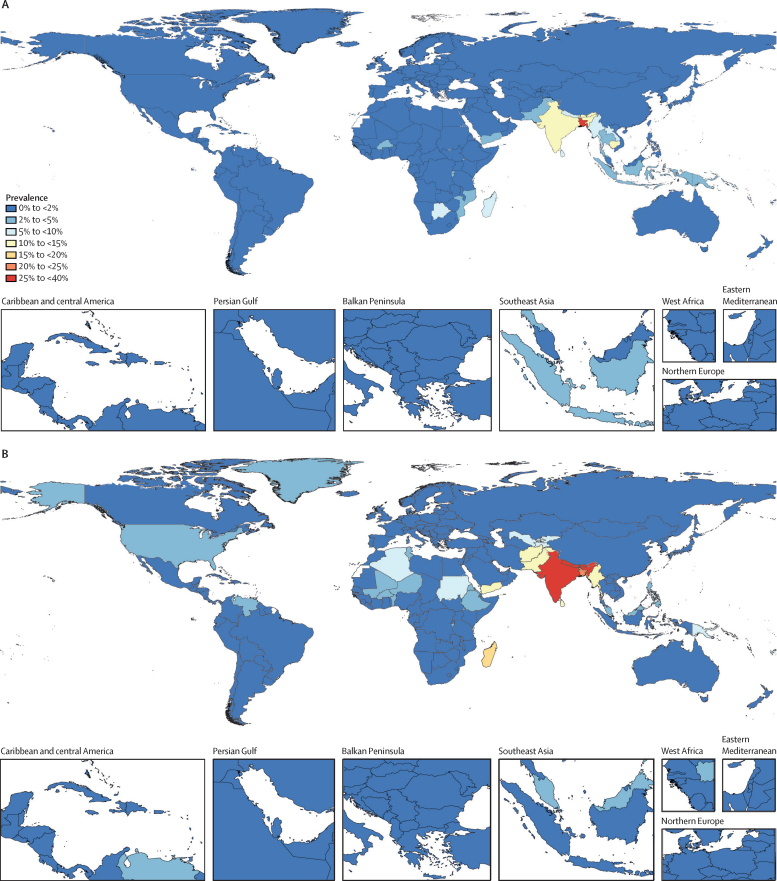
Age-standardised prevalence of chewing tobacco use in females (A) and males (B) aged 15 years and older, in 2019

Globally, 273·9 million (95% UI 258·5–290·9) people used chewing tobacco in 2019 (appendix pp 62–69). The global age-standardised prevalence of chewing tobacco use in 2019 among people aged 15 years and older was 4·72% (4·46–5·01) and was 6·55% (6·10–7·03) among males and 2·87% (2·60–3·14) among females (table). Most people (228·2 million [213·6–244·7]; 83·29% [82·15–84·42]) who used chewing tobacco in 2019 resided in the south Asia region. The largest populations of people who use chewing tobacco are in India (185·8 million [171·3–202·5] users; 67·83% [65·77–69·75] of global users) and Bangladesh (25·7 million [23·7–27·6]; 9·37% [8·59–10·25] of global users. Nepal, Bhutan, and Palau also had very high prevalences of chewing tobacco use in 2019, with 4·4 million (4·1–4·8) users in Nepal, 113 040 (102 587–123 860) in Bhutan, and 3440 (3090–3819) in Palau. Among males aged 15 years and older in 2019, the age-standardised prevalence in south Asia was 24·65% (22·81–26·69), while the lowest prevalence globally was 0·17% (0·15–0·20) in southern Latin America (figure 1; appendix p 70). Similarly, the age-standardised prevalence for females in south Asia was 12·13% (10·91–13·45) in 2019, much greater than the lowest age-standardised prevalence globally, which was in western Europe (0·15% [0·14–0·17]; figure 1; appendix p 69). Outside of the south Asia region, the countries with the highest prevalence of chewing tobacco use in 2019 were, for males, Palau (25·76% [22·37–29·75]), Madagascar (16·98% [14·66–19·30]), Myanmar (14·18% [11·94–16·53]), and Sri Lanka (13·57% [11·39–15·77]; figure 1; appendix pp 30–37). For females, the highest prevalence of use was observed in Palau (24·42% [20·04–29·17]), Cambodia (12·84% [11·05–14·70]), Laos (6·73% [5·31–8·24]), and Botswana (6·54% [5·32–7·92]; figure 1; appendix pp 30–37).

**Table:** Prevalence and annualised rate of change between 1990 and 2019 of current chewing tobacco use in the 12 locations with the highest age-standardised prevalence of chewing tobacco use in either 1990 or 2019, by sex

	**Females**					**Males**				
	Prevalence		Annualised rate of change			Prevalence		Annualised rate of change		
	1990	2019	1990–2005	2005–19	1990–2019	1990	2019	1990–2005	2005–19	1990–2019
Global	2·41% (2·14 to 2·74)	2·87% (2·60 to 3·14)	0·73% (−0·23 to 1·60)	0·46% (−0·36 to 1·27)	0·60% (0·04 to 1·11)	5·84% (5·31 to 6·40)	6·55% (6·10 to 7·03)	0·65% (−0·04 to 1·31)	0·12% (−0·48 to 0·76)	0·39% (−0·01 to 0·83)
Cambodia	14·57% (12·45 to 16·95)	12·84% (11·05 to 14·70)	0·45% (−0·58 to 1·48)	−1·38% (−2·49 to −0·33)	−0·43% (−1·22 to 0·30)	1·62% (1·34 to 1·96)	1·70% (1·39 to 2·07)	0·69% (−0·62 to 2·04)	−0·42% (−1·93 to 1·08)	0·15% (−0·73 to 1·10)
Myanmar	6·63% (5·23 to 8·43)	6·53% (5·14 to 8·20)	0·60% (−0·99 to 2·13)	−0·75% (−2·35 to 1·08)	−0·05% (−1·15 to 1·02)	13·51% (11·19 to 16·07)	14·18% (11·94 to 16·53)	1·24% (0·03 to 2·43)	−0·97% (−2·30 to 0·33)	0·17% (−0·62 to 1·06)
Sri Lanka	5·78% (4·53 to 7·25)	5·15% (4·05 to 6·39)	−0·05% (−1·62 to 1·52)	−0·78% (−2·51 to 0·85)	−0·40% (−1·51 to 0·66)	12·43% (10·39 to 14·73)	13·57% (11·39 to 15·77)	0·65% (−0·45 to 1·82)	−0·06% (−1·27 to 1·15)	0·30% (−0·45 to 1·10)
Marshall Islands	2·97% (2·06 to 4·28)	4·06% (2·98 to 5·40)	0·69% (−1·97 to 3·20)	1·55% (−1·23 to 4·17)	1·10% (−0·56 to 2·76)	9·14% (7·14 to 11·51)	10·36% (8·25 to 12·63)	0·52% (−1·14 to 2·25)	0·35% (−1·19 to 1·94)	0·44% (−0·66 to 1·55)
Yemen	5·94% (4·78 to 7·28)	4·35% (3·37 to 5·43)	0·55% (−0·95 to 2·24)	−2·83% (−4·44 to −1·19)	−1·08% (−2·17 to 0·03)	14·09% (11·85 to 16·60)	10·73% (9·02 to 12·83)	0·32% (−0·83 to 1·51)	−2·30% (−3·47 to −1·13)	−0·94% (−1·72 to −0·14)
Bangladesh	27·88% (24·37 to 31·73)	25·39% (23·11 to 27·78)	0·36% (−0·57 to 1·29)	−1·04% (−1·81 to −0·25)	−0·32% (−0·92 to 0·29)	21·86% (19·04 to 24·69)	21·98% (19·91 to 24·15)	0·32% (−0·59 to 1·23)	−0·30% (−1·14 to 0·53)	0·02% (−0·53 to 0·60)
Bhutan	13·76% (11·23 to 16·58)	14·22% (11·83 to 16·67)	0·10% (−1·23 to 1·40)	0·14% (−1·23 to 1·58)	0·12% (−0·75 to 0·99)	25·87% (22·11 to 29·77)	27·15% (24·12 to 30·09)	0·13% (−0·82 to 1·15)	0·21% (−0·81 to 1·21)	0·17% (−0·45 to 0·79)
India	11·79% (9·75 to 14·18)	11·53% (10·03 to 13·14)	0·09% (−1·26 to 1·39)	−0·24% (−1·47 to 1·00)	−0·07% (−0·87 to 0·74)	27·68% (24·33 to 31·24)	25·98% (23·64 to 28·52)	0·23% (−0·70 to 1·12)	−0·69% (−1·49 to 0·14)	−0·21% (−0·76 to 0·37)
Nepal	8·63% (6·88 to 10·43)	8·21% (6·64 to 9·86)	0·11% (−1·27 to 1·54)	−0·47% (−2·00 to 0·98)	−0·17% (−1·10 to 0·79)	37·05% (32·86 to 41·48)	39·16% (36·12 to 42·41)	0·29% (−0·48 to 1·06)	0·09% (−0·63 to 0·82)	0·19% (−0·27 to 0·71)
Pakistan	5·14% (4·07 to 6·56)	4·91% (3·94 to 6·01)	−0·12% (−1·69 to 1·33)	−0·19% (−1·81 to 1·40)	−0·15% (−1·18 to 0·86)	14·35% (11·96 to 16·96)	14·03% (12·11 to 16·22)	0·10% (−0·95 to 1·25)	−0·26% (−1·55 to 1·08)	−0·07% (−0·82 to 0·70)
Madagascar	6·29% (4·72 to 8·08)	6·38% (4·91 to 7·97)	0·73% (−0·99 to 2·48)	−0·66% (−2·49 to 1·42)	0·06% (−1·21 to 1·35)	16·19% (13·58 to 19·01)	16·98% (14·66 to 19·30)	1·06% (0·04 to 2·17)	−0·79% (−1·88 to 0·37)	0·17% (−0·59 to 0·94)
Palau	18·28% (14·60 to 22·43)	24·42% (20·04 to 29·17)	0·70% (−0·97 to 2·25)	1·33% (−0·24 to 2·79)	1·00% (0·03 to 1·98)	21·32% (17·86 to 25·14)	25·76% (22·37 to 29·75)	0·43% (−0·64 to 1·54)	0·90% (−0·25 to 2·15)	0·66% (−0·05 to 1·39)

Data are given to two decimal places. Data in parentheses are 95% uncertainty intervals. Countries are ordered according to GBD super-region and region. GBD=Global Burden of Diseases, Injuries, and Risk Factors Study.

Globally, prevalence of chewing tobacco use has increased slightly over time. The annualised rate of change between 1990 and 2019 for both sexes combined was 0·46% (95% UI 0·13 to 0·79), and was 0·39% (−0·01 to 0·83) for males and 0·60% (0·04 to 1·11) for females (appendix p 23). We identified high-prevalence locations by ranking the age-standardised prevalence of both sexes in 1990 and 2019. Here we concentrate on the 12 countries with the highest prevalence in either 1990 or 2019, or both. Within these countries, males in Yemen and females in Palau were the only demographic groups that had significant changes in prevalence between 1990 and 2019, with a significant decrease among males in Yemen (annualised rate of change –0·94% [–1·72 to –0·14]) and a significant increase among females in Palau (1·00% [0·03 to 1·98]; table). However, for these data on males in Yemen, further investigation is needed into the quality of the data due to conflict in this country during the study period.

**Figure 2:**
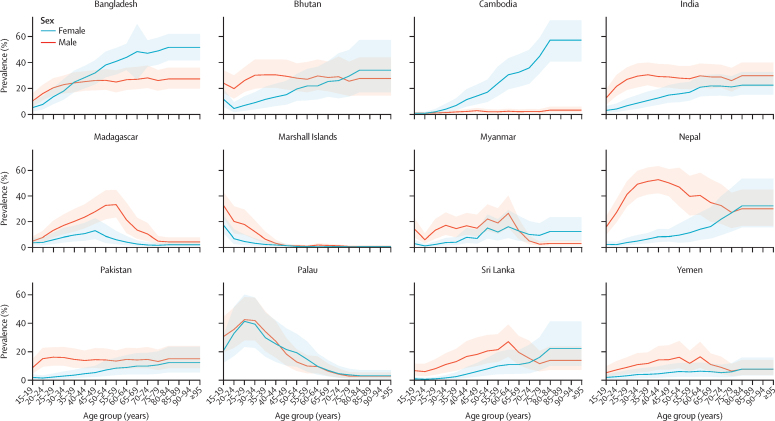
Age-sex pattern of prevalence of chewing tobacco use in 2019 among the 12 locations with the highest age-standardised prevalence of chewing tobacco use in either 1990 or 2019, or both The bold lines are prevalence estimates, with shaded areas indicating 95% uncertainty intervals.

Although temporal trends varied only slightly across these 12 countries, prevalence by age and sex differed much more. Globally in 2019, prevalence increased with age for females until age 80–84 years, after which it decreased, whereas for males prevalence increased up to age 35–39 years and then decreased in older age groups (appendix p 23). However, this global trend was not always reflected in the high-prevalence locations. In 2019, prevalence among males in the top 12 countries tended to decrease or flatten out in older age groups, with some countries observing peaks in prevalence in either young or middle-aged adults—eg, prevalence peaked at 52·73% (95% UI 42·13–63·31) in males aged 40–44 years in Nepal and at 42·29% (27·90–58·09) in males aged 25–29 years in Palau (figure 2). However, some countries had a more constant prevalence across age groups—eg, in Bangladesh, prevalence among males aged 20–24 years was 15·95% (10·88–22·62) and among males aged 80–84 years was 27·23% (19·80–35·93; figure 2). Among females, prevalence often increased into older age groups. In Cambodia and Sri Lanka, prevalence of chewing tobacco use increased in each age group, with prevalences of 0·83% (0·31–1·86) in those aged 20–24 years and 35·71% (23·57–49·63) in those aged 70–74 years in Cambodia, and 0·76% (0·29–1·66) in those aged 20–24 years, and 12·29% (4·89–24·71) in those aged 70–74 years in Sri Lanka (figure 2). Pakistan and Yemen had similar prevalences across age groups, whereas Madagascar and Palau had peaks in prevalence in females, among those aged 45–49 years in Madagascar (12·79% [6·40–22·50]) and among those aged 25–29 years in Palau (41·12% [23·31–59·80]; figure 2).

**Figure 3:**
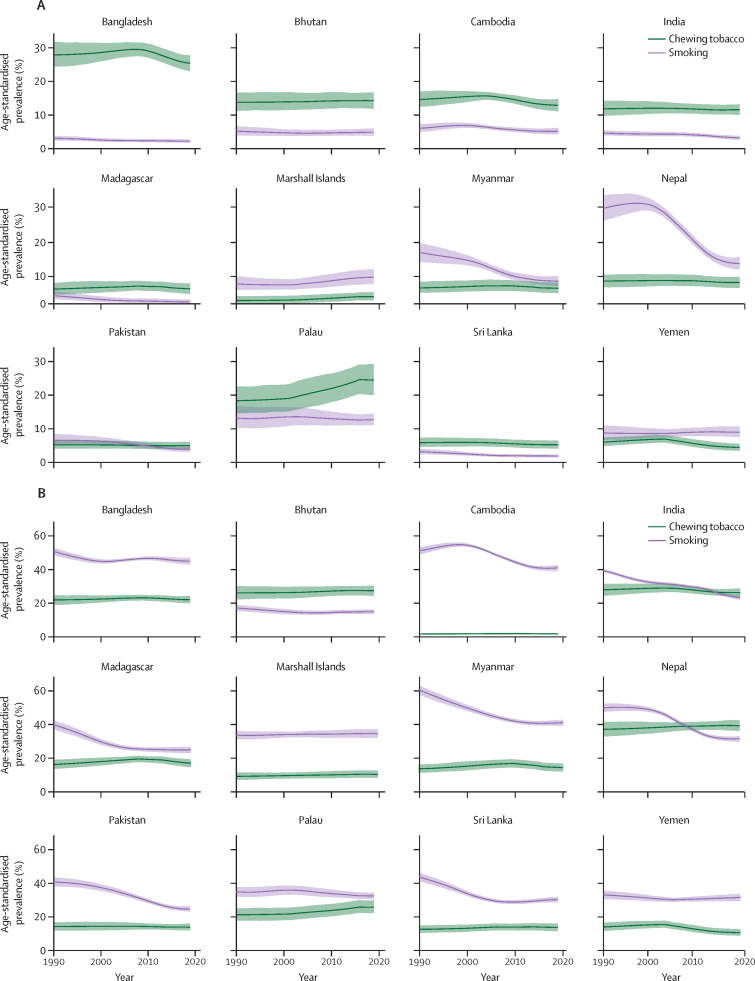
Age-standardised prevalence of chewing tobacco use versus prevalence of smoking among the 12 locations with the highest age-standardised prevalence of both-sex chewing tobacco use, in females (A) and males (B), in 1990–2019 Bold lines are prevalence estimates, with shaded areas showing the 95% uncertainty intervals.

Prevalence of chewing tobacco use was often quite high at young ages. In 2019, 126 (62%) of 204 locations had higher prevalence among males aged 15–19 years than the age-standardised prevalence for males older than 19 years; among females, 135 (66%) locations had a higher prevalence among those aged 15–19 years than the age-standardised prevalence for females older than 19 years. For both sexes combined, seven locations—Marshall Islands, Federated States of Micronesia, Papua New Guinea, Bhutan, Guam, Northern Mariana Islands, and Palau—had prevalences of more than 10% in this age group (appendix pp 46–61). In 2019, south Asia and Oceania were the regions with the highest prevalence among people aged 15–19 years (figure 3; appendix p 29). Among males aged 15–19 years, the Marshall Islands had the highest prevalence of chewing tobacco use in 2019, at 32·50% (95% UI 22·82–42·74). Palau (30·55% [19·63–44·26]), Federated States of Micronesia (28·91% [19·52–39·75]), Northern Mariana Islands (27·78% [18·02–40·14]), and Bhutan (23·97% [16·34–33·82]) comprise the other top five countries for males in this age group (appendix pp 29, 43–58). The list is similar among females; the Federated States of Micronesia had the highest prevalence (22·55% [13·68–33·76]), with Northern Mariana Islands (21·62% [12·36–34·12]), Palau (20·85% [12·24–31·53]), Marshall Islands (17·04% [10·97–24·66]), and Papua New Guinea (13·24% [7·45–20·57]) comprising the rest of the top five countries among females in this age group (appendix pp 29, 43–58).

Unlike the prevalence of chewing tobacco use, **t**he global age-standardised prevalence of smoking decreased significantly between 1990 and 2019 (annualised rate of change: –1·21% [95% UI –1·26 to –1·16]). Among females in 2019, chewing tobacco use was more common than smoking tobacco use in eight of 12 countries, and in individuals aged 15–19 years in six of 12 countries. Similarly, among males in 2019, prevalence of chewing tobacco use was higher than smoking tobacco use in three of 12 countries and in individuals aged 15–19 years in four of 12 countries (appendix pp 24–27, 46–61).

Between 1990 and 2019, among the 12 countries with the highest prevalence of chewing tobacco use, nine had significant decreases in prevalence of smoking among males and seven had significant decreases among females (appendix pp 38–45). Among males in Nepal and India, the prevalence of chewing tobacco use surpassed the prevalence of smoking tobacco use in the past 5–10 years (figure 3). Among females, the difference in prevalence of chewing tobacco use versus prevalence of smoking tobacco use varied substantially by country (figure 3). For example, for females in Nepal and Myanmar, smoking prevalence decreased significantly over 1990–2019, whereas the prevalence of chewing tobacco use was stable over this period. In Madagascar and Palau, the prevalence of chewing tobacco use among females surpassed the prevalence of smoking tobacco in the past decade (figure 3).

Two countries in 2019 had a higher prevalence of chewing tobacco use than of smoking tobacco use among people aged 15–19 years. Among males in 2019, Uzbekistan had a significantly higher prevalence of chewing tobacco use than of smoking tobacco use (12·68% [95% UI 6·92–21·30] *vs* 1·65% [1·03–2·49]; 184 995 [100 912–310 674] chewing tobacco users *vs* 24 044 [14 985–36 351] smokers; appendix pp 46–61). Among females, Bangladesh had significantly higher prevalence of chewing tobacco use than smoking tobacco use (5·08% [2·24–10·01] *vs* 1·06% [0·42–2·15]; 402 545 [177 719–793 059] chewing tobacco users *vs* 83 927 [33 649–162 605] smokers; appendix pp 46–61).

We did a sensitivity analysis to compare our final model to a model that only used chewing tobacco data (no adjusted unspecified smokeless tobacco data). Overall, the correlation of the two estimates was 0·821, and on average our final model was 0·83 percentage points lower globally than when just using the chewing tobacco data. Additional comparisons are provided in the appendix (pp 28–29).

## Discussion

In 2019, 273·9 million (95% UI 258·5–290·9) people used chewing tobacco, and age-standardised prevalence for people aged 15 and older was 4·72% (4·46–5·01). 83·29% of chewing tobacco users live in south Asia, with 185·8 million chewing tobacco users residing in India. Other countries with high prevalence of chewing tobacco use include Palau, Bangladesh, and Nepal, which together had 30·1 million chewing tobacco users in 2019. A major concern emerging from our analyses is that the prevalence of chewing tobacco use has remained constant and high. Of the 12 countries with the highest prevalences of chewing tobacco use, 11 had no significant decreases in prevalence of chewing tobacco use among males, whereas nine had significant decreases in prevalence of smoking tobacco use among males, and no countries had a significant decrease in prevalence of chewing tobacco use among females, whereas seven had significant decreases in prevalence of smoking tobacco use among females. Among females in 2019, we found that use of chewing tobacco was more common than smoking in eight of the 12 highest prevalence countries for people aged 15 years and older, and among just those aged 15–19 years in six of 12 countries. Among males, prevalence of chewing tobacco use was higher than smoking in three of 12 countries and in individuals aged 15–19 years in four of 12 countries. The serious adverse health effects resulting from chewing tobacco use^11^, ^12^, ^15^, ^16^, ^17^ necessitate stronger regulations and policies than are currently in place, particularly in countries with persistently high prevalence.

Much of the previous research on the prevalence of chewing tobacco use has focused on a particular country,^18^, ^19^, ^20^ region,^3^, ^4^, ^21^ source,^3^, ^18^, ^19^, ^20^, ^21^ time period,^11^, ^20^, ^21^ or age group,^5^, ^22^ making formation of a comprehensive global picture of where chewing tobacco is used most, among which age groups and sexes, and how these trends have changed over time very difficult. Our study is a step towards understanding this full picture so that policy makers, public health officials, and advocacy organisations have access to a full set of comparable estimates for use in addressing this harmful substance. Additionally, our aim to combine multiple sources and definitions of smokeless tobacco use across 204 locations has highlighted data synthesis issues due to definition variations and differences in data granularity that should be addressed in future surveillance of smokeless tobacco use.

Underscoring the importance of strengthening control on use of chewing tobacco, we found that countries with high use of chewing tobacco had almost no change in prevalence between 1990 and 2019, whereas several of these locations had significant decreases in smoking prevalence during the same period. This finding is especially true among females, in whom the prevalence of chewing tobacco use was often close to, if not larger than, the prevalence of smoking. This trend in high-prevalence countries has been noted previously,^27^ but our findings highlight this association over an extended time period and across the sexes. These findings might be due to a combination of factors, including less widespread application of the WHO FCTC articles on chewing tobacco,^2^, ^28^ complex cultural reasons such as wider social acceptability and beliefs about associated benefits,^6^, ^7^, ^8^ and targeted advertising.^29^ Cambodia stands out as a place where the prevalence of chewing tobacco use is very high among females, particularly in comparison with among males, perhaps due to a variety of different environmental and cultural reasons.^30^ Because the prevalence of chewing tobacco use nears or surpasses the prevalence of smoking tobacco use in some countries, efforts must be intensified and the scope of tobacco control be expanded to explicitly address smokeless tobacco products.

Our findings also call attention to chewing tobacco use among adolescents, because seven countries had prevalences of more than 10% among people aged 15–19 years in 2019. Additionally, as observed among females, some locations with high chewing tobacco use among young people also had significantly higher prevalences of chewing tobacco use than of smoking tobacco use in 2019. These locations might be emerging markets for chewing tobacco, which should be reflected in how these countries enact the FCTC articles. Initiation of use during youth, consumption, and patterns of use should also continue to be studied.

Our findings should be considered in the context of the limitations of the study. First, we did not quantify dual use of chewing tobacco and smoking, which is important to understand for both policy setting and burden implications.^31^ Tracking and understanding dual use might be important to uncover potential issues with targeted advertising, differential cessation success, or particularly problematic adolescent use.^31^, ^32^ Second, our study relies on self-reported data and reporting biases might be present that vary across age groups, sexes, geographical regions, and socioeconomic statuses. The nature of these biases is not yet known, and previous studies indicate mixed scale and scope.^33^, ^34^, ^35^ However, because we measured prevalence and not amount of chewing tobacco used, under-reporting of smokeless tobacco use is unlikely to affect these results to a large degree. Third, although we aimed to better address the main limitation of modelling the prevalence of chewing tobacco use—a combination of data sparsity and compositional bias in survey questions across locations—higher quality data would improve our estimates and ensure that the location-year-age-sex ratios we used to adjust the unspecified smokeless tobacco data are accurate. Surveys that ask about locally relevant products would aid in providing estimates that are more precise and rely less on smoothing across age, time, and location. Improved questionnaires could also allow for analyses further differentiated by smokeless tobacco subtype or local products, or both, which would be beneficial for policy making. For example, a handful of Indian states banned gutkha (chewing tobacco preparation including betel nut) in 2013, which some studies have shown might have led people to purchase other types of smokeless tobacco,^8^, ^36^ which cannot be captured in the current study. Additionally, both more granular data and additional data sources can help to avoid any instances where trends across age groups and over time are caused by different survey methods, although we do not believe that this limitation would substantially change our findings of this study. Future work should explore disaggregation by other subgroups beyond age and sex. For example, previous studies have shown that socioeconomic status, educational attainment, and urbanicity might affect smokeless tobacco use.^3^ Additionally, subnational analysis will be important for future studies, because local evidence is crucial to local policy setting, and previous studies^18^, ^37^ have shown wide variation in the prevalence of chewing tobacco use within some countries. Specifically, an analysis of chewing tobacco use across subnational units in India should be prioritised.^38^, ^39^

Chewing tobacco use continues to persist even with many countries' commitment to the WHO FCTC articles, and there is a large opportunity for policies and programmes to better target the use of these products. In the absence of stronger policies that are effectively implemented, these trends might stay the same as they have in the past. Additionally, the popularity of these products among adolescents, especially in places where prevalence of smoking has not historically been high, indicates the potential for these products to gain users in locations that do not currently have high use of chewing tobacco. Even as countries face competing political priorities and challenges from the tobacco industry, increased expansion, implementation, and enforcement of the WHO FCTC articles for smokeless tobacco in addition to locally targeted policies is integral to stemming the chewing tobacco epidemic.

For the **GBD 2019 Data Input Sources Tool** see http://ghdx.healthdata.org/gbd-2019/data-input-sources

For the **Global Health Data Exchange** see http://ghdx.healthdata.org/

For the **Global Health Data Exchange** see http://ghdx.healthdata.org

**This online publication has been corrected. The corrected version first appeared at thelancet.com/public-health on June 2, 2021**

## Data sharing

To download the data used in these analyses, please visit the Global Health Data Exchange GBD 2019 website.

## Declaration of interests

ViA reports personal fees from Bayer Healthcare, Boehringer Ingelheim/Lilly alliance, Bristol Myers Squibb/Pfizer alliance, and Novo Nordisk outside of the submitted work. RA reports consultancy and speakers' fees from UCB, Sandoz, AbbVie, Zentiva, Teva, Laropharm, Cegedim, Angelini, B Braun, Biessen Pharma, Hofigal, AstraZeneca, and Stada. BA reports personal fees from Australian Institute of Sports, grants and non-financial support from Natural Remedies, and non-financial support from Zydus Cadila outside of the submitted work. SI reports grants from National Heart Foundation of Australia, and Australian National Health and Medical Research Council outside the submitted work. KK reports non-financial support from UGC Centre of Advanced Study (CAS II), Department of Anthropology, Panjab University, Chandigarh, India, outside of the submitted work. TRM reports contracts from Gov't Plaintiff Lawyers, JUUL, outside of the submitted work. JAS reports consultancy fees from Crealta/Horizon, Medisys, Fidia, Two Labs Inc, Adept Field Solutions, Clinical Care Options, Clearview Healthcare Partners, Putnam Associates, Focus Forward, Navigant Consulting, Spherix, MedIQ, UBM LLC, Trio Health, Medscape, WebMD, Practice Point Communications, and the National Institutes of Health and the American College of Rheumatology; receives payment for lectures as a member on the speaker's bureau of Simply Speaking; owns stock options in TPT Global Tech, Vaxart pharmaceuticals, and Charlotte's Web Holdings; previously owned stock options in Amarin, Viking, and Moderna pharmaceuticals; held a placement on the steering committee of OMERACT, an international organisation that develops measures for clinical trials and receives arm's length funding from 12 pharmaceutical companies; serves on the US Food and Drug Administration Arthritis Advisory Committee; is a member of the Veterans Affairs Rheumatology Field Advisory Committee; is the editor and is the Director of the University of Alabama at Birmingham Cochrane Musculoskeletal Group Satellite Center on Network Meta-analysis, all outside of the submitted work. JS reports ownership of companies providing services to Itrim, Amgen, Janssen, Novo Nordisk, Eli Lilly, Boehringer Ingelheim, Bayer, Pfizer, and AstraZeneca outside of the submitted work. DJS reports personal fees from Lundbeck, Takeda, Johnson & Johnson, and Servier outside of the submitted work. SS reports grants from Edwards Lifesciences, Medtronic, Boston Scientific, and Abbott; and personal fees from Boston Scientific, Teleflex, and BTG outside of the submitted work. All other authors declare no competing interests.
