# Correction to *Lancet Public Health* 2021; published online May 27. https://doi.org/10.1016/S2468-2667(21)00065-7

**DOI:** 10.1016/S2468-2667(21)00132-8

**Published:** 2021-06-03

*GBD 2019 Tobacco Collaborators. Spatial, temporal, and demographic patterns in prevalence of chewing tobacco use in 204 countries and territories, 1990–2019: a systematic analysis from the Global Burden of Disease Study 2019.* Lancet Public Health *2021; published online May 27. https://doi.org/10.1016/S2468-2667(21)00065-7*—In this Article, the GBD 2019 Chewing Tobacco Collaborators list and accompanying affiliations have been updated. These corrections have been made to the online version as of June 2, 2021.

