# Supplementary material for: Spatial, temporal, and demographic patterns in prevalence of chewing tobacco use in 204 countries and territories, 1990–2019: a systematic analysis from the Global Burden of Disease Study 2019
Source: Lancet Public Health. 2021 May 28;6(7):e482–99. doi: 10.1016/S2468-2667(21)00065-7 (PMC8251505; doi:10.1016/S2468-2667(21)00065-7)
Supplement: Supplementary appendix [file mmc1.pdf]

THE LANCET
Public Health

Supplementary appendix

This appendix formed part of the original submission and has been peer reviewed. We post it as supplied by the authors.

Supplement to: GBD 2019 Chewing Tobacco Collaborators. Spatial, temporal, and demographic patterns in prevalence of chewing tobacco use in 204 countries and territories, 1990–2019: a systematic analysis from the Global Burden of Disease Study 2019. *Lancet Public Health* 2021; published online May 27. http://dx.doi.org/10.1016/S2468-2667(21)00065-7.

Supplementary Methods

Spatial, temporal, and demographic patterns in chewing tobacco prevalence, 1990-2019: a systematic analysis of 204 countries and territories using new methods from the Global Burden of Disease Study 2019

GBD 2019 Chewing Tobacco Collaborators

Contents

Table of Common Abbreviations 4
Geographies Estimated 5
Demographics 11
Exposure 12
Case Definitions 12
Inclusion Criteria 12
Data extraction 12
Mapping definitions 15
Age and Sex Splitting 16
Chewing Tobacco Prevalence Modelling 18
Supplementary Figures and Tables 22
Supplementary Figure 1. 23
Supplementary Figure 2. 23
Supplementary Figure 3. 24
Supplementary Figure 4. 26
Supplementary Figure 5. 28
Supplementary Figure 6. 29
Supplementary Table 1. 30
Supplementary Table 2. 38
Supplementary Table 3. 46
Supplementary Table 4. 62
Supplementary Table 5. 70
Authors' Contributions 71
Managing the estimation or publications process 71
Writing the first draft of the manuscript 71
Primary responsibility for applying analytical methods to produce estimates 71
Primary responsibility for seeking, cataloguing, extracting, or cleaning data; designing or coding figures and tables 71
Providing data or critical feedback on data sources 71
Developing methods or computational machinery 72
Providing critical feedback on methods or results 72
Drafting the work or revising is critically for important intellectual content 74

Extracting, cleaning, or cataloging data; designing or coding figures and tables 75
Managing the overall research enterprise 75

Table of Common Abbreviations

Abbreviation	Definition
WHO	World Health Organization
UI	Uncertainty Interval
FCTC	Framework Convention on Tobacco Control
SLT	Unspecified smokeless tobacco (includes both chewing and non-chewing products)
GATHER	Guidelines for Accurate and Transparent Health Estimates Reporting
ST-GPR	Spatio-temporal Gaussian process regression

Geographies Estimated

For the Global Burden of Disease Study 2019, we produced estimates for 204 countries and territories (Table S1). These countries and territories were nested in 21 aggregate regions and seven aggregate super-regions.

Table S1. Geographic Hierarchy

Location	Super-Region	Region
**Armenia**	Central Europe, Eastern Europe, and Central Asia	Central Asia
**Azerbaijan**	Central Europe, Eastern Europe, and Central Asia	Central Asia
**Georgia**	Central Europe, Eastern Europe, and Central Asia	Central Asia
**Kazakhstan**	Central Europe, Eastern Europe, and Central Asia	Central Asia
**Kyrgyzstan**	Central Europe, Eastern Europe, and Central Asia	Central Asia
**Mongolia**	Central Europe, Eastern Europe, and Central Asia	Central Asia
**Tajikistan**	Central Europe, Eastern Europe, and Central Asia	Central Asia
**Turkmenistan**	Central Europe, Eastern Europe, and Central Asia	Central Asia
**Uzbekistan**	Central Europe, Eastern Europe, and Central Asia	Central Asia
**Albania**	Central Europe, Eastern Europe, and Central Asia	Central Europe
**Bosnia and Herzegovina**	Central Europe, Eastern Europe, and Central Asia	Central Europe
**Bulgaria**	Central Europe, Eastern Europe, and Central Asia	Central Europe
**Croatia**	Central Europe, Eastern Europe, and Central Asia	Central Europe
**Czechia**	Central Europe, Eastern Europe, and Central Asia	Central Europe
**Hungary**	Central Europe, Eastern Europe, and Central Asia	Central Europe
**Montenegro**	Central Europe, Eastern Europe, and Central Asia	Central Europe
**North Macedonia**	Central Europe, Eastern Europe, and Central Asia	Central Europe
**Poland**	Central Europe, Eastern Europe, and Central Asia	Central Europe
**Romania**	Central Europe, Eastern Europe, and Central Asia	Central Europe
**Serbia**	Central Europe, Eastern Europe, and Central Asia	Central Europe
**Slovakia**	Central Europe, Eastern Europe, and Central Asia	Central Europe
**Slovenia**	Central Europe, Eastern Europe, and Central Asia	Central Europe
**Belarus**	Central Europe, Eastern Europe, and Central Asia	Eastern Europe
**Estonia**	Central Europe, Eastern Europe, and Central Asia	Eastern Europe
**Latvia**	Central Europe, Eastern Europe, and Central Asia	Eastern Europe
**Lithuania**	Central Europe, Eastern Europe, and Central Asia	Eastern Europe
**Republic of Moldova**	Central Europe, Eastern Europe, and Central Asia	Eastern Europe
**Russian Federation**	Central Europe, Eastern Europe, and Central Asia	Eastern Europe

**Ukraine**	Central Europe, Eastern Europe, and Central Asia	Eastern Europe
**Australia**	High-income	Australasia
**New Zealand**	High-income	Australasia
**Brunei Darussalam**	High-income	High-income Asia Pacific
**Japan**	High-income	High-income Asia Pacific
**Republic of Korea**	High-income	High-income Asia Pacific
**Singapore**	High-income	High-income Asia Pacific
**Canada**	High-income	High-income North America
**Greenland**	High-income	High-income North America
**United States of America**	High-income	High-income North America
**Argentina**	High-income	Southern Latin America
**Chile**	High-income	Southern Latin America
**Uruguay**	High-income	Southern Latin America
**Andorra**	High-income	Western Europe
**Austria**	High-income	Western Europe
**Belgium**	High-income	Western Europe
**Cyprus**	High-income	Western Europe
**Denmark**	High-income	Western Europe
**Finland**	High-income	Western Europe
**France**	High-income	Western Europe
**Germany**	High-income	Western Europe
**Greece**	High-income	Western Europe
**Iceland**	High-income	Western Europe
**Ireland**	High-income	Western Europe
**Israel**	High-income	Western Europe
**Italy**	High-income	Western Europe
**Luxembourg**	High-income	Western Europe
**Malta**	High-income	Western Europe
**Monaco**	High-income	Western Europe
**Netherlands**	High-income	Western Europe
**Norway**	High-income	Western Europe
**Portugal**	High-income	Western Europe
**San Marino**	High-income	Western Europe
**Spain**	High-income	Western Europe
**Sweden**	High-income	Western Europe
**Switzerland**	High-income	Western Europe
**United Kingdom**	High-income	Western Europe
**Bolivia (Plurinational State of)**	Latin America and Caribbean	Andean Latin America
**Ecuador**	Latin America and Caribbean	Andean Latin America

**Peru**	Latin America and Caribbean	Andean Latin America
**Antigua and Barbuda**	Latin America and Caribbean	Caribbean
**Bahamas**	Latin America and Caribbean	Caribbean
**Barbados**	Latin America and Caribbean	Caribbean
**Belize**	Latin America and Caribbean	Caribbean
**Bermuda**	Latin America and Caribbean	Caribbean
**Cuba**	Latin America and Caribbean	Caribbean
**Dominica**	Latin America and Caribbean	Caribbean
**Dominican Republic**	Latin America and Caribbean	Caribbean
**Grenada**	Latin America and Caribbean	Caribbean
**Guyana**	Latin America and Caribbean	Caribbean
**Haiti**	Latin America and Caribbean	Caribbean
**Jamaica**	Latin America and Caribbean	Caribbean
**Puerto Rico**	Latin America and Caribbean	Caribbean
**Saint Kitts and Nevis**	Latin America and Caribbean	Caribbean
**Saint Lucia**	Latin America and Caribbean	Caribbean
**Saint Vincent and the Grenadines**	Latin America and Caribbean	Caribbean
**Suriname**	Latin America and Caribbean	Caribbean
**Trinidad and Tobago**	Latin America and Caribbean	Caribbean
**United States Virgin Islands**	Latin America and Caribbean	Caribbean
**Colombia**	Latin America and Caribbean	Central Latin America
**Costa Rica**	Latin America and Caribbean	Central Latin America
**El Salvador**	Latin America and Caribbean	Central Latin America
**Guatemala**	Latin America and Caribbean	Central Latin America
**Honduras**	Latin America and Caribbean	Central Latin America
**Mexico**	Latin America and Caribbean	Central Latin America
**Nicaragua**	Latin America and Caribbean	Central Latin America
**Panama**	Latin America and Caribbean	Central Latin America
**Venezuela (Bolivarian Republic of)**	Latin America and Caribbean	Central Latin America
**Brazil**	Latin America and Caribbean	Tropical Latin America
**Paraguay**	Latin America and Caribbean	Tropical Latin America
**Afghanistan**	North Africa and Middle East	North Africa and Middle East
**Algeria**	North Africa and Middle East	North Africa and Middle East
**Bahrain**	North Africa and Middle East	North Africa and Middle East
**Egypt**	North Africa and Middle East	North Africa and Middle East
**Iran (Islamic Republic of)**	North Africa and Middle East	North Africa and Middle East
**Iraq**	North Africa and Middle East	North Africa and Middle East
**Jordan**	North Africa and Middle East	North Africa and Middle East

**Kuwait**	North Africa and Middle East	North Africa and Middle East
**Lebanon**	North Africa and Middle East	North Africa and Middle East
**Libya**	North Africa and Middle East	North Africa and Middle East
**Morocco**	North Africa and Middle East	North Africa and Middle East
**Oman**	North Africa and Middle East	North Africa and Middle East
**Palestine**	North Africa and Middle East	North Africa and Middle East
**Qatar**	North Africa and Middle East	North Africa and Middle East
**Saudi Arabia**	North Africa and Middle East	North Africa and Middle East
**Sudan**	North Africa and Middle East	North Africa and Middle East
**Syrian Arab Republic**	North Africa and Middle East	North Africa and Middle East
**Tunisia**	North Africa and Middle East	North Africa and Middle East
**Turkey**	North Africa and Middle East	North Africa and Middle East
**United Arab Emirates**	North Africa and Middle East	North Africa and Middle East
**Yemen**	North Africa and Middle East	North Africa and Middle East
**Bangladesh**	South Asia	South Asia
**Bhutan**	South Asia	South Asia
**India**	South Asia	South Asia
**Nepal**	South Asia	South Asia
**Pakistan**	South Asia	South Asia
**China**	Southeast Asia, East Asia, and Oceania	East Asia
**Democratic People's Republic of Korea**	Southeast Asia, East Asia, and Oceania	East Asia
**Taiwan (Province of China)**	Southeast Asia, East Asia, and Oceania	East Asia
**American Samoa**	Southeast Asia, East Asia, and Oceania	Oceania
**Cook Islands**	Southeast Asia, East Asia, and Oceania	Oceania
**Fiji**	Southeast Asia, East Asia, and Oceania	Oceania
**Guam**	Southeast Asia, East Asia, and Oceania	Oceania
**Kiribati**	Southeast Asia, East Asia, and Oceania	Oceania
**Marshall Islands**	Southeast Asia, East Asia, and Oceania	Oceania
**Micronesia (Federated States of)**	Southeast Asia, East Asia, and Oceania	Oceania
**Nauru**	Southeast Asia, East Asia, and Oceania	Oceania
**Niue**	Southeast Asia, East Asia, and Oceania	Oceania
**Northern Mariana Islands**	Southeast Asia, East Asia, and Oceania	Oceania
**Palau**	Southeast Asia, East Asia, and Oceania	Oceania
**Papua New Guinea**	Southeast Asia, East Asia, and Oceania	Oceania
**Samoa**	Southeast Asia, East Asia, and Oceania	Oceania
**Solomon Islands**	Southeast Asia, East Asia, and Oceania	Oceania
**Tokelau**	Southeast Asia, East Asia, and Oceania	Oceania
**Tonga**	Southeast Asia, East Asia, and Oceania	Oceania

**Tuvalu**	Southeast Asia, East Asia, and Oceania	Oceania
**Vanuatu**	Southeast Asia, East Asia, and Oceania	Oceania
**Cambodia**	Southeast Asia, East Asia, and Oceania	Southeast Asia
**Indonesia**	Southeast Asia, East Asia, and Oceania	Southeast Asia
**Laos**	Southeast Asia, East Asia, and Oceania	Southeast Asia
**Malaysia**	Southeast Asia, East Asia, and Oceania	Southeast Asia
**Maldives**	Southeast Asia, East Asia, and Oceania	Southeast Asia
**Mauritius**	Southeast Asia, East Asia, and Oceania	Southeast Asia
**Myanmar**	Southeast Asia, East Asia, and Oceania	Southeast Asia
**Philippines**	Southeast Asia, East Asia, and Oceania	Southeast Asia
**Seychelles**	Southeast Asia, East Asia, and Oceania	Southeast Asia
**Sri Lanka**	Southeast Asia, East Asia, and Oceania	Southeast Asia
**Thailand**	Southeast Asia, East Asia, and Oceania	Southeast Asia
**Timor-Leste**	Southeast Asia, East Asia, and Oceania	Southeast Asia
**Viet Nam**	Southeast Asia, East Asia, and Oceania	Southeast Asia
**Angola**	Sub-Saharan Africa	Central Sub-Saharan Africa
**Central African Republic**	Sub-Saharan Africa	Central Sub-Saharan Africa
**Congo**	Sub-Saharan Africa	Central Sub-Saharan Africa
**Democratic Republic of the Congo**	Sub-Saharan Africa	Central Sub-Saharan Africa
**Equatorial Guinea**	Sub-Saharan Africa	Central Sub-Saharan Africa
**Gabon**	Sub-Saharan Africa	Central Sub-Saharan Africa
**Burundi**	Sub-Saharan Africa	Eastern Sub-Saharan Africa
**Comoros**	Sub-Saharan Africa	Eastern Sub-Saharan Africa
**Djibouti**	Sub-Saharan Africa	Eastern Sub-Saharan Africa
**Eritrea**	Sub-Saharan Africa	Eastern Sub-Saharan Africa
**Ethiopia**	Sub-Saharan Africa	Eastern Sub-Saharan Africa
**Kenya**	Sub-Saharan Africa	Eastern Sub-Saharan Africa
**Madagascar**	Sub-Saharan Africa	Eastern Sub-Saharan Africa
**Malawi**	Sub-Saharan Africa	Eastern Sub-Saharan Africa
**Mozambique**	Sub-Saharan Africa	Eastern Sub-Saharan Africa
**Rwanda**	Sub-Saharan Africa	Eastern Sub-Saharan Africa
**Somalia**	Sub-Saharan Africa	Eastern Sub-Saharan Africa
**South Sudan**	Sub-Saharan Africa	Eastern Sub-Saharan Africa
**Uganda**	Sub-Saharan Africa	Eastern Sub-Saharan Africa
**United Republic of Tanzania**	Sub-Saharan Africa	Eastern Sub-Saharan Africa
**Zambia**	Sub-Saharan Africa	Eastern Sub-Saharan Africa
**Botswana**	Sub-Saharan Africa	Southern Sub-Saharan Africa
**Eswatini**	Sub-Saharan Africa	Southern Sub-Saharan Africa

**Lesotho**	Sub-Saharan Africa	Southern Sub-Saharan Africa
**Namibia**	Sub-Saharan Africa	Southern Sub-Saharan Africa
**South Africa**	Sub-Saharan Africa	Southern Sub-Saharan Africa
**Zimbabwe**	Sub-Saharan Africa	Southern Sub-Saharan Africa
**Benin**	Sub-Saharan Africa	Western Sub-Saharan Africa
**Burkina Faso**	Sub-Saharan Africa	Western Sub-Saharan Africa
**Cabo Verde**	Sub-Saharan Africa	Western Sub-Saharan Africa
**Cameroon**	Sub-Saharan Africa	Western Sub-Saharan Africa
**Chad**	Sub-Saharan Africa	Western Sub-Saharan Africa
**Cote d'Ivoire**	Sub-Saharan Africa	Western Sub-Saharan Africa
**Gambia**	Sub-Saharan Africa	Western Sub-Saharan Africa
**Ghana**	Sub-Saharan Africa	Western Sub-Saharan Africa
**Guinea**	Sub-Saharan Africa	Western Sub-Saharan Africa
**Guinea-Bissau**	Sub-Saharan Africa	Western Sub-Saharan Africa
**Liberia**	Sub-Saharan Africa	Western Sub-Saharan Africa
**Mali**	Sub-Saharan Africa	Western Sub-Saharan Africa
**Mauritania**	Sub-Saharan Africa	Western Sub-Saharan Africa
**Niger**	Sub-Saharan Africa	Western Sub-Saharan Africa
**Nigeria**	Sub-Saharan Africa	Western Sub-Saharan Africa
**Sao Tome and Principe**	Sub-Saharan Africa	Western Sub-Saharan Africa
**Senegal**	Sub-Saharan Africa	Western Sub-Saharan Africa
**Sierra Leone**	Sub-Saharan Africa	Western Sub-Saharan Africa
**Togo**	Sub-Saharan Africa	Western Sub-Saharan Africa

Demographics

For the 204 countries and territories included in analysis, we produced estimates by sex and five-year age group from 1990 to 2019. Additionally, we produced estimates aggregated by age:

- Age-standardized prevalence reflects age-weighted estimates for age 15+ using an age standard calculated using the non-weighted mean of the GBD year's age-specific proportional distributions for national locations with populations greater than 5 million in the GBD year (1). Because it is unweighted, the proportion for a particular age in a large country, such as India, will not contribute more to this mean age standard than a smaller country.

Age Group	Age Weight
Early Neonatal	0.000397
Late Neonatal	0.001181
Post Neonatal	0.018683
1 to 4	0.079125
5 to 9	0.095772
10 to 14	0.089964
15 to 19	0.083257
20 to 24	0.078671
25 to 29	0.076342
30 to 34	0.073308
35 to 39	0.068075
40 to 44	0.061351
45 to 49	0.055067
50 to 54	0.049188
55 to 59	0.043432
60 to 64	0.036825
65 to 69	0.029901
70 to 74	0.022722
75 to 79	0.016071
80 to 84	0.01112
85 to 89	0.006161
90 to 94	0.002544
95 plus	0.000844

Supplementary Table S2. Age weights used for age standardization.

Exposure

Case Definitions

We defined current users of chewing tobacco as individuals who currently use any chewing tobacco product on a daily or occasional basis. This includes a variety of chewing tobacco products, including but not limited to gutkha, shammah, betel quid, toombak, zarda, plug, nass, and rape (2).

We defined current users of non-chewing tobacco as individuals who currently use any smokeless tobacco product that is not a chewing tobacco product on a daily or occasional basis. This includes a variety of smokeless, non-chewing tobacco products, including but not limited to snus, tooth powder, dry snuff, and mishri (2).

We defined current users of all smokeless tobacco products as individuals who currently use any smokeless tobacco product on a daily or occasional basis.

Current use is defined as use within the last 30 days.

Inclusion Criteria

We systematically reviewed surveys with information on tobacco use available in the Global Health Data Exchange (GHDx, http://ghdx.healthdata.org/). Surveys were extracted if they met our inclusion criteria, which were the following:

- Included indicators of tobacco use that met any of our standard case definitions
- Representative of the general population of one of the 204 countries and territories included in our analysis
    - Includes most surveys where the documentation explicitly states that it is representative of an entire location, and there is no other reason listed in the documentation that clearly conflicts with this statement
    - Excludes surveys conducted exclusively among sub-populations (including those with a specific disease, racial/ethnic minorities, pregnant women, etc.)
- One exception is that school-based surveys were included for individuals in primary or secondary school. Most surveys covering tobacco use among youth rely on school-based data collection, resulting in a paucity of population-representative data. As a result, we chose to include school-based surveys in order to have broad data coverage for youth ages 10-17.Data collection occurred between January 1, 1980 and December 31, 2019
- Tobacco use was self-reported by the individual, not by a proxy respondent
- Respondents were ages 10 and above
- The sample size for a particular location-age-sex-year-definition is greater than 10.

Data extraction

We extracted primary data from individual-level microdata, survey report tabulations, and scientific literature. When available, we prefer to extract individual-level microdata. If individual-level data were unavailable from a survey meeting our inclusion criteria, we extract available tabulated data from the

survey report. Tabulated data are population-level data aggregated to various degrees. Scientific literature data are tabulated data, but we define the former as articles containing data that were published in peer-reviewed journals, print or online. We extracted data on current smokeless tobacco use reported as any combination of frequency of use (daily, occasional, and unspecified, which includes both daily and occasional smokers) and type of smokeless tobacco used (chewing tobacco, non-chewing tobacco, or unspecified smokeless tobacco). Data points with either very high or very low prevalence for any of these three definitions were compared to other available data. If the sample size of the point was low and the prevalence was largely inconsistent with other available data, it was excluded from the model. In the preliminary models (before the final chewing tobacco prevalence model), the following percentage of location/age/sex/year-specific points were marked as outliers and were removed from the model:

- Chewing tobacco: 4.5%
- Non-chewing tobacco: 4.1%
- Unspecified smokeless tobacco: 1.5%

For microdata, we extracted relevant demographic information, including age, sex, location, and year, as well as survey metadata, including survey weights, primary sampling units (PSU), and strata. This information allowed us to tabulate individual-level data in the standard GBD five-year age-sex groups and produce accurate estimates of uncertainty. When survey design indicators are not reported in the microdata, we assume a conservative design effect of 2.25. For survey report tabulations, we extracted data at the most granular age-sex group provided.

We estimated the prevalence of current chewing tobacco using a collection of different models (see methods elsewhere in Appendix). In total, these models included 752 country-location-sources. Figure S5 depicts the number of data sources used for each country. Out of 204 countries and territories, 185 (90.69%) had at least one data source and 58 (28.43%) had at least five data sources. 57 (27.94%) of countries had their most recent data source from either 2017 or 2018.

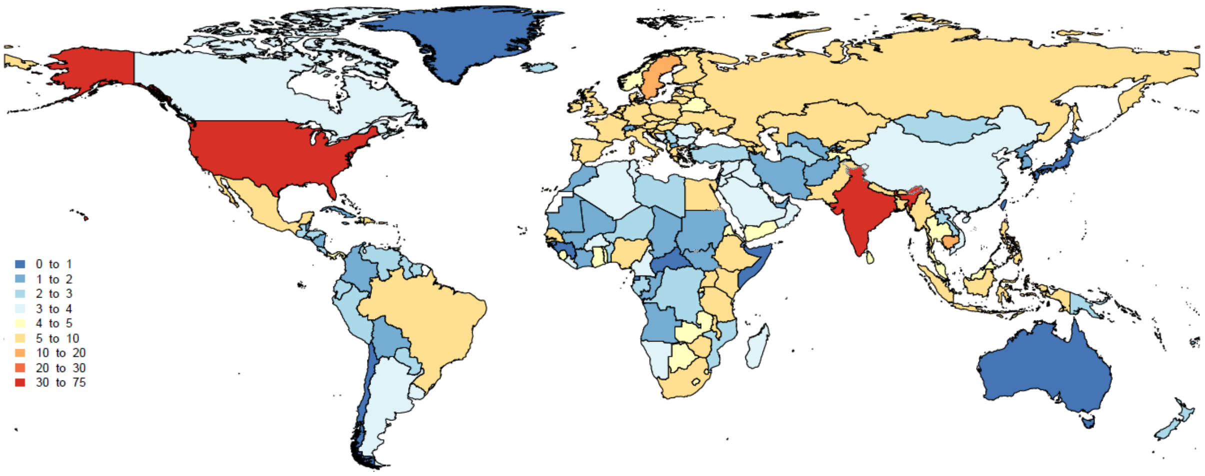

Figure S4. Number of sources per country for all component models.

The final chewing tobacco prevalence model included data from 738 country-location-sources. Figure S5 depicts the number of data sources used for each country. Out of 204 countries and territories, 185 (90.69%) had at least one data source and 57 (27.94%) had at least five data sources. 57 (27.94%) of countries had their most recent data source from either 2017 or 2018.

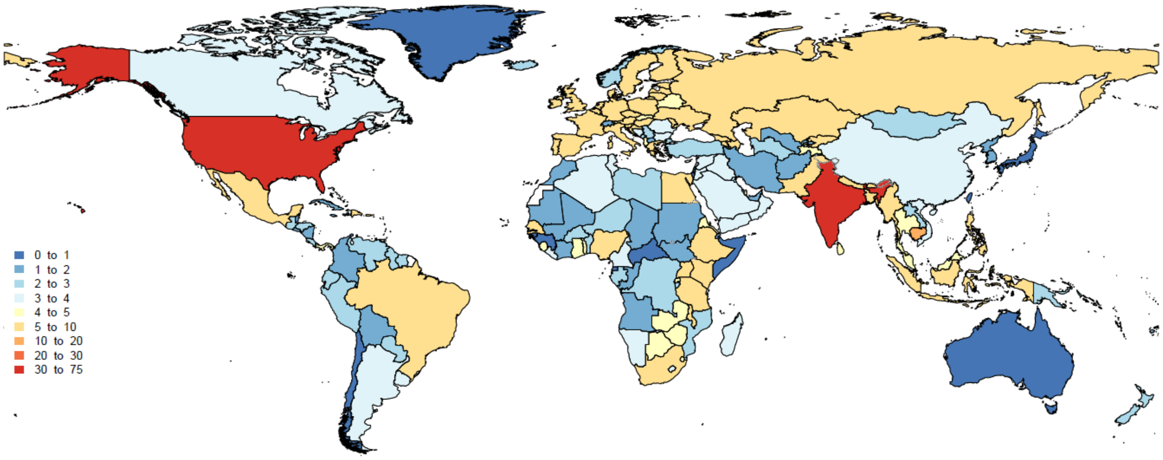

Figure S5. Number of sources per country for the final chewing tobacco prevalence model.

A complete list of data sources is available from the GBD 2019 Data Input Sources Tool: http://ghdx.healthdata.org/gbd-2019/data-input-sources.

Mapping definitions

There were 262 unique definitions of chewing, non-chewing, and smokeless tobacco products. The following table illustrates how broader categories of these definitions were mapped to each of these categories. However, although this table covers many of the definitions found, it does not cover all possible definitions.

Additionally, although some definitions were explicitly mapped to smokeless tobacco (not chewing tobacco or non-chewing tobacco), data that provided chewing tobacco and non-chewing tobacco prevalence were aggregated in order to calculate smokeless tobacco.

Definition	Mapped Category
Snus	Non-chewing tobacco
Dry snuff	Non-chewing tobacco
Tobacco toothpaste	Non-chewing tobacco
Nasal tobacco	Non-chewing tobacco
Nass (naswar, nasvai)	Chewing tobacco
Toombak	Chewing tobacco
Khaini	Chewing tobacco
Zarda	Chewing tobacco
Gutkha	Chewing tobacco
Rapé	Chewing tobacco
Shammah	Chewing tobacco
All smokeless tobacco	Smokeless tobacco
[list of both chewing and non-chewing tobacco]	Smokeless tobacco

Age and Sex Splitting

We split data reported in broader age groups than the GBD 5-year age groups or as both sexes combined by adapting the method reported in Ng et al. (3) to split using a sex- geography- time specific reference age pattern. We separated the data into two sets: a training dataset, with data already falling into GBD sex-specific 5-year age groups, and a split dataset, which reported data in aggregated age or sex groups. We then used spatiotemporal Gaussian Process Regression (ST-GPR) to estimate sex-geography-time specific age patterns using data in the training dataset. The estimated age patterns were used to split each source in the split dataset using the following equation:

$$\phi = \frac{N_{a,c,k}^{a+x}}{Pop_{a,s,c}}\left(\frac{R_{a,s}}{R_{a,s}^{a+x}}\right)$$

For example, for splitting the chewing tobacco prevalence model:

$N_{a,c,k}^{a+x}$: number of chewing tobacco users reported in survey k, for country c, sex s and an age group spanning age a to $a + x$;

$R_{a,s}$: total number of chewing tobacco users reported in the reference surveys for the target five-year age group a;

$R_{a,s}^{a+x}$: total number of chewing tobacco users also reported in the reference surveys but spanning the age group a to $a + x$;

$Pop_{a,s,c}$: the population of the target five-year age group, a, for country c, and sex s

The ST-GPR model used to estimate the age patterns for age-sex splitting used an age weight parameter value that minimises the effect of any age smoothing. This parameter choice allows the estimated age pattern to be driven by data, rather than being enforced by any smoothing parameters of the model. Because these age-sex split data points will be incorporated in the final ST-GPR exposure model, we do not want to doubly enforce a modelled age pattern for a given sex-location-year on a given aggregate data point.

For each of the smokeless tobacco definitions used, this model had reasonable coverage across geographies. The number of sources per super region for each of the models is as follows:

**Super Region**	**Unspecified Smokeless Tobacco**	**Chewing**	**Non-Chewing**
	**Number of Sources**		
North Africa and Middle East	27	4	4
Sub-Saharan Africa	101	56	58
Central Europe, Eastern Europe, and Central Asia	112	12	14

High-income	173	20	21
Latin America and Caribbean	60	11	9
South Asia	27	46	13
Southeast Asia, East Asia, and Oceania	67	16	11

Note that one source can cover multiple ages and sexes, and this is not measured in the above table.

Chewing Tobacco Prevalence Modelling

We used ST-GPR to model chewing tobacco prevalence. Full details on the ST-GPR method are reported elsewhere(4). Briefly, the mean function input to GPR is a complete time series of estimates generated from a mixed effects hierarchical linear model plus weighted residuals smoothed across time, space and age. The linear model formula for chewing tobacco, fit separately by sex using restricted maximum likelihood in R, is:

$$logit(p_{g,a,t}) = \beta_0 + \sum_{k=1}^{18} \beta_k I_{A[a]} + \alpha_s + \alpha_r + \alpha_g + \epsilon_{g,a,t}$$

Where $I_{A[a]}$ is a dummy variable indicating specific age group A that the prevalence point $p_{g,a,t}$ captures, and α_s, α_r, and α_g are super region, region, and geography random intercepts, respectively.

The model hyperparameters used are as follows:

Data Category	Lambda	Omega	Zeta	Scale
**<5 Sources**	0.04	0.87	0.01	20
**5-9 Sources**	0.04	1.48	0.01	20
**10-19 Sources**	0.04	1.48	0.005	20
**20+ Sources**	0.04	1.84	0.005	20

We ran three ST-GPR models for each smokeless tobacco category (chew, non-chew, and unspecified smokeless), and a final chewing tobacco prevalence model post-adjustment (described below). The same hyperparameters were used across all models.

All smokeless tobacco prevalence adjustment

Using the 1000 draws from each of the prevalence ST-GPR models, we calculated 1000 draws of chewing tobacco prevalence divided by the sum of chewing tobacco and non-chewing tobacco prevalence for each location, age group, sex, and year. For every draw in each age-sex-location-year (a, s, l, y):

$$ratio\_chew_{a,s,l,y} = \frac{chewing\ tobacco\ prevalence_{a,s,l,y}}{(non\ chewing\ tobacco\ prevalence_{a,s,l,y} + chewing\ tobacco\ prevalence_{a,s,ly,})}.$$

The draws were unordered, as we did not want to enforce an assumption about the relationship between the levels of chewing tobacco and non-chewing tobacco prevalence.

There exists a potential issue with the accuracy of these estimates by smoothing over age, time, and location. However, we are less likely to have large modeling issues in the high prevalence locations, which is the focus of this paper, because of the data availability in those locations. The following table shows the percentage of age-sex-location-year-specific data points that had to be adjusted (i.e. unspecified smokeless tobacco prevalence adjusted by this ratio) by location/sex, ordered from smallest to largest percentage:

Location	Sex	Percentage Adjusted	Product Specificity Available
Bangladesh	Males	3	Location

Bangladesh	Females	4	Location
Yemen	Males	6	Location
Yemen	Females	6	Location
Nepal	Females	12	Location
Cambodia	Females	23	Location
India	Females	26	Location
India	Males	27	Location
Cambodia	Males	31	Location
Nepal	Males	38	Location
Madagascar	Males	55	Location
Madagascar	Females	56	Location
Myanmar	Males	67	Location
Myanmar	Females	67	Location
Bhutan	Males	68	Location
Bhutan	Females	68	Location
Pakistan	Males	70	Location
Pakistan	Females	70	Location
Marshall Islands	Males	83	Location
Marshall Islands	Females	83	Location
Sri Lanka	Males	100	Region
Sri Lanka	Females	100	Region
Palau	Males	100	Region
Palau	Females	100	Region

The column "Product Specificity" indicates at which geographic level there are product-specific data available across all years, ages, and sexes. This table indicates that many locations do not need to rely on the data adjustment for most of their data points. For the locations that do use this adjustment for more than half of the data points, all but Sri Lanka and Palau have product-specific data for that location to help inform the adjustment ratio.

The draws of the ratio were multiplied by the draws from the all smokeless tobacco prevalence model to adjust the smokeless tobacco estimates to reflect chewing tobacco prevalence:

$$SLT\ adjusted\ to\ chewing\ tobacco\ data_{a,s,l,y} = SLT\ data_{a,s,l,y} \times ratio\_chew_{a,s,l,y}$$

These adjusted draws were then averaged to get the mean estimate. The variance across the ratios was calculated for each location, year, age, and sex, and was added to the variance from the original all smokeless tobacco draws.

Final chewing tobacco prevalence model

To calculate the final chewing tobacco prevalence, an ST-GPR model was run with both the original chewing tobacco data (post-age sex splitting) and the adjusted all smokeless tobacco data. These

adjusted data add more information to the model – as surveys will often only ask about all smokeless tobacco consumption – while taking into consideration the uncertainty from the ratio calculation.

Although this model allows for information sharing across ages, years, and locations, surveys that collect the same information but using very different methods could result in time or age trends that are more a reflection of these differences than true changes over time or age. However, the time trends were generally quite stable (Supplementary Table 1), and thus it is less likely that sources are causing systematic issues over time. Additionally, because the highest prevalence locations all had at least four sources (and eleven out of twelve highest prevalence locations have eight or more data sources), this limitation is less likely to significantly influence our findings, since this paper focused primarily on these locations. Finally, 94% of our data points come from major survey series – GYTS, GATS, DHS, STEPS, Eurobarometer, MICS, and United States-specific survey series administered over multiple years, such as BRFSS and the United States National Youth Tobacco survey. Given that these surveys tend to ask questions consistently both over time and between countries, variation due to the survey instrument used is likely to not be a significant source of uncertainty and variation in our analysis.

Supplementary Results

Spatial, temporal, and demographic patterns in chewing tobacco prevalence 1990-2019: a systematic analysis of 204 countries and territories using new methods from the Global Burden of Disease Study 2019

GBD 2019 Chewing Tobacco Collaborators

Supplementary Figures and Tables

Supplementary Figure 1. Overview of current chewing tobacco prevalence estimation. ST-GPR=spatiotemporal Gaussian process regression.

Supplementary Figure 2. Global prevalence of chewing tobacco for people aged 15 plus, by age and sex, in 2019.

Supplementary Figure 3. Ratio of age-standardized chewing tobacco prevalence to smoking prevalence in 1990 and 2019 for males aged 15 plus.

Supplementary Figure 4. Ratio of age-standardized chewing tobacco prevalence to smoking prevalence in 1990 and 2019 for females aged 15 plus.

Supplementary Figure 5. Sensitivity analysis of chewing tobacco prevalence modeled using only the data available for chewing tobacco explicitly (x-axis) versus both the original chewing tobacco data plus the adjusted unspecified smokeless tobacco data (y-axis). Colored by super region.

Supplementary Figure 6. By sex, ages 15–19, current chewing tobacco prevalence in 2019.

Supplementary Table 1. Age-standardized prevalence of current chewing tobacco use in 1990 and 2019 for males and females aged 15 plus, as well as annualized rate of change. Locations organized by GBD region.

Supplementary Table 2. Age-standardized prevalence of current smoked tobacco use in 1990 and 2019 for males and females aged 15 plus, as well as annualized rate of change. Locations organized by GBD region.

Supplementary Table 3. Prevalence and number of users of current chewing tobacco and smoking use among people ages 15-19 in 1990 and 2019. Locations ordered by GBD super region and region.

Supplementary Table 4. Number (in thousands) of current chewing tobacco use in 1990 and 2019 for males and females aged 15 plus. Locations organized alphabetically.

Supplementary Table 5. Age-standardized chewing tobacco prevalence in 2019 by region, for males and females separately.

Supplementary Figure 1.

Overview of current chewing tobacco prevalence estimation. ST-GPR=spatiotemporal Gaussian process regression.

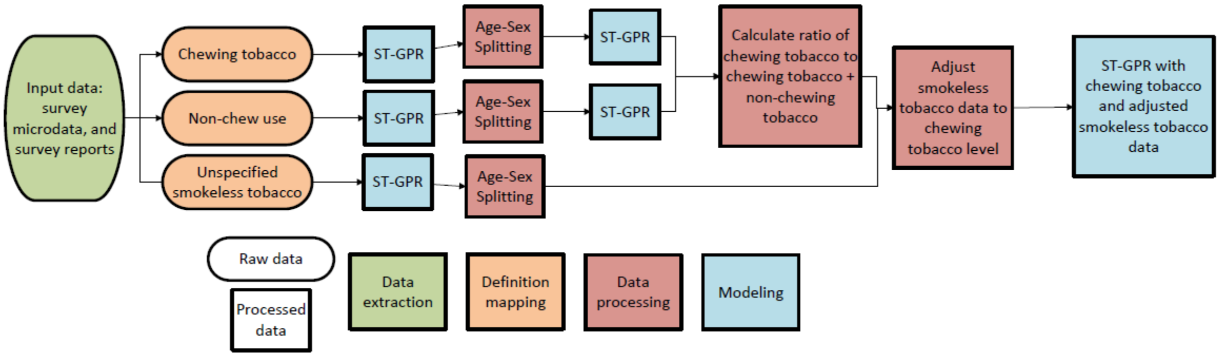

Supplementary Figure 2.

Global prevalence of chewing tobacco for people aged 15 plus, by age and sex, in 2019.

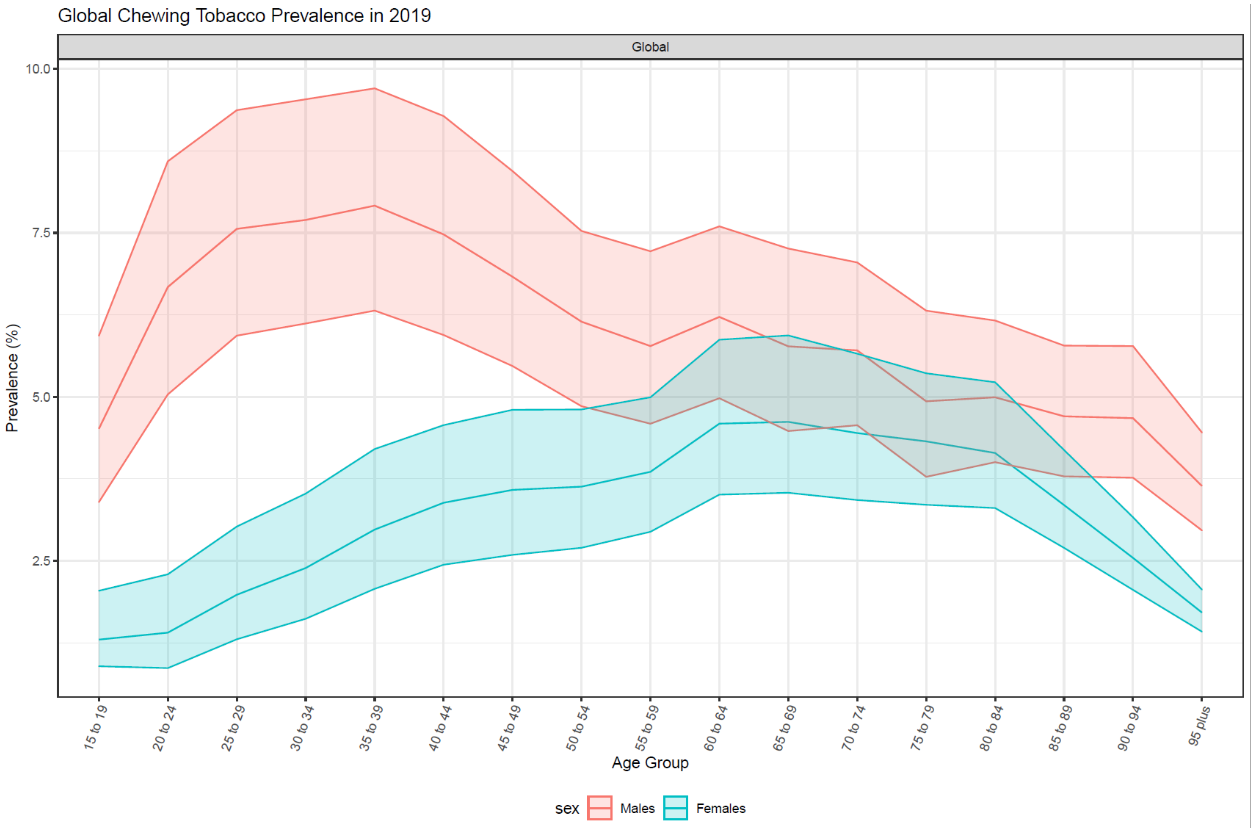

Supplementary Figure 3.

Ratio of age-standardized chewing tobacco prevalence to smoking prevalence in 1990 and 2019 for males aged 15 plus.

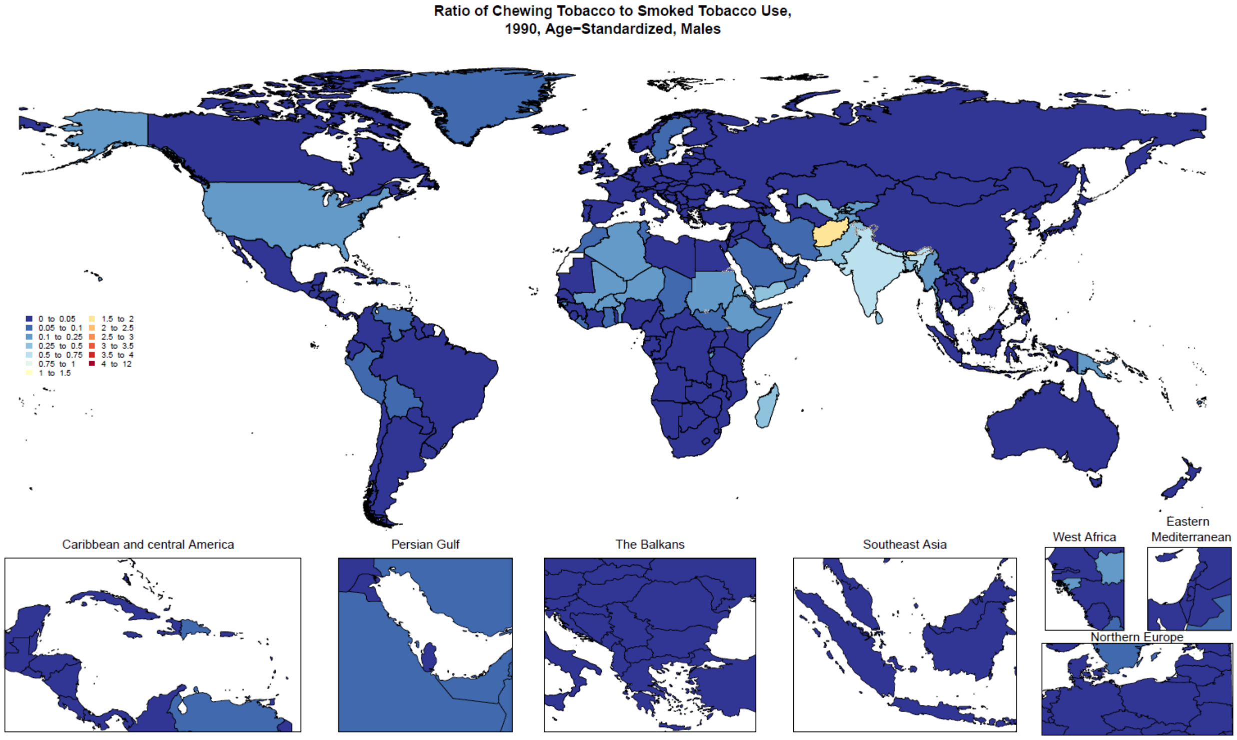

Ratio of Chewing Tobacco to Smoked Tobacco Use, 2019, Age−Standardized, Males

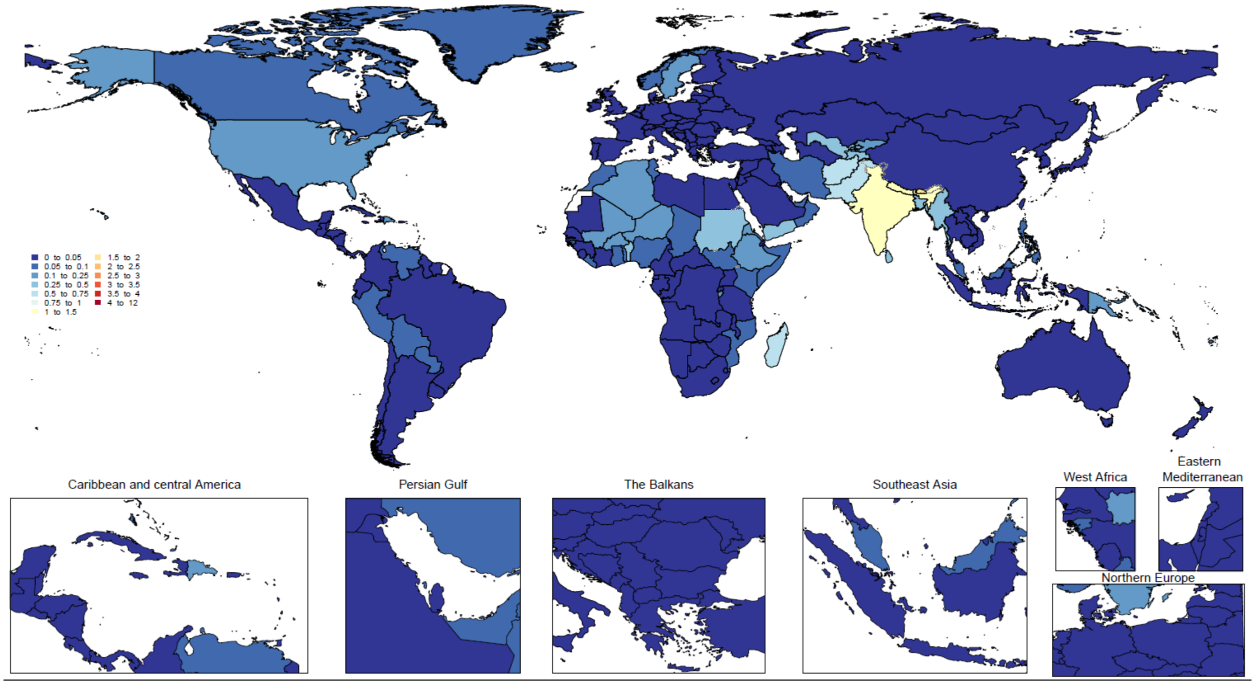

Supplementary Figure 4.

Ratio of age-standardized chewing tobacco prevalence to smoking prevalence in 1990 and 2019 for females aged 15 plus.

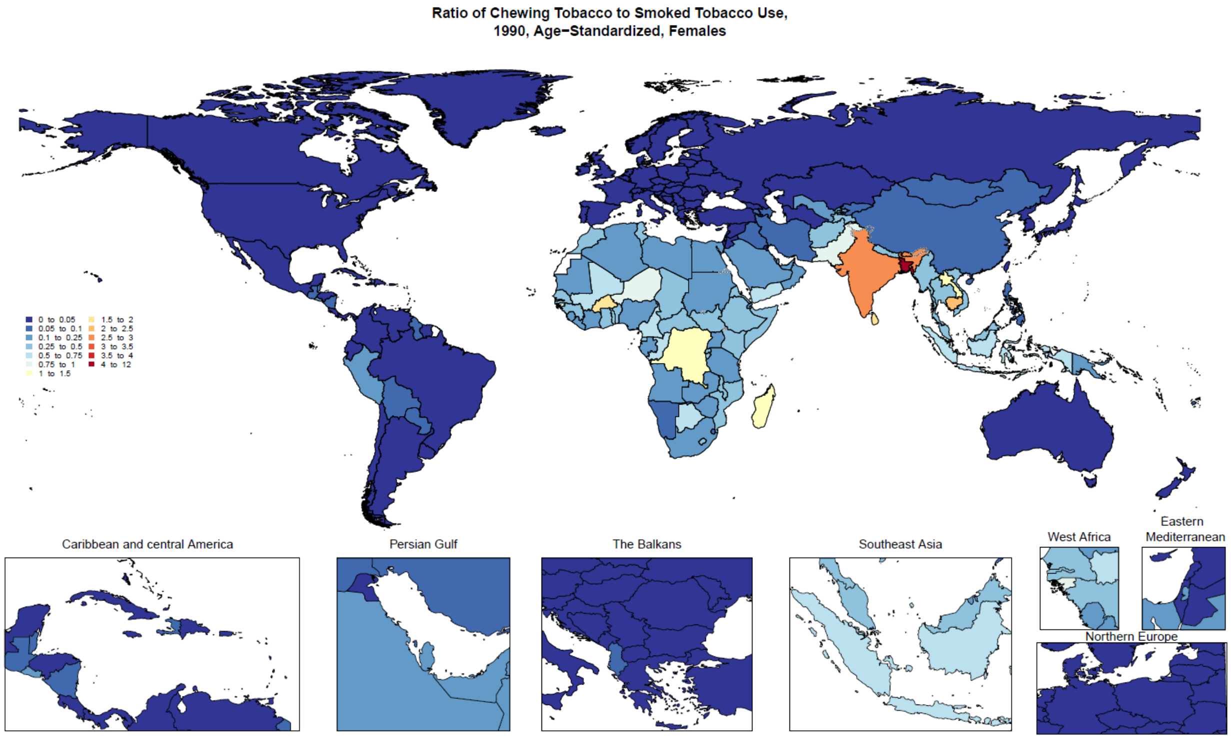

Ratio of Chewing Tobacco to Smoked Tobacco Use, 2019, Age−Standardized, Females

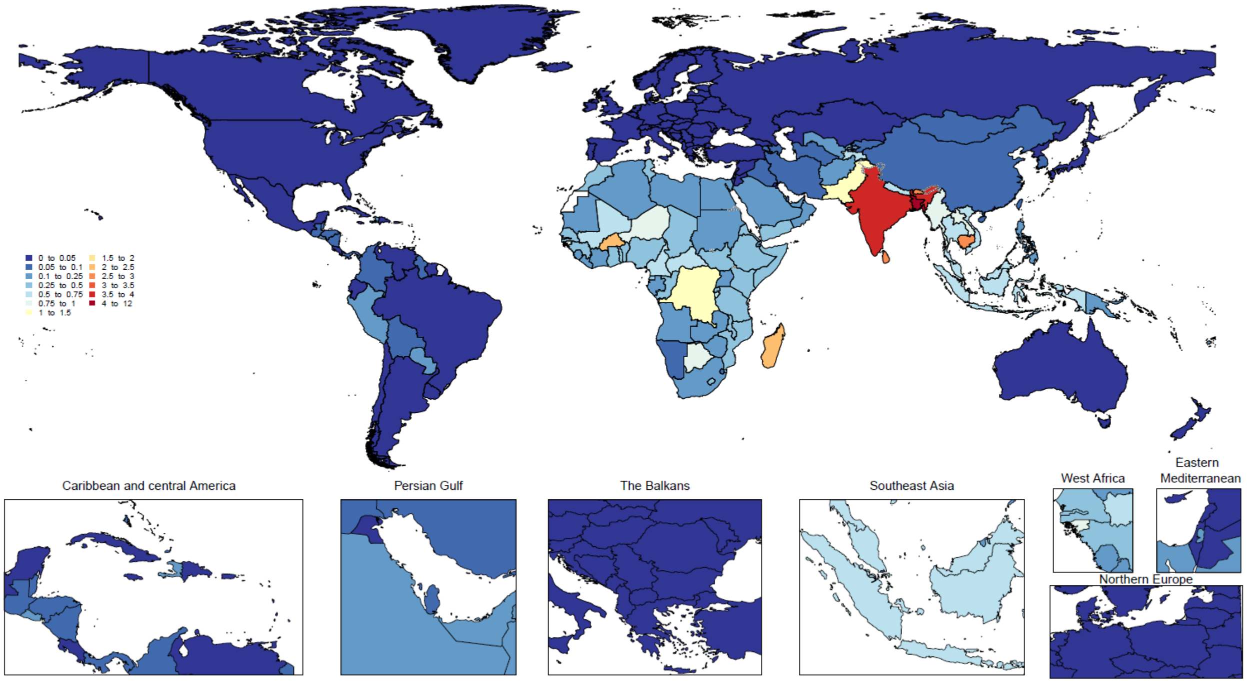

Supplementary Figure 5.

Sensitivity analysis of chewing tobacco prevalence modeled using only the data available for chewing tobacco explicitly (x-axis) versus both the original chewing tobacco data plus the adjusted unspecified smokeless tobacco data (y-axis). Colored by super region.

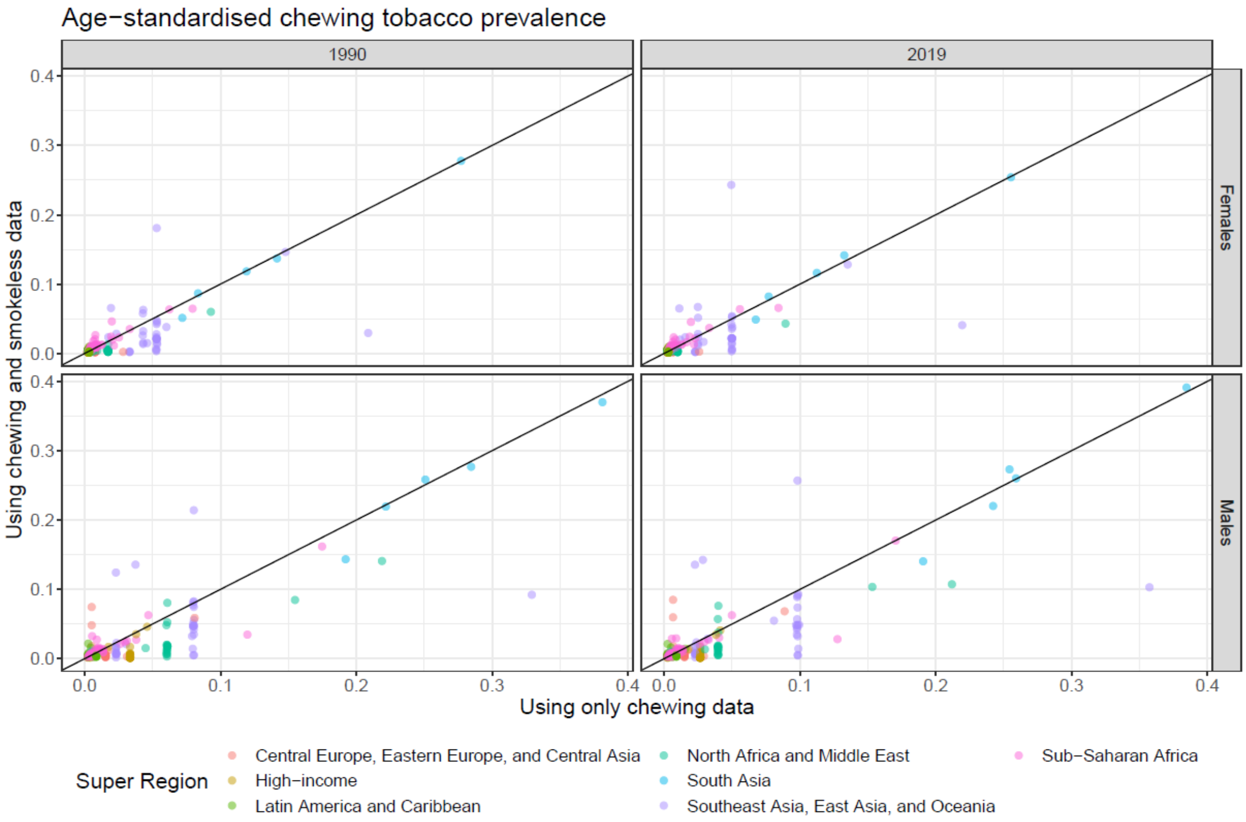

The following results are across all years 1990-2019. Overall, the correlation of the two estimates is 0.821. On average, when using data on chewing and smokeless tobacco, the estimates of the age-standardised prevalence of chewing tobacco are 0.83 percentage points lower globally than when just using the chewing tobacco data. The largest percentage point increases in all-age chewing tobacco prevalence when using both the chewing tobacco and adjusted, unspecified smokeless tobacco data are seen in Palau, Myanmar, and Sri Lanka (14.9, 8.3, and 6.4 percentage points, respectively). The largest decreases are seen in the Marshall Islands, Nauru, and Niue (21.2, 6.5, and 6.5 percentage points, respectively). The estimates for males decreased slightly more than the female estimates (1 percentage points versus 0.64 percentage points) when using both the chewing tobacco and adjusted, unspecified smokeless tobacco data.

Among countries with age-standardised prevalence of chewing tobacco greater than 5%, using both data types results in a 0.046 percentage point decrease in the total age-standardised chewing tobacco prevalence. The Marshall Islands, Yemen, and Kiribati had the largest decreases when using both data types (21.2, 6.1. and 3.6 percentage points, respectively). The largest increases are the same as those listed above. Additionally, among just these locations, the male estimates decreased slightly (-0.22 percentage points) while the female estimates increased slightly (0.14 percentage points).

Using data on both chewing and smokeless tobacco allows us to incorporate information from an additional 410 data sources (there are 170 data sources on chewing model alone) and we believe that the information added by the inclusion of the smokeless tobacco data sources improves the estimation.

Supplementary Figure 6.

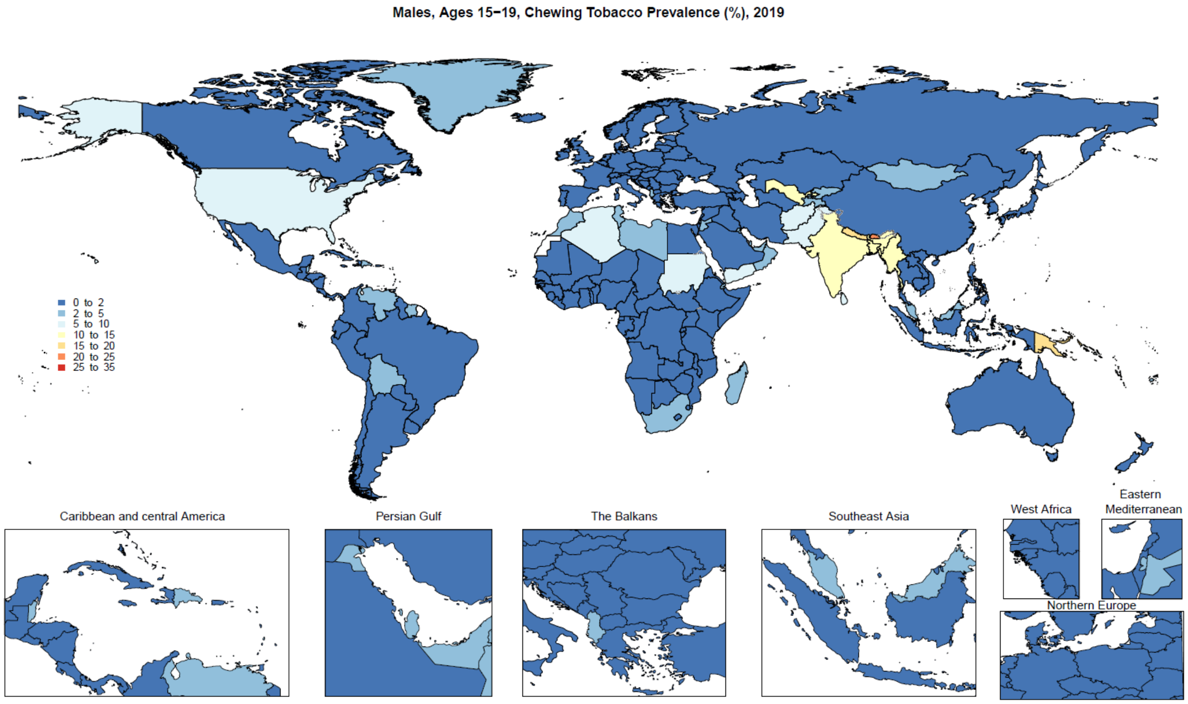

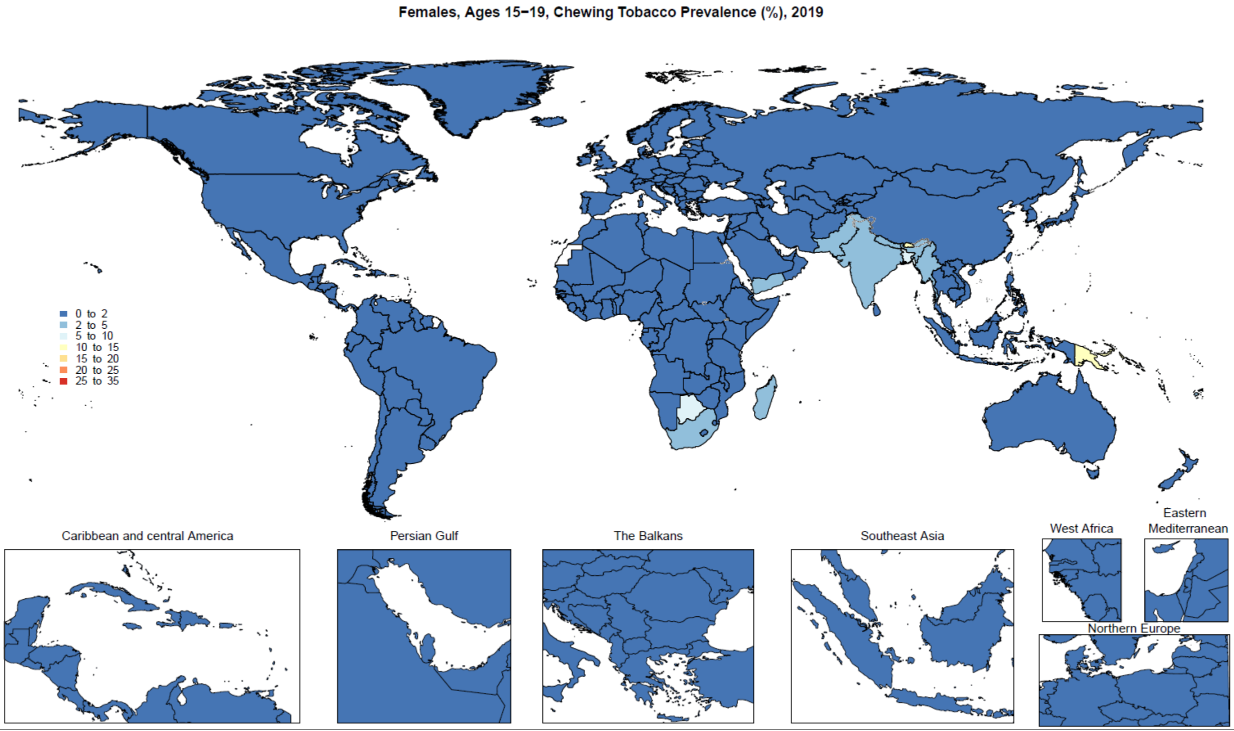

Supplementary Table 1. Age-standardized prevalence of current chewing tobacco use in 1990 and 2019 for males and females aged 15 plus, as well as annualized rate of change. Locations organized by GBD region.

	Males					Females				
	Prevalence		Annualized Rate of Change (ARC)			Prevalence		Annualized Rate of Change (ARC)		
**Location**	**1990**	**2019**	**1990-2005**	**2005-2019**	**1990-2019**	**1990**	**2019**	**1990-2005**	**2005-2019**	**1990-2019**
Global	5.84% (5.31 to 6.40)	6.55% (6.10 to 7.03)	0.65% (-0.04 to 1.31)	0.12% (-0.48 to 0.76)	0.39% (-0.01 to 0.83)	2.41% (2.14 to 2.74)	2.87% (2.60 to 3.14)	0.73% (-0.23 to 1.60)	0.46% (-0.36 to 1.27)	0.60% (0.04 to 1.11)
China	0.73% (0.59 to 0.92)	0.86% (0.68 to 1.07)	0.01% (-1.58 to 1.53)	1.09% (-0.59 to 2.79)	0.53% (-0.46 to 1.61)	0.23% (0.17 to 0.30)	0.23% (0.17 to 0.32)	0.00% (-2.12 to 2.01)	0.15% (-2.04 to 2.36)	0.07% (-1.25 to 1.45)
Democratic People's Republic of Korea	0.19% (0.16 to 0.23)	0.19% (0.15 to 0.23)	-0.01% (-1.51 to 1.48)	-0.08% (-1.62 to 1.48)	-0.05% (-1.09 to 0.92)	0.19% (0.14 to 0.25)	0.19% (0.15 to 0.26)	0.06% (-1.91 to 2.15)	0.05% (-2.09 to 2.07)	0.05% (-1.17 to 1.43)
Taiwan (Province of China)	0.62% (0.49 to 0.76)	0.60% (0.47 to 0.75)	-0.02% (-1.66 to 1.58)	-0.16% (-1.81 to 1.43)	-0.09% (-1.16 to 0.93)	0.30% (0.23 to 0.41)	0.28% (0.21 to 0.37)	-0.12% (-2.13 to 1.92)	-0.45% (-2.58 to 1.70)	-0.28% (-1.61 to 1.10)
Cambodia	1.62% (1.34 to 1.96)	1.70% (1.39 to 2.07)	0.69% (-0.62 to 2.04)	-0.42% (-1.93 to 1.08)	0.15% (-0.73 to 1.10)	14.57% (12.45 to 16.95)	12.84% (11.05 to 14.70)	0.45% (-0.58 to 1.48)	-1.38% (-2.49 to -0.33)	-0.43% (-1.22 to 0.30)
Indonesia	0.40% (0.32 to 0.48)	0.43% (0.36 to 0.52)	0.05% (-1.31 to 1.47)	0.59% (-0.72 to 2.12)	0.31% (-0.57 to 1.25)	2.37% (1.81 to 3.05)	2.02% (1.51 to 2.62)	-0.72% (-2.80 to 1.27)	-0.40% (-2.38 to 1.52)	-0.56% (-1.83 to 0.75)
Laos	0.76% (0.61 to 0.93)	0.73% (0.58 to 0.90)	0.02% (-1.54 to 1.58)	-0.30% (-1.73 to 1.21)	-0.13% (-1.12 to 0.88)	6.30% (4.91 to 7.99)	6.73% (5.31 to 8.24)	0.19% (-1.39 to 1.78)	0.28% (-1.39 to 1.97)	0.23% (-0.82 to 1.24)
Malaysia	2.12% (1.71 to 2.58)	2.88% (2.32 to 3.51)	0.51% (-0.99 to 1.86)	1.64% (0.27 to 3.10)	1.06% (0.08 to 1.96)	1.52% (1.17 to 2.01)	1.66% (1.27 to 2.13)	0.07% (-1.94 to 1.81)	0.60% (-1.13 to 2.40)	0.32% (-1.00 to 1.54)
Maldives	5.09% (4.12 to 6.10)	5.47% (4.61 to 6.59)	0.94% (-0.36 to 2.35)	-0.48% (-1.87 to 0.92)	0.25% (-0.66 to 1.16)	3.84% (2.97 to 4.88)	3.78% (2.96 to 4.71)	0.60% (-1.02 to 2.19)	-0.76% (-2.54 to 1.03)	-0.06% (-1.29 to 1.08)
Myanmar	13.51% (11.19 to 16.07)	14.18% (11.94 to 16.53)	1.24% (0.03 to 2.43)	-0.97% (-2.30 to 0.33)	0.17% (-0.62 to 1.06)	6.63% (5.23 to 8.43)	6.53% (5.14 to 8.20)	0.60% (-0.99 to 2.13)	-0.75% (-2.35 to 1.08)	-0.05% (-1.15 to 1.02)
Philippines	2.28% (1.89 to 2.75)	2.34% (1.93 to 2.81)	0.16% (-1.19 to 1.45)	0.01% (-1.41 to 1.37)	0.09% (-0.78 to 0.95)	1.09% (0.85 to 1.40)	1.04% (0.79 to 1.33)	-0.05% (-1.79 to 1.72)	-0.31% (-2.17 to 1.51)	-0.18% (-1.33 to 0.98)
Sri Lanka	12.43% (10.39 to 14.73)	13.57% (11.39 to 15.77)	0.65% (-0.45 to 1.82)	-0.06% (-1.27 to 1.15)	0.30% (-0.45 to 1.10)	5.78% (4.53 to 7.25)	5.15% (4.05 to 6.39)	-0.05% (-1.62 to 1.52)	-0.78% (-2.51 to 0.85)	-0.40% (-1.51 to 0.66)
Thailand	1.17% (0.97 to 1.42)	1.20% (1.00 to 1.45)	0.06% (-1.19 to 1.37)	0.10% (-1.25 to 1.46)	0.08% (-0.75 to 0.94)	2.60% (1.96 to 3.27)	2.34% (1.82 to 2.99)	0.28% (-1.38 to 2.08)	-1.05% (-2.98 to 0.88)	-0.36% (-1.56 to 0.94)
Timor-Leste	1.21% (1.00 to 1.48)	1.29% (1.05 to 1.56)	0.25% (-1.13 to 1.63)	0.16% (-1.26 to 1.61)	0.21% (-0.73 to 1.16)	2.84% (2.21 to 3.64)	2.36% (1.84 to 3.08)	0.19% (-1.64 to 1.87)	-1.54% (-3.27 to 0.23)	-0.64% (-1.77 to 0.52)
Viet Nam	0.61% (0.50 to 0.75)	0.64% (0.51 to 0.78)	0.00% (-1.41 to 1.42)	0.28% (-1.12 to 1.72)	0.14% (-0.85 to 1.08)	1.53% (1.14 to 2.02)	1.43% (1.09 to 1.86)	-0.04% (-1.91 to 1.85)	-0.45% (-2.33 to 1.56)	-0.23% (-1.46 to 1.11)
Fiji	3.55% (2.76 to 4.51)	3.51% (2.82 to 4.41)	-0.04% (-1.66 to 1.66)	-0.03% (-1.82 to 1.57)	-0.04% (-1.21 to 1.07)	1.53% (1.13 to 2.09)	1.56% (1.15 to 2.12)	0.02% (-2.00 to 2.14)	0.09% (-2.17 to 2.36)	0.05% (-1.38 to 1.49)
Kiribati	5.59% (4.35 to 7.08)	5.72% (4.58 to 7.04)	0.07% (-1.65 to 1.72)	0.10% (-1.67 to 1.88)	0.08% (-0.98 to 1.14)	1.32% (0.93 to 1.88)	1.31% (0.92 to 1.88)	-0.03% (-2.47 to 2.59)	-0.02% (-2.48 to 2.58)	-0.02% (-1.70 to 1.60)
Marshall Islands	9.14% (7.14 to 11.51)	10.36% (8.25 to 12.63)	0.52% (-1.14 to 2.25)	0.35% (-1.19 to 1.94)	0.44% (-0.66 to 1.55)	2.97% (2.06 to 4.28)	4.06% (2.98 to 5.40)	0.69% (-1.97 to 3.20)	1.55% (-1.23 to 4.17)	1.10% (-0.56 to 2.76)
Micronesia (Federated States of)	8.41% (6.35 to 10.62)	9.10% (7.35 to 11.27)	0.37% (-1.25 to 2.07)	0.18% (-1.60 to 1.93)	0.28% (-0.81 to 1.36)	4.75% (3.24 to 6.84)	5.39% (4.00 to 7.30)	0.65% (-1.58 to 3.12)	0.27% (-2.63 to 3.26)	0.47% (-1.12 to 2.16)

Papua New Guinea	7.51% (5.83 to 9.56)	7.39% (5.83 to 9.20)	0.23% (-1.38 to 1.86)	-0.36% (-2.00 to 1.33)	-0.05% (-1.14 to 1.01)	4.25% (2.87 to 6.12)	4.19% (3.07 to 5.61)	0.29% (-2.22 to 2.85)	-0.36% (-2.74 to 2.04)	-0.02% (-1.57 to 1.57)
Samoa	1.45% (1.10 to 1.85)	1.44% (1.11 to 1.84)	-0.05% (-1.88 to 1.85)	0.03% (-1.78 to 1.93)	-0.01% (-1.19 to 1.20)	0.65% (0.46 to 0.91)	0.65% (0.45 to 0.93)	-0.01% (-2.41 to 2.38)	0.07% (-2.52 to 2.67)	0.03% (-1.61 to 1.64)
Solomon Islands	3.28% (2.61 to 4.13)	3.25% (2.53 to 4.12)	-0.06% (-1.87 to 1.69)	0.00% (-1.74 to 1.86)	-0.03% (-1.21 to 1.02)	2.15% (1.49 to 3.03)	2.21% (1.60 to 3.03)	0.10% (-2.44 to 2.40)	0.09% (-2.50 to 2.47)	0.10% (-1.51 to 1.64)
Tonga	4.71% (3.63 to 6.00)	4.88% (3.78 to 6.26)	-0.04% (-1.75 to 1.75)	0.29% (-1.47 to 2.06)	0.12% (-1.05 to 1.31)	2.09% (1.50 to 2.93)	2.16% (1.53 to 2.99)	0.03% (-2.38 to 2.39)	0.21% (-2.25 to 2.55)	0.11% (-1.38 to 1.73)
Vanuatu	4.18% (3.27 to 5.24)	4.24% (3.30 to 5.26)	0.01% (-1.58 to 1.72)	0.08% (-1.62 to 1.87)	0.05% (-1.00 to 1.19)	2.11% (1.50 to 2.98)	2.19% (1.56 to 2.98)	0.02% (-2.32 to 2.25)	0.23% (-2.27 to 2.67)	0.12% (-1.31 to 1.68)
Armenia	0.24% (0.19 to 0.30)	0.24% (0.19 to 0.30)	-0.05% (-1.69 to 1.51)	0.03% (-1.67 to 1.69)	-0.01% (-1.09 to 1.06)	0.21% (0.15 to 0.28)	0.20% (0.15 to 0.27)	0.05% (-2.04 to 2.25)	-0.23% (-2.35 to 1.92)	-0.09% (-1.50 to 1.31)
Azerbaijan	0.28% (0.22 to 0.35)	0.29% (0.22 to 0.36)	0.00% (-1.61 to 1.49)	0.24% (-1.50 to 1.94)	0.12% (-1.06 to 1.23)	0.19% (0.14 to 0.25)	0.19% (0.14 to 0.26)	0.05% (-2.10 to 2.32)	0.13% (-2.00 to 2.33)	0.09% (-1.41 to 1.53)
Georgia	0.42% (0.33 to 0.54)	0.44% (0.35 to 0.55)	-0.08% (-1.93 to 1.62)	0.36% (-1.34 to 2.03)	0.14% (-0.89 to 1.25)	0.22% (0.16 to 0.29)	0.22% (0.16 to 0.30)	-0.10% (-2.17 to 2.13)	0.11% (-2.11 to 2.30)	0.00% (-1.42 to 1.52)
Kazakhstan	0.83% (0.67 to 1.03)	0.90% (0.73 to 1.09)	-0.03% (-1.52 to 1.41)	0.58% (-0.91 to 2.12)	0.27% (-0.70 to 1.20)	0.20% (0.14 to 0.27)	0.19% (0.14 to 0.25)	-0.04% (-2.08 to 2.03)	-0.36% (-2.46 to 1.76)	-0.20% (-1.58 to 1.17)
Kyrgyzstan	5.91% (4.82 to 7.13)	6.81% (5.67 to 8.02)	0.92% (-0.30 to 2.29)	0.03% (-1.35 to 1.50)	0.49% (-0.40 to 1.44)	0.25% (0.18 to 0.33)	0.24% (0.18 to 0.32)	0.07% (-2.14 to 2.02)	-0.18% (-2.18 to 1.88)	-0.05% (-1.40 to 1.30)
Mongolia	1.84% (1.37 to 2.46)	1.83% (1.36 to 2.43)	-0.02% (-1.87 to 1.90)	0.00% (-2.07 to 2.04)	-0.01% (-1.26 to 1.31)	0.56% (0.38 to 0.84)	0.56% (0.37 to 0.83)	0.08% (-2.72 to 3.00)	-0.11% (-3.16 to 2.91)	-0.01% (-1.91 to 1.91)
Tajikistan	4.84% (3.93 to 5.94)	5.98% (4.80 to 7.23)	0.13% (-1.26 to 1.63)	1.37% (-0.09 to 2.91)	0.73% (-0.16 to 1.74)	0.58% (0.44 to 0.76)	0.65% (0.49 to 0.83)	-0.01% (-1.88 to 2.00)	0.83% (-1.10 to 2.72)	0.40% (-0.78 to 1.64)
Turkmenistan	0.40% (0.32 to 0.50)	0.37% (0.29 to 0.46)	-0.08% (-1.65 to 1.50)	-0.51% (-2.04 to 0.93)	-0.29% (-1.32 to 0.70)	0.17% (0.13 to 0.23)	0.17% (0.13 to 0.23)	-0.07% (-1.99 to 1.98)	-0.10% (-2.08 to 1.93)	-0.08% (-1.35 to 1.23)
Uzbekistan	7.47% (5.85 to 9.17)	8.57% (7.06 to 10.46)	0.14% (-1.35 to 1.65)	0.84% (-0.69 to 2.34)	0.48% (-0.41 to 1.45)	0.21% (0.15 to 0.28)	0.21% (0.15 to 0.28)	0.01% (-2.16 to 2.23)	-0.07% (-2.39 to 2.11)	-0.03% (-1.45 to 1.45)
Albania	1.15% (0.88 to 1.49)	1.20% (0.93 to 1.51)	0.06% (-1.71 to 1.82)	0.24% (-1.55 to 2.01)	0.15% (-1.01 to 1.26)	0.38% (0.26 to 0.54)	0.40% (0.28 to 0.57)	-0.09% (-2.39 to 2.49)	0.42% (-2.06 to 2.94)	0.16% (-1.41 to 1.87)
Bosnia and Herzegovina	0.56% (0.41 to 0.74)	0.65% (0.50 to 0.85)	-0.05% (-1.99 to 1.88)	1.16% (-0.82 to 3.23)	0.54% (-0.73 to 1.79)	0.32% (0.23 to 0.44)	0.34% (0.25 to 0.47)	-0.09% (-2.41 to 2.03)	0.67% (-1.82 to 3.07)	0.27% (-1.30 to 1.79)
Bulgaria	0.63% (0.50 to 0.80)	0.91% (0.72 to 1.13)	0.02% (-1.56 to 1.56)	2.57% (0.96 to 4.12)	1.25% (0.22 to 2.23)	0.34% (0.26 to 0.45)	0.46% (0.35 to 0.59)	0.04% (-2.07 to 2.08)	2.09% (0.12 to 3.86)	1.03% (-0.30 to 2.33)
Croatia	0.32% (0.24 to 0.44)	0.36% (0.27 to 0.48)	0.00% (-1.93 to 2.05)	0.84% (-1.25 to 2.84)	0.40% (-0.87 to 1.64)	0.25% (0.17 to 0.36)	0.26% (0.18 to 0.40)	-0.06% (-2.53 to 2.46)	0.49% (-2.13 to 3.43)	0.21% (-1.58 to 1.96)
Czechia	0.31% (0.25 to 0.38)	0.49% (0.39 to 0.61)	0.04% (-1.48 to 1.59)	3.16% (1.68 to 4.73)	1.54% (0.53 to 2.58)	0.23% (0.17 to 0.30)	0.30% (0.23 to 0.40)	-0.01% (-1.91 to 1.91)	1.99% (-0.03 to 4.12)	0.95% (-0.28 to 2.17)
Hungary	0.22% (0.17 to 0.27)	0.24% (0.19 to 0.30)	0.02% (-1.51 to 1.63)	0.49% (-1.21 to 2.22)	0.25% (-0.80 to 1.37)	0.18% (0.13 to 0.24)	0.19% (0.14 to 0.25)	0.08% (-1.84 to 2.09)	0.34% (-1.56 to 2.34)	0.20% (-1.02 to 1.50)
North Macedonia	0.44% (0.33 to 0.57)	0.57% (0.44 to 0.75)	0.01% (-1.83 to 1.98)	1.86% (0.01 to 3.74)	0.91% (-0.35 to 2.21)	0.27% (0.19 to 0.37)	0.31% (0.22 to 0.43)	-0.02% (-2.25 to 2.27)	1.15% (-1.17 to 3.52)	0.54% (-0.91 to 2.02)
Montenegro	0.56% (0.42 to 0.75)	0.72% (0.54 to 0.95)	0.00% (-1.88 to 1.98)	1.79% (-0.30 to 3.87)	0.86% (-0.38 to 2.26)	0.31% (0.22 to 0.43)	0.35% (0.25 to 0.50)	-0.06% (-2.51 to 2.40)	1.04% (-1.41 to 3.30)	0.47% (-1.15 to 2.02)
Poland	0.32% (0.25 to 0.41)	0.39% (0.31 to 0.48)	0.01% (-1.61 to 1.65)	1.30% (-0.26 to 3.02)	0.63% (-0.47 to 1.72)	0.23% (0.17 to 0.31)	0.26% (0.19 to 0.35)	-0.01% (-2.30 to 2.18)	0.94% (-1.27 to 3.09)	0.45% (-1.03 to 1.83)
Romania	0.37% (0.29 to 0.46)	0.49% (0.39 to 0.61)	-0.03% (-1.61 to 1.41)	1.98% (0.37 to 3.60)	0.94% (-0.12 to 1.97)	0.21% (0.16 to 0.27)	0.23% (0.17 to 0.30)	-0.02% (-1.90 to 1.97)	0.60% (-1.37 to 2.64)	0.28% (-0.98 to 1.55)
Serbia	0.47% (0.36 to 0.61)	0.58% (0.45 to 0.75)	-0.02% (-1.91 to 1.79)	1.52% (-0.42 to 3.52)	0.72% (-0.48 to 1.96)	0.28% (0.21 to 0.40)	0.33% (0.24 to 0.46)	0.00% (-2.25 to 2.29)	1.10% (-1.24 to 3.34)	0.53% (-1.01 to 2.03)
Slovakia	0.22% (0.18 to 0.28)	0.25% (0.20 to 0.32)	0.02% (-1.59 to 1.56)	1.04% (-0.54 to 2.74)	0.51% (-0.51 to 1.51)	0.18% (0.14 to 0.23)	0.19% (0.14 to 0.25)	-0.01% (-2.02 to 1.91)	0.59% (-1.46 to 2.77)	0.28% (-1.00 to 1.50)
Slovenia	0.31% (0.24 to 0.39)	0.41% (0.32 to 0.52)	0.02% (-1.61 to 1.74)	1.95% (0.44 to 3.70)	0.95% (-0.07 to 2.04)	0.22% (0.16 to 0.29)	0.26% (0.19 to 0.35)	0.03% (-1.94 to 2.00)	1.16% (-0.93 to 3.33)	0.58% (-0.79 to 1.85)

Belarus	0.30% (0.23 to 0.39)	0.28% (0.22 to 0.36)	-0.11% (-2.02 to 1.68)	-0.34% (-2.11 to 1.53)	-0.22% (-1.46 to 0.90)	0.24% (0.17 to 0.33)	0.22% (0.16 to 0.30)	-0.02% (-2.21 to 2.36)	-0.62% (-2.89 to 1.38)	-0.31% (-1.65 to 1.15)
Estonia	0.32% (0.24 to 0.43)	0.58% (0.43 to 0.76)	0.28% (-1.62 to 2.14)	3.96% (2.01 to 5.84)	2.06% (0.89 to 3.30)	0.25% (0.18 to 0.35)	0.35% (0.26 to 0.49)	0.10% (-2.12 to 2.15)	2.16% (-0.14 to 4.76)	1.09% (-0.43 to 2.57)
Latvia	0.24% (0.19 to 0.33)	0.28% (0.21 to 0.38)	0.08% (-1.88 to 2.02)	0.87% (-1.23 to 3.07)	0.46% (-0.91 to 1.81)	0.24% (0.17 to 0.36)	0.27% (0.18 to 0.38)	0.04% (-2.55 to 2.51)	0.54% (-2.25 to 3.09)	0.28% (-1.46 to 2.03)
Lithuania	0.20% (0.16 to 0.26)	0.24% (0.19 to 0.31)	-0.01% (-1.76 to 1.74)	1.15% (-0.61 to 2.98)	0.55% (-0.56 to 1.75)	0.18% (0.14 to 0.25)	0.20% (0.15 to 0.28)	0.03% (-1.97 to 1.96)	0.64% (-1.54 to 2.81)	0.32% (-1.00 to 1.62)
Republic of Moldova	0.29% (0.23 to 0.37)	0.28% (0.22 to 0.36)	-0.07% (-1.77 to 1.67)	-0.23% (-2.13 to 1.51)	-0.15% (-1.31 to 1.05)	0.23% (0.17 to 0.31)	0.23% (0.16 to 0.31)	-0.09% (-2.28 to 2.16)	-0.05% (-2.30 to 2.28)	-0.07% (-1.56 to 1.36)
Russian Federation	0.35% (0.26 to 0.48)	0.35% (0.26 to 0.46)	-0.03% (-1.97 to 2.20)	-0.04% (-2.17 to 2.07)	-0.03% (-1.36 to 1.31)	0.32% (0.23 to 0.47)	0.33% (0.23 to 0.45)	0.00% (-2.65 to 2.52)	0.08% (-2.41 to 2.58)	0.04% (-1.54 to 1.72)
Ukraine	0.37% (0.29 to 0.48)	0.39% (0.30 to 0.50)	-0.02% (-1.77 to 1.57)	0.29% (-1.53 to 2.18)	0.13% (-1.08 to 1.28)	0.22% (0.16 to 0.30)	0.23% (0.16 to 0.31)	0.04% (-2.21 to 2.00)	0.18% (-1.97 to 2.42)	0.10% (-1.29 to 1.51)
Brunei Darussalam	0.80% (0.65 to 0.98)	0.82% (0.67 to 1.00)	-0.06% (-1.50 to 1.41)	0.19% (-1.34 to 1.63)	0.06% (-0.88 to 1.06)	0.88% (0.67 to 1.16)	0.92% (0.68 to 1.22)	0.07% (-2.06 to 2.17)	0.23% (-1.85 to 2.17)	0.15% (-1.15 to 1.41)
Japan	0.67% (0.53 to 0.81)	0.67% (0.54 to 0.83)	-0.98% (-2.50 to 0.41)	1.11% (-0.38 to 2.58)	0.03% (-0.89 to 1.01)	0.32% (0.24 to 0.43)	0.46% (0.34 to 0.60)	0.43% (-1.61 to 2.35)	2.09% (-0.04 to 4.13)	1.23% (-0.15 to 2.54)
Republic of Korea	0.58% (0.47 to 0.70)	0.58% (0.47 to 0.71)	-0.66% (-2.13 to 0.80)	0.71% (-0.78 to 2.20)	0.00% (-0.97 to 0.97)	0.31% (0.23 to 0.41)	0.37% (0.28 to 0.48)	0.15% (-1.74 to 2.10)	1.21% (-0.74 to 3.24)	0.66% (-0.71 to 1.93)
Singapore	0.66% (0.53 to 0.80)	0.67% (0.54 to 0.83)	-0.95% (-2.42 to 0.50)	1.14% (-0.42 to 2.63)	0.06% (-0.87 to 1.04)	0.32% (0.24 to 0.43)	0.46% (0.35 to 0.61)	0.50% (-1.42 to 2.42)	2.08% (-0.13 to 4.14)	1.27% (-0.13 to 2.62)
Australia	0.61% (0.49 to 0.76)	0.55% (0.44 to 0.68)	-1.54% (-3.01 to - 0.16)	0.84% (-0.59 to 2.27)	-0.39% (-1.29 to 0.48)	0.23% (0.17 to 0.32)	0.27% (0.20 to 0.37)	-0.01% (-2.14 to 2.04)	1.11% (-1.16 to 3.35)	0.53% (-0.88 to 2.01)
New Zealand	0.50% (0.41 to 0.61)	0.49% (0.39 to 0.60)	-0.94% (-2.33 to 0.44)	0.74% (-0.74 to 2.24)	-0.13% (-1.04 to 0.81)	0.44% (0.31 to 0.61)	0.50% (0.36 to 0.70)	0.13% (-2.24 to 2.40)	0.83% (-1.49 to 3.25)	0.47% (-1.04 to 1.96)
Andorra	0.20% (0.16 to 0.24)	0.23% (0.18 to 0.28)	0.08% (-1.37 to 1.46)	0.88% (-0.60 to 2.43)	0.46% (-0.50 to 1.38)	0.15% (0.11 to 0.19)	0.16% (0.12 to 0.21)	0.06% (-1.87 to 1.94)	0.50% (-1.40 to 2.37)	0.27% (-0.99 to 1.54)
Austria	0.21% (0.17 to 0.26)	0.26% (0.21 to 0.32)	0.40% (-1.10 to 1.67)	1.10% (-0.49 to 2.55)	0.74% (-0.21 to 1.64)	0.15% (0.11 to 0.20)	0.16% (0.12 to 0.21)	0.07% (-1.89 to 2.05)	0.53% (-1.47 to 2.54)	0.29% (-1.02 to 1.48)
Belgium	0.14% (0.11 to 0.17)	0.14% (0.12 to 0.18)	0.00% (-1.49 to 1.51)	0.14% (-1.30 to 1.65)	0.07% (-0.94 to 1.01)	0.14% (0.10 to 0.18)	0.14% (0.10 to 0.18)	-0.05% (-2.01 to 1.84)	0.06% (-2.04 to 2.01)	0.01% (-1.26 to 1.21)
Cyprus	0.15% (0.13 to 0.19)	0.16% (0.13 to 0.20)	-0.07% (-1.47 to 1.35)	0.27% (-1.26 to 1.80)	0.09% (-0.84 to 0.97)	0.13% (0.10 to 0.18)	0.14% (0.10 to 0.18)	0.00% (-2.00 to 1.95)	0.13% (-1.79 to 2.14)	0.06% (-1.31 to 1.36)
Denmark	0.24% (0.19 to 0.28)	0.27% (0.22 to 0.34)	0.16% (-1.20 to 1.56)	0.82% (-0.63 to 2.33)	0.48% (-0.44 to 1.44)	0.15% (0.12 to 0.20)	0.18% (0.13 to 0.23)	0.15% (-1.64 to 2.10)	0.78% (-1.23 to 2.72)	0.45% (-0.79 to 1.76)
Finland	0.27% (0.21 to 0.34)	0.36% (0.28 to 0.45)	0.59% (-1.13 to 2.16)	1.52% (-0.24 to 3.18)	1.04% (-0.03 to 2.13)	0.14% (0.10 to 0.19)	0.15% (0.12 to 0.20)	0.07% (-1.85 to 1.90)	0.66% (-1.24 to 2.60)	0.36% (-0.90 to 1.52)
France	0.14% (0.12 to 0.18)	0.14% (0.12 to 0.18)	-0.06% (-1.57 to 1.48)	0.17% (-1.34 to 1.66)	0.05% (-0.87 to 0.97)	0.13% (0.10 to 0.17)	0.13% (0.10 to 0.18)	-0.02% (-1.84 to 2.07)	0.09% (-1.73 to 2.04)	0.03% (-1.37 to 1.31)
Germany	0.17% (0.14 to 0.20)	0.21% (0.17 to 0.26)	0.18% (-1.24 to 1.64)	1.46% (-0.09 to 2.96)	0.80% (-0.11 to 1.68)	0.14% (0.11 to 0.19)	0.16% (0.12 to 0.22)	0.06% (-1.96 to 2.12)	0.82% (-1.14 to 2.70)	0.43% (-0.85 to 1.83)
Greece	0.15% (0.12 to 0.18)	0.16% (0.13 to 0.19)	0.09% (-1.37 to 1.63)	0.13% (-1.29 to 1.72)	0.11% (-0.84 to 1.10)	0.14% (0.11 to 0.19)	0.15% (0.11 to 0.19)	0.12% (-1.73 to 1.96)	0.09% (-1.97 to 2.15)	0.11% (-1.17 to 1.39)
Iceland	0.49% (0.41 to 0.61)	0.93% (0.74 to 1.14)	0.78% (-0.70 to 2.33)	3.66% (2.19 to 5.18)	2.17% (1.21 to 3.11)	0.25% (0.19 to 0.32)	0.36% (0.27 to 0.48)	0.40% (-1.37 to 2.37)	2.08% (0.03 to 3.98)	1.21% (0.04 to 2.43)
Ireland	0.14% (0.11 to 0.18)	0.15% (0.12 to 0.18)	0.00% (-1.46 to 1.44)	0.15% (-1.35 to 1.61)	0.07% (-0.89 to 1.12)	0.13% (0.10 to 0.18)	0.14% (0.10 to 0.18)	0.01% (-1.85 to 1.96)	0.10% (-2.01 to 2.14)	0.05% (-1.14 to 1.31)
Israel	0.20% (0.16 to 0.24)	0.23% (0.18 to 0.28)	0.06% (-1.42 to 1.49)	0.88% (-0.66 to 2.29)	0.45% (-0.40 to 1.31)	0.15% (0.11 to 0.19)	0.16% (0.12 to 0.21)	0.04% (-2.02 to 2.04)	0.46% (-1.36 to 2.45)	0.24% (-0.99 to 1.52)
Italy	0.16% (0.13 to 0.20)	0.17% (0.14 to 0.21)	0.22% (-1.35 to 1.67)	0.29% (-1.13 to 1.81)	0.25% (-0.73 to 1.24)	0.13% (0.10 to 0.18)	0.14% (0.10 to 0.18)	0.02% (-2.02 to 1.90)	0.13% (-1.95 to 2.22)	0.07% (-1.20 to 1.35)
Luxembourg	0.14% (0.12 to 0.17)	0.15% (0.12 to 0.18)	0.00% (-1.50 to 1.33)	0.20% (-1.24 to 1.65)	0.10% (-0.88 to 1.01)	0.13% (0.10 to 0.18)	0.14% (0.10 to 0.18)	-0.05% (-2.01 to 1.89)	0.17% (-1.91 to 2.20)	0.05% (-1.28 to 1.33)

Malta	0.16% (0.13 to 0.19)	0.17% (0.13 to 0.21)	-0.11% (-1.54 to 1.27)	0.58% (-0.87 to 2.20)	0.22% (-0.81 to 1.21)	0.13% (0.10 to 0.18)	0.14% (0.10 to 0.18)	0.02% (-1.93 to 1.90)	0.12% (-1.79 to 2.17)	0.07% (-1.18 to 1.35)
Netherlands	0.14% (0.12 to 0.17)	0.14% (0.12 to 0.18)	-0.10% (-1.45 to 1.25)	0.17% (-1.28 to 1.81)	0.03% (-0.86 to 1.05)	0.13% (0.10 to 0.18)	0.13% (0.10 to 0.17)	-0.03% (-1.95 to 1.82)	0.06% (-2.13 to 2.20)	0.01% (-1.30 to 1.31)
Norway	0.56% (0.45 to 0.68)	1.17% (0.95 to 1.43)	0.92% (-0.52 to 2.33)	4.35% (2.93 to 5.78)	2.58% (1.66 to 3.50)	0.29% (0.22 to 0.38)	0.48% (0.36 to 0.62)	0.61% (-1.48 to 2.58)	2.85% (1.01 to 4.84)	1.69% (0.33 to 2.97)
Portugal	0.14% (0.11 to 0.17)	0.15% (0.12 to 0.18)	0.04% (-1.38 to 1.45)	0.34% (-1.20 to 1.82)	0.18% (-0.80 to 1.10)	0.13% (0.10 to 0.18)	0.14% (0.11 to 0.18)	0.02% (-1.94 to 1.85)	0.19% (-1.77 to 2.03)	0.10% (-1.10 to 1.32)
Spain	0.14% (0.11 to 0.17)	0.14% (0.12 to 0.17)	0.00% (-1.40 to 1.45)	0.17% (-1.36 to 1.57)	0.08% (-0.86 to 1.03)	0.13% (0.10 to 0.17)	0.14% (0.10 to 0.18)	0.03% (-1.86 to 1.79)	0.06% (-1.95 to 2.00)	0.04% (-1.28 to 1.21)
Sweden	1.71% (1.39 to 2.10)	1.72% (1.39 to 2.12)	0.22% (-1.29 to 1.75)	-0.20% (-1.74 to 1.37)	0.02% (-0.97 to 1.00)	0.35% (0.26 to 0.47)	0.39% (0.28 to 0.52)	0.71% (-1.39 to 2.60)	-0.11% (-2.35 to 2.16)	0.31% (-1.07 to 1.66)
Switzerland	0.21% (0.17 to 0.26)	0.26% (0.21 to 0.33)	0.18% (-1.37 to 1.80)	1.40% (-0.15 to 3.03)	0.77% (-0.25 to 1.80)	0.15% (0.11 to 0.21)	0.17% (0.12 to 0.22)	0.04% (-1.78 to 2.02)	0.57% (-1.36 to 2.52)	0.30% (-1.02 to 1.62)
United Kingdom	0.16% (0.13 to 0.19)	0.16% (0.13 to 0.19)	-0.22% (-1.72 to 1.33)	0.12% (-1.40 to 1.59)	-0.05% (-0.99 to 0.98)	0.13% (0.10 to 0.17)	0.14% (0.10 to 0.18)	0.01% (-1.77 to 1.84)	0.13% (-1.80 to 2.16)	0.07% (-1.09 to 1.27)
Argentina	0.16% (0.12 to 0.20)	0.15% (0.12 to 0.19)	-0.03% (-1.63 to 1.71)	-0.25% (-1.88 to 1.51)	-0.14% (-1.23 to 1.01)	0.26% (0.18 to 0.36)	0.25% (0.17 to 0.35)	0.01% (-2.29 to 2.46)	-0.27% (-2.94 to 2.29)	-0.13% (-1.75 to 1.52)
Chile	0.28% (0.22 to 0.34)	0.21% (0.17 to 0.27)	-1.13% (-2.63 to 0.28)	-0.67% (-2.21 to 0.83)	-0.91% (-1.86 to 0.12)	0.22% (0.16 to 0.30)	0.22% (0.16 to 0.29)	-0.05% (-2.25 to 1.99)	-0.06% (-2.28 to 2.17)	-0.06% (-1.48 to 1.35)
Uruguay	0.18% (0.14 to 0.22)	0.16% (0.13 to 0.21)	-0.05% (-1.64 to 1.42)	-0.51% (-2.05 to 1.11)	-0.27% (-1.29 to 0.77)	0.21% (0.16 to 0.28)	0.20% (0.15 to 0.27)	-0.06% (-2.02 to 1.93)	-0.22% (-2.53 to 1.85)	-0.14% (-1.53 to 1.22)
Canada	1.72% (1.40 to 2.12)	1.38% (1.11 to 1.69)	-0.51% (-1.98 to 0.98)	-1.01% (-2.56 to 0.53)	-0.75% (-1.75 to 0.22)	0.25% (0.19 to 0.32)	0.22% (0.17 to 0.29)	-0.27% (-2.11 to 1.65)	-0.49% (-2.58 to 1.46)	-0.38% (-1.62 to 0.88)
United States of America	4.59% (4.06 to 5.21)	4.07% (3.32 to 4.91)	-0.41% (-1.66 to 0.84)	-0.44% (-1.78 to 0.85)	-0.43% (-1.25 to 0.30)	0.36% (0.27 to 0.46)	0.39% (0.29 to 0.52)	0.17% (-1.74 to 2.12)	0.41% (-1.53 to 2.53)	0.29% (-0.97 to 1.59)
Antigua and Barbuda	0.83% (0.60 to 1.14)	0.83% (0.62 to 1.10)	0.09% (-2.05 to 2.19)	-0.12% (-2.32 to 2.31)	-0.01% (-1.43 to 1.40)	0.38% (0.27 to 0.56)	0.38% (0.27 to 0.54)	0.12% (-2.37 to 2.73)	-0.13% (-2.80 to 2.72)	0.00% (-1.63 to 1.60)
Bahamas	0.55% (0.41 to 0.75)	0.58% (0.43 to 0.81)	0.04% (-2.12 to 2.15)	0.32% (-1.74 to 2.43)	0.17% (-1.22 to 1.58)	0.16% (0.12 to 0.22)	0.16% (0.12 to 0.21)	-0.05% (-2.01 to 2.08)	0.00% (-2.13 to 2.04)	-0.03% (-1.26 to 1.28)
Barbados	0.35% (0.26 to 0.52)	0.43% (0.30 to 0.61)	0.10% (-2.60 to 2.57)	1.22% (-1.42 to 3.80)	0.64% (-1.13 to 2.35)	0.23% (0.17 to 0.33)	0.25% (0.18 to 0.35)	0.00% (-2.38 to 2.20)	0.40% (-2.12 to 2.81)	0.20% (-1.33 to 1.65)
Belize	0.65% (0.48 to 0.89)	0.74% (0.54 to 0.98)	0.09% (-1.93 to 2.03)	0.80% (-1.17 to 2.81)	0.44% (-0.93 to 1.75)	0.29% (0.21 to 0.40)	0.31% (0.22 to 0.42)	0.00% (-2.25 to 2.14)	0.35% (-2.09 to 2.74)	0.17% (-1.26 to 1.70)
Cuba	0.49% (0.38 to 0.65)	0.54% (0.41 to 0.69)	0.08% (-1.82 to 1.88)	0.53% (-1.26 to 2.46)	0.30% (-0.88 to 1.49)	0.27% (0.20 to 0.36)	0.29% (0.20 to 0.40)	0.04% (-2.35 to 2.21)	0.49% (-1.98 to 2.82)	0.26% (-1.19 to 1.64)
Dominica	0.40% (0.30 to 0.52)	0.41% (0.31 to 0.53)	-0.01% (-1.91 to 1.86)	0.25% (-1.70 to 2.21)	0.11% (-1.06 to 1.29)	0.16% (0.12 to 0.20)	0.16% (0.12 to 0.22)	0.09% (-1.88 to 1.95)	0.30% (-1.74 to 2.46)	0.19% (-1.15 to 1.49)
Dominican Republic	1.18% (0.92 to 1.49)	1.44% (1.13 to 1.82)	0.54% (-1.20 to 2.28)	0.90% (-0.90 to 2.71)	0.71% (-0.33 to 1.84)	0.30% (0.23 to 0.39)	0.29% (0.22 to 0.39)	0.12% (-1.83 to 2.09)	-0.20% (-2.30 to 1.82)	-0.03% (-1.31 to 1.26)
Grenada	0.66% (0.53 to 0.83)	0.67% (0.52 to 0.86)	0.00% (-1.61 to 1.48)	0.13% (-1.60 to 1.84)	0.06% (-0.98 to 1.11)	0.22% (0.16 to 0.31)	0.23% (0.17 to 0.32)	0.02% (-2.17 to 2.30)	0.36% (-1.94 to 2.74)	0.18% (-1.32 to 1.70)
Guyana	0.48% (0.38 to 0.60)	0.54% (0.42 to 0.70)	0.08% (-1.61 to 1.64)	0.79% (-0.98 to 2.64)	0.42% (-0.65 to 1.59)	0.27% (0.20 to 0.37)	0.31% (0.22 to 0.44)	0.07% (-2.15 to 2.37)	0.71% (-1.70 to 3.31)	0.38% (-1.11 to 1.97)
Haiti	0.46% (0.37 to 0.57)	0.44% (0.35 to 0.54)	-0.19% (-1.81 to 1.31)	-0.11% (-1.68 to 1.42)	-0.15% (-1.15 to 0.83)	0.37% (0.28 to 0.50)	0.39% (0.30 to 0.52)	0.07% (-2.05 to 2.11)	0.35% (-1.78 to 2.47)	0.21% (-1.15 to 1.48)
Jamaica	0.57% (0.43 to 0.76)	0.64% (0.49 to 0.83)	0.10% (-1.84 to 1.98)	0.72% (-1.24 to 2.66)	0.40% (-0.81 to 1.64)	0.27% (0.20 to 0.37)	0.29% (0.21 to 0.39)	0.05% (-2.29 to 2.40)	0.45% (-2.19 to 2.64)	0.24% (-1.32 to 1.77)
Saint Lucia	0.65% (0.49 to 0.86)	0.73% (0.55 to 1.02)	0.07% (-1.90 to 2.11)	0.78% (-1.37 to 2.95)	0.41% (-0.95 to 1.79)	0.24% (0.17 to 0.35)	0.26% (0.18 to 0.37)	0.08% (-2.50 to 2.68)	0.47% (-2.20 to 3.18)	0.26% (-1.42 to 1.90)
Saint Vincent and the Grenadines	0.33% (0.24 to 0.45)	0.32% (0.23 to 0.43)	0.08% (-2.08 to 2.15)	-0.34% (-2.49 to 1.74)	-0.12% (-1.49 to 1.23)	0.20% (0.15 to 0.29)	0.19% (0.14 to 0.27)	0.01% (-2.31 to 2.30)	-0.25% (-2.68 to 2.05)	-0.11% (-1.61 to 1.35)
Suriname	0.82% (0.61 to 1.12)	0.85% (0.62 to 1.12)	0.12% (-2.01 to 2.15)	0.16% (-2.18 to 2.41)	0.14% (-1.23 to 1.51)	0.32% (0.22 to 0.45)	0.31% (0.22 to 0.43)	0.10% (-2.40 to 2.61)	-0.23% (-2.63 to 2.20)	-0.06% (-1.59 to 1.43)

Trinidad and Tobago	0.57% (0.41 to 0.78)	0.57% (0.41 to 0.79)	0.01% (-2.16 to 2.31)	0.06% (-2.37 to 2.40)	0.03% (-1.49 to 1.54)	0.29% (0.20 to 0.42)	0.29% (0.20 to 0.45)	-0.05% (-2.56 to 2.42)	0.02% (-2.33 to 2.58)	-0.02% (-1.64 to 1.73)
Bolivia (Plurinational State of)	1.02% (0.76 to 1.34)	0.93% (0.69 to 1.27)	0.00% (-1.91 to 1.95)	-0.63% (-2.64 to 1.61)	-0.31% (-1.64 to 1.12)	0.62% (0.43 to 0.91)	0.58% (0.39 to 0.83)	-0.02% (-2.50 to 2.43)	-0.55% (-3.18 to 1.98)	-0.27% (-1.91 to 1.40)
Ecuador	0.23% (0.18 to 0.31)	0.22% (0.17 to 0.29)	-0.01% (-1.95 to 2.03)	-0.32% (-2.22 to 1.55)	-0.16% (-1.33 to 1.03)	0.20% (0.14 to 0.27)	0.19% (0.14 to 0.26)	0.04% (-2.19 to 2.22)	-0.20% (-2.49 to 2.00)	-0.07% (-1.45 to 1.37)
Peru	0.75% (0.57 to 0.97)	0.65% (0.49 to 0.85)	-0.01% (-1.83 to 1.91)	-1.01% (-2.98 to 0.96)	-0.49% (-1.72 to 0.75)	0.67% (0.47 to 0.97)	0.55% (0.39 to 0.77)	-0.03% (-2.55 to 2.45)	-1.39% (-4.03 to 1.12)	-0.68% (-2.35 to 1.00)
Colombia	0.67% (0.51 to 0.84)	0.68% (0.52 to 0.87)	0.03% (-1.68 to 1.72)	0.06% (-1.85 to 1.84)	0.05% (-1.12 to 1.19)	0.47% (0.33 to 0.67)	0.49% (0.35 to 0.69)	0.04% (-2.29 to 2.32)	0.28% (-2.19 to 2.93)	0.16% (-1.50 to 1.61)
Costa Rica	0.25% (0.19 to 0.32)	0.24% (0.19 to 0.30)	0.00% (-1.62 to 1.57)	-0.16% (-1.89 to 1.47)	-0.08% (-1.11 to 0.94)	0.21% (0.15 to 0.28)	0.20% (0.15 to 0.27)	0.01% (-2.15 to 2.22)	-0.15% (-2.45 to 2.07)	-0.07% (-1.52 to 1.33)
El Salvador	0.63% (0.49 to 0.78)	0.61% (0.48 to 0.78)	-0.06% (-1.77 to 1.75)	-0.08% (-1.89 to 1.76)	-0.07% (-1.19 to 1.11)	0.44% (0.32 to 0.61)	0.44% (0.33 to 0.62)	0.02% (-2.37 to 2.28)	0.02% (-2.31 to 2.45)	0.02% (-1.53 to 1.53)
Guatemala	0.30% (0.24 to 0.39)	0.30% (0.23 to 0.38)	0.00% (-1.69 to 1.61)	0.03% (-1.64 to 1.71)	0.02% (-1.12 to 1.15)	0.27% (0.20 to 0.36)	0.27% (0.19 to 0.36)	0.03% (-2.00 to 2.09)	-0.04% (-2.20 to 2.03)	0.00% (-1.30 to 1.46)
Honduras	0.44% (0.36 to 0.56)	0.45% (0.36 to 0.56)	-0.03% (-1.58 to 1.45)	0.12% (-1.35 to 1.60)	0.04% (-0.93 to 1.02)	0.28% (0.21 to 0.37)	0.29% (0.21 to 0.40)	-0.02% (-2.12 to 2.01)	0.32% (-1.85 to 2.55)	0.14% (-1.23 to 1.62)
Mexico	0.49% (0.39 to 0.61)	0.47% (0.37 to 0.58)	-0.04% (-1.62 to 1.63)	-0.27% (-1.99 to 1.44)	-0.15% (-1.20 to 0.94)	0.32% (0.23 to 0.44)	0.32% (0.24 to 0.44)	0.08% (-2.27 to 2.28)	-0.07% (-2.19 to 2.18)	0.01% (-1.45 to 1.44)
Nicaragua	0.80% (0.61 to 1.02)	0.79% (0.61 to 1.07)	0.03% (-1.97 to 2.00)	-0.06% (-2.03 to 1.81)	-0.01% (-1.20 to 1.24)	0.52% (0.37 to 0.73)	0.52% (0.36 to 0.74)	-0.02% (-2.42 to 2.47)	0.09% (-2.63 to 2.72)	0.03% (-1.60 to 1.53)
Panama	0.40% (0.32 to 0.50)	0.40% (0.31 to 0.51)	-0.02% (-1.58 to 1.59)	0.03% (-1.66 to 1.66)	0.01% (-1.00 to 1.08)	0.34% (0.25 to 0.46)	0.35% (0.26 to 0.48)	0.08% (-2.15 to 2.31)	0.16% (-2.33 to 2.53)	0.12% (-1.33 to 1.62)
Venezuela (Bolivarian Republic of)	2.18% (1.73 to 2.72)	2.14% (1.72 to 2.63)	0.02% (-1.43 to 1.49)	-0.15% (-1.75 to 1.35)	-0.06% (-1.10 to 0.85)	0.51% (0.39 to 0.68)	0.50% (0.38 to 0.67)	-0.01% (-2.00 to 2.04)	-0.02% (-2.27 to 2.08)	-0.02% (-1.37 to 1.30)
Brazil	0.42% (0.34 to 0.52)	0.46% (0.37 to 0.58)	0.20% (-1.25 to 1.63)	0.54% (-0.99 to 2.12)	0.37% (-0.65 to 1.30)	0.33% (0.25 to 0.42)	0.33% (0.25 to 0.44)	0.07% (-1.74 to 1.79)	0.07% (-1.79 to 1.94)	0.07% (-1.16 to 1.31)
Paraguay	1.60% (1.29 to 1.93)	1.61% (1.31 to 1.96)	0.00% (-1.47 to 1.41)	0.05% (-1.45 to 1.47)	0.02% (-0.89 to 1.02)	0.99% (0.77 to 1.29)	0.99% (0.76 to 1.26)	0.00% (-1.70 to 1.72)	-0.03% (-1.81 to 1.71)	-0.01% (-1.22 to 1.17)
Algeria	7.99% (6.54 to 9.55)	7.66% (6.34 to 9.24)	0.17% (-1.05 to 1.48)	-0.48% (-1.79 to 0.85)	-0.15% (-0.98 to 0.72)	0.44% (0.33 to 0.57)	0.36% (0.28 to 0.46)	-0.07% (-1.77 to 1.63)	-1.33% (-3.15 to 0.53)	-0.68% (-1.88 to 0.50)
Bahrain	1.81% (1.46 to 2.23)	1.68% (1.32 to 2.08)	-0.07% (-1.69 to 1.38)	-0.46% (-2.08 to 1.25)	-0.26% (-1.30 to 0.73)	0.47% (0.35 to 0.62)	0.39% (0.29 to 0.53)	-0.38% (-2.24 to 1.58)	-0.91% (-2.87 to 1.09)	-0.63% (-1.89 to 0.70)
Egypt	0.84% (0.68 to 1.03)	0.73% (0.59 to 0.88)	0.01% (-1.32 to 1.43)	-1.08% (-2.66 to 0.42)	-0.52% (-1.44 to 0.36)	0.23% (0.18 to 0.30)	0.23% (0.17 to 0.30)	-0.01% (-1.90 to 1.99)	-0.19% (-2.43 to 1.90)	-0.10% (-1.31 to 1.24)
Iran (Islamic Republic of)	1.64% (1.32 to 2.00)	1.47% (1.17 to 1.82)	-0.13% (-1.61 to 1.34)	-0.63% (-2.24 to 0.89)	-0.37% (-1.35 to 0.61)	0.41% (0.31 to 0.53)	0.32% (0.24 to 0.42)	-0.38% (-2.15 to 1.41)	-1.23% (-3.15 to 0.74)	-0.79% (-2.08 to 0.46)
Iraq	0.69% (0.53 to 0.89)	0.60% (0.47 to 0.76)	-0.07% (-1.76 to 1.58)	-0.97% (-2.72 to 0.76)	-0.51% (-1.66 to 0.64)	0.25% (0.19 to 0.34)	0.23% (0.17 to 0.32)	-0.04% (-2.08 to 2.05)	-0.57% (-2.91 to 1.60)	-0.29% (-1.71 to 1.07)
Jordan	2.03% (1.63 to 2.52)	1.85% (1.44 to 2.38)	-0.05% (-1.56 to 1.55)	-0.62% (-2.40 to 1.12)	-0.33% (-1.39 to 0.73)	0.48% (0.36 to 0.63)	0.38% (0.29 to 0.51)	-0.33% (-2.31 to 1.52)	-1.25% (-3.24 to 0.78)	-0.77% (-2.09 to 0.55)
Kuwait	1.74% (1.38 to 2.14)	1.61% (1.29 to 2.04)	-0.04% (-1.55 to 1.44)	-0.49% (-2.10 to 1.15)	-0.26% (-1.28 to 0.76)	0.23% (0.17 to 0.30)	0.22% (0.16 to 0.30)	-0.18% (-2.24 to 2.07)	-0.29% (-2.34 to 1.79)	-0.23% (-1.63 to 1.09)
Lebanon	1.53% (1.23 to 1.86)	1.35% (1.09 to 1.67)	-0.16% (-1.69 to 1.31)	-0.72% (-2.29 to 0.78)	-0.43% (-1.41 to 0.55)	0.49% (0.37 to 0.65)	0.40% (0.29 to 0.54)	-0.33% (-2.32 to 1.53)	-1.21% (-3.25 to 0.90)	-0.76% (-2.07 to 0.56)
Libya	1.35% (1.08 to 1.68)	1.32% (1.04 to 1.67)	-0.05% (-1.61 to 1.58)	-0.09% (-1.73 to 1.51)	-0.07% (-1.15 to 1.03)	0.37% (0.27 to 0.49)	0.34% (0.25 to 0.47)	-0.13% (-2.30 to 2.17)	-0.40% (-2.69 to 1.92)	-0.26% (-1.70 to 1.14)
Morocco	1.83% (1.46 to 2.28)	1.70% (1.35 to 2.10)	-0.07% (-1.52 to 1.42)	-0.44% (-2.10 to 1.19)	-0.25% (-1.26 to 0.79)	0.49% (0.37 to 0.66)	0.42% (0.31 to 0.58)	-0.30% (-2.26 to 1.72)	-0.77% (-2.97 to 1.49)	-0.53% (-1.96 to 0.92)
Palestine	1.98% (1.56 to 2.49)	1.91% (1.48 to 2.46)	0.02% (-1.64 to 1.58)	-0.27% (-1.91 to 1.41)	-0.12% (-1.18 to 0.94)	0.55% (0.41 to 0.74)	0.49% (0.35 to 0.70)	-0.32% (-2.34 to 1.96)	-0.49% (-2.78 to 2.05)	-0.40% (-1.85 to 1.12)
Oman	1.30% (1.01 to 1.65)	1.20% (0.93 to 1.53)	0.01% (-1.75 to 1.70)	-0.58% (-2.29 to 1.14)	-0.28% (-1.43 to 0.83)	0.24% (0.18 to 0.34)	0.22% (0.15 to 0.29)	-0.07% (-2.32 to 2.25)	-0.77% (-3.14 to 1.50)	-0.41% (-1.89 to 0.99)

Qatar	1.08% (0.79 to 1.47)	1.03% (0.77 to 1.41)	0.20% (-1.91 to 2.32)	-0.50% (-2.81 to 1.67)	-0.14% (-1.50 to 1.23)	0.30% (0.21 to 0.43)	0.27% (0.20 to 0.38)	-0.06% (-2.50 to 2.29)	-0.51% (-3.07 to 1.89)	-0.28% (-1.81 to 1.21)
Saudi Arabia	1.02% (0.80 to 1.29)	1.02% (0.80 to 1.29)	-0.01% (-1.52 to 1.65)	-0.06% (-1.73 to 1.67)	-0.03% (-1.16 to 1.05)	0.32% (0.24 to 0.43)	0.29% (0.22 to 0.40)	-0.09% (-2.09 to 1.83)	-0.62% (-2.60 to 1.50)	-0.35% (-1.59 to 0.90)
Syrian Arab Republic	0.33% (0.26 to 0.43)	0.50% (0.37 to 0.66)	1.39% (-0.50 to 3.33)	1.43% (-0.60 to 3.43)	1.41% (0.18 to 2.59)	0.26% (0.18 to 0.36)	0.31% (0.22 to 0.44)	0.84% (-1.57 to 3.31)	0.35% (-2.28 to 2.96)	0.61% (-0.90 to 2.23)
Tunisia	4.85% (4.00 to 5.79)	3.77% (3.08 to 4.56)	-0.67% (-1.91 to 0.62)	-1.09% (-2.40 to 0.31)	-0.87% (-1.71 to 0.01)	1.35% (1.02 to 1.76)	1.05% (0.79 to 1.34)	-0.55% (-2.26 to 1.40)	-1.23% (-3.03 to 0.64)	-0.88% (-2.06 to 0.34)
Turkey	0.62% (0.49 to 0.80)	0.54% (0.42 to 0.69)	-0.15% (-1.91 to 1.56)	-0.90% (-2.75 to 0.77)	-0.51% (-1.61 to 0.61)	0.23% (0.17 to 0.31)	0.19% (0.14 to 0.26)	-0.21% (-2.44 to 2.09)	-0.86% (-3.08 to 1.30)	-0.52% (-1.90 to 0.92)
United Arab Emirates	1.90% (1.52 to 2.37)	1.78% (1.39 to 2.24)	-0.04% (-1.58 to 1.42)	-0.45% (-2.00 to 1.18)	-0.24% (-1.22 to 0.77)	0.51% (0.38 to 0.67)	0.43% (0.32 to 0.59)	-0.28% (-2.36 to 1.97)	-0.86% (-3.23 to 1.21)	-0.56% (-1.96 to 0.89)
Yemen	14.09% (11.85 to 16.60)	10.73% (9.02 to 12.83)	0.32% (-0.83 to 1.51)	-2.30% (-3.47 to -1.13)	-0.94% (-1.72 to -0.14)	5.94% (4.78 to 7.28)	4.35% (3.37 to 5.43)	0.55% (-0.95 to 2.24)	-2.83% (-4.44 to -1.19)	-1.08% (-2.17 to 0.03)
Afghanistan	8.50% (6.74 to 10.35)	10.31% (8.59 to 12.27)	0.59% (-0.87 to 2.12)	0.76% (-0.75 to 2.22)	0.67% (-0.28 to 1.63)	0.42% (0.33 to 0.54)	0.37% (0.28 to 0.48)	-0.24% (-2.02 to 1.57)	-0.69% (-2.66 to 1.36)	-0.46% (-1.68 to 0.79)
Bangladesh	21.86% (19.04 to 24.69)	21.98% (19.91 to 24.15)	0.32% (-0.59 to 1.23)	-0.30% (-1.14 to 0.53)	0.02% (-0.53 to 0.60)	27.88% (24.37 to 31.73)	25.39% (23.11 to 27.78)	0.36% (-0.57 to 1.29)	-1.04% (-1.81 to -0.25)	-0.32% (-0.92 to 0.29)
Bhutan	25.87% (22.11 to 29.77)	27.15% (24.12 to 30.09)	0.13% (-0.82 to 1.15)	0.21% (-0.81 to 1.21)	0.17% (-0.45 to 0.79)	13.76% (11.23 to 16.58)	14.22% (11.83 to 16.67)	0.10% (-1.23 to 1.40)	0.14% (-1.23 to 1.58)	0.12% (-0.75 to 0.99)
India	27.68% (24.33 to 31.24)	25.98% (23.64 to 28.52)	0.23% (-0.70 to 1.12)	-0.69% (-1.49 to 0.14)	-0.21% (-0.76 to 0.37)	11.79% (9.75 to 14.18)	11.53% (10.03 to 13.14)	0.09% (-1.26 to 1.39)	-0.24% (-1.47 to 1.00)	-0.07% (-0.87 to 0.74)
Nepal	37.05% (32.86 to 41.48)	39.16% (36.12 to 42.41)	0.29% (-0.48 to 1.06)	0.09% (-0.63 to 0.82)	0.19% (-0.27 to 0.71)	8.63% (6.88 to 10.43)	8.21% (6.64 to 9.86)	0.11% (-1.27 to 1.54)	-0.47% (-2.00 to 0.98)	-0.17% (-1.10 to 0.79)
Pakistan	14.35% (11.96 to 16.96)	14.03% (12.11 to 16.22)	0.10% (-0.95 to 1.25)	-0.26% (-1.55 to 1.08)	-0.07% (-0.82 to 0.70)	5.14% (4.07 to 6.56)	4.91% (3.94 to 6.01)	-0.12% (-1.69 to 1.33)	-0.19% (-1.81 to 1.40)	-0.15% (-1.18 to 0.86)
Angola	0.58% (0.46 to 0.72)	0.58% (0.46 to 0.72)	0.02% (-1.44 to 1.56)	0.04% (-1.54 to 1.71)	0.03% (-1.04 to 1.08)	0.35% (0.26 to 0.46)	0.34% (0.26 to 0.45)	0.04% (-1.88 to 1.97)	-0.13% (-1.98 to 1.69)	-0.04% (-1.26 to 1.17)
Central African Republic	0.53% (0.43 to 0.65)	0.53% (0.43 to 0.65)	-0.03% (-1.49 to 1.32)	0.03% (-1.48 to 1.45)	0.00% (-0.95 to 0.97)	0.87% (0.65 to 1.13)	0.81% (0.62 to 1.03)	0.07% (-1.79 to 1.98)	-0.61% (-2.59 to 1.31)	-0.26% (-1.41 to 1.00)
Congo	0.44% (0.34 to 0.54)	0.44% (0.35 to 0.55)	0.10% (-1.53 to 1.66)	0.01% (-1.64 to 1.69)	0.06% (-0.98 to 1.15)	0.88% (0.66 to 1.16)	0.88% (0.67 to 1.14)	-0.01% (-2.05 to 1.72)	0.01% (-1.94 to 2.16)	0.00% (-1.31 to 1.29)
Democratic Republic of the Congo	0.69% (0.56 to 0.83)	0.68% (0.55 to 0.83)	-0.02% (-1.35 to 1.42)	0.01% (-1.37 to 1.38)	-0.01% (-1.00 to 0.88)	1.97% (1.50 to 2.56)	1.63% (1.25 to 2.11)	0.63% (-1.17 to 2.41)	-2.05% (-3.95 to 0.05)	-0.66% (-1.90 to 0.64)
Equatorial Guinea	0.54% (0.44 to 0.66)	0.53% (0.43 to 0.66)	-0.04% (-1.48 to 1.39)	-0.04% (-1.53 to 1.54)	-0.04% (-1.01 to 0.87)	0.87% (0.66 to 1.18)	0.81% (0.63 to 1.06)	0.07% (-1.91 to 1.94)	-0.58% (-2.53 to 1.19)	-0.25% (-1.55 to 0.95)
Gabon	0.27% (0.21 to 0.34)	0.27% (0.22 to 0.34)	-0.01% (-1.70 to 1.72)	0.14% (-1.53 to 1.85)	0.07% (-1.02 to 1.21)	0.50% (0.38 to 0.65)	0.51% (0.38 to 0.65)	0.07% (-1.83 to 2.02)	0.05% (-1.81 to 1.83)	0.06% (-1.19 to 1.31)
Burundi	3.49% (2.80 to 4.26)	2.81% (2.28 to 3.44)	1.32% (-0.01 to 2.72)	-2.97% (-4.51 to -1.40)	-0.75% (-1.66 to 0.20)	2.29% (1.72 to 3.06)	2.22% (1.69 to 2.88)	0.55% (-1.41 to 2.37)	-0.82% (-2.80 to 1.30)	-0.11% (-1.44 to 1.15)
Comoros	6.28% (5.15 to 7.62)	6.29% (5.24 to 7.47)	0.29% (-1.00 to 1.50)	-0.29% (-1.69 to 1.07)	0.01% (-0.95 to 0.88)	4.69% (3.53 to 6.08)	4.51% (3.44 to 5.76)	0.09% (-1.61 to 2.04)	-0.38% (-2.35 to 1.51)	-0.14% (-1.33 to 1.10)
Djibouti	1.62% (1.30 to 2.01)	1.55% (1.24 to 1.93)	0.00% (-1.51 to 1.50)	-0.29% (-1.93 to 1.28)	-0.14% (-1.16 to 0.79)	1.15% (0.87 to 1.47)	1.14% (0.86 to 1.48)	0.04% (-1.71 to 1.92)	-0.05% (-1.90 to 1.96)	-0.01% (-1.15 to 1.26)
Eritrea	3.26% (2.66 to 4.00)	2.88% (2.32 to 3.51)	0.02% (-1.37 to 1.43)	-0.90% (-2.35 to 0.54)	-0.43% (-1.33 to 0.53)	0.26% (0.19 to 0.34)	0.27% (0.20 to 0.35)	-0.02% (-1.77 to 1.90)	0.13% (-1.92 to 2.08)	0.06% (-1.16 to 1.44)
Ethiopia	2.14% (1.73 to 2.61)	2.11% (1.74 to 2.57)	0.44% (-1.01 to 1.80)	-0.57% (-1.97 to 0.80)	-0.05% (-1.02 to 0.90)	0.43% (0.32 to 0.57)	0.41% (0.31 to 0.54)	0.03% (-1.77 to 1.92)	-0.33% (-2.46 to 1.60)	-0.15% (-1.44 to 1.04)
Kenya	1.36% (1.10 to 1.68)	1.39% (1.12 to 1.70)	0.01% (-1.42 to 1.47)	0.18% (-1.24 to 1.66)	0.09% (-0.90 to 1.02)	1.18% (0.90 to 1.54)	1.18% (0.91 to 1.55)	0.03% (-1.92 to 1.96)	-0.03% (-1.99 to 1.96)	0.00% (-1.22 to 1.26)
Madagascar	16.19% (13.58 to 19.01)	16.98% (14.66 to 19.30)	1.06% (0.04 to 2.17)	-0.79% (-1.88 to 0.37)	0.17% (-0.59 to 0.94)	6.29% (4.72 to 8.08)	6.38% (4.91 to 7.97)	0.73% (-0.99 to 2.48)	-0.66% (-2.49 to 1.42)	0.06% (-1.21 to 1.35)
Malawi	0.53% (0.42 to 0.66)	0.51% (0.41 to 0.63)	0.08% (-1.34 to 1.65)	-0.35% (-1.92 to 1.14)	-0.13% (-1.11 to 0.86)	1.30% (1.01 to 1.71)	1.18% (0.89 to 1.53)	0.08% (-1.79 to 1.92)	-0.83% (-2.81 to 1.08)	-0.36% (-1.63 to 0.82)

Mauritius	1.60% (1.32 to 1.94)	1.81% (1.46 to 2.18)	0.57% (-0.82 to 1.88)	0.24% (-1.26 to 1.70)	0.41% (-0.48 to 1.30)	3.23% (2.51 to 4.13)	2.84% (2.19 to 3.62)	-0.11% (-1.75 to 1.53)	-0.81% (-2.62 to 1.05)	-0.45% (-1.57 to 0.76)
Mozambique	1.24% (1.01 to 1.53)	1.23% (0.98 to 1.53)	-0.05% (-1.43 to 1.45)	-0.02% (-1.50 to 1.51)	-0.03% (-1.05 to 1.02)	2.65% (2.00 to 3.51)	2.34% (1.76 to 3.11)	0.06% (-1.91 to 1.99)	-0.97% (-2.97 to 1.02)	-0.44% (-1.70 to 0.88)
Rwanda	0.30% (0.24 to 0.38)	0.30% (0.24 to 0.36)	0.00% (-1.61 to 1.46)	-0.13% (-1.81 to 1.43)	-0.07% (-1.11 to 0.98)	2.44% (1.85 to 3.17)	2.42% (1.89 to 3.09)	0.06% (-1.74 to 1.96)	-0.12% (-2.04 to 1.81)	-0.03% (-1.23 to 1.20)
Seychelles	0.87% (0.69 to 1.09)	0.81% (0.64 to 1.03)	0.06% (-1.60 to 1.73)	-0.54% (-2.29 to 1.18)	-0.23% (-1.32 to 0.90)	1.38% (1.05 to 1.82)	1.13% (0.84 to 1.50)	-0.28% (-2.21 to 1.64)	-1.12% (-3.07 to 0.96)	-0.69% (-1.93 to 0.61)
Somalia	1.30% (1.04 to 1.60)	1.21% (0.98 to 1.48)	-0.02% (-1.47 to 1.39)	-0.49% (-2.01 to 1.01)	-0.24% (-1.17 to 0.68)	1.01% (0.77 to 1.30)	1.00% (0.75 to 1.29)	0.02% (-1.92 to 1.88)	-0.10% (-2.20 to 1.72)	-0.04% (-1.32 to 1.25)
United Republic of Tanzania	0.79% (0.64 to 0.96)	0.77% (0.63 to 0.94)	0.08% (-1.19 to 1.39)	-0.27% (-1.81 to 1.21)	-0.09% (-1.01 to 0.80)	0.74% (0.57 to 0.96)	0.77% (0.59 to 0.98)	0.01% (-1.72 to 1.86)	0.24% (-1.60 to 2.10)	0.13% (-1.04 to 1.41)
Uganda	0.65% (0.52 to 0.80)	0.68% (0.54 to 0.85)	0.13% (-1.45 to 1.54)	0.18% (-1.48 to 1.75)	0.15% (-0.89 to 1.15)	0.97% (0.75 to 1.24)	0.91% (0.70 to 1.18)	0.10% (-1.65 to 1.84)	-0.52% (-2.44 to 1.38)	-0.20% (-1.42 to 0.93)
Zambia	0.65% (0.52 to 0.79)	0.73% (0.60 to 0.89)	0.07% (-1.34 to 1.56)	0.82% (-0.55 to 2.24)	0.43% (-0.51 to 1.37)	1.08% (0.83 to 1.38)	1.20% (0.93 to 1.55)	0.04% (-1.87 to 1.85)	0.71% (-1.10 to 2.55)	0.36% (-0.85 to 1.54)
Botswana	0.95% (0.75 to 1.19)	0.87% (0.69 to 1.12)	0.06% (-1.59 to 1.73)	-0.64% (-2.39 to 1.01)	-0.28% (-1.42 to 0.82)	6.49% (5.07 to 8.19)	6.54% (5.32 to 7.92)	0.45% (-1.13 to 2.07)	-0.41% (-2.21 to 1.39)	0.03% (-0.99 to 1.18)
Lesotho	1.42% (1.12 to 1.76)	1.39% (1.12 to 1.69)	0.09% (-1.43 to 1.56)	-0.25% (-1.88 to 1.22)	-0.07% (-1.07 to 0.90)	0.57% (0.43 to 0.73)	0.59% (0.44 to 0.77)	0.01% (-1.93 to 1.89)	0.28% (-1.68 to 2.48)	0.14% (-1.13 to 1.43)
Namibia	0.91% (0.74 to 1.16)	0.82% (0.66 to 1.02)	0.13% (-1.39 to 1.57)	-0.92% (-2.44 to 0.74)	-0.38% (-1.38 to 0.62)	0.95% (0.73 to 1.22)	0.92% (0.72 to 1.19)	0.11% (-1.68 to 1.98)	-0.28% (-2.15 to 1.60)	-0.08% (-1.23 to 1.07)
South Africa	1.19% (0.88 to 1.56)	0.85% (0.65 to 1.13)	-0.51% (-2.57 to 1.48)	-1.85% (-3.89 to -0.01)	-1.16% (-2.51 to 0.08)	2.04% (1.53 to 2.62)	2.00% (1.56 to 2.57)	-0.23% (-2.08 to 1.65)	0.11% (-1.81 to 1.90)	-0.06% (-1.19 to 1.17)
Eswatini	0.54% (0.43 to 0.66)	0.48% (0.39 to 0.60)	0.03% (-1.36 to 1.47)	-0.82% (-2.37 to 0.69)	-0.38% (-1.36 to 0.58)	0.27% (0.20 to 0.36)	0.28% (0.21 to 0.37)	-0.02% (-2.05 to 2.02)	0.36% (-1.65 to 2.35)	0.17% (-1.11 to 1.46)
Zimbabwe	0.41% (0.33 to 0.52)	0.38% (0.30 to 0.47)	-0.02% (-1.54 to 1.59)	-0.61% (-2.34 to 0.99)	-0.31% (-1.36 to 0.77)	0.51% (0.39 to 0.68)	0.63% (0.47 to 0.84)	0.00% (-1.81 to 1.92)	1.40% (-0.64 to 3.49)	0.68% (-0.58 to 1.92)
Benin	2.76% (2.26 to 3.35)	2.93% (2.39 to 3.58)	0.01% (-1.35 to 1.32)	0.41% (-1.02 to 1.81)	0.20% (-0.67 to 1.07)	0.84% (0.66 to 1.10)	0.83% (0.64 to 1.07)	0.11% (-1.70 to 1.92)	-0.20% (-2.16 to 1.64)	-0.04% (-1.21 to 1.18)
Burkina Faso	2.41% (1.95 to 2.93)	2.43% (1.99 to 2.96)	0.38% (-1.07 to 1.73)	-0.36% (-2.01 to 1.21)	0.02% (-0.96 to 0.98)	3.51% (2.66 to 4.53)	3.64% (2.84 to 4.64)	0.44% (-1.35 to 2.34)	-0.19% (-2.13 to 1.81)	0.14% (-1.03 to 1.32)
Cameroon	0.72% (0.59 to 0.87)	0.72% (0.60 to 0.89)	0.01% (-1.36 to 1.38)	0.05% (-1.30 to 1.35)	0.03% (-0.83 to 0.93)	1.09% (0.83 to 1.40)	1.12% (0.84 to 1.44)	-0.01% (-1.84 to 1.92)	0.19% (-1.72 to 2.03)	0.09% (-1.14 to 1.28)
Cabo Verde	1.50% (1.20 to 1.86)	1.40% (1.13 to 1.72)	0.00% (-1.50 to 1.48)	-0.52% (-1.87 to 1.04)	-0.25% (-1.20 to 0.69)	1.78% (1.37 to 2.30)	1.68% (1.27 to 2.22)	0.05% (-1.73 to 1.92)	-0.49% (-2.49 to 1.47)	-0.21% (-1.49 to 0.99)
Chad	1.32% (1.07 to 1.61)	1.32% (1.08 to 1.59)	0.02% (-1.47 to 1.43)	-0.04% (-1.54 to 1.42)	-0.01% (-0.96 to 0.94)	1.30% (1.00 to 1.68)	1.34% (1.02 to 1.71)	0.13% (-1.77 to 1.96)	0.12% (-1.83 to 2.12)	0.12% (-1.05 to 1.39)
Côte d'Ivoire	0.42% (0.34 to 0.52)	0.42% (0.34 to 0.51)	0.05% (-1.32 to 1.40)	-0.05% (-1.50 to 1.36)	0.00% (-0.88 to 0.93)	0.85% (0.64 to 1.09)	0.83% (0.64 to 1.08)	0.00% (-1.87 to 1.75)	-0.18% (-2.13 to 1.80)	-0.09% (-1.29 to 1.11)
Gambia	0.98% (0.80 to 1.20)	1.04% (0.84 to 1.28)	0.10% (-1.26 to 1.55)	0.32% (-1.12 to 1.79)	0.21% (-0.74 to 1.16)	0.67% (0.51 to 0.86)	0.65% (0.49 to 0.84)	0.04% (-1.88 to 1.93)	-0.24% (-2.16 to 1.61)	-0.10% (-1.31 to 1.17)
Ghana	1.03% (0.83 to 1.25)	0.95% (0.77 to 1.17)	0.18% (-1.23 to 1.59)	-0.79% (-2.26 to 0.74)	-0.29% (-1.26 to 0.66)	0.62% (0.48 to 0.79)	0.66% (0.51 to 0.87)	0.03% (-1.75 to 1.76)	0.40% (-1.41 to 2.35)	0.21% (-0.99 to 1.46)
Guinea	0.89% (0.72 to 1.07)	0.84% (0.68 to 1.03)	-0.01% (-1.35 to 1.46)	-0.40% (-1.80 to 1.03)	-0.20% (-1.14 to 0.72)	0.84% (0.65 to 1.08)	0.83% (0.64 to 1.07)	-0.01% (-1.82 to 1.94)	-0.10% (-1.90 to 1.72)	-0.06% (-1.20 to 1.17)
Guinea-Bissau	0.89% (0.72 to 1.07)	0.84% (0.68 to 1.04)	-0.02% (-1.33 to 1.46)	-0.42% (-1.98 to 1.01)	-0.21% (-1.19 to 0.77)	0.85% (0.64 to 1.09)	0.83% (0.63 to 1.08)	-0.04% (-1.85 to 1.73)	-0.09% (-1.88 to 1.73)	-0.07% (-1.35 to 1.11)
Liberia	0.91% (0.73 to 1.11)	0.82% (0.66 to 1.03)	0.30% (-1.15 to 1.79)	-1.03% (-2.48 to 0.47)	-0.34% (-1.36 to 0.61)	0.50% (0.39 to 0.65)	0.50% (0.39 to 0.66)	0.01% (-1.95 to 1.85)	-0.04% (-1.78 to 1.84)	-0.01% (-1.16 to 1.13)
Mali	2.57% (2.05 to 3.21)	2.73% (2.23 to 3.29)	0.42% (-1.12 to 1.85)	-0.01% (-1.54 to 1.58)	0.22% (-0.82 to 1.20)	1.22% (0.92 to 1.61)	1.22% (0.93 to 1.59)	0.07% (-1.87 to 1.99)	-0.05% (-1.91 to 2.03)	0.01% (-1.29 to 1.26)
Mauritania	1.02% (0.82 to 1.25)	1.02% (0.81 to 1.26)	0.02% (-1.45 to 1.52)	-0.01% (-1.53 to 1.49)	0.00% (-1.00 to 0.96)	0.98% (0.76 to 1.27)	0.99% (0.76 to 1.27)	-0.05% (-2.01 to 1.76)	0.15% (-1.74 to 1.96)	0.05% (-1.22 to 1.24)

Niger	2.75% (2.22 to 3.37)	3.03% (2.44 to 3.65)	0.54% (-0.87 to 1.90)	0.12% (-1.41 to 1.65)	0.34% (-0.62 to 1.26)	1.14% (0.86 to 1.48)	1.31% (1.02 to 1.69)	0.40% (-1.47 to 2.29)	0.60% (-1.58 to 2.68)	0.50% (-0.68 to 1.75)
Nigeria	0.47% (0.38 to 0.58)	0.44% (0.36 to 0.54)	0.01% (-1.49 to 1.44)	-0.47% (-1.93 to 1.11)	-0.22% (-1.20 to 0.79)	0.36% (0.27 to 0.46)	0.35% (0.27 to 0.45)	0.05% (-1.75 to 1.79)	-0.16% (-1.97 to 1.72)	-0.05% (-1.23 to 1.12)
Sao Tome and Principe	0.47% (0.38 to 0.58)	0.45% (0.36 to 0.56)	0.01% (-1.46 to 1.54)	-0.37% (-1.86 to 1.17)	-0.17% (-1.15 to 0.82)	0.51% (0.39 to 0.65)	0.49% (0.37 to 0.64)	0.08% (-1.82 to 1.94)	-0.38% (-2.27 to 1.62)	-0.14% (-1.31 to 1.09)
Senegal	0.29% (0.23 to 0.35)	0.25% (0.20 to 0.31)	0.06% (-1.42 to 1.55)	-0.93% (-2.43 to 0.52)	-0.42% (-1.37 to 0.60)	0.65% (0.50 to 0.86)	0.62% (0.47 to 0.80)	-0.03% (-1.93 to 2.02)	-0.38% (-2.20 to 1.68)	-0.20% (-1.41 to 1.06)
Sierra Leone	1.27% (1.03 to 1.57)	1.21% (0.99 to 1.46)	0.04% (-1.35 to 1.32)	-0.38% (-1.74 to 1.04)	-0.16% (-1.13 to 0.73)	1.37% (1.06 to 1.73)	1.32% (1.02 to 1.69)	0.01% (-1.69 to 1.93)	-0.25% (-2.01 to 1.50)	-0.11% (-1.20 to 1.02)
Togo	0.83% (0.66 to 1.01)	0.76% (0.62 to 0.91)	-0.02% (-1.44 to 1.36)	-0.56% (-2.02 to 0.91)	-0.28% (-1.23 to 0.67)	1.04% (0.79 to 1.37)	0.99% (0.75 to 1.32)	0.00% (-1.92 to 1.87)	-0.30% (-2.21 to 1.49)	-0.15% (-1.37 to 1.03)
American Samoa	4.73% (3.64 to 6.10)	4.86% (3.76 to 6.23)	-0.05% (-1.78 to 1.60)	0.25% (-1.59 to 2.02)	0.10% (-1.06 to 1.32)	2.06% (1.43 to 2.91)	2.19% (1.54 to 3.08)	0.03% (-2.37 to 2.51)	0.39% (-1.98 to 2.90)	0.20% (-1.43 to 1.83)
Bermuda	0.46% (0.36 to 0.59)	0.52% (0.39 to 0.69)	0.17% (-1.71 to 1.98)	0.75% (-1.22 to 2.71)	0.45% (-0.85 to 1.62)	0.24% (0.18 to 0.32)	0.25% (0.19 to 0.34)	0.07% (-2.17 to 2.12)	0.33% (-1.86 to 2.59)	0.19% (-1.31 to 1.65)
Cook Islands	4.82% (3.67 to 6.11)	4.53% (3.55 to 5.67)	0.23% (-1.43 to 1.94)	-0.68% (-2.41 to 1.07)	-0.21% (-1.37 to 0.91)	2.35% (1.63 to 3.35)	2.22% (1.62 to 3.06)	0.33% (-1.94 to 2.63)	-0.77% (-3.25 to 1.66)	-0.20% (-1.74 to 1.38)
Greenland	3.56% (2.87 to 4.40)	3.45% (2.77 to 4.25)	-0.15% (-1.66 to 1.25)	-0.07% (-1.55 to 1.44)	-0.11% (-1.08 to 0.85)	0.33% (0.25 to 0.43)	0.36% (0.28 to 0.48)	0.22% (-1.80 to 2.13)	0.44% (-1.59 to 2.53)	0.33% (-0.90 to 1.51)
Guam	7.73% (6.11 to 9.73)	9.32% (7.82 to 11.05)	0.62% (-0.78 to 2.09)	0.69% (-0.88 to 2.29)	0.66% (-0.32 to 1.64)	2.76% (1.99 to 3.83)	3.54% (2.77 to 4.46)	1.10% (-1.07 to 3.11)	0.63% (-1.51 to 2.84)	0.87% (-0.56 to 2.22)
Monaco	0.20% (0.16 to 0.24)	0.23% (0.18 to 0.27)	0.09% (-1.47 to 1.64)	0.87% (-0.64 to 2.34)	0.47% (-0.47 to 1.48)	0.15% (0.11 to 0.19)	0.16% (0.12 to 0.21)	0.08% (-1.91 to 1.97)	0.46% (-1.56 to 2.45)	0.26% (-0.98 to 1.51)
Nauru	0.48% (0.37 to 0.60)	0.45% (0.35 to 0.56)	0.00% (-1.54 to 1.63)	-0.47% (-2.11 to 1.10)	-0.23% (-1.26 to 0.85)	0.34% (0.25 to 0.44)	0.32% (0.24 to 0.44)	-0.01% (-2.01 to 2.02)	-0.34% (-2.42 to 1.70)	-0.17% (-1.38 to 1.10)
Niue	0.53% (0.42 to 0.69)	0.56% (0.43 to 0.72)	-0.05% (-1.62 to 1.73)	0.42% (-1.24 to 2.21)	0.17% (-0.94 to 1.28)	0.50% (0.35 to 0.73)	0.51% (0.35 to 0.72)	-0.09% (-2.78 to 2.44)	0.18% (-2.52 to 2.63)	0.04% (-1.59 to 1.65)
Northern Mariana Islands	8.20% (6.38 to 10.50)	8.99% (7.18 to 11.31)	0.30% (-1.35 to 2.03)	0.35% (-1.45 to 2.07)	0.33% (-0.80 to 1.48)	4.50% (3.06 to 6.58)	5.23% (3.75 to 6.99)	0.61% (-1.98 to 3.22)	0.45% (-2.34 to 3.36)	0.54% (-1.15 to 2.23)
Palau	21.32% (17.86 to 25.14)	25.76% (22.37 to 29.75)	0.43% (-0.64 to 1.54)	0.90% (-0.25 to 2.15)	0.66% (-0.05 to 1.39)	18.28% (14.60 to 22.43)	24.42% (20.04 to 29.17)	0.70% (-0.97 to 2.25)	1.33% (-0.24 to 2.79)	1.00% (0.03 to 1.98)
Puerto Rico	0.40% (0.31 to 0.52)	0.44% (0.34 to 0.58)	-0.01% (-1.80 to 1.69)	0.64% (-1.26 to 2.55)	0.31% (-0.87 to 1.49)	0.28% (0.21 to 0.38)	0.27% (0.20 to 0.37)	-0.02% (-2.22 to 2.39)	-0.11% (-2.35 to 2.12)	-0.06% (-1.55 to 1.35)
Saint Kitts and Nevis	0.26% (0.20 to 0.34)	0.29% (0.22 to 0.38)	-0.01% (-1.82 to 1.72)	0.83% (-0.93 to 2.85)	0.40% (-0.81 to 1.67)	0.18% (0.13 to 0.24)	0.19% (0.14 to 0.27)	0.08% (-1.90 to 2.21)	0.40% (-1.75 to 2.64)	0.24% (-1.07 to 1.68)
San Marino	0.20% (0.16 to 0.24)	0.22% (0.18 to 0.27)	0.06% (-1.31 to 1.46)	0.70% (-0.80 to 2.16)	0.37% (-0.55 to 1.28)	0.14% (0.11 to 0.19)	0.15% (0.12 to 0.19)	0.08% (-1.84 to 1.95)	0.31% (-1.63 to 2.29)	0.19% (-1.02 to 1.48)
Tokelau	4.72% (3.61 to 6.05)	4.85% (3.76 to 6.09)	0.00% (-1.66 to 1.61)	0.19% (-1.71 to 1.91)	0.09% (-1.06 to 1.17)	2.07% (1.48 to 2.95)	2.18% (1.53 to 3.10)	0.04% (-2.40 to 2.37)	0.33% (-2.18 to 2.74)	0.18% (-1.34 to 1.80)
Tuvalu	4.71% (3.66 to 5.96)	4.87% (3.76 to 6.28)	0.00% (-1.78 to 1.62)	0.23% (-1.54 to 1.98)	0.11% (-1.13 to 1.24)	2.07% (1.43 to 2.89)	2.16% (1.54 to 3.03)	-0.02% (-2.38 to 2.45)	0.31% (-2.18 to 2.81)	0.14% (-1.36 to 1.67)
United States Virgin Islands	0.33% (0.25 to 0.43)	0.41% (0.31 to 0.54)	0.40% (-1.38 to 2.23)	1.18% (-0.87 to 3.02)	0.78% (-0.45 to 1.98)	0.19% (0.14 to 0.24)	0.20% (0.15 to 0.26)	0.12% (-1.81 to 2.06)	0.35% (-1.66 to 2.35)	0.23% (-1.01 to 1.55)
South Sudan	1.45% (1.18 to 1.76)	1.38% (1.12 to 1.68)	-0.03% (-1.38 to 1.33)	-0.30% (-1.77 to 1.14)	-0.16% (-1.05 to 0.75)	1.05% (0.81 to 1.35)	1.04% (0.78 to 1.35)	0.01% (-1.82 to 1.87)	-0.09% (-2.07 to 1.85)	-0.04% (-1.26 to 1.29)
Sudan	5.28% (4.23 to 6.55)	5.73% (4.64 to 6.96)	0.14% (-1.26 to 1.64)	0.45% (-1.11 to 1.93)	0.29% (-0.69 to 1.23)	0.24% (0.18 to 0.32)	0.21% (0.16 to 0.28)	-0.27% (-2.30 to 1.66)	-0.59% (-2.81 to 1.45)	-0.42% (-1.83 to 0.91)

Supplementary Table 2. Age-standardized prevalence of current smoked tobacco use in 1990 and 2019 for males and females aged 15 plus, as well as annualized rate of change. Locations organized by GBD region.

	Males					Females				
	Prevalence		Annualized Rate of Change (ARC)			Prevalence		Annualized Rate of Change (ARC)		
**Location**	**1990**	**2019**	**1990-2005**	**2005-2019**	**1990-2019**	**1990**	**2019**	**1990-2005**	**2005-2019**	**1990-2019**
China	60.82% (59.64 to 61.93)	49.73% (48.29 to 51.09)	-1.32% (-1.48 to -1.14)	-0.03% (-0.26 to 0.20)	-0.69% (-0.81 to -0.58)	4.49% (3.93 to 5.13)	3.54% (2.91 to 4.18)	-0.94% (-2.06 to 0.20)	-0.71% (-2.10 to 0.64)	-0.83% (-1.62 to -0.12)
Democratic People's Republic of Korea	47.62% (44.97 to 50.40)	43.55% (41.33 to 45.65)	0.10% (-0.29 to 0.48)	-0.75% (-1.14 to -0.36)	-0.31% (-0.57 to -0.03)	4.09% (3.14 to 5.27)	4.48% (3.57 to 5.69)	0.53% (-1.27 to 2.25)	0.11% (-1.76 to 1.93)	0.33% (-0.80 to 1.43)
Taiwan (Province of China)	48.44% (45.68 to 51.38)	39.60% (37.46 to 41.68)	-1.05% (-1.46 to -0.64)	-0.31% (-0.78 to 0.15)	-0.69% (-0.96 to -0.42)	7.05% (5.34 to 9.20)	4.98% (3.94 to 6.21)	-0.60% (-2.36 to 1.21)	-1.81% (-3.89 to 0.20)	-1.19% (-2.46 to -0.03)
Cambodia	50.62% (48.46 to 52.77)	40.62% (38.82 to 42.32)	-0.14% (-0.42 to 0.16)	-1.42% (-1.76 to -1.08)	-0.76% (-0.96 to -0.55)	5.97% (4.92 to 7.25)	5.09% (4.17 to 6.10)	0.22% (-1.12 to 1.60)	-1.38% (-2.75 to -0.04)	-0.55% (-1.46 to 0.35)
Indonesia	54.55% (53.03 to 56.06)	58.33% (56.98 to 59.57)	0.91% (0.70 to 1.11)	-0.49% (-0.68 to -0.32)	0.23% (0.11 to 0.35)	3.36% (2.78 to 4.04)	3.60% (3.00 to 4.31)	1.63% (0.27 to 2.98)	-1.26% (-2.67 to 0.24)	0.23% (-0.65 to 1.14)
Laos	45.06% (42.44 to 47.75)	49.10% (46.85 to 51.24)	-0.32% (-0.72 to 0.07)	0.96% (0.58 to 1.35)	0.30% (0.04 to 0.55)	5.49% (4.21 to 7.09)	6.98% (5.73 to 8.29)	0.03% (-1.56 to 1.65)	1.72% (0.01 to 3.40)	0.84% (-0.22 to 1.85)
Malaysia	51.85% (48.99 to 54.76)	40.26% (38.12 to 42.28)	-0.67% (-1.01 to -0.30)	-1.09% (-1.52 to -0.66)	-0.87% (-1.14 to -0.60)	4.40% (3.44 to 5.55)	3.20% (2.57 to 3.92)	-0.14% (-1.68 to 1.42)	-2.10% (-3.90 to -0.42)	-1.09% (-2.22 to 0.04)
Maldives	49.54% (46.69 to 52.38)	46.53% (43.81 to 48.95)	-1.19% (-1.56 to -0.83)	0.83% (0.37 to 1.30)	-0.22% (-0.49 to 0.03)	7.96% (6.28 to 9.92)	7.35% (5.88 to 9.03)	-1.06% (-2.56 to 0.43)	0.57% (-1.22 to 2.22)	-0.27% (-1.37 to 0.74)
Myanmar	60.58% (57.76 to 63.18)	41.05% (39.16 to 42.81)	-2.01% (-2.29 to -1.69)	-0.63% (-1.03 to -0.25)	-1.34% (-1.57 to -1.12)	16.87% (14.14 to 19.56)	8.59% (7.24 to 10.03)	-2.11% (-3.18 to -0.89)	-2.56% (-3.79 to -1.31)	-2.33% (-3.12 to -1.50)
Philippines	57.50% (55.04 to 60.02)	40.88% (39.29 to 42.49)	-0.84% (-1.16 to -0.53)	-1.53% (-1.86 to -1.20)	-1.18% (-1.38 to -0.97)	13.34% (11.19 to 15.93)	8.24% (7.24 to 9.31)	-0.56% (-1.76 to 0.67)	-2.82% (-3.91 to -1.79)	-1.66% (-2.44 to -0.88)
Sri Lanka	43.53% (41.01 to 46.01)	30.11% (28.52 to 31.75)	-2.55% (-2.97 to -2.14)	0.10% (-0.36 to 0.56)	-1.27% (-1.54 to -0.99)	3.09% (2.39 to 3.94)	1.80% (1.39 to 2.26)	-2.87% (-4.69 to -1.16)	-0.77% (-2.63 to 1.06)	-1.86% (-3.02 to -0.66)
Thailand	53.48% (51.15 to 55.88)	39.82% (38.49 to 41.30)	-1.64% (-1.95 to -1.36)	-0.35% (-0.66 to -0.02)	-1.02% (-1.20 to -0.83)	6.57% (5.41 to 7.82)	3.49% (2.89 to 4.19)	-2.40% (-3.77 to -0.90)	-1.96% (-3.43 to -0.51)	-2.19% (-3.15 to -1.30)
Timor-Leste	63.72% (61.09 to 66.36)	64.63% (62.72 to 66.62)	-0.11% (-0.39 to 0.17)	0.22% (-0.04 to 0.46)	0.05% (-0.12 to 0.22)	5.44% (4.32 to 6.81)	5.15% (4.18 to 6.21)	-0.24% (-1.78 to 1.36)	-0.12% (-1.72 to 1.52)	-0.19% (-1.24 to 0.82)
Viet Nam	59.11% (57.31 to 61.05)	47.07% (45.33 to 48.83)	-0.92% (-1.15 to -0.68)	-0.64% (-0.94 to -0.36)	-0.79% (-0.96 to -0.61)	4.03% (3.30 to 4.84)	2.68% (2.13 to 3.30)	-1.19% (-2.67 to 0.26)	-1.65% (-3.25 to 0.04)	-1.41% (-2.40 to -0.50)
Fiji	49.93% (47.08 to 52.41)	42.52% (39.97 to 45.39)	-0.72% (-1.07 to -0.33)	-0.38% (-0.84 to 0.07)	-0.55% (-0.84 to -0.29)	18.02% (14.60 to 21.93)	14.46% (12.00 to 17.30)	-1.12% (-2.54 to 0.24)	-0.37% (-1.83 to 1.02)	-0.76% (-1.67 to 0.21)
Kiribati	59.70% (57.11 to 62.44)	63.83% (61.71 to 65.91)	0.63% (0.34 to 0.92)	-0.20% (-0.47 to 0.05)	0.23% (0.03 to 0.43)	35.09% (30.10 to 40.14)	35.13% (31.52 to 38.57)	0.83% (-0.07 to 1.82)	-0.87% (-1.65 to -0.15)	0.01% (-0.63 to 0.67)
Marshall Islands	33.96% (31.51 to 36.46)	34.96% (32.29 to 37.52)	0.13% (-0.43 to 0.68)	0.06% (-0.48 to 0.58)	0.10% (-0.28 to 0.49)	7.78% (6.05 to 10.00)	9.75% (7.74 to 12.01)	0.18% (-1.57 to 1.98)	1.43% (-0.30 to 3.07)	0.78% (-0.33 to 1.94)
Micronesia (Federated States of)	60.04% (57.19 to 63.02)	62.24% (59.66 to 64.82)	0.07% (-0.25 to 0.38)	0.19% (-0.13 to 0.49)	0.12% (-0.08 to 0.32)	32.54% (27.65 to 37.79)	36.36% (30.98 to 41.53)	0.31% (-0.80 to 1.35)	0.46% (-0.69 to 1.65)	0.38% (-0.35 to 1.05)
Papua New Guinea	48.62% (45.84 to 51.63)	40.15% (37.74 to 42.64)	-0.15% (-0.53 to 0.23)	-1.20% (-1.66 to -0.74)	-0.66% (-0.94 to -0.37)	23.61% (18.95 to 28.07)	18.42% (15.77 to 21.27)	0.07% (-1.11 to 1.34)	-1.83% (-3.05 to -0.53)	-0.85% (-1.69 to 0.03)
Samoa	44.56% (41.88 to 47.27)	39.43% (37.24 to 41.58)	-0.07% (-0.48 to 0.36)	-0.80% (-1.24 to -0.32)	-0.42% (-0.70 to -0.15)	16.56% (13.26 to 20.26)	14.47% (12.47 to 16.62)	0.00% (-1.30 to 1.35)	-0.94% (-2.31 to 0.40)	-0.46% (-1.36 to 0.42)
Solomon Islands	50.24% (47.29 to 53.18)	52.16% (49.91 to 54.25)	-0.38% (-0.75 to 0.03)	0.68% (0.29 to 1.05)	0.13% (-0.10 to 0.38)	18.29% (14.64 to 22.32)	19.35% (16.95 to 21.79)	-0.50% (-1.77 to 0.82)	0.97% (-0.32 to 2.18)	0.21% (-0.62 to 1.06)

Tonga	54.68% (52.77 to 56.71)	42.45% (40.19 to 44.82)	-0.80% (-1.14 to -0.47)	-0.95% (-1.34 to -0.51)	-0.87% (-1.08 to -0.65)	13.57% (11.81 to 15.56)	13.33% (11.34 to 15.35)	0.18% (-1.02 to 1.31)	-0.33% (-1.72 to 0.96)	-0.06% (-0.79 to 0.69)
Vanuatu	43.35% (40.66 to 46.14)	38.19% (35.98 to 40.34)	0.14% (-0.25 to 0.55)	-1.06% (-1.57 to -0.57)	-0.44% (-0.74 to -0.14)	7.07% (5.36 to 9.14)	5.70% (4.54 to 6.95)	0.71% (-0.91 to 2.39)	-2.28% (-4.04 to -0.64)	-0.73% (-1.96 to 0.46)
Armenia	60.43% (58.14 to 62.58)	55.32% (53.56 to 57.05)	-0.22% (-0.48 to 0.02)	-0.39% (-0.66 to -0.12)	-0.30% (-0.49 to -0.14)	3.32% (2.53 to 4.25)	3.10% (2.48 to 3.79)	0.03% (-1.66 to 1.87)	-0.52% (-2.28 to 1.14)	-0.24% (-1.43 to 1.03)
Azerbaijan	44.28% (41.39 to 46.94)	43.55% (41.95 to 45.18)	0.32% (-0.05 to 0.71)	-0.46% (-0.83 to -0.10)	-0.06% (-0.31 to 0.19)	1.96% (1.42 to 2.59)	2.50% (1.86 to 3.30)	2.10% (0.14 to 4.05)	-0.49% (-2.52 to 1.66)	0.85% (-0.51 to 2.24)
Georgia	49.01% (46.20 to 51.63)	51.81% (49.95 to 53.62)	0.19% (-0.15 to 0.58)	0.20% (-0.14 to 0.52)	0.19% (-0.03 to 0.44)	6.40% (5.04 to 8.00)	7.03% (5.97 to 8.15)	0.41% (-1.21 to 1.99)	0.25% (-1.12 to 1.58)	0.33% (-0.63 to 1.29)
Kazakhstan	50.79% (48.76 to 52.81)	43.20% (41.31 to 45.03)	-0.10% (-0.38 to 0.21)	-1.05% (-1.43 to -0.69)	-0.56% (-0.76 to -0.37)	7.57% (6.14 to 9.35)	7.69% (6.46 to 9.16)	1.55% (-0.10 to 3.00)	-1.52% (-3.00 to 0.02)	0.06% (-1.01 to 0.99)
Kyrgyzstan	46.08% (44.66 to 47.54)	43.59% (41.74 to 45.53)	-0.48% (-0.75 to -0.22)	0.12% (-0.21 to 0.46)	-0.19% (-0.38 to -0.01)	3.17% (2.44 to 4.02)	4.65% (3.77 to 5.63)	1.96% (0.42 to 3.70)	0.65% (-1.11 to 2.34)	1.33% (0.25 to 2.48)
Mongolia	46.60% (44.28 to 48.69)	51.71% (49.59 to 53.90)	0.35% (0.03 to 0.71)	0.37% (0.05 to 0.69)	0.36% (0.14 to 0.59)	5.78% (4.67 to 7.15)	8.26% (6.86 to 9.84)	1.36% (-0.06 to 2.77)	1.10% (-0.41 to 2.63)	1.23% (0.25 to 2.19)
Tajikistan	31.26% (28.65 to 33.96)	16.92% (15.65 to 18.29)	-2.52% (-3.10 to -1.98)	-1.68% (-2.41 to -0.97)	-2.12% (-2.51 to -1.73)	2.26% (1.65 to 2.99)	1.29% (0.98 to 1.68)	-1.99% (-3.95 to -0.06)	-1.87% (-3.79 to 0.07)	-1.93% (-3.31 to -0.56)
Turkmenistan	40.35% (37.34 to 43.29)	21.56% (19.79 to 23.38)	-1.21% (-1.71 to -0.68)	-3.18% (-3.82 to -2.54)	-2.16% (-2.55 to -1.78)	4.97% (3.67 to 6.59)	3.05% (2.34 to 3.91)	-0.29% (-2.13 to 1.60)	-3.17% (-5.22 to -1.06)	-1.68% (-3.02 to -0.36)
Uzbekistan	18.25% (16.59 to 20.14)	24.82% (23.11 to 26.65)	2.20% (1.51 to 2.84)	-0.16% (-0.76 to 0.45)	1.06% (0.62 to 1.48)	1.34% (0.98 to 1.75)	1.94% (1.48 to 2.48)	2.69% (0.85 to 4.56)	-0.22% (-2.33 to 1.84)	1.28% (-0.01 to 2.67)
Albania	41.10% (38.49 to 43.86)	51.47% (49.73 to 53.10)	0.20% (-0.23 to 0.63)	1.40% (1.07 to 1.70)	0.78% (0.53 to 1.02)	6.56% (5.16 to 8.22)	11.95% (9.87 to 14.19)	2.07% (0.52 to 3.54)	2.09% (0.52 to 3.72)	2.08% (1.04 to 3.09)
Bosnia and Herzegovina	38.38% (36.05 to 40.92)	45.05% (42.64 to 47.49)	1.32% (0.85 to 1.76)	-0.26% (-0.66 to 0.16)	0.55% (0.25 to 0.84)	21.78% (18.33 to 25.34)	30.54% (26.83 to 34.24)	1.39% (0.33 to 2.59)	0.93% (-0.08 to 1.79)	1.17% (0.51 to 1.84)
Bulgaria	53.45% (50.71 to 56.21)	42.52% (40.39 to 44.57)	-0.65% (-0.98 to -0.31)	-0.94% (-1.35 to -0.54)	-0.79% (-1.04 to -0.56)	38.99% (33.26 to 44.57)	32.49% (29.55 to 35.42)	-0.49% (-1.36 to 0.49)	-0.76% (-1.67 to 0.05)	-0.62% (-1.21 to -0.01)
Croatia	47.51% (45.17 to 49.94)	39.12% (37.19 to 41.00)	-1.19% (-1.60 to -0.80)	-0.11% (-0.53 to 0.31)	-0.67% (-0.91 to -0.43)	37.09% (33.08 to 41.48)	32.65% (29.93 to 35.45)	-1.30% (-2.13 to -0.48)	0.49% (-0.28 to 1.21)	-0.44% (-0.92 to 0.03)
Czechia	43.55% (40.99 to 45.88)	34.07% (32.49 to 35.59)	-1.00% (-1.39 to -0.58)	-0.68% (-1.09 to -0.29)	-0.85% (-1.11 to -0.60)	29.17% (26.56 to 32.24)	23.22% (21.35 to 25.18)	-0.46% (-1.15 to 0.21)	-1.13% (-1.78 to -0.49)	-0.78% (-1.18 to -0.35)
Hungary	43.70% (41.71 to 45.66)	34.19% (32.05 to 36.11)	-0.41% (-0.78 to -0.03)	-1.31% (-1.76 to -0.87)	-0.85% (-1.09 to -0.58)	32.50% (29.85 to 35.52)	26.45% (23.76 to 29.25)	0.20% (-0.45 to 0.82)	-1.69% (-2.54 to -0.82)	-0.71% (-1.20 to -0.23)
North Macedonia	48.20% (45.16 to 51.07)	47.08% (45.05 to 49.20)	-0.20% (-0.57 to 0.21)	0.04% (-0.37 to 0.46)	-0.08% (-0.34 to 0.18)	29.97% (25.19 to 34.97)	31.05% (28.34 to 33.93)	-0.26% (-1.25 to 0.73)	0.55% (-0.44 to 1.52)	0.13% (-0.55 to 0.77)
Montenegro	40.96% (38.05 to 43.99)	37.49% (35.72 to 39.26)	0.48% (0.05 to 0.93)	-1.14% (-1.60 to -0.66)	-0.30% (-0.60 to -0.01)	32.71% (27.72 to 37.81)	32.66% (30.25 to 35.22)	0.90% (-0.03 to 1.95)	-0.95% (-1.85 to 0.02)	0.00% (-0.59 to 0.63)
Poland	53.45% (52.06 to 54.97)	31.80% (30.39 to 33.29)	-2.59% (-2.85 to -2.34)	-0.93% (-1.29 to -0.52)	-1.79% (-1.98 to -1.60)	29.71% (27.96 to 31.28)	24.41% (22.54 to 26.29)	-1.01% (-1.49 to -0.54)	-0.32% (-1.00 to 0.34)	-0.68% (-1.03 to -0.33)
Romania	47.31% (44.70 to 50.17)	38.37% (36.78 to 39.96)	-0.76% (-1.15 to -0.41)	-0.68% (-1.06 to -0.28)	-0.72% (-0.98 to -0.47)	21.63% (19.17 to 24.29)	23.47% (21.68 to 25.55)	0.16% (-0.70 to 1.03)	0.42% (-0.38 to 1.19)	0.29% (-0.21 to 0.77)
Serbia	41.48% (38.94 to 43.97)	38.83% (36.91 to 40.78)	0.11% (-0.29 to 0.54)	-0.59% (-0.98 to -0.22)	-0.23% (-0.50 to 0.05)	31.53% (27.59 to 35.81)	37.76% (34.49 to 41.10)	-0.09% (-0.93 to 0.83)	1.40% (0.72 to 2.03)	0.63% (0.08 to 1.16)
Slovakia	43.42% (40.60 to 46.09)	33.25% (31.21 to 35.32)	-1.13% (-1.55 to -0.70)	-0.69% (-1.23 to -0.17)	-0.92% (-1.23 to -0.61)	24.78% (20.49 to 29.54)	20.91% (18.70 to 23.29)	-0.61% (-1.69 to 0.59)	-0.54% (-1.48 to 0.37)	-0.58% (-1.30 to 0.17)
Slovenia	33.52% (30.85 to 36.08)	29.79% (28.05 to 31.54)	-0.71% (-1.20 to -0.20)	-0.08% (-0.62 to 0.46)	-0.41% (-0.73 to -0.05)	22.99% (19.34 to 27.17)	24.88% (22.63 to 27.36)	-0.07% (-1.22 to 1.03)	0.66% (-0.18 to 1.53)	0.28% (-0.41 to 0.92)
Belarus	52.95% (50.35 to 55.55)	50.19% (47.86 to 52.54)	-0.50% (-0.81 to -0.15)	0.15% (-0.27 to 0.54)	-0.18% (-0.43 to 0.05)	18.19% (14.50 to 22.72)	23.61% (20.66 to 26.63)	-0.25% (-1.63 to 1.13)	2.16% (0.87 to 3.38)	0.91% (0.02 to 1.81)
Estonia	44.18% (42.48 to 45.91)	31.68% (30.23 to 33.09)	0.06% (-0.22 to 0.33)	-2.45% (-2.80 to -2.08)	-1.15% (-1.36 to -0.94)	21.75% (19.90 to 23.75)	18.89% (17.16 to 20.70)	0.66% (0.07 to 1.30)	-1.72% (-2.46 to -1.01)	-0.49% (-0.98 to -0.05)
Latvia	53.91% (51.58 to 56.11)	46.95% (45.18 to 48.66)	-0.61% (-0.91 to -0.30)	-0.33% (-0.66 to -0.03)	-0.48% (-0.66 to -0.29)	22.12% (18.90 to 25.67)	21.26% (19.25 to 23.40)	-0.24% (-1.33 to 0.84)	-0.01% (-0.81 to 0.70)	-0.13% (-0.72 to 0.50)

Lithuania	46.57% (44.66 to 48.43)	37.91% (36.10 to 39.80)	-0.59% (-0.91 to -0.25)	-0.83% (-1.22 to -0.45)	-0.71% (-0.94 to -0.48)	15.83% (13.96 to 17.87)	20.31% (18.38 to 22.55)	0.73% (-0.15 to 1.63)	1.00% (0.22 to 1.81)	0.86% (0.34 to 1.41)
Republic of Moldova	39.18% (36.45 to 41.84)	40.64% (38.56 to 42.69)	0.60% (0.17 to 1.06)	-0.38% (-0.81 to 0.06)	0.13% (-0.15 to 0.42)	6.53% (5.21 to 8.07)	7.20% (5.93 to 8.68)	0.74% (-0.84 to 2.28)	-0.08% (-1.71 to 1.60)	0.34% (-0.65 to 1.35)
Russian Federation	55.31% (53.69 to 56.87)	45.55% (44.14 to 46.99)	0.36% (0.14 to 0.57)	-1.77% (-2.02 to -1.53)	-0.67% (-0.82 to -0.51)	12.37% (10.87 to 14.02)	15.54% (13.89 to 17.28)	2.71% (1.81 to 3.57)	-1.27% (-2.10 to -0.43)	0.79% (0.19 to 1.39)
Ukraine	58.55% (56.43 to 60.46)	41.96% (40.32 to 43.55)	-0.14% (-0.38 to 0.11)	-2.23% (-2.55 to -1.94)	-1.15% (-1.34 to -0.97)	15.79% (13.18 to 18.62)	14.44% (12.70 to 16.31)	0.57% (-0.59 to 1.74)	-1.24% (-2.21 to -0.29)	-0.30% (-1.04 to 0.40)
Brunei Darussalam	42.84% (40.30 to 45.40)	28.54% (26.05 to 31.20)	-2.61% (-3.14 to -2.09)	-0.11% (-0.71 to 0.47)	-1.40% (-1.78 to -1.03)	7.95% (6.33 to 9.93)	5.39% (4.28 to 6.78)	-2.71% (-4.36 to -0.96)	0.13% (-1.71 to 1.85)	-1.34% (-2.46 to -0.28)
Japan	57.37% (56.10 to 58.70)	33.44% (31.43 to 35.52)	-1.64% (-1.85 to -1.44)	-2.10% (-2.54 to -1.67)	-1.86% (-2.08 to -1.65)	13.38% (12.37 to 14.41)	10.21% (8.71 to 11.94)	0.40% (-0.20 to 1.00)	-2.37% (-3.48 to -1.16)	-0.94% (-1.50 to -0.35)
Republic of Korea	56.78% (54.34 to 58.98)	42.48% (40.13 to 44.75)	-1.15% (-1.42 to -0.84)	-0.85% (-1.31 to -0.43)	-1.00% (-1.25 to -0.75)	6.49% (5.08 to 8.29)	5.21% (4.14 to 6.58)	-0.40% (-2.11 to 1.23)	-1.12% (-2.84 to 0.61)	-0.75% (-1.93 to 0.38)
Singapore	30.11% (27.63 to 32.73)	20.63% (18.90 to 22.42)	-1.46% (-2.07 to -0.88)	-1.13% (-1.80 to -0.50)	-1.30% (-1.74 to -0.88)	10.26% (8.15 to 12.86)	6.82% (5.38 to 8.41)	-1.13% (-2.79 to 0.39)	-1.70% (-3.54 to -0.06)	-1.41% (-2.54 to -0.32)
Australia	31.59% (31.02 to 32.22)	16.41% (15.10 to 17.78)	-1.92% (-2.14 to -1.67)	-2.63% (-3.20 to -2.02)	-2.26% (-2.56 to -1.98)	27.01% (26.34 to 27.68)	14.36% (12.18 to 16.73)	-2.16% (-2.48 to -1.81)	-2.23% (-3.37 to -1.09)	-2.19% (-2.74 to -1.65)
New Zealand	28.57% (27.83 to 29.31)	18.41% (17.45 to 19.42)	-0.94% (-1.18 to -0.70)	-2.14% (-2.56 to -1.69)	-1.52% (-1.73 to -1.30)	27.46% (26.53 to 28.37)	15.16% (13.89 to 16.51)	-0.91% (-1.20 to -0.64)	-3.27% (-3.92 to -2.62)	-2.05% (-2.38 to -1.73)
Andorra	41.96% (39.19 to 44.74)	31.37% (28.56 to 34.16)	-0.98% (-1.53 to -0.42)	-1.04% (-1.59 to -0.45)	-1.00% (-1.37 to -0.65)	30.55% (26.38 to 35.16)	24.38% (19.59 to 29.46)	-0.76% (-2.09 to 0.42)	-0.82% (-2.20 to 0.43)	-0.79% (-1.65 to 0.05)
Austria	42.07% (40.93 to 43.34)	36.08% (33.99 to 38.28)	-0.17% (-0.44 to 0.11)	-0.92% (-1.38 to -0.48)	-0.53% (-0.77 to -0.30)	29.17% (27.68 to 30.62)	26.10% (23.38 to 28.99)	0.68% (0.26 to 1.11)	-1.53% (-2.37 to -0.68)	-0.39% (-0.78 to 0.01)
Belgium	41.08% (39.86 to 42.33)	24.91% (23.64 to 26.20)	-1.56% (-1.83 to -1.30)	-1.91% (-2.34 to -1.51)	-1.73% (-1.95 to -1.52)	30.00% (28.73 to 31.29)	21.44% (19.61 to 23.27)	-1.05% (-1.42 to -0.68)	-1.28% (-2.00 to -0.61)	-1.16% (-1.52 to -0.82)
Cyprus	47.44% (44.32 to 50.41)	44.82% (42.61 to 47.08)	0.12% (-0.27 to 0.55)	-0.53% (-0.95 to -0.10)	-0.20% (-0.46 to 0.10)	24.36% (20.02 to 29.30)	21.34% (18.68 to 24.26)	-0.37% (-1.48 to 0.72)	-0.53% (-1.64 to 0.61)	-0.45% (-1.27 to 0.32)
Denmark	44.59% (43.84 to 45.34)	22.65% (21.32 to 24.07)	-2.41% (-2.60 to -2.23)	-2.26% (-2.70 to -1.80)	-2.34% (-2.55 to -2.11)	44.85% (44.00 to 45.70)	22.64% (20.63 to 24.83)	-3.34% (-3.57 to -3.12)	-1.31% (-1.99 to -0.61)	-2.36% (-2.68 to -2.03)
Finland	34.36% (33.29 to 35.40)	24.76% (23.35 to 26.23)	-1.24% (-1.52 to -0.97)	-1.01% (-1.46 to -0.57)	-1.13% (-1.34 to -0.91)	26.36% (24.52 to 28.45)	18.11% (16.32 to 20.25)	-1.56% (-2.15 to -1.02)	-1.01% (-1.85 to -0.20)	-1.30% (-1.72 to -0.83)
France	44.52% (43.66 to 45.39)	36.86% (35.07 to 38.60)	-1.05% (-1.31 to -0.76)	-0.23% (-0.64 to 0.15)	-0.65% (-0.83 to -0.47)	31.65% (30.75 to 32.61)	31.27% (28.80 to 33.87)	-0.26% (-0.61 to 0.05)	0.19% (-0.48 to 0.84)	-0.04% (-0.34 to 0.25)
Germany	37.71% (36.74 to 38.69)	29.90% (28.41 to 31.45)	-0.55% (-0.78 to -0.32)	-1.07% (-1.45 to -0.69)	-0.80% (-1.00 to -0.61)	28.08% (27.08 to 29.12)	23.03% (21.10 to 24.92)	-0.36% (-0.67 to -0.06)	-1.04% (-1.65 to -0.42)	-0.69% (-1.00 to -0.38)
Greece	54.12% (53.07 to 55.19)	44.14% (42.27 to 46.08)	-0.63% (-0.81 to -0.43)	-0.79% (-1.14 to -0.43)	-0.70% (-0.86 to -0.54)	32.39% (31.24 to 33.58)	32.18% (29.50 to 35.10)	0.97% (0.62 to 1.28)	-1.09% (-1.75 to -0.45)	-0.03% (-0.36 to 0.29)
Iceland	32.49% (30.73 to 34.37)	15.78% (14.66 to 17.13)	-2.72% (-3.20 to -2.24)	-2.24% (-2.87 to -1.61)	-2.49% (-2.82 to -2.15)	29.06% (25.95 to 32.44)	15.15% (13.09 to 17.46)	-2.19% (-3.12 to -1.31)	-2.31% (-3.39 to -1.20)	-2.25% (-2.88 to -1.63)
Ireland	35.77% (34.98 to 36.59)	21.92% (20.25 to 23.60)	-1.11% (-1.37 to -0.83)	-2.31% (-2.90 to -1.78)	-1.69% (-1.99 to -1.42)	31.62% (30.70 to 32.54)	22.82% (20.39 to 25.29)	-0.71% (-1.05 to -0.39)	-1.58% (-2.48 to -0.74)	-1.13% (-1.52 to -0.75)
Israel	39.95% (37.60 to 42.12)	26.41% (24.60 to 28.33)	-1.71% (-2.19 to -1.27)	-1.13% (-1.68 to -0.55)	-1.43% (-1.74 to -1.13)	24.82% (21.54 to 28.46)	14.87% (12.75 to 17.16)	-2.70% (-3.71 to -1.63)	-0.77% (-2.07 to 0.47)	-1.77% (-2.48 to -1.10)
Italy	39.35% (38.49 to 40.16)	27.35% (25.85 to 28.84)	-1.52% (-1.69 to -1.34)	-0.98% (-1.40 to -0.57)	-1.26% (-1.47 to -1.05)	26.34% (25.52 to 27.23)	18.93% (17.33 to 20.63)	-1.01% (-1.28 to -0.75)	-1.28% (-1.96 to -0.61)	-1.14% (-1.46 to -0.82)
Luxembourg	38.33% (36.43 to 40.21)	26.19% (24.50 to 27.97)	-1.10% (-1.52 to -0.63)	-1.54% (-2.06 to -0.99)	-1.31% (-1.58 to -1.01)	29.61% (27.71 to 31.56)	21.32% (19.26 to 23.30)	-0.66% (-1.35 to 0.00)	-1.64% (-2.52 to -0.82)	-1.14% (-1.55 to -0.73)
Malta	42.04% (39.66 to 44.44)	26.89% (24.94 to 28.76)	-3.01% (-3.44 to -2.59)	0.03% (-0.54 to 0.56)	-1.54% (-1.87 to -1.21)	26.03% (22.71 to 29.22)	22.03% (19.22 to 24.89)	-2.11% (-2.98 to -1.10)	1.07% (0.04 to 2.06)	-0.58% (-1.27 to 0.08)
Netherlands	43.42% (42.34 to 44.65)	24.98% (23.59 to 26.44)	-1.60% (-1.82 to -1.41)	-2.23% (-2.65 to -1.81)	-1.91% (-2.13 to -1.70)	38.38% (37.16 to 39.54)	20.43% (18.64 to 22.24)	-2.16% (-2.41 to -1.90)	-2.20% (-2.85 to -1.53)	-2.18% (-2.51 to -1.85)
Norway	40.29% (38.46 to 42.11)	20.20% (18.67 to 21.97)	-2.18% (-2.69 to -1.71)	-2.60% (-3.22 to -1.95)	-2.38% (-2.71 to -2.05)	39.09% (36.58 to 41.58)	16.54% (14.34 to 18.94)	-3.60% (-4.29 to -2.95)	-2.30% (-3.39 to -1.21)	-2.97% (-3.51 to -2.41)

Portugal	41.05% (40.03 to 42.10)	33.37% (31.68 to 34.95)	-0.77% (-1.08 to -0.48)	-0.65% (-1.09 to -0.19)	-0.71% (-0.91 to -0.53)	17.20% (16.25 to 18.27)	22.45% (20.24 to 24.69)	0.90% (0.30 to 1.50)	0.93% (-0.02 to 1.83)	0.92% (0.52 to 1.29)
Spain	50.89% (49.98 to 51.81)	30.75% (29.10 to 32.46)	-0.98% (-1.17 to -0.78)	-2.55% (-2.97 to -2.14)	-1.74% (-1.93 to -1.54)	32.27% (31.31 to 33.29)	24.65% (22.61 to 26.82)	0.38% (0.10 to 0.68)	-2.33% (-3.00 to -1.63)	-0.93% (-1.24 to -0.61)
Sweden	22.36% (21.30 to 23.41)	12.37% (11.28 to 13.49)	-2.55% (-2.96 to -2.14)	-1.50% (-2.19 to -0.81)	-2.04% (-2.39 to -1.69)	27.24% (25.68 to 28.75)	14.40% (12.57 to 16.53)	-2.19% (-2.68 to -1.68)	-2.22% (-3.25 to -1.20)	-2.21% (-2.72 to -1.69)
Switzerland	38.26% (37.58 to 38.97)	29.26% (27.23 to 31.23)	-0.98% (-1.25 to -0.73)	-0.87% (-1.39 to -0.39)	-0.93% (-1.16 to -0.70)	28.82% (27.97 to 29.60)	23.36% (20.67 to 26.30)	-0.95% (-1.36 to -0.50)	-0.50% (-1.35 to 0.39)	-0.73% (-1.16 to -0.30)
United Kingdom	33.82% (33.31 to 34.37)	21.73% (20.43 to 22.93)	-1.25% (-1.39 to -1.11)	-1.82% (-2.28 to -1.40)	-1.53% (-1.75 to -1.33)	31.52% (30.91 to 32.03)	18.11% (16.30 to 19.95)	-1.39% (-1.55 to -1.23)	-2.48% (-3.26 to -1.75)	-1.92% (-2.28 to -1.57)
Argentina	35.44% (32.58 to 38.40)	29.18% (27.37 to 31.00)	-0.62% (-1.09 to -0.13)	-0.72% (-1.35 to -0.08)	-0.67% (-1.03 to -0.29)	25.58% (21.59 to 30.63)	19.65% (17.30 to 22.16)	-0.54% (-1.71 to 0.58)	-1.30% (-2.46 to -0.16)	-0.90% (-1.64 to -0.14)
Chile	41.64% (38.97 to 44.32)	36.19% (33.86 to 38.35)	-0.38% (-0.82 to 0.07)	-0.60% (-1.08 to -0.13)	-0.48% (-0.80 to -0.17)	37.58% (32.85 to 42.52)	32.65% (28.67 to 36.65)	-0.29% (-1.13 to 0.67)	-0.69% (-1.76 to 0.29)	-0.48% (-1.09 to 0.16)
Uruguay	40.37% (37.99 to 42.69)	31.05% (28.76 to 33.31)	-0.72% (-1.13 to -0.23)	-1.11% (-1.66 to -0.58)	-0.91% (-1.23 to -0.55)	28.32% (24.80 to 31.64)	23.10% (19.36 to 26.74)	0.17% (-0.62 to 1.09)	-1.65% (-2.89 to -0.55)	-0.71% (-1.42 to -0.02)
Canada	34.45% (33.55 to 35.34)	18.33% (16.81 to 19.84)	-2.12% (-2.44 to -1.82)	-2.24% (-2.85 to -1.65)	-2.18% (-2.51 to -1.88)	30.64% (29.62 to 31.66)	15.86% (13.40 to 18.58)	-2.68% (-3.10 to -2.24)	-1.86% (-3.09 to -0.67)	-2.28% (-2.87 to -1.70)
United States of America	28.34% (27.87 to 28.81)	19.88% (18.78 to 21.01)	-0.83% (-0.99 to -0.65)	-1.65% (-2.09 to -1.22)	-1.22% (-1.42 to -1.03)	24.90% (24.38 to 25.46)	15.26% (13.93 to 16.73)	-1.45% (-1.67 to -1.24)	-1.95% (-2.61 to -1.24)	-1.69% (-2.01 to -1.36)
Antigua and Barbuda	9.57% (8.47 to 10.78)	12.53% (11.14 to 14.07)	0.94% (0.12 to 1.81)	0.93% (0.07 to 1.71)	0.93% (0.38 to 1.46)	4.30% (3.28 to 5.66)	5.56% (4.29 to 6.97)	0.99% (-0.88 to 2.92)	0.78% (-0.96 to 2.62)	0.89% (-0.23 to 2.06)
Bahamas	11.52% (10.19 to 12.96)	11.45% (10.43 to 12.56)	-0.39% (-1.19 to 0.41)	0.38% (-0.38 to 1.18)	-0.02% (-0.50 to 0.50)	3.67% (2.79 to 4.80)	3.18% (2.51 to 4.04)	-0.44% (-2.36 to 1.38)	-0.52% (-2.36 to 1.71)	-0.48% (-1.67 to 0.83)
Barbados	16.53% (14.80 to 18.31)	13.94% (12.58 to 15.48)	-0.06% (-0.78 to 0.68)	-1.15% (-1.93 to -0.38)	-0.59% (-1.07 to -0.09)	4.86% (3.64 to 6.32)	4.61% (3.60 to 5.88)	0.09% (-1.80 to 1.87)	-0.45% (-2.48 to 1.61)	-0.17% (-1.44 to 1.11)
Belize	22.10% (20.01 to 24.11)	22.48% (20.40 to 24.60)	-0.21% (-0.85 to 0.45)	0.34% (-0.33 to 1.01)	0.06% (-0.41 to 0.50)	3.85% (3.00 to 4.98)	4.13% (3.18 to 5.24)	0.18% (-1.63 to 1.91)	0.32% (-1.59 to 2.10)	0.25% (-1.03 to 1.40)
Cuba	42.97% (40.08 to 45.82)	31.80% (29.45 to 34.38)	-0.54% (-1.00 to -0.08)	-1.57% (-2.12 to -1.03)	-1.04% (-1.37 to -0.69)	23.41% (19.33 to 28.03)	15.28% (12.66 to 17.88)	-0.14% (-1.31 to 1.10)	-2.89% (-4.23 to -1.53)	-1.47% (-2.33 to -0.56)
Dominica	15.58% (13.83 to 17.49)	15.10% (13.42 to 16.84)	-0.74% (-1.58 to 0.09)	0.57% (-0.27 to 1.36)	-0.11% (-0.64 to 0.44)	6.85% (5.24 to 8.76)	6.29% (4.82 to 8.04)	-0.44% (-2.24 to 1.36)	-0.15% (-1.89 to 1.68)	-0.30% (-1.50 to 0.84)
Dominican Republic	14.74% (13.34 to 16.10)	13.65% (12.57 to 14.94)	0.68% (0.07 to 1.34)	-1.27% (-1.89 to -0.62)	-0.26% (-0.68 to 0.20)	11.83% (9.82 to 13.99)	9.27% (7.70 to 10.92)	0.40% (-0.86 to 1.68)	-2.17% (-3.46 to -0.89)	-0.84% (-1.74 to 0.04)
Grenada	16.51% (14.81 to 18.46)	17.67% (15.84 to 19.60)	0.57% (-0.16 to 1.31)	-0.12% (-0.93 to 0.64)	0.23% (-0.28 to 0.73)	5.35% (4.04 to 6.94)	5.39% (4.16 to 6.85)	0.55% (-1.32 to 2.32)	-0.52% (-2.38 to 1.36)	0.03% (-1.25 to 1.26)
Guyana	23.48% (21.19 to 25.86)	24.92% (23.28 to 26.75)	0.11% (-0.50 to 0.75)	0.31% (-0.34 to 0.99)	0.21% (-0.23 to 0.65)	4.73% (3.60 to 6.09)	4.20% (3.32 to 5.19)	-0.36% (-2.11 to 1.31)	-0.44% (-2.28 to 1.28)	-0.40% (-1.58 to 0.70)
Haiti	18.71% (16.92 to 20.73)	10.12% (9.22 to 11.00)	-2.57% (-3.30 to -1.88)	-1.63% (-2.39 to -0.89)	-2.12% (-2.57 to -1.67)	5.23% (4.01 to 6.58)	2.65% (2.05 to 3.36)	-1.86% (-3.61 to 0.00)	-2.87% (-4.77 to -1.01)	-2.35% (-3.64 to -1.11)
Jamaica	27.43% (25.14 to 29.74)	20.14% (18.49 to 21.80)	-0.31% (-0.88 to 0.26)	-1.87% (-2.55 to -1.21)	-1.07% (-1.47 to -0.66)	8.79% (7.47 to 10.51)	6.82% (5.73 to 8.09)	0.07% (-1.15 to 1.27)	-1.89% (-3.34 to -0.42)	-0.88% (-1.77 to 0.01)
Saint Lucia	22.15% (19.86 to 24.30)	19.15% (17.41 to 21.06)	-0.15% (-0.81 to 0.53)	-0.87% (-1.64 to -0.07)	-0.50% (-0.95 to -0.03)	6.26% (4.85 to 8.06)	5.27% (4.17 to 6.49)	-0.28% (-1.95 to 1.40)	-0.91% (-2.81 to 1.08)	-0.58% (-1.72 to 0.58)
Saint Vincent and the Grenadines	21.38% (19.38 to 23.41)	20.28% (18.53 to 22.06)	-0.50% (-1.18 to 0.19)	0.16% (-0.61 to 0.91)	-0.18% (-0.59 to 0.25)	4.21% (3.25 to 5.36)	4.24% (3.22 to 5.53)	0.68% (-1.30 to 2.36)	-0.70% (-2.68 to 1.35)	0.02% (-1.18 to 1.19)
Suriname	41.54% (38.33 to 44.56)	34.16% (31.70 to 36.55)	-0.33% (-0.80 to 0.15)	-1.05% (-1.60 to -0.50)	-0.67% (-1.01 to -0.32)	12.04% (9.46 to 15.14)	8.85% (7.07 to 10.96)	-0.44% (-1.98 to 1.13)	-1.72% (-3.38 to 0.01)	-1.06% (-2.18 to 0.03)
Trinidad and Tobago	33.10% (30.44 to 35.87)	28.86% (26.63 to 31.16)	-0.96% (-1.53 to -0.37)	0.05% (-0.56 to 0.64)	-0.47% (-0.87 to -0.08)	9.00% (7.07 to 11.48)	8.17% (6.44 to 10.10)	-0.80% (-2.43 to 0.85)	0.17% (-1.57 to 1.84)	-0.33% (-1.43 to 0.81)
Bolivia (Plurinational State of)	19.78% (17.86 to 21.99)	18.23% (16.76 to 19.75)	-2.42% (-3.09 to -1.70)	2.01% (1.28 to 2.73)	-0.28% (-0.72 to 0.17)	7.48% (5.63 to 9.66)	7.31% (5.94 to 8.85)	-2.02% (-3.68 to -0.28)	2.04% (0.21 to 3.81)	-0.06% (-1.19 to 1.07)
Ecuador	26.98% (24.93 to 29.24)	24.68% (23.50 to 25.94)	-0.73% (-1.31 to -0.16)	0.15% (-0.36 to 0.65)	-0.31% (-0.65 to 0.03)	6.14% (4.87 to 7.53)	4.97% (4.14 to 5.98)	-0.53% (-2.01 to 0.89)	-0.94% (-2.47 to 0.50)	-0.73% (-1.69 to 0.25)

Peru	11.39% (10.02 to 12.84)	7.33% (6.56 to 8.20)	0.34% (-0.47 to 1.17)	-3.52% (-4.41 to -2.63)	-1.52% (-2.08 to -0.97)	5.34% (3.99 to 6.84)	4.02% (3.22 to 4.95)	0.27% (-1.35 to 2.01)	-2.30% (-4.09 to -0.47)	-0.97% (-2.19 to 0.30)
Colombia	31.82% (29.36 to 34.61)	14.73% (13.57 to 15.95)	-1.66% (-2.20 to -1.12)	-3.72% (-4.36 to -3.05)	-2.65% (-3.05 to -2.26)	14.75% (11.89 to 18.08)	9.71% (8.42 to 11.17)	-0.86% (-2.26 to 0.48)	-2.03% (-3.26 to -0.81)	-1.43% (-2.34 to -0.50)
Costa Rica	28.41% (26.08 to 30.92)	15.34% (14.10 to 16.61)	-1.83% (-2.40 to -1.20)	-2.44% (-3.11 to -1.80)	-2.12% (-2.54 to -1.70)	14.03% (11.47 to 16.98)	7.19% (5.93 to 8.67)	-1.17% (-2.56 to 0.17)	-3.52% (-4.91 to -2.15)	-2.30% (-3.22 to -1.34)
El Salvador	12.97% (11.53 to 14.59)	17.35% (15.82 to 18.94)	0.79% (-0.01 to 1.60)	1.23% (0.49 to 2.05)	1.01% (0.49 to 1.52)	2.75% (2.05 to 3.56)	3.70% (2.91 to 4.63)	1.04% (-0.72 to 2.84)	1.01% (-0.81 to 2.83)	1.03% (-0.18 to 2.17)
Guatemala	24.07% (21.91 to 26.38)	20.52% (19.05 to 22.03)	-0.43% (-1.05 to 0.21)	-0.68% (-1.32 to -0.06)	-0.55% (-0.98 to -0.15)	4.64% (3.60 to 5.84)	4.34% (3.43 to 5.41)	-0.27% (-1.98 to 1.39)	-0.18% (-1.91 to 1.52)	-0.23% (-1.34 to 0.90)
Honduras	30.00% (27.44 to 32.60)	23.98% (22.19 to 25.87)	-0.92% (-1.49 to -0.31)	-0.62% (-1.20 to -0.01)	-0.77% (-1.15 to -0.38)	6.02% (4.86 to 7.50)	5.25% (4.15 to 6.61)	-0.88% (-2.42 to 0.74)	-0.03% (-1.80 to 1.66)	-0.47% (-1.50 to 0.52)
Mexico	45.44% (43.54 to 47.19)	26.97% (25.66 to 28.23)	-2.53% (-2.85 to -2.20)	-1.02% (-1.42 to -0.61)	-1.80% (-2.02 to -1.58)	17.43% (15.54 to 19.44)	9.01% (7.79 to 10.28)	-2.61% (-3.46 to -1.70)	-1.93% (-3.06 to -0.89)	-2.28% (-2.92 to -1.66)
Nicaragua	28.12% (25.82 to 30.73)	21.52% (19.50 to 23.71)	-1.76% (-2.44 to -1.08)	-0.02% (-0.70 to 0.63)	-0.92% (-1.39 to -0.47)	6.63% (5.08 to 8.42)	5.46% (4.15 to 7.01)	-0.93% (-2.86 to 0.96)	-0.39% (-2.16 to 1.53)	-0.67% (-1.90 to 0.55)
Panama	20.42% (18.78 to 22.18)	12.42% (11.29 to 13.74)	-2.08% (-2.77 to -1.36)	-1.33% (-2.05 to -0.59)	-1.72% (-2.14 to -1.28)	7.23% (5.72 to 9.18)	4.57% (3.58 to 5.71)	-1.06% (-2.79 to 0.71)	-2.15% (-3.82 to -0.57)	-1.58% (-2.71 to -0.38)
Venezuela (Bolivarian Republic of)	35.35% (32.46 to 38.43)	22.84% (20.54 to 25.18)	-2.10% (-2.70 to -1.47)	-0.87% (-1.57 to -0.20)	-1.51% (-1.91 to -1.08)	17.50% (13.99 to 21.18)	11.74% (9.37 to 14.54)	-1.64% (-3.04 to -0.25)	-1.10% (-2.69 to 0.45)	-1.38% (-2.38 to -0.40)
Brazil	39.47% (37.52 to 41.49)	10.85% (10.14 to 11.56)	-3.23% (-3.58 to -2.87)	-5.76% (-6.32 to -5.20)	-4.45% (-4.74 to -4.16)	27.11% (25.75 to 28.49)	6.86% (6.02 to 7.73)	-3.37% (-3.79 to -2.93)	-6.21% (-7.24 to -5.24)	-4.74% (-5.22 to -4.29)
Paraguay	40.86% (38.20 to 43.61)	24.63% (22.67 to 26.66)	-1.55% (-2.04 to -1.07)	-1.96% (-2.55 to -1.42)	-1.75% (-2.14 to -1.40)	14.48% (11.99 to 17.17)	8.33% (6.79 to 10.07)	-1.80% (-3.13 to -0.49)	-2.03% (-3.41 to -0.63)	-1.91% (-2.86 to -0.98)
Algeria	35.27% (32.44 to 38.24)	32.75% (30.58 to 34.79)	-0.79% (-1.35 to -0.23)	0.32% (-0.29 to 0.90)	-0.26% (-0.61 to 0.08)	2.48% (1.87 to 3.21)	1.74% (1.34 to 2.24)	-1.33% (-3.16 to 0.43)	-1.11% (-3.15 to 0.69)	-1.22% (-2.49 to -0.02)
Bahrain	23.62% (22.30 to 25.05)	23.36% (21.41 to 25.54)	-1.15% (-1.70 to -0.57)	1.14% (0.53 to 1.80)	-0.04% (-0.40 to 0.33)	6.48% (5.44 to 7.59)	5.10% (4.13 to 6.33)	-2.05% (-3.47 to -0.51)	0.47% (-1.30 to 2.20)	-0.83% (-1.78 to 0.09)
Egypt	38.44% (35.88 to 41.17)	43.41% (42.20 to 44.67)	0.22% (-0.23 to 0.65)	0.64% (0.30 to 0.98)	0.42% (0.16 to 0.68)	1.05% (0.78 to 1.40)	1.06% (0.80 to 1.37)	1.63% (-0.36 to 3.65)	-1.71% (-3.88 to 0.56)	0.01% (-1.31 to 1.41)
Iran (Islamic Republic of)	24.41% (22.25 to 26.86)	24.91% (23.25 to 26.53)	0.06% (-0.54 to 0.67)	0.09% (-0.44 to 0.61)	0.07% (-0.32 to 0.47)	4.44% (3.43 to 5.74)	4.73% (3.84 to 5.75)	0.37% (-1.32 to 1.98)	0.07% (-1.61 to 1.64)	0.22% (-1.07 to 1.35)
Iraq	46.30% (43.55 to 49.09)	37.56% (35.39 to 39.58)	-1.29% (-1.65 to -0.86)	-0.12% (-0.55 to 0.31)	-0.72% (-1.00 to -0.42)	4.60% (3.61 to 5.88)	3.53% (2.81 to 4.38)	-0.80% (-2.47 to 0.79)	-1.03% (-2.82 to 0.72)	-0.91% (-2.06 to 0.22)
Jordan	47.64% (45.04 to 50.34)	53.01% (51.48 to 54.55)	-0.67% (-1.06 to -0.28)	1.48% (1.18 to 1.79)	0.37% (0.16 to 0.58)	10.83% (8.68 to 13.38)	11.83% (10.11 to 13.76)	-0.18% (-1.67 to 1.23)	0.85% (-0.44 to 2.15)	0.31% (-0.59 to 1.27)
Kuwait	39.31% (36.58 to 41.96)	33.69% (31.53 to 35.83)	-0.56% (-1.02 to -0.09)	-0.50% (-1.01 to 0.01)	-0.53% (-0.84 to -0.22)	5.24% (4.09 to 6.60)	4.72% (3.78 to 5.76)	-0.24% (-1.91 to 1.41)	-0.48% (-2.22 to 1.19)	-0.36% (-1.56 to 0.75)
Lebanon	36.92% (34.33 to 39.58)	46.11% (44.00 to 48.23)	0.99% (0.52 to 1.51)	0.53% (0.16 to 0.88)	0.77% (0.46 to 1.07)	18.07% (14.97 to 21.47)	25.98% (22.85 to 29.02)	2.05% (0.89 to 3.30)	0.42% (-0.53 to 1.29)	1.26% (0.51 to 2.03)
Libya	38.43% (35.72 to 40.99)	38.62% (36.28 to 41.02)	-0.64% (-1.08 to -0.22)	0.72% (0.20 to 1.24)	0.02% (-0.30 to 0.35)	1.52% (1.14 to 2.02)	1.46% (1.10 to 1.94)	-1.20% (-3.14 to 0.67)	1.01% (-0.95 to 3.06)	-0.13% (-1.44 to 1.12)
Morocco	33.64% (31.15 to 36.01)	22.46% (20.85 to 24.10)	-1.72% (-2.24 to -1.16)	-1.05% (-1.58 to -0.52)	-1.39% (-1.72 to -1.04)	1.58% (1.16 to 2.11)	1.06% (0.79 to 1.42)	-1.92% (-3.94 to 0.05)	-0.77% (-2.91 to 1.42)	-1.37% (-2.70 to 0.03)
Palestine	44.59% (42.81 to 46.36)	40.79% (38.59 to 43.18)	-0.14% (-0.45 to 0.17)	-0.48% (-0.90 to -0.07)	-0.31% (-0.55 to -0.07)	4.06% (3.30 to 4.94)	3.89% (3.07 to 4.84)	0.29% (-1.21 to 1.83)	-0.63% (-2.28 to 1.07)	-0.16% (-1.20 to 0.88)
Oman	20.95% (19.52 to 22.39)	15.78% (14.46 to 17.27)	-3.09% (-3.76 to -2.46)	1.29% (0.63 to 1.93)	-0.98% (-1.35 to -0.62)	2.15% (1.69 to 2.73)	1.88% (1.45 to 2.43)	-1.72% (-3.50 to 0.13)	0.90% (-0.94 to 2.80)	-0.45% (-1.61 to 0.72)
Qatar	22.86% (20.89 to 25.00)	21.86% (20.27 to 23.64)	-0.32% (-0.91 to 0.28)	0.03% (-0.69 to 0.74)	-0.15% (-0.58 to 0.28)	2.94% (2.04 to 4.07)	2.97% (2.24 to 3.94)	0.34% (-1.87 to 2.53)	-0.25% (-2.44 to 2.03)	0.05% (-1.37 to 1.50)
Saudi Arabia	16.26% (15.07 to 17.48)	22.57% (21.09 to 24.05)	2.22% (1.65 to 2.80)	-0.03% (-0.59 to 0.50)	1.13% (0.80 to 1.48)	1.52% (1.19 to 1.98)	2.17% (1.69 to 2.71)	2.25% (0.48 to 3.95)	0.14% (-1.83 to 2.10)	1.23% (0.01 to 2.38)
Syrian Arab Republic	50.25% (47.57 to 52.88)	41.94% (38.99 to 44.82)	-0.79% (-1.21 to -0.37)	-0.45% (-0.90 to 0.01)	-0.62% (-0.92 to -0.36)	8.20% (6.42 to 10.26)	6.20% (4.77 to 7.97)	-1.16% (-2.83 to 0.51)	-0.76% (-2.49 to 0.88)	-0.97% (-2.20 to 0.20)

Tunisia	51.13% (48.53 to 53.86)	45.41% (43.23 to 47.66)	-0.34% (-0.71 to 0.03)	-0.49% (-0.88 to -0.11)	-0.41% (-0.64 to -0.18)	3.42% (2.66 to 4.37)	2.70% (2.12 to 3.45)	-0.68% (-2.39 to 1.05)	-0.97% (-2.88 to 0.87)	-0.82% (-1.97 to 0.35)
Turkey	55.37% (52.70 to 58.06)	43.25% (41.60 to 44.88)	-0.95% (-1.29 to -0.61)	-0.75% (-1.04 to -0.44)	-0.85% (-1.05 to -0.65)	16.11% (14.17 to 18.35)	18.38% (16.56 to 20.30)	0.68% (-0.20 to 1.61)	0.22% (-0.67 to 1.07)	0.46% (-0.12 to 1.00)
United Arab Emirates	22.73% (20.65 to 25.16)	17.91% (16.54 to 19.33)	0.48% (-0.15 to 1.15)	-2.21% (-2.86 to -1.54)	-0.82% (-1.25 to -0.40)	3.75% (2.90 to 4.76)	3.79% (2.96 to 4.81)	1.61% (-0.08 to 3.36)	-1.65% (-3.43 to 0.15)	0.04% (-1.09 to 1.26)
Yemen	33.10% (30.71 to 35.53)	31.52% (29.45 to 33.64)	-0.62% (-1.09 to -0.12)	0.31% (-0.23 to 0.82)	-0.17% (-0.52 to 0.18)	8.66% (6.68 to 10.91)	8.90% (7.46 to 10.67)	-0.02% (-1.64 to 1.65)	0.24% (-1.28 to 1.71)	0.11% (-0.95 to 1.16)
Afghanistan	5.61% (4.90 to 6.41)	17.00% (15.69 to 18.40)	5.70% (4.84 to 6.54)	1.83% (1.03 to 2.66)	3.83% (3.27 to 4.36)	0.98% (0.73 to 1.28)	2.67% (2.10 to 3.39)	5.03% (3.11 to 6.97)	1.81% (-0.23 to 3.80)	3.48% (2.27 to 4.65)
Bangladesh	50.71% (48.59 to 52.78)	44.90% (43.01 to 46.98)	-0.72% (-1.01 to -0.42)	-0.09% (-0.43 to 0.25)	-0.42% (-0.63 to -0.22)	2.99% (2.38 to 3.73)	2.13% (1.68 to 2.66)	-1.69% (-3.29 to -0.14)	-0.61% (-2.43 to 1.08)	-1.17% (-2.23 to -0.15)
Bhutan	17.03% (15.28 to 18.79)	14.79% (13.43 to 16.15)	-1.23% (-1.93 to -0.54)	0.31% (-0.43 to 1.06)	-0.48% (-0.95 to -0.01)	5.13% (3.90 to 6.66)	4.80% (3.79 to 5.95)	-0.82% (-2.74 to 1.01)	0.43% (-1.53 to 2.25)	-0.22% (-1.42 to 0.95)
India	39.03% (38.05 to 39.97)	23.04% (21.85 to 24.21)	-1.58% (-1.81 to -1.33)	-2.08% (-2.51 to -1.65)	-1.82% (-2.01 to -1.62)	4.54% (3.87 to 5.33)	3.10% (2.59 to 3.74)	-0.47% (-1.69 to 0.67)	-2.23% (-3.77 to -0.61)	-1.32% (-2.16 to -0.48)
Nepal	49.85% (47.32 to 52.38)	31.38% (29.82 to 32.95)	-0.99% (-1.32 to -0.64)	-2.24% (-2.65 to -1.87)	-1.60% (-1.84 to -1.36)	29.76% (26.10 to 33.59)	13.64% (11.89 to 15.58)	-0.90% (-1.72 to 0.06)	-4.61% (-5.63 to -3.58)	-2.69% (-3.33 to -2.04)
Pakistan	40.67% (38.17 to 43.37)	24.71% (23.28 to 26.29)	-1.22% (-1.66 to -0.78)	-2.25% (-2.77 to -1.71)	-1.72% (-2.02 to -1.41)	6.47% (5.04 to 8.39)	3.77% (3.05 to 4.65)	-0.92% (-2.59 to 0.78)	-2.86% (-4.48 to -1.15)	-1.85% (-2.99 to -0.69)
Angola	21.58% (19.47 to 23.88)	18.68% (17.23 to 20.23)	-1.26% (-1.92 to -0.61)	0.32% (-0.44 to 1.06)	-0.50% (-0.91 to -0.06)	3.09% (2.29 to 4.06)	2.74% (2.14 to 3.51)	-1.11% (-2.98 to 0.81)	0.34% (-1.60 to 2.18)	-0.41% (-1.57 to 0.73)
Central African Republic	20.30% (18.09 to 22.65)	15.02% (13.33 to 16.79)	-1.85% (-2.64 to -1.11)	-0.17% (-0.99 to 0.68)	-1.04% (-1.54 to -0.56)	2.21% (1.66 to 2.88)	1.60% (1.22 to 2.12)	-1.87% (-3.84 to 0.04)	-0.29% (-2.32 to 1.80)	-1.11% (-2.40 to 0.15)
Congo	16.11% (14.37 to 17.72)	20.68% (19.03 to 22.55)	0.46% (-0.20 to 1.13)	1.30% (0.61 to 1.98)	0.86% (0.44 to 1.34)	1.54% (1.15 to 2.04)	1.81% (1.33 to 2.42)	1.82% (-0.36 to 3.95)	-0.79% (-3.01 to 1.52)	0.56% (-0.95 to 1.94)
Democratic Republic of the Congo	23.02% (20.89 to 25.46)	21.60% (19.97 to 23.37)	-1.21% (-1.84 to -0.56)	0.85% (0.10 to 1.56)	-0.22% (-0.65 to 0.19)	1.49% (1.10 to 1.96)	1.36% (1.01 to 1.79)	-1.25% (-3.27 to 0.67)	0.72% (-1.30 to 2.77)	-0.30% (-1.66 to 1.03)
Equatorial Guinea	27.02% (24.36 to 29.51)	27.48% (25.00 to 29.80)	-0.43% (-1.06 to 0.18)	0.58% (-0.15 to 1.25)	0.06% (-0.39 to 0.50)	2.47% (1.79 to 3.50)	2.50% (1.81 to 3.47)	-0.34% (-2.72 to 1.88)	0.46% (-1.95 to 3.00)	0.05% (-1.55 to 1.58)
Gabon	18.76% (16.92 to 20.89)	21.94% (20.28 to 23.74)	0.65% (0.02 to 1.34)	0.42% (-0.34 to 1.19)	0.54% (0.05 to 1.04)	2.88% (2.12 to 3.84)	3.55% (2.71 to 4.54)	0.86% (-1.10 to 2.75)	0.59% (-1.44 to 2.86)	0.73% (-0.65 to 2.11)
Burundi	25.56% (23.25 to 27.88)	16.26% (15.08 to 17.55)	-3.81% (-4.45 to -3.18)	0.85% (0.09 to 1.57)	-1.56% (-1.97 to -1.16)	6.32% (4.77 to 8.12)	4.23% (3.38 to 5.28)	-0.99% (-2.62 to 0.72)	-1.80% (-3.69 to 0.36)	-1.38% (-2.59 to -0.15)
Comoros	27.62% (25.25 to 30.13)	23.27% (21.54 to 25.07)	-0.46% (-1.06 to 0.10)	-0.73% (-1.40 to -0.12)	-0.59% (-0.99 to -0.20)	3.25% (2.47 to 4.25)	2.99% (2.31 to 3.76)	0.35% (-1.45 to 2.15)	-0.96% (-2.95 to 1.00)	-0.28% (-1.49 to 1.04)
Djibouti	36.48% (34.01 to 39.11)	42.22% (39.64 to 44.92)	0.21% (-0.28 to 0.70)	0.82% (0.38 to 1.29)	0.50% (0.19 to 0.84)	4.90% (3.84 to 6.07)	5.52% (4.36 to 6.90)	0.15% (-1.41 to 1.81)	0.70% (-0.90 to 2.36)	0.41% (-0.72 to 1.64)
Eritrea	16.05% (14.26 to 17.87)	12.49% (11.21 to 13.95)	-1.25% (-1.98 to -0.49)	-0.45% (-1.29 to 0.35)	-0.86% (-1.40 to -0.32)	0.90% (0.66 to 1.19)	0.70% (0.52 to 0.91)	-1.06% (-3.26 to 0.98)	-0.67% (-2.70 to 1.35)	-0.87% (-2.31 to 0.44)
Ethiopia	12.40% (11.02 to 13.72)	10.45% (9.60 to 11.37)	-2.13% (-2.89 to -1.38)	1.07% (0.32 to 1.85)	-0.59% (-1.07 to -0.09)	1.39% (1.02 to 1.83)	1.31% (1.01 to 1.69)	-1.66% (-3.66 to 0.08)	1.42% (-0.49 to 3.41)	-0.18% (-1.39 to 1.09)
Kenya	29.90% (27.66 to 32.39)	19.15% (17.83 to 20.41)	-1.46% (-1.97 to -0.94)	-1.61% (-2.17 to -1.04)	-1.53% (-1.89 to -1.17)	3.94% (3.06 to 4.99)	2.52% (2.00 to 3.20)	1.23% (-0.39 to 2.81)	-4.53% (-6.28 to -2.68)	-1.55% (-2.71 to -0.36)
Madagascar	40.07% (37.36 to 42.77)	24.94% (22.93 to 26.79)	-2.81% (-3.24 to -2.36)	-0.38% (-1.03 to 0.26)	-1.64% (-2.00 to -1.28)	4.36% (3.42 to 5.63)	2.57% (2.00 to 3.30)	-2.65% (-4.53 to -0.89)	-0.94% (-3.03 to 1.05)	-1.82% (-2.96 to -0.62)
Malawi	24.09% (22.27 to 26.12)	23.99% (22.59 to 25.41)	-0.17% (-0.72 to 0.38)	0.16% (-0.34 to 0.65)	-0.01% (-0.36 to 0.33)	3.75% (2.84 to 4.83)	3.11% (2.44 to 3.93)	1.16% (-0.61 to 2.91)	-2.57% (-4.35 to -0.85)	-0.64% (-1.86 to 0.56)
Mauritius	47.35% (44.90 to 49.80)	41.17% (39.18 to 43.25)	-1.38% (-1.75 to -1.02)	0.48% (0.08 to 0.87)	-0.48% (-0.73 to -0.25)	5.92% (4.72 to 7.48)	5.40% (4.36 to 6.60)	-0.96% (-2.58 to 0.55)	0.37% (-1.32 to 2.02)	-0.32% (-1.33 to 0.72)
Mozambique	25.02% (22.91 to 27.27)	23.59% (21.64 to 25.71)	-0.29% (-0.87 to 0.32)	-0.11% (-0.72 to 0.55)	-0.20% (-0.65 to 0.22)	6.51% (5.10 to 8.13)	5.94% (4.68 to 7.39)	0.82% (-0.71 to 2.35)	-1.52% (-3.21 to 0.19)	-0.31% (-1.44 to 0.85)
Rwanda	22.11% (20.10 to 24.22)	21.73% (20.41 to 23.23)	-0.05% (-0.57 to 0.51)	-0.07% (-0.71 to 0.57)	-0.06% (-0.45 to 0.36)	8.78% (7.01 to 10.87)	8.51% (7.00 to 10.14)	0.46% (-0.99 to 1.87)	-0.70% (-2.25 to 0.84)	-0.10% (-1.04 to 0.89)

Seychelles	44.62% (41.63 to 47.79)	39.69% (36.91 to 42.36)	-0.59% (-1.04 to -0.12)	-0.21% (-0.75 to 0.30)	-0.40% (-0.76 to -0.07)	7.58% (5.88 to 9.56)	7.01% (5.58 to 8.62)	0.33% (-1.38 to 1.94)	-0.89% (-2.53 to 0.71)	-0.26% (-1.43 to 0.91)
Somalia	24.38% (22.11 to 26.94)	20.50% (18.32 to 22.67)	-0.80% (-1.49 to -0.16)	-0.38% (-1.07 to 0.39)	-0.60% (-1.08 to -0.15)	3.48% (2.64 to 4.37)	2.82% (2.17 to 3.66)	-0.70% (-2.50 to 1.05)	-0.76% (-2.65 to 0.98)	-0.73% (-1.95 to 0.39)
United Republic of Tanzania	29.47% (27.14 to 32.15)	17.97% (16.76 to 19.17)	-0.91% (-1.45 to -0.32)	-2.56% (-3.13 to -1.98)	-1.70% (-2.08 to -1.32)	4.15% (3.22 to 5.34)	2.96% (2.35 to 3.68)	0.84% (-0.90 to 2.58)	-3.32% (-5.05 to -1.53)	-1.17% (-2.32 to -0.07)
Uganda	19.22% (17.54 to 21.06)	14.57% (13.60 to 15.58)	0.62% (0.06 to 1.24)	-2.64% (-3.21 to -2.09)	-0.95% (-1.35 to -0.56)	4.29% (3.32 to 5.38)	4.18% (3.43 to 5.02)	1.55% (0.06 to 2.98)	-1.81% (-3.54 to -0.09)	-0.08% (-1.05 to 0.94)
Zambia	25.23% (23.06 to 27.40)	26.30% (24.93 to 27.68)	0.38% (-0.18 to 0.93)	-0.11% (-0.56 to 0.37)	0.14% (-0.21 to 0.51)	6.54% (5.23 to 7.93)	5.82% (4.75 to 6.99)	2.10% (0.73 to 3.50)	-3.08% (-4.56 to -1.51)	-0.40% (-1.36 to 0.59)
Botswana	38.88% (36.23 to 41.69)	36.31% (34.44 to 38.18)	-1.12% (-1.57 to -0.68)	0.71% (0.24 to 1.21)	-0.23% (-0.54 to 0.06)	10.49% (8.25 to 12.96)	8.39% (7.02 to 9.77)	-0.78% (-2.24 to 0.71)	-0.74% (-2.14 to 0.72)	-0.76% (-1.65 to 0.24)
Lesotho	32.10% (29.50 to 34.75)	41.51% (39.59 to 43.50)	0.25% (-0.29 to 0.77)	1.58% (1.11 to 2.03)	0.89% (0.55 to 1.22)	1.81% (1.36 to 2.38)	1.85% (1.42 to 2.42)	0.85% (-1.01 to 2.79)	-0.74% (-2.64 to 1.18)	0.08% (-1.13 to 1.42)
Namibia	29.97% (27.77 to 32.11)	23.45% (21.88 to 25.12)	-0.82% (-1.35 to -0.29)	-0.88% (-1.39 to -0.36)	-0.85% (-1.20 to -0.49)	14.45% (12.06 to 17.21)	10.64% (8.83 to 12.71)	-0.13% (-1.35 to 1.21)	-2.05% (-3.30 to -0.77)	-1.05% (-1.97 to -0.18)
South Africa	48.87% (47.28 to 50.37)	35.41% (33.77 to 36.92)	-2.25% (-2.56 to -1.93)	0.11% (-0.28 to 0.46)	-1.11% (-1.30 to -0.92)	16.79% (15.00 to 18.79)	10.37% (8.75 to 12.24)	-1.78% (-2.79 to -0.79)	-1.55% (-2.79 to -0.32)	-1.67% (-2.40 to -0.98)
Eswatini	21.73% (20.06 to 23.42)	14.99% (13.62 to 16.46)	-1.67% (-2.24 to -1.11)	-0.87% (-1.56 to -0.17)	-1.28% (-1.70 to -0.84)	3.98% (3.12 to 4.84)	2.87% (2.28 to 3.67)	-0.93% (-2.56 to 0.67)	-1.34% (-2.99 to 0.47)	-1.13% (-2.22 to -0.01)
Zimbabwe	33.22% (30.67 to 35.60)	34.36% (32.63 to 36.04)	-0.62% (-1.10 to -0.10)	0.91% (0.46 to 1.33)	0.12% (-0.19 to 0.43)	3.86% (3.03 to 4.87)	3.47% (2.73 to 4.29)	0.83% (-0.87 to 2.42)	-1.65% (-3.36 to -0.01)	-0.37% (-1.53 to 0.74)
Benin	18.43% (16.62 to 20.25)	10.61% (9.77 to 11.52)	-1.06% (-1.73 to -0.40)	-2.81% (-3.58 to -2.10)	-1.90% (-2.36 to -1.46)	3.13% (2.44 to 4.04)	1.69% (1.28 to 2.15)	-0.31% (-2.09 to 1.51)	-4.07% (-5.84 to -2.27)	-2.13% (-3.43 to -0.93)
Burkina Faso	23.78% (21.47 to 26.05)	17.04% (15.71 to 18.45)	-0.84% (-1.52 to -0.20)	-1.48% (-2.16 to -0.82)	-1.15% (-1.58 to -0.70)	2.22% (1.65 to 2.91)	1.47% (1.10 to 1.93)	0.77% (-1.19 to 2.82)	-3.77% (-5.70 to -1.83)	-1.42% (-2.76 to -0.04)
Cameroon	22.44% (20.28 to 24.65)	14.78% (13.46 to 16.18)	-1.23% (-1.82 to -0.63)	-1.66% (-2.47 to -0.90)	-1.44% (-1.91 to -0.98)	2.02% (1.55 to 2.57)	1.51% (1.14 to 1.94)	-0.38% (-2.13 to 1.40)	-1.68% (-3.45 to 0.25)	-1.01% (-2.21 to 0.21)
Cabo Verde	17.28% (15.57 to 19.07)	9.39% (8.41 to 10.51)	-2.70% (-3.45 to -1.98)	-1.47% (-2.31 to -0.63)	-2.10% (-2.63 to -1.59)	4.71% (3.61 to 5.86)	3.00% (2.36 to 3.83)	-2.13% (-3.64 to -0.50)	-0.93% (-2.77 to 1.01)	-1.55% (-2.71 to -0.35)
Chad	23.01% (20.91 to 25.17)	15.99% (14.65 to 17.36)	-0.64% (-1.30 to -0.04)	-1.91% (-2.62 to -1.25)	-1.25% (-1.70 to -0.79)	4.35% (3.36 to 5.65)	2.83% (2.23 to 3.63)	0.47% (-1.18 to 2.23)	-3.55% (-5.32 to -1.70)	-1.47% (-2.68 to -0.27)
Côte d'Ivoire	19.73% (17.82 to 21.79)	21.82% (20.05 to 23.69)	0.97% (0.27 to 1.62)	-0.32% (-1.07 to 0.42)	0.35% (-0.11 to 0.82)	3.81% (2.92 to 4.78)	3.79% (2.89 to 4.83)	0.97% (-0.72 to 2.65)	-1.08% (-2.95 to 0.81)	-0.02% (-1.17 to 1.10)
Gambia	35.00% (32.44 to 37.81)	23.63% (21.93 to 25.32)	-1.65% (-2.15 to -1.13)	-1.04% (-1.70 to -0.35)	-1.35% (-1.72 to -1.00)	2.08% (1.60 to 2.67)	1.33% (1.02 to 1.72)	-1.80% (-3.60 to -0.04)	-1.27% (-3.14 to 0.75)	-1.54% (-2.77 to -0.33)
Ghana	11.33% (10.24 to 12.59)	10.46% (9.42 to 11.63)	-0.67% (-1.38 to 0.07)	0.14% (-0.67 to 0.92)	-0.28% (-0.80 to 0.24)	1.99% (1.50 to 2.61)	2.08% (1.58 to 2.72)	0.54% (-1.28 to 2.37)	-0.26% (-2.29 to 1.85)	0.15% (-1.07 to 1.54)
Guinea	32.08% (29.08 to 35.04)	29.18% (26.65 to 31.94)	-0.22% (-0.79 to 0.41)	-0.45% (-1.05 to 0.20)	-0.33% (-0.74 to 0.07)	2.19% (1.69 to 2.81)	2.03% (1.54 to 2.56)	-0.47% (-2.26 to 1.30)	-0.03% (-1.74 to 1.80)	-0.25% (-1.48 to 0.91)
Guinea-Bissau	7.30% (6.36 to 8.28)	8.46% (7.42 to 9.54)	-1.32% (-2.23 to -0.42)	2.46% (1.47 to 3.41)	0.51% (-0.08 to 1.12)	1.05% (0.80 to 1.35)	1.07% (0.81 to 1.39)	-1.28% (-3.19 to 0.58)	1.48% (-0.58 to 3.46)	0.06% (-1.19 to 1.32)
Liberia	15.77% (14.12 to 17.62)	13.72% (12.50 to 15.03)	-0.14% (-0.86 to 0.55)	-0.84% (-1.57 to -0.12)	-0.48% (-0.95 to 0.02)	2.58% (1.92 to 3.30)	2.57% (2.03 to 3.24)	1.00% (-0.81 to 2.81)	-1.08% (-2.98 to 0.74)	0.00% (-1.27 to 1.24)
Mali	17.74% (16.03 to 19.51)	22.46% (20.85 to 24.07)	1.57% (0.92 to 2.24)	0.01% (-0.57 to 0.61)	0.82% (0.40 to 1.26)	1.68% (1.27 to 2.24)	2.44% (1.83 to 3.22)	1.94% (-0.10 to 3.90)	0.57% (-1.51 to 2.56)	1.27% (-0.08 to 2.61)
Mauritania	37.46% (34.60 to 40.35)	29.34% (26.83 to 31.77)	-1.06% (-1.57 to -0.51)	-0.62% (-1.24 to 0.05)	-0.84% (-1.21 to -0.43)	9.37% (7.28 to 11.85)	8.08% (6.28 to 10.15)	0.11% (-1.56 to 1.72)	-1.17% (-2.85 to 0.60)	-0.51% (-1.72 to 0.65)
Niger	12.69% (11.21 to 14.30)	14.15% (12.83 to 15.58)	1.38% (0.63 to 2.16)	-0.70% (-1.51 to 0.13)	0.38% (-0.18 to 0.93)	1.41% (1.06 to 1.92)	1.63% (1.23 to 2.16)	2.52% (0.53 to 4.45)	-1.65% (-3.56 to 0.28)	0.51% (-0.85 to 1.81)
Nigeria	12.12% (11.10 to 13.20)	7.43% (6.69 to 8.21)	-1.50% (-2.16 to -0.82)	-1.89% (-2.68 to -1.09)	-1.69% (-2.17 to -1.23)	2.51% (2.01 to 3.12)	1.16% (0.86 to 1.54)	-2.40% (-4.17 to -0.42)	-2.99% (-5.13 to -0.92)	-2.69% (-3.91 to -1.39)
Sao Tome and Principe	6.30% (5.57 to 7.12)	7.80% (6.95 to 8.69)	1.76% (0.95 to 2.57)	-0.36% (-1.18 to 0.45)	0.74% (0.18 to 1.24)	1.51% (1.13 to 2.02)	1.70% (1.29 to 2.22)	1.79% (0.00 to 3.65)	-1.05% (-3.18 to 1.02)	0.42% (-0.94 to 1.84)

Senegal	29.89% (26.95 to 32.74)	14.21% (13.18 to 15.31)	-1.65% (-2.24 to -1.03)	-3.54% (-4.17 to -2.91)	-2.56% (-2.95 to -2.12)	2.53% (1.89 to 3.33)	1.46% (1.11 to 1.90)	-0.16% (-1.92 to 1.63)	-3.75% (-5.76 to -1.75)	-1.89% (-3.19 to -0.67)
Sierra Leone	35.22% (32.58 to 38.02)	30.54% (28.68 to 32.56)	-0.52% (-0.99 to -0.02)	-0.46% (-1.01 to 0.11)	-0.49% (-0.83 to -0.11)	10.78% (8.49 to 13.48)	7.84% (6.34 to 9.61)	-0.40% (-1.96 to 1.01)	-1.83% (-3.56 to -0.12)	-1.09% (-2.18 to 0.01)
Togo	24.54% (22.37 to 26.72)	14.02% (12.83 to 15.20)	-2.04% (-2.60 to -1.40)	-1.81% (-2.57 to -1.11)	-1.93% (-2.37 to -1.51)	3.67% (2.77 to 4.78)	2.26% (1.73 to 2.92)	-1.16% (-3.07 to 0.68)	-2.22% (-4.09 to -0.24)	-1.67% (-2.95 to -0.44)
American Samoa	43.98% (40.70 to 47.00)	42.10% (39.16 to 45.17)	-0.17% (-0.66 to 0.31)	-0.12% (-0.62 to 0.36)	-0.15% (-0.47 to 0.18)	23.63% (19.06 to 28.30)	22.40% (18.35 to 27.25)	0.05% (-1.22 to 1.37)	-0.43% (-1.68 to 0.85)	-0.18% (-1.12 to 0.78)
Bermuda	18.28% (16.21 to 20.36)	18.08% (16.47 to 19.90)	0.31% (-0.36 to 1.05)	-0.40% (-1.14 to 0.33)	-0.04% (-0.50 to 0.47)	8.68% (6.53 to 11.01)	8.32% (6.80 to 10.17)	0.24% (-1.30 to 1.91)	-0.54% (-2.43 to 1.44)	-0.13% (-1.17 to 0.99)
Cook Islands	40.72% (37.66 to 43.76)	36.67% (34.38 to 39.12)	-0.22% (-0.72 to 0.26)	-0.51% (-1.10 to 0.06)	-0.36% (-0.71 to -0.01)	24.60% (20.01 to 29.08)	24.20% (20.46 to 28.58)	0.15% (-1.15 to 1.42)	-0.27% (-1.60 to 1.01)	-0.05% (-0.85 to 0.79)
Greenland	49.12% (45.96 to 52.29)	44.06% (40.91 to 47.25)	-0.03% (-0.43 to 0.37)	-0.75% (-1.24 to -0.28)	-0.38% (-0.69 to -0.07)	44.86% (39.40 to 50.48)	42.31% (36.49 to 48.43)	0.41% (-0.47 to 1.29)	-0.86% (-1.82 to 0.05)	-0.20% (-0.81 to 0.44)
Guam	35.52% (32.71 to 38.22)	30.29% (28.56 to 32.06)	-0.24% (-0.74 to 0.30)	-0.88% (-1.43 to -0.37)	-0.55% (-0.87 to -0.22)	22.04% (18.38 to 26.05)	20.05% (17.81 to 22.28)	-0.03% (-1.19 to 1.23)	-0.63% (-1.58 to 0.33)	-0.32% (-1.06 to 0.40)
Monaco	38.69% (35.71 to 41.69)	29.03% (26.36 to 31.69)	-1.10% (-1.68 to -0.55)	-0.87% (-1.47 to -0.20)	-0.99% (-1.38 to -0.59)	29.51% (24.63 to 34.93)	23.59% (18.90 to 28.58)	-0.86% (-2.23 to 0.39)	-0.69% (-2.14 to 0.72)	-0.78% (-1.69 to 0.08)
Nauru	45.18% (42.33 to 48.02)	43.29% (40.86 to 45.79)	0.06% (-0.36 to 0.53)	-0.37% (-0.81 to 0.07)	-0.15% (-0.43 to 0.15)	43.62% (38.33 to 49.13)	40.32% (36.32 to 44.64)	0.60% (-0.22 to 1.46)	-1.20% (-1.94 to -0.45)	-0.27% (-0.86 to 0.32)
Niue	29.26% (26.75 to 31.98)	27.00% (24.96 to 29.05)	-0.41% (-0.98 to 0.16)	-0.14% (-0.82 to 0.53)	-0.28% (-0.67 to 0.13)	16.23% (12.76 to 20.21)	15.80% (13.05 to 18.80)	-0.13% (-1.53 to 1.29)	-0.03% (-1.68 to 1.67)	-0.08% (-1.05 to 0.96)
Northern Mariana Islands	47.20% (44.11 to 50.43)	40.99% (37.98 to 43.93)	-0.36% (-0.82 to 0.11)	-0.62% (-1.15 to -0.10)	-0.49% (-0.82 to -0.16)	22.10% (17.63 to 26.59)	18.02% (14.26 to 22.13)	-0.30% (-1.75 to 1.20)	-1.15% (-2.73 to 0.41)	-0.71% (-1.62 to 0.29)
Palau	34.83% (32.13 to 37.68)	32.44% (30.56 to 34.31)	0.08% (-0.51 to 0.59)	-0.59% (-1.15 to -0.02)	-0.24% (-0.58 to 0.11)	13.08% (10.26 to 16.62)	12.64% (10.93 to 14.49)	0.18% (-1.34 to 1.74)	-0.40% (-1.81 to 1.09)	-0.10% (-1.01 to 0.77)
Puerto Rico	19.58% (18.04 to 21.22)	15.97% (14.86 to 17.18)	-0.53% (-1.11 to 0.06)	-0.89% (-1.48 to -0.30)	-0.70% (-1.05 to -0.32)	10.07% (8.51 to 11.78)	8.57% (7.42 to 9.87)	-0.53% (-1.70 to 0.60)	-0.57% (-1.68 to 0.58)	-0.55% (-1.32 to 0.22)
Saint Kitts and Nevis	10.34% (9.13 to 11.68)	10.46% (9.30 to 11.78)	1.20% (0.37 to 1.99)	-1.20% (-2.04 to -0.34)	0.04% (-0.56 to 0.63)	2.92% (2.20 to 3.70)	2.87% (2.16 to 3.75)	0.63% (-1.14 to 2.41)	-0.80% (-2.63 to 1.02)	-0.06% (-1.35 to 1.19)
San Marino	36.39% (33.41 to 39.41)	21.61% (19.74 to 23.43)	-2.06% (-2.58 to -1.45)	-1.52% (-2.27 to -0.78)	-1.80% (-2.21 to -1.40)	28.53% (23.33 to 34.24)	18.10% (15.20 to 21.51)	-1.58% (-2.80 to -0.20)	-1.55% (-2.94 to 0.03)	-1.57% (-2.44 to -0.70)
Tokelau	41.82% (38.85 to 45.15)	39.55% (36.41 to 42.55)	-0.07% (-0.57 to 0.43)	-0.32% (-0.89 to 0.19)	-0.19% (-0.54 to 0.17)	16.79% (13.20 to 20.69)	16.62% (12.99 to 20.60)	0.15% (-1.40 to 1.60)	-0.23% (-1.87 to 1.39)	-0.04% (-1.04 to 0.94)
Tuvalu	43.89% (40.80 to 46.90)	45.37% (42.64 to 48.03)	0.08% (-0.38 to 0.51)	0.16% (-0.31 to 0.68)	0.11% (-0.16 to 0.42)	19.11% (14.96 to 23.55)	20.70% (17.33 to 24.40)	0.24% (-1.15 to 1.70)	0.33% (-1.24 to 1.91)	0.29% (-0.67 to 1.24)
United States Virgin Islands	10.84% (9.67 to 12.12)	8.28% (7.38 to 9.22)	-0.28% (-1.06 to 0.51)	-1.63% (-2.48 to -0.84)	-0.93% (-1.45 to -0.36)	6.67% (5.31 to 8.35)	4.98% (3.98 to 6.18)	-0.32% (-1.93 to 1.38)	-1.74% (-3.35 to -0.10)	-1.00% (-2.14 to 0.10)
South Sudan	25.05% (22.86 to 27.49)	20.58% (18.54 to 22.83)	-0.89% (-1.56 to -0.23)	-0.45% (-1.17 to 0.27)	-0.68% (-1.14 to -0.25)	3.37% (2.57 to 4.30)	2.71% (2.05 to 3.43)	-0.82% (-2.55 to 0.91)	-0.67% (-2.49 to 1.02)	-0.75% (-1.90 to 0.31)
Sudan	22.33% (20.27 to 24.65)	19.54% (18.00 to 21.13)	0.14% (-0.48 to 0.86)	-1.11% (-1.76 to -0.41)	-0.46% (-0.90 to -0.02)	2.33% (1.77 to 3.02)	1.95% (1.46 to 2.54)	0.52% (-1.17 to 2.26)	-1.86% (-3.77 to 0.24)	-0.63% (-1.91 to 0.73)

Supplementary Table 3. Prevalence and number of users of current chewing tobacco and smoking use among people ages 15-19 in 1990 and 2019. Locations ordered by GBD super region and region.

		Males				Females			
		Prevalence		Number of Users		Prevalence		Number of Users	
Location	Year	Chewing	Smoking	Chewing	Smoking	Chewing	Smoking	Chewing	Smoking
China	1990	0.8% (0.4-1.4)	17.5% (14.8-20.5)	504,536 (231,388-913,131)	11,422,772 (9,635,950-13,368,609)	0.4% (0.1-1.0)	0.8% (0.4-1.7)	255,463 (90,017-612,689)	519,492 (220,283-1,055,524)
China	2019	0.9% (0.4-1.7)	15.0% (11.8-18.9)	365,150 (161,316-664,312)	6,030,743 (4,732,009-7,592,313)	0.4% (0.2-1.0)	2.2% (1.1-4.0)	156,564 (57,538-364,957)	765,957 (399,293-1,385,402)
Democratic People's Republic of Korea	1990	0.2% (0.1-0.4)	14.4% (9.4-20.1)	1,731 (807-3,387)	118,878 (77,390-166,045)	0.2% (0.1-0.5)	1.3% (0.5-2.7)	2,229 (843-4,735)	12,855 (4,679-27,469)
Democratic People's Republic of Korea	2019	0.2% (0.1-0.4)	14.1% (10.4-18.8)	1,860 (841-3,530)	129,447 (95,654-172,191)	0.2% (0.1-0.5)	1.7% (0.6-3.4)	2,005 (739-4,673)	14,749 (5,540-29,960)
Taiwan (Province of China)	1990	0.7% (0.3-1.4)	16.5% (11.0-23.1)	6,820 (3,172-12,839)	151,612 (101,031-213,020)	0.5% (0.2-1.0)	0.6% (0.2-1.4)	3,960 (1,501-8,635)	5,626 (2,023-12,454)
Taiwan (Province of China)	2019	0.8% (0.4-1.5)	12.6% (9.1-16.9)	4,976 (2,319-9,974)	82,051 (59,390-110,000)	0.5% (0.2-1.0)	0.5% (0.2-1.1)	2,679 (996-5,984)	2,744 (1,023-6,285)
Cambodia	1990	0.8% (0.4-1.6)	5.5% (3.6-8.0)	4,170 (1,855-7,737)	27,283 (17,793-39,532)	0.7% (0.3-1.5)	0.9% (0.3-1.9)	3,512 (1,309-7,755)	4,615 (1,701-9,604)
Cambodia	2019	0.9% (0.4-1.7)	6.1% (4.3-8.4)	7,116 (3,217-13,119)	46,562 (32,320-64,081)	0.8% (0.3-1.8)	1.3% (0.5-2.6)	5,758 (2,128-12,696)	9,295 (3,845-18,637)
Indonesia	1990	0.3% (0.1-0.5)	30.0% (25.2-35.5)	27,209 (11,966-51,442)	2,951,012 (2,480,200-3,496,467)	0.3% (0.1-0.6)	1.3% (0.6-2.7)	27,439 (10,404-61,421)	133,231 (55,019-271,000)
Indonesia	2019	0.4% (0.2-0.8)	26.9% (22.8-31.4)	48,945 (23,169-94,676)	3,134,963 (2,660,063-3,663,850)	0.4% (0.1-0.8)	2.4% (1.2-4.3)	41,024 (15,678-89,173)	269,154 (132,569-483,349)
Lao People's Democratic Republic	1990	1.1% (0.5-1.9)	19.5% (13.7-26.4)	2,180 (1,027-4,006)	40,212 (28,235-54,511)	1.6% (0.6-3.6)	2.9% (1.1-6.5)	3,626 (1,335-7,958)	6,337 (2,398-14,381)
Lao People's Democratic Republic	2019	1.1% (0.5-2.0)	21.9% (18.1-26.3)	3,917 (1,865-7,220)	78,271 (64,584-93,863)	1.8% (0.7-3.6)	4.5% (2.4-7.7)	6,155 (2,416-12,531)	15,903 (8,344-26,893)
Malaysia	1990	2.1% (1.0-4.0)	37.4% (28.5-46.7)	18,760 (8,862-35,987)	332,793 (253,729-414,710)	1.3% (0.5-2.9)	4.2% (1.7-8.4)	11,585 (4,000-25,729)	36,541 (15,074-73,342)
Malaysia	2019	2.5% (1.2-4.6)	20.6% (16.5-24.6)	35,090 (16,633-64,718)	287,887 (231,027-344,309)	1.4% (0.6-3.0)	3.7% (2.0-6.4)	19,041 (7,772-39,237)	49,523 (26,763-85,456)
Maldives	1990	1.8% (0.8-3.3)	15.9% (11.0-22.6)	205 (93-388)	1,846 (1,275-2,631)	0.7% (0.2-1.5)	7.4% (3.1-14.9)	81 (29-178)	861 (361-1,737)
Maldives	2019	1.8% (0.8-3.3)	20.6% (16.2-25.6)	297 (134-538)	3,334 (2,625-4,143)	0.7% (0.3-1.5)	8.3% (4.6-13.9)	94 (35-208)	1,165 (644-1,945)
Myanmar	1990	15.6% (8.2-25.7)	35.1% (26.4-44.8)	330,339 (174,440-544,592)	744,686 (560,984-951,121)	3.4% (1.3-7.3)	3.6% (1.4-7.3)	73,239 (28,504-155,390)	76,818 (29,590-154,894)
Myanmar	2019	14.0% (7.7-22.5)	26.6% (22.1-31.4)	347,457 (191,135-557,991)	660,036 (548,533-778,505)	2.8% (1.1-5.8)	2.4% (1.1-4.4)	68,372 (28,083-145,089)	58,966 (27,486-108,901)
Philippines	1990	1.7% (0.8-3.1)	38.3% (30.2-47.1)	59,316 (27,616-107,471)	1,317,571 (1,036,905-1,617,066)	0.6% (0.2-1.2)	8.1% (3.9-14.7)	20,289 (7,647-42,300)	276,655 (132,980-502,003)
Philippines	2019	1.7% (0.8-3.1)	22.8% (19.0-27.0)	95,532 (43,669-172,184)	1,255,393 (1,043,112-1,483,863)	0.6% (0.2-1.4)	7.7% (4.8-11.7)	30,421 (11,225-72,350)	403,930 (253,502-614,633)
Sri Lanka	1990	7.2% (3.4-13.0)	10.6% (7.1-14.5)	61,979 (29,040-111,817)	91,339 (60,797-124,814)	1.2% (0.4-2.6)	1.7% (0.7-3.5)	9,750 (3,525-21,943)	13,991 (5,799-29,821)
Sri Lanka	2019	6.7% (3.4-11.6)	6.1% (4.5-8.2)	58,799 (29,405-101,182)	53,686 (38,988-71,213)	1.0% (0.4-2.3)	0.8% (0.3-1.7)	8,888 (3,415-19,515)	7,077 (2,772-14,905)

Thailand	1990	0.8% (0.4-1.5)	29.1% (23.1-35.5)	24,159 (11,145-45,227)	854,879 (677,525-1,040,875)	0.5% (0.2-1.0)	2.9% (1.4-5.6)	13,448 (5,009-29,282)	84,981 (40,658-164,280)
Thailand	2019	1.1% (0.5-2.0)	24.5% (20.6-28.8)	23,373 (11,427-43,384)	535,754 (449,788-628,571)	0.5% (0.2-1.1)	3.2% (1.6-5.7)	10,419 (3,755-22,447)	67,406 (34,403-121,054)
Timor-Leste	1990	1.3% (0.6-2.5)	50.9% (40.5-61.8)	509 (233-999)	20,039 (15,944-24,298)	2.1% (0.8-4.4)	2.8% (1.1-6.2)	713 (274-1,518)	970 (371-2,156)
Timor-Leste	2019	1.4% (0.7-2.7)	38.2% (32.4-44.6)	1,169 (560-2,183)	31,413 (26,640-36,593)	2.2% (0.9-4.8)	5.3% (2.9-8.8)	1,735 (672-3,735)	4,154 (2,239-6,885)
Viet Nam	1990	0.4% (0.2-0.8)	17.2% (12.7-22.6)	14,021 (6,517-27,022)	611,338 (449,654-801,778)	0.3% (0.1-0.7)	0.9% (0.3-2.0)	11,408 (4,034-25,138)	31,944 (11,616-70,588)
Viet Nam	2019	0.4% (0.2-0.8)	12.2% (9.1-15.7)	13,805 (6,354-26,297)	423,276 (317,403-543,571)	0.3% (0.1-0.7)	1.0% (0.4-2.1)	10,161 (3,866-22,592)	31,807 (12,322-69,911)
Fiji	1990	4.2% (2.0-7.9)	25.9% (19.7-32.7)	1,651 (782-3,100)	10,214 (7,752-12,886)	2.3% (0.8-5.0)	14.7% (7.5-24.9)	872 (315-1,894)	5,555 (2,837-9,455)
Fiji	2019	4.0% (1.9-7.5)	24.5% (20.0-29.6)	1,565 (752-2,955)	9,598 (7,821-11,571)	2.2% (0.8-4.7)	11.5% (7.4-17.2)	814 (307-1,748)	4,283 (2,756-6,406)
Kiribati	1990	11.0% (5.4-20.1)	40.2% (31.3-49.6)	397 (196-725)	1,451 (1,132-1,792)	3.5% (1.2-7.5)	18.2% (9.0-31.3)	124 (43-262)	637 (315-1,098)
Kiribati	2019	11.7% (7.3-17.8)	42.8% (36.1-49.9)	646 (402-983)	2,363 (1,990-2,753)	3.5% (1.4-7.4)	26.3% (17.7-36.5)	196 (78-411)	1,459 (986-2,029)
Marshall Islands	1990	21.0% (11.7-32.4)	34.3% (26.4-43.2)	521 (291-804)	852 (657-1,074)	9.2% (3.9-18.2)	8.0% (3.4-16.9)	218 (92-433)	190 (80-401)
Marshall Islands	2019	32.5% (22.8-42.7)	36.8% (31.1-42.8)	905 (635-1,190)	1,023 (865-1,192)	17.0% (11.0-24.7)	14.9% (9.7-21.2)	461 (297-667)	402 (262-572)
Micronesia (Federated States of)	1990	24.4% (12.8-40.3)	53.8% (43.3-64.2)	1,432 (749-2,366)	3,161 (2,545-3,769)	18.3% (7.6-33.9)	30.7% (15.1-49.9)	1,042 (434-1,932)	1,747 (859-2,841)
Micronesia (Federated States of)	2019	28.9% (19.5-39.8)	59.0% (51.4-65.9)	1,659 (1,120-2,281)	3,385 (2,948-3,782)	22.5% (13.7-33.8)	37.6% (26.8-49.6)	1,218 (739-1,823)	2,032 (1,448-2,677)
Papua New Guinea	1990	18.8% (9.4-30.4)	50.5% (40.0-60.2)	42,195 (21,137-67,989)	113,213 (89,552-134,855)	13.6% (6.0-26.8)	27.0% (13.6-45.5)	28,137 (12,330-55,366)	55,868 (28,129-93,945)
Papua New Guinea	2019	18.3% (11.1-27.2)	44.1% (37.4-50.9)	92,544 (55,920-137,507)	222,662 (189,026-257,044)	13.2% (7.4-20.6)	26.5% (19.0-34.7)	59,586 (33,525-92,580)	119,322 (85,708-155,954)
Samoa	1990	2.9% (1.4-5.5)	18.7% (12.9-25.7)	322 (153-601)	2,047 (1,409-2,810)	1.6% (0.6-3.6)	9.7% (4.1-19.0)	152 (53-334)	908 (382-1,777)
Samoa	2019	3.0% (1.4-5.3)	21.2% (17.4-25.5)	368 (179-653)	2,616 (2,152-3,147)	1.7% (0.6-3.8)	7.7% (4.5-12.1)	191 (70-432)	883 (515-1,380)
Solomon Islands	1990	6.6% (3.1-11.6)	38.8% (28.9-48.3)	1,309 (627-2,312)	7,741 (5,776-9,655)	5.6% (2.2-11.3)	19.4% (8.7-34.1)	1,066 (409-2,144)	3,679 (1,647-6,469)
Solomon Islands	2019	6.5% (3.4-11.1)	35.8% (30.6-41.7)	2,262 (1,193-3,873)	12,525 (10,686-14,587)	5.9% (2.8-11.1)	15.1% (10.3-21.3)	1,922 (917-3,621)	4,908 (3,363-6,930)
Tonga	1990	8.8% (4.3-15.7)	25.8% (17.8-35.1)	530 (257-939)	1,545 (1,066-2,104)	5.0% (1.9-10.7)	11.3% (4.9-22.5)	278 (106-593)	622 (269-1,241)
Tonga	2019	9.3% (4.6-16.5)	24.6% (19.5-30.5)	504 (247-894)	1,332 (1,058-1,653)	5.1% (1.9-11.5)	11.5% (6.5-18.2)	263 (99-590)	586 (333-931)
Vanuatu	1990	6.5% (3.1-11.8)	38.8% (29.2-49.7)	507 (241-920)	3,020 (2,278-3,869)	4.8% (1.8-9.8)	15.1% (7.2-27.2)	360 (133-744)	1,140 (547-2,057)
Vanuatu	2019	6.4% (3.0-11.6)	24.0% (19.3-29.1)	922 (433-1,670)	3,455 (2,782-4,191)	4.7% (1.8-9.4)	11.7% (7.1-17.4)	663 (254-1,326)	1,651 (1,000-2,464)
Armenia	1990	0.4% (0.2-0.8)	20.4% (14.8-27.3)	631 (285-1,154)	30,206 (21,936-40,315)	0.4% (0.1-0.8)	1.0% (0.4-2.3)	483 (184-1,046)	1,392 (557-3,197)
Armenia	2019	0.4% (0.2-0.8)	16.6% (12.5-21.1)	372 (168-677)	14,647 (11,087-18,687)	0.3% (0.1-0.7)	1.3% (0.5-2.6)	244 (89-550)	1,010 (427-2,008)
Azerbaijan	1990	0.5% (0.2-0.9)	13.2% (9.0-18.7)	1,712 (772-3,310)	48,858 (33,363-68,941)	0.3% (0.1-0.7)	1.1% (0.4-2.3)	1,061 (392-2,306)	3,673 (1,325-7,855)

Azerbaijan	2019	0.5% (0.3-1.0)	16.9% (13.5-20.6)	1,875 (903-3,474)	60,017 (47,850-73,203)	0.3% (0.1-0.8)	1.5% (0.7-3.2)	1,080 (398-2,367)	4,789 (2,061-10,101)
Georgia	1990	0.8% (0.4-1.4)	25.6% (18.5-34.0)	1,698 (793-3,172)	56,799 (40,970-75,372)	0.4% (0.2-0.9)	3.7% (1.5-7.1)	905 (349-1,948)	7,611 (3,186-14,647)
Georgia	2019	0.9% (0.4-1.6)	24.2% (20.2-28.6)	894 (425-1,708)	25,442 (21,249-30,029)	0.5% (0.2-1.1)	7.0% (4.4-10.6)	428 (160-1,012)	6,399 (4,013-9,727)
Kazakhstan	1990	0.9% (0.4-1.7)	22.2% (17.2-28.1)	6,602 (2,976-12,289)	164,565 (127,335-208,484)	0.4% (0.1-0.9)	7.6% (3.6-13.9)	2,607 (923-6,066)	53,478 (25,580-98,073)
Kazakhstan	2019	0.8% (0.4-1.6)	11.1% (8.4-14.5)	5,004 (2,425-9,758)	66,660 (50,863-87,408)	0.3% (0.1-0.7)	5.5% (3.0-9.1)	1,795 (634-4,012)	32,258 (17,788-53,215)
Kyrgyzstan	1990	3.9% (1.9-7.1)	13.9% (10.8-17.6)	8,660 (4,237-15,630)	30,596 (23,584-38,539)	0.4% (0.2-0.9)	2.0% (0.8-4.4)	898 (346-1,953)	4,292 (1,728-9,307)
Kyrgyzstan	2019	4.1% (2.0-7.3)	12.9% (9.8-17.0)	10,999 (5,308-19,578)	34,558 (26,222-45,262)	0.4% (0.2-0.9)	3.0% (1.4-5.6)	1,082 (418-2,431)	7,735 (3,560-14,413)
Mongolia	1990	4.7% (2.3-8.7)	14.6% (10.1-19.9)	5,650 (2,717-10,365)	17,441 (11,986-23,660)	1.9% (0.7-4.1)	3.2% (1.3-6.6)	2,150 (767-4,786)	3,743 (1,531-7,670)
Mongolia	2019	4.8% (2.3-8.8)	19.1% (15.0-23.9)	5,319 (2,526-9,776)	21,338 (16,697-26,680)	1.9% (0.6-3.9)	4.5% (2.4-8.1)	2,027 (677-4,170)	4,845 (2,553-8,645)
Tajikistan	1990	1.8% (0.8-3.2)	9.4% (6.2-13.7)	4,856 (2,266-8,913)	25,855 (17,183-37,712)	0.6% (0.2-1.3)	1.1% (0.4-2.3)	1,665 (612-3,667)	2,969 (1,126-6,457)
Tajikistan	2019	2.1% (1.0-3.9)	4.9% (3.3-6.9)	9,377 (4,496-17,225)	21,712 (14,806-30,682)	0.6% (0.2-1.3)	0.8% (0.3-1.6)	2,572 (948-5,389)	3,304 (1,276-6,861)
Turkmenistan	1990	0.4% (0.2-0.8)	18.1% (12.3-24.9)	832 (395-1,491)	35,566 (24,163-49,013)	0.2% (0.1-0.5)	1.3% (0.5-2.8)	401 (143-895)	2,398 (928-5,270)
Turkmenistan	2019	0.4% (0.2-0.8)	10.2% (6.9-14.2)	858 (391-1,697)	21,409 (14,462-29,795)	0.2% (0.1-0.4)	0.8% (0.3-1.6)	388 (141-852)	1,473 (563-3,120)
Uzbekistan	1990	9.9% (5.0-18.0)	1.2% (0.7-1.8)	105,099 (52,589-190,434)	12,338 (7,527-18,870)	0.4% (0.1-0.8)	0.3% (0.1-0.7)	3,956 (1,351-8,786)	3,485 (1,225-7,549)
Uzbekistan	2019	12.7% (6.9-21.3)	1.6% (1.0-2.5)	184,995 (100,912-310,674)	24,044 (14,985-36,351)	0.4% (0.1-0.8)	0.5% (0.2-1.1)	5,110 (1,796-11,607)	7,020 (2,681-15,241)
Albania	1990	2.3% (1.1-4.3)	17.7% (12.0-23.7)	3,850 (1,830-7,291)	29,972 (20,339-40,158)	1.0% (0.4-2.2)	6.9% (3.1-13.2)	1,611 (583-3,548)	11,154 (5,063-21,442)
Albania	2019	2.2% (1.1-4.1)	23.7% (19.0-28.9)	2,219 (1,074-4,176)	23,825 (19,157-29,068)	1.0% (0.3-2.1)	9.1% (5.3-14.1)	878 (317-1,892)	8,380 (4,902-13,015)
Bosnia and Herzegovina	1990	1.3% (0.6-2.6)	12.8% (8.8-17.6)	2,722 (1,238-5,260)	25,842 (17,839-35,717)	0.7% (0.3-1.5)	9.0% (4.1-16.2)	1,327 (519-2,863)	17,102 (7,854-30,732)
Bosnia and Herzegovina	2019	1.5% (0.7-2.7)	23.4% (18.2-29.0)	1,391 (657-2,633)	22,399 (17,455-27,821)	0.7% (0.2-1.6)	19.1% (11.8-28.4)	659 (227-1,467)	17,483 (10,797-25,982)
Bulgaria	1990	0.7% (0.3-1.3)	33.7% (25.7-42.3)	2,206 (1,014-4,171)	107,739 (82,195-135,089)	0.4% (0.2-1.0)	41.0% (24.8-59.1)	1,336 (476-3,045)	125,414 (75,919-180,809)
Bulgaria	2019	1.0% (0.5-2.0)	26.2% (21.2-31.9)	1,690 (806-3,196)	42,690 (34,488-51,982)	0.6% (0.2-1.3)	36.3% (27.5-45.7)	877 (329-1,915)	55,532 (42,115-69,986)
Croatia	1990	0.9% (0.4-1.8)	40.2% (34.5-46.6)	1,616 (741-3,155)	69,702 (59,792-80,734)	0.7% (0.3-1.6)	33.2% (25.2-42.0)	1,179 (419-2,632)	55,170 (41,794-69,699)
Croatia	2019	1.1% (0.5-2.0)	32.2% (27.5-36.9)	1,175 (561-2,152)	35,090 (29,973-40,138)	0.8% (0.3-1.7)	30.5% (22.7-38.9)	800 (292-1,748)	31,534 (23,497-40,252)
Czechia	1990	0.4% (0.2-0.8)	37.7% (31.5-43.5)	1,708 (778-3,419)	162,647 (135,694-187,461)	0.3% (0.1-0.6)	23.3% (17.1-30.1)	1,238 (446-2,639)	96,274 (70,706-124,017)
Czechia	2019	0.7% (0.3-1.3)	27.4% (23.6-31.3)	1,683 (788-3,200)	66,574 (57,399-76,275)	0.4% (0.2-0.9)	26.6% (20.6-32.8)	1,015 (374-2,103)	61,215 (47,439-75,383)
Hungary	1990	0.3% (0.2-0.6)	35.6% (30.4-41.1)	1,296 (604-2,404)	140,893 (120,363-162,457)	0.3% (0.1-0.6)	29.5% (22.3-37.5)	985 (358-2,228)	110,991 (83,867-140,770)
Hungary	2019	0.4% (0.2-0.8)	25.0% (19.4-31.1)	990 (450-1,904)	62,163 (48,281-77,378)	0.3% (0.1-0.7)	27.8% (18.5-38.8)	729 (284-1,579)	65,313 (43,641-91,190)

North Macedonia	1990	1.0% (0.5-2.0)	21.3% (15.3-27.9)	921 (417-1,794)	18,775 (13,482-24,572)	0.6% (0.2-1.3)	15.9% (8.1-27.7)	474 (169-1,065)	13,290 (6,761-23,180)
North Macedonia	2019	1.3% (0.6-2.5)	21.2% (17.4-24.9)	808 (379-1,583)	13,349 (10,990-15,690)	0.6% (0.2-1.4)	13.5% (9.6-18.3)	382 (135-811)	7,936 (5,678-10,776)
Montenegro	1990	1.6% (0.7-3.0)	14.9% (10.1-21.3)	427 (193-814)	4,028 (2,720-5,744)	0.7% (0.3-1.7)	12.0% (5.3-21.9)	192 (70-430)	3,087 (1,371-5,653)
Montenegro	2019	1.8% (0.8-3.2)	14.0% (11.3-17.4)	371 (171-678)	2,959 (2,385-3,661)	0.8% (0.3-1.7)	9.1% (5.9-13.2)	151 (57-329)	1,789 (1,156-2,588)
Poland	1990	0.6% (0.3-1.2)	40.5% (37.5-43.5)	9,294 (4,167-17,678)	595,977 (552,524-640,230)	0.5% (0.2-1.1)	22.5% (19.7-25.5)	6,668 (2,439-15,001)	317,199 (277,576-358,505)
Poland	2019	0.7% (0.3-1.4)	27.6% (23.7-31.8)	6,796 (3,182-12,888)	257,184 (220,925-296,728)	0.5% (0.2-1.2)	25.1% (20.1-31.1)	4,722 (1,790-10,252)	222,603 (178,388-275,857)
Romania	1990	0.4% (0.2-0.7)	35.0% (28.6-41.9)	3,440 (1,551-6,486)	343,502 (280,657-411,379)	0.2% (0.1-0.5)	12.0% (7.8-17.6)	2,210 (849-4,646)	113,609 (73,756-166,732)
Romania	2019	0.4% (0.2-0.8)	24.9% (21.1-29.4)	2,369 (1,089-4,453)	131,874 (111,638-155,472)	0.3% (0.1-0.6)	20.4% (15.2-26.4)	1,305 (476-3,030)	101,774 (75,880-131,784)
Serbia	1990	1.1% (0.5-2.1)	13.4% (9.4-18.3)	3,922 (1,827-7,388)	47,817 (33,739-65,597)	0.6% (0.2-1.3)	10.5% (5.0-18.2)	2,105 (783-4,403)	36,326 (17,443-62,981)
Serbia	2019	1.2% (0.6-2.3)	25.7% (22.3-29.7)	3,263 (1,555-6,186)	70,592 (61,195-81,353)	0.7% (0.2-1.6)	31.9% (26.2-37.5)	1,735 (620-3,965)	80,762 (66,336-94,994)
Slovakia	1990	0.3% (0.1-0.6)	37.4% (31.6-43.3)	713 (328-1,342)	81,747 (69,063-94,745)	0.3% (0.1-0.6)	23.2% (15.7-31.8)	539 (191-1,181)	48,892 (33,075-67,089)
Slovakia	2019	0.5% (0.2-0.9)	31.7% (26.3-36.8)	699 (323-1,254)	42,918 (35,702-49,839)	0.3% (0.1-0.7)	32.9% (25.7-40.6)	434 (167-945)	42,281 (33,003-52,182)
Slovenia	1990	0.5% (0.2-1.0)	33.4% (26.8-39.9)	382 (174-738)	24,410 (19,595-29,143)	0.3% (0.1-0.8)	30.1% (20.7-40.2)	238 (90-526)	21,108 (14,483-28,168)
Slovenia	2019	0.8% (0.4-1.5)	26.2% (22.1-30.8)	374 (175-700)	12,474 (10,521-14,628)	0.4% (0.2-1.0)	23.8% (18.4-29.7)	199 (70-432)	10,588 (8,215-13,245)
Belarus	1990	0.6% (0.3-1.2)	41.4% (31.6-50.9)	2,344 (1,080-4,336)	154,012 (117,576-189,414)	0.4% (0.2-1.0)	18.1% (8.1-33.2)	1,600 (592-3,551)	66,135 (29,446-121,077)
Belarus	2019	0.6% (0.3-1.1)	22.9% (18.0-28.2)	1,302 (583-2,513)	52,391 (41,000-64,493)	0.4% (0.1-0.8)	17.8% (11.9-25.4)	766 (281-1,710)	38,270 (25,481-54,644)
Estonia	1990	0.8% (0.4-1.5)	35.9% (31.4-40.4)	467 (216-855)	20,433 (17,843-22,982)	0.5% (0.2-1.0)	19.4% (14.6-24.7)	273 (100-560)	10,345 (7,784-13,153)
Estonia	2019	1.7% (0.8-2.9)	21.8% (18.1-26.0)	528 (251-926)	6,867 (5,694-8,192)	0.8% (0.3-1.7)	18.7% (13.2-25.7)	225 (83-520)	5,596 (3,953-7,686)
Latvia	1990	0.7% (0.3-1.3)	44.4% (38.1-50.6)	649 (303-1,273)	41,964 (36,038-47,859)	0.7% (0.3-1.6)	29.1% (20.8-38.3)	664 (245-1,438)	25,901 (18,503-34,067)
Latvia	2019	0.9% (0.4-1.7)	34.0% (29.4-38.9)	410 (188-750)	15,340 (13,286-17,554)	0.9% (0.3-1.8)	29.9% (22.4-38.7)	381 (141-764)	12,885 (9,667-16,665)
Lithuania	1990	0.4% (0.2-0.7)	37.2% (31.8-43.2)	537 (241-1,042)	52,602 (44,971-61,110)	0.3% (0.1-0.7)	18.3% (12.4-25.5)	396 (146-890)	24,706 (16,781-34,472)
Lithuania	2019	0.6% (0.3-1.0)	30.8% (27.4-34.7)	400 (184-720)	21,909 (19,484-24,629)	0.4% (0.2-0.9)	27.8% (23.4-32.3)	273 (102-588)	18,652 (15,728-21,715)
Republic of Moldova	1990	0.6% (0.3-1.1)	24.7% (17.7-32.4)	1,024 (481-1,914)	43,355 (31,133-56,851)	0.5% (0.2-1.1)	6.9% (3.3-12.4)	864 (308-1,870)	11,694 (5,648-20,915)
Republic of Moldova	2019	0.5% (0.2-1.0)	21.2% (16.8-26.0)	546 (223-1,047)	21,246 (16,922-26,138)	0.5% (0.2-1.1)	6.5% (3.4-11.1)	480 (163-1,039)	6,176 (3,208-10,581)
Russian Federation	1990	1.0% (0.5-1.9)	38.1% (33.1-43.8)	55,580 (24,983-101,899)	2,041,949 (1,777,675-2,349,274)	0.8% (0.3-1.9)	15.0% (10.0-22.5)	42,455 (16,558-98,237)	776,775 (514,291-1,163,519)
Russian Federation	2019	0.9% (0.4-1.8)	19.3% (15.9-22.7)	35,798 (16,935-68,277)	728,692 (600,782-859,515)	0.8% (0.3-1.9)	14.2% (9.7-19.9)	29,209 (9,924-67,039)	513,114 (347,847-717,445)
Ukraine	1990	0.7% (0.3-1.3)	46.8% (40.0-53.6)	12,904 (5,984-24,831)	877,764 (751,114-1,005,145)	0.4% (0.1-0.8)	26.4% (18.1-36.3)	6,737 (2,419-14,584)	476,750 (327,109-654,286)

Ukraine	2019	0.8% (0.4-1.5)	26.6% (21.6-31.9)	8,445 (3,875-16,229)	280,151 (227,751-336,047)	0.4% (0.2-1.0)	15.1% (9.9-21.6)	4,447 (1,637-10,051)	150,101 (98,846-215,464)
Brunei Darussalam	1990	0.5% (0.2-1.0)	27.1% (19.7-35.7)	67 (30-130)	3,412 (2,474-4,490)	0.9% (0.3-1.9)	6.4% (2.6-13.4)	107 (40-227)	755 (308-1,587)
Brunei Darussalam	2019	0.6% (0.3-1.1)	21.9% (16.4-28.0)	109 (50-210)	4,215 (3,158-5,405)	1.0% (0.3-2.1)	7.5% (3.8-12.8)	165 (59-363)	1,268 (648-2,178)
Japan	1990	0.6% (0.3-1.2)	22.6% (16.6-29.8)	33,897 (15,994-63,487)	1,191,284 (873,391-1,572,269)	0.4% (0.1-0.9)	5.8% (2.5-11.7)	21,321 (7,003-47,532)	293,300 (124,592-584,526)
Japan	2019	0.6% (0.3-1.1)	12.6% (8.2-18.2)	18,067 (8,039-34,478)	388,396 (254,038-561,142)	0.5% (0.2-1.2)	3.6% (1.4-7.3)	15,885 (5,515-34,989)	103,587 (40,151-213,613)
Republic of Korea	1990	0.5% (0.2-1.0)	13.3% (8.9-19.2)	11,795 (5,310-22,517)	310,453 (207,957-447,053)	0.4% (0.1-0.9)	5.5% (2.3-10.9)	8,700 (3,103-21,064)	122,840 (51,294-243,505)
Republic of Korea	2019	0.5% (0.2-1.0)	10.9% (8.0-14.6)	6,895 (3,144-13,529)	152,110 (112,310-203,180)	0.4% (0.1-0.8)	4.4% (2.1-8.2)	4,940 (1,671-10,713)	56,341 (27,079-104,451)
Singapore	1990	0.6% (0.3-1.2)	18.7% (13.0-24.6)	913 (413-1,732)	27,208 (19,018-35,921)	0.4% (0.2-0.9)	11.7% (5.6-20.6)	544 (205-1,151)	14,745 (7,068-25,957)
Singapore	2019	0.6% (0.3-1.1)	10.7% (7.6-14.3)	791 (356-1,481)	14,676 (10,428-19,621)	0.6% (0.2-1.2)	7.1% (3.4-13.2)	655 (245-1,436)	8,250 (4,007-15,367)
Australia	1990	0.6% (0.3-1.2)	31.3% (29.3-33.2)	4,564 (2,009-8,476)	221,069 (207,569-234,556)	0.4% (0.2-1.0)	35.1% (32.5-37.7)	2,817 (1,030-6,574)	238,742 (220,774-256,117)
Australia	2019	0.6% (0.3-1.1)	8.7% (6.3-11.4)	4,171 (1,923-8,046)	63,551 (45,988-83,676)	0.6% (0.2-1.3)	8.1% (4.6-12.9)	3,968 (1,486-8,745)	56,650 (32,360-90,231)
New Zealand	1990	0.5% (0.2-0.9)	23.6% (21.6-25.6)	690 (327-1,344)	36,164 (33,067-39,227)	0.9% (0.3-1.9)	26.8% (24.0-29.6)	1,288 (457-2,819)	39,648 (35,570-43,791)
New Zealand	2019	0.5% (0.2-1.0)	17.0% (14.0-20.3)	753 (345-1,448)	25,305 (20,826-30,226)	0.9% (0.3-2.1)	12.8% (9.1-17.7)	1,304 (486-2,959)	18,355 (13,028-25,275)
Andorra	1990	0.2% (0.1-0.4)	33.0% (24.8-41.7)	4 (2-8)	681 (512-862)	0.2% (0.1-0.4)	32.0% (16.9-51.1)	3 (1-7)	606 (319-966)
Andorra	2019	0.2% (0.1-0.4)	23.7% (16.3-32.1)	5 (2-9)	510 (351-693)	0.2% (0.1-0.4)	23.2% (10.3-41.6)	4 (1-8)	474 (210-851)
Austria	1990	0.2% (0.1-0.3)	34.2% (30.5-38.2)	524 (247-917)	91,708 (81,615-102,240)	0.1% (0.1-0.3)	30.6% (24.9-37.0)	382 (142-862)	78,581 (64,077-95,024)
Austria	2019	0.3% (0.1-0.5)	27.8% (21.3-35.2)	603 (274-1,137)	66,258 (50,793-83,783)	0.2% (0.1-0.4)	22.5% (14.4-32.4)	350 (126-764)	48,679 (31,125-69,976)
Belgium	1990	0.1% (0.1-0.3)	33.0% (29.3-37.2)	469 (207-907)	112,395 (99,753-126,680)	0.1% (0.1-0.3)	26.2% (21.7-31.2)	479 (171-1,048)	85,108 (70,646-101,419)
Belgium	2019	0.1% (0.1-0.3)	19.8% (16.2-24.2)	454 (211-848)	63,277 (51,620-77,164)	0.1% (0.1-0.3)	15.9% (11.2-21.7)	458 (168-1,024)	48,520 (34,273-66,504)
Cyprus	1990	0.1% (0.1-0.3)	31.0% (25.6-37.1)	47 (22-93)	9,799 (8,087-11,717)	0.1% (0.1-0.3)	13.9% (8.2-21.7)	42 (16-93)	4,063 (2,406-6,355)
Cyprus	2019	0.2% (0.1-0.3)	30.8% (24.7-37.5)	52 (24-98)	10,397 (8,344-12,676)	0.2% (0.1-0.3)	11.4% (6.4-19.5)	48 (18-105)	3,623 (2,022-6,194)
Denmark	1990	0.2% (0.1-0.4)	29.7% (27.6-31.9)	353 (157-665)	55,729 (51,827-59,853)	0.2% (0.1-0.4)	33.8% (31.4-36.6)	284 (102-651)	60,630 (56,228-65,691)
Denmark	2019	0.2% (0.1-0.4)	16.8% (13.3-20.5)	384 (174-750)	29,508 (23,387-35,954)	0.2% (0.1-0.4)	22.0% (15.8-30.0)	292 (108-645)	36,748 (26,393-50,224)
Finland	1990	0.2% (0.1-0.4)	32.3% (29.3-35.6)	359 (155-667)	50,207 (45,563-55,319)	0.1% (0.0-0.3)	30.3% (26.3-34.5)	212 (74-447)	44,992 (38,973-51,184)
Finland	2019	0.3% (0.1-0.5)	21.6% (17.3-26.8)	436 (202-797)	32,934 (26,349-40,732)	0.2% (0.1-0.3)	22.0% (14.5-31.9)	222 (77-495)	31,758 (20,884-45,987)
France	1990	0.1% (0.1-0.3)	42.5% (39.5-45.3)	3,220 (1,500-6,068)	962,188 (894,568-1,025,941)	0.1% (0.1-0.3)	40.2% (36.5-44.3)	3,171 (1,158-6,737)	869,612 (790,189-957,313)
France	2019	0.1% (0.1-0.3)	31.9% (27.2-37.3)	3,062 (1,383-5,705)	673,883 (573,525-788,037)	0.2% (0.1-0.3)	30.9% (23.7-39.2)	3,026 (1,101-6,542)	619,566 (474,908-786,928)

Germany	1990	0.2% (0.1-0.3)	33.3% (30.0-36.8)	3,462 (1,566-6,636)	748,626 (675,613-827,734)	0.2% (0.1-0.3)	23.9% (20.4-27.8)	3,278 (1,170-7,269)	510,893 (434,648-593,352)
Germany	2019	0.2% (0.1-0.4)	22.3% (18.2-26.6)	4,065 (1,799-7,739)	481,319 (393,350-574,150)	0.2% (0.1-0.4)	18.5% (12.4-25.3)	3,279 (1,173-7,450)	360,095 (242,305-492,204)
Greece	1990	0.1% (0.1-0.3)	27.7% (24.4-31.1)	587 (255-1,126)	111,563 (98,467-125,274)	0.1% (0.1-0.3)	24.1% (21.1-27.2)	564 (203-1,235)	92,547 (81,093-104,532)
Greece	2019	0.2% (0.1-0.3)	22.9% (18.5-28.0)	417 (198-807)	59,723 (48,318-72,872)	0.2% (0.1-0.4)	18.1% (11.4-27.0)	394 (143-890)	45,301 (28,573-67,368)
Iceland	1990	0.6% (0.3-1.1)	29.9% (25.7-34.3)	61 (27-114)	3,234 (2,776-3,703)	0.3% (0.1-0.7)	34.1% (25.6-43.6)	31 (11-67)	3,502 (2,636-4,478)
Iceland	2019	0.9% (0.4-1.8)	12.6% (9.7-16.0)	97 (46-190)	1,359 (1,046-1,727)	0.4% (0.1-0.9)	10.8% (6.9-15.8)	42 (15-91)	1,153 (735-1,693)
Ireland	1990	0.2% (0.1-0.3)	30.2% (28.2-32.4)	276 (123-564)	53,615 (50,081-57,381)	0.1% (0.1-0.3)	23.1% (20.8-25.2)	247 (90-541)	38,958 (35,205-42,623)
Ireland	2019	0.2% (0.1-0.3)	15.2% (11.4-19.8)	261 (122-498)	24,667 (18,513-32,141)	0.2% (0.1-0.3)	17.1% (10.6-24.6)	236 (87-540)	26,757 (16,616-38,479)
Israel	1990	0.2% (0.1-0.4)	27.1% (22.2-32.4)	480 (221-906)	65,984 (54,033-79,007)	0.2% (0.1-0.4)	18.7% (12.9-26.3)	378 (138-880)	42,829 (29,622-60,405)
Israel	2019	0.2% (0.1-0.4)	20.1% (15.7-25.0)	826 (383-1,561)	74,550 (58,354-92,979)	0.2% (0.1-0.4)	9.6% (5.0-16.0)	613 (216-1,337)	33,770 (17,688-56,677)
Italy	1990	0.2% (0.1-0.3)	26.3% (24.2-28.5)	3,944 (1,748-7,735)	588,891 (542,551-637,485)	0.1% (0.0-0.3)	17.9% (15.7-20.3)	3,047 (1,071-6,883)	384,806 (338,139-435,973)
Italy	2019	0.2% (0.1-0.4)	22.0% (18.7-25.6)	2,845 (1,268-5,605)	328,573 (278,895-381,691)	0.2% (0.1-0.3)	17.0% (13.6-21.5)	2,165 (813-4,770)	237,060 (189,080-299,125)
Luxembourg	1990	0.1% (0.1-0.3)	32.0% (25.9-38.0)	16 (8-32)	3,654 (2,961-4,343)	0.1% (0.1-0.3)	24.6% (19.2-31.5)	16 (5-36)	2,643 (2,066-3,387)
Luxembourg	2019	0.1% (0.1-0.3)	20.8% (16.7-25.9)	25 (11-46)	3,574 (2,872-4,453)	0.1% (0.1-0.3)	21.0% (15.1-27.7)	24 (9-53)	3,427 (2,463-4,534)
Malta	1990	0.2% (0.1-0.3)	34.8% (28.8-41.3)	24 (11-47)	4,977 (4,118-5,905)	0.2% (0.1-0.3)	32.6% (23.5-43.2)	20 (7-46)	4,371 (3,152-5,805)
Malta	2019	0.2% (0.1-0.4)	15.2% (11.7-19.0)	21 (9-40)	1,579 (1,215-1,973)	0.2% (0.1-0.3)	17.8% (12.6-24.0)	15 (5-35)	1,800 (1,282-2,428)
Netherlands	1990	0.1% (0.1-0.3)	31.4% (27.8-35.5)	759 (348-1,430)	177,744 (157,395-200,410)	0.1% (0.0-0.3)	34.9% (30.7-38.8)	737 (269-1,599)	189,209 (166,823-210,642)
Netherlands	2019	0.1% (0.1-0.3)	20.0% (16.0-24.5)	745 (339-1,436)	106,317 (84,820-129,835)	0.1% (0.0-0.3)	14.5% (9.9-20.0)	677 (242-1,542)	73,326 (49,925-101,182)
Norway	1990	0.4% (0.2-0.8)	36.4% (33.3-39.4)	693 (303-1,320)	58,388 (53,356-63,200)	0.3% (0.1-0.6)	40.2% (36.6-44.1)	419 (143-944)	61,851 (56,214-67,818)
Norway	2019	0.7% (0.3-1.3)	13.2% (10.3-16.9)	1,185 (560-2,169)	21,806 (17,021-27,913)	0.4% (0.1-0.8)	10.2% (6.5-15.1)	560 (208-1,223)	15,850 (10,123-23,480)
Portugal	1990	0.2% (0.1-0.3)	29.7% (26.9-32.5)	669 (315-1,283)	130,290 (117,862-142,416)	0.2% (0.1-0.3)	22.0% (18.9-25.6)	644 (244-1,344)	93,993 (80,797-109,064)
Portugal	2019	0.2% (0.1-0.3)	27.5% (22.8-32.2)	506 (232-959)	80,932 (67,329-94,818)	0.2% (0.1-0.4)	29.5% (22.2-38.1)	497 (169-1,106)	83,234 (62,702-107,391)
Spain	1990	0.1% (0.1-0.3)	38.1% (35.6-40.5)	2,362 (1,144-4,580)	644,590 (601,997-686,032)	0.1% (0.1-0.3)	39.1% (36.3-42.2)	2,406 (877-5,067)	632,951 (588,234-682,826)
Spain	2019	0.1% (0.1-0.3)	19.5% (15.6-23.7)	1,687 (771-3,156)	226,027 (181,046-274,998)	0.2% (0.1-0.3)	21.7% (16.1-29.1)	1,705 (594-3,780)	238,696 (176,759-320,932)
Sweden	1990	1.4% (0.7-2.6)	26.6% (23.1-30.0)	4,017 (1,884-7,359)	76,729 (66,602-86,512)	0.3% (0.1-0.7)	35.0% (29.3-41.3)	875 (298-1,968)	96,101 (80,527-113,437)
Sweden	2019	1.4% (0.6-2.6)	13.6% (9.7-17.9)	3,963 (1,768-7,489)	39,603 (28,379-52,370)	0.3% (0.1-0.7)	18.0% (10.6-27.0)	915 (312-2,001)	48,723 (28,646-73,102)
Switzerland	1990	0.3% (0.1-0.5)	26.8% (24.6-28.9)	614 (265-1,118)	58,179 (53,528-62,750)	0.2% (0.1-0.4)	22.8% (20.4-25.7)	378 (141-887)	46,603 (41,680-52,542)

Switzerland	2019	0.4% (0.2-0.8)	17.4% (14.0-21.2)	969 (455-1,858)	38,819 (31,275-47,195)	0.2% (0.1-0.5)	12.0% (8.6-16.4)	454 (170-977)	24,831 (17,716-33,942)
United Kingdom	1990	0.1% (0.1-0.3)	30.0% (28.4-31.6)	2,790 (1,283-5,122)	602,567 (570,024-635,139)	0.1% (0.1-0.3)	29.4% (27.5-31.5)	2,816 (1,083-6,357)	567,440 (530,446-606,956)
United Kingdom	2019	0.1% (0.1-0.3)	18.9% (15.3-23.1)	2,751 (1,235-5,371)	357,097 (288,551-435,221)	0.2% (0.1-0.3)	18.3% (12.7-25.1)	2,727 (1,033-6,068)	329,602 (228,504-452,744)
Argentina	1990	0.3% (0.1-0.5)	22.6% (16.1-30.5)	4,059 (1,743-7,759)	325,882 (231,447-438,482)	0.7% (0.3-1.5)	12.8% (5.9-23.5)	10,097 (3,614-21,319)	184,883 (84,795-339,003)
Argentina	2019	0.3% (0.1-0.5)	27.9% (22.9-33.6)	4,706 (2,174-8,914)	504,137 (413,524-607,298)	0.7% (0.2-1.5)	12.4% (6.9-20.1)	11,668 (4,253-26,084)	218,500 (121,861-355,883)
Chile	1990	0.3% (0.2-0.7)	12.4% (8.2-17.1)	2,282 (1,018-4,347)	81,134 (53,521-111,859)	0.4% (0.1-0.9)	39.9% (22.9-59.9)	2,631 (945-5,683)	258,266 (148,432-387,466)
Chile	2019	0.3% (0.1-0.5)	6.7% (4.6-9.4)	1,797 (861-3,405)	42,272 (29,207-59,479)	0.4% (0.1-0.9)	31.7% (19.4-45.5)	2,556 (897-5,588)	191,269 (117,333-274,781)
Uruguay	1990	0.2% (0.1-0.4)	32.2% (25.4-39.6)	290 (134-563)	42,088 (33,252-51,882)	0.3% (0.1-0.8)	38.1% (25.3-53.1)	435 (159-969)	48,551 (32,154-67,583)
Uruguay	2019	0.2% (0.1-0.4)	19.0% (14.9-24.0)	265 (114-506)	24,219 (19,027-30,644)	0.3% (0.1-0.8)	20.4% (13.2-29.0)	419 (156-960)	25,043 (16,194-35,609)
Canada	1990	2.0% (0.9-3.8)	26.0% (23.4-28.6)	19,865 (9,279-37,392)	254,604 (229,388-280,414)	0.3% (0.1-0.6)	28.6% (25.6-31.8)	2,685 (982-5,633)	266,883 (238,750-296,798)
Canada	2019	1.8% (0.8-3.1)	10.1% (7.5-13.3)	18,351 (8,564-32,518)	105,606 (78,421-138,027)	0.3% (0.1-0.7)	9.5% (5.5-14.9)	2,801 (963-6,448)	94,173 (54,187-147,255)
United States of America	1990	5.5% (3.7-7.9)	24.8% (23.6-26.1)	506,706 (341,976-732,002)	2,305,967 (2,191,753-2,422,225)	0.4% (0.2-1.0)	24.4% (22.9-25.8)	39,101 (14,967-88,775)	2,152,510 (2,025,037-2,281,947)
United States of America	2019	5.0% (2.6-8.4)	14.0% (11.3-16.8)	554,672 (285,362-931,510)	1,546,104 (1,254,726-1,858,938)	0.6% (0.2-1.3)	10.4% (7.1-14.2)	58,608 (21,519-133,469)	1,097,427 (754,791-1,504,072)
Antigua and Barbuda	1990	2.5% (1.2-4.6)	5.8% (3.9-8.6)	72 (34-133)	166 (111-246)	1.1% (0.4-2.3)	5.0% (2.2-10.2)	30 (11-65)	141 (62-288)
Antigua and Barbuda	2019	2.2% (1.1-4.1)	8.4% (6.0-11.6)	78 (37-143)	295 (210-405)	1.0% (0.4-2.1)	9.4% (5.8-14.4)	33 (12-72)	315 (195-483)
Bahamas	1990	1.6% (0.7-3.0)	9.5% (6.5-13.7)	216 (100-405)	1,289 (877-1,846)	0.2% (0.1-0.6)	5.4% (2.3-10.8)	33 (11-76)	731 (309-1,452)
Bahamas	2019	1.8% (0.8-3.3)	15.9% (12.4-20.0)	266 (119-508)	2,415 (1,879-3,032)	0.2% (0.1-0.5)	6.6% (3.6-10.8)	37 (13-79)	1,022 (559-1,661)
Barbados	1990	1.4% (0.7-2.7)	10.6% (7.2-14.6)	162 (80-307)	1,213 (821-1,661)	0.5% (0.2-1.3)	9.1% (4.4-16.2)	61 (24-143)	1,010 (495-1,810)
Barbados	2019	1.8% (0.8-3.3)	12.8% (9.1-17.1)	180 (86-338)	1,295 (919-1,738)	0.6% (0.2-1.4)	11.7% (6.8-19.1)	59 (21-137)	1,140 (660-1,861)
Belize	1990	1.8% (0.8-3.5)	21.0% (15.2-28.0)	191 (83-362)	2,175 (1,570-2,901)	0.7% (0.2-1.5)	7.7% (3.5-14.9)	69 (25-151)	795 (358-1,543)
Belize	2019	2.1% (0.9-3.9)	19.6% (14.8-24.7)	456 (209-861)	4,338 (3,269-5,478)	0.7% (0.2-1.6)	8.9% (4.8-14.7)	156 (56-365)	2,010 (1,086-3,306)
Cuba	1990	1.0% (0.5-1.9)	16.5% (11.1-23.2)	6,044 (2,682-11,314)	96,426 (64,467-135,138)	0.6% (0.2-1.2)	13.6% (6.2-24.7)	3,059 (1,056-6,501)	75,417 (34,527-137,054)
Cuba	2019	1.1% (0.5-2.1)	12.2% (9.3-15.7)	3,830 (1,750-7,307)	43,109 (32,921-55,545)	0.6% (0.2-1.4)	10.5% (6.6-15.0)	1,971 (688-4,464)	34,787 (21,855-49,610)
Dominica	1990	0.4% (0.2-0.7)	14.8% (10.4-20.0)	15 (7-29)	599 (422-813)	0.2% (0.1-0.4)	12.2% (5.9-20.6)	8 (3-16)	462 (222-779)
Dominica	2019	0.4% (0.2-0.8)	14.7% (10.2-20.3)	13 (6-24)	437 (302-603)	0.2% (0.1-0.5)	9.3% (4.4-17.1)	6 (2-15)	260 (124-476)
Dominican Republic	1990	1.9% (0.9-3.5)	4.6% (3.1-6.8)	7,452 (3,490-13,540)	17,917 (12,034-26,585)	0.3% (0.1-0.7)	3.6% (1.7-6.7)	1,347 (498-3,008)	15,198 (7,214-28,108)
Dominican Republic	2019	2.1% (1.0-3.8)	4.9% (3.5-6.7)	10,348 (5,048-18,780)	24,052 (17,159-32,552)	0.3% (0.1-0.8)	4.7% (2.6-7.9)	1,674 (611-3,718)	22,811 (12,692-38,234)

Grenada	1990	1.0% (0.4-1.9)	12.2% (8.5-16.9)	44 (19-85)	542 (377-749)	0.4% (0.2-1.0)	8.1% (3.9-15.1)	19 (7-43)	351 (169-651)
Grenada	2019	1.0% (0.5-2.0)	15.8% (12.1-20.1)	45 (21-90)	716 (551-913)	0.5% (0.2-1.2)	9.5% (5.4-14.9)	20 (7-47)	389 (220-606)
Guyana	1990	0.8% (0.4-1.5)	12.4% (8.7-17.0)	360 (170-658)	5,358 (3,742-7,336)	0.6% (0.2-1.3)	7.0% (3.2-13.1)	264 (96-575)	3,097 (1,434-5,816)
Guyana	2019	1.1% (0.5-2.1)	14.5% (11.1-18.9)	424 (199-786)	5,384 (4,103-6,996)	0.8% (0.3-1.7)	7.5% (4.1-12.7)	282 (96-605)	2,679 (1,455-4,558)
Haiti	1990	0.3% (0.1-0.5)	10.3% (6.9-14.5)	883 (430-1,661)	31,600 (21,167-44,438)	0.4% (0.2-1.0)	6.3% (2.8-11.8)	1,424 (562-3,259)	20,711 (9,082-38,652)
Haiti	2019	0.3% (0.1-0.6)	5.7% (3.8-8.2)	1,906 (858-3,664)	34,553 (23,173-49,754)	0.5% (0.2-1.1)	3.7% (1.7-7.0)	2,944 (1,015-6,493)	23,043 (10,466-43,020)
Jamaica	1990	1.4% (0.6-2.6)	14.5% (10.1-20.2)	1,791 (803-3,338)	18,539 (12,929-25,810)	0.6% (0.2-1.2)	9.4% (4.6-16.3)	737 (273-1,595)	12,211 (6,006-21,072)
Jamaica	2019	1.5% (0.7-2.9)	17.9% (14.8-21.8)	1,913 (918-3,581)	22,371 (18,508-27,192)	0.6% (0.2-1.3)	14.4% (10.4-19.1)	726 (265-1,538)	17,288 (12,470-22,973)
Saint Lucia	1990	1.8% (0.9-3.4)	15.6% (11.0-20.8)	137 (64-257)	1,180 (833-1,570)	0.6% (0.2-1.4)	9.1% (4.2-17.1)	50 (18-111)	697 (322-1,313)
Saint Lucia	2019	2.2% (1.0-4.2)	13.6% (10.3-17.4)	152 (71-287)	933 (704-1,194)	0.8% (0.3-1.7)	11.3% (6.8-17.4)	50 (18-113)	750 (446-1,150)
Saint Vincent and the Grenadines	1990	1.0% (0.5-1.9)	19.4% (14.3-25.7)	62 (29-122)	1,239 (914-1,641)	0.5% (0.2-1.1)	11.6% (6.2-19.3)	29 (10-66)	715 (386-1,196)
Saint Vincent and the Grenadines	2019	0.9% (0.4-1.9)	25.4% (20.1-31.3)	43 (20-88)	1,200 (951-1,480)	0.4% (0.2-1.0)	11.8% (6.5-19.4)	19 (7-43)	523 (287-861)
Suriname	1990	2.4% (1.2-4.5)	29.2% (22.3-36.8)	498 (238-923)	5,997 (4,581-7,557)	0.7% (0.3-1.7)	11.8% (5.9-21.2)	142 (49-321)	2,264 (1,129-4,078)
Suriname	2019	2.4% (1.1-4.3)	25.1% (20.1-30.1)	577 (277-1,048)	6,087 (4,859-7,292)	0.7% (0.2-1.5)	10.9% (6.4-16.8)	151 (56-338)	2,491 (1,459-3,826)
Trinidad and Tobago	1990	1.9% (0.9-3.7)	24.1% (17.7-30.9)	1,121 (505-2,148)	13,853 (10,154-17,754)	0.8% (0.3-1.8)	11.9% (5.7-21.3)	455 (182-979)	6,621 (3,200-11,837)
Trinidad and Tobago	2019	2.0% (0.9-3.7)	24.3% (20.1-29.3)	895 (421-1,690)	11,098 (9,161-13,355)	0.8% (0.3-1.9)	12.6% (8.1-18.0)	359 (133-856)	5,561 (3,556-7,936)
Bolivia (Plurinational State of)	1990	2.4% (1.2-4.6)	20.5% (13.9-28.3)	7,929 (3,915-14,948)	66,967 (45,591-92,581)	1.5% (0.6-3.0)	9.5% (3.9-18.7)	4,836 (1,827-10,091)	31,415 (12,831-61,928)
Bolivia (Plurinational State of)	2019	2.4% (1.1-4.4)	22.5% (17.8-28.6)	12,828 (6,082-24,025)	121,298 (96,234-154,373)	1.4% (0.5-3.0)	13.0% (7.8-19.9)	7,299 (2,697-15,665)	68,516 (41,158-105,148)
Ecuador	1990	0.7% (0.3-1.2)	16.4% (11.4-22.9)	3,511 (1,570-6,646)	88,339 (61,332-123,449)	0.4% (0.2-0.9)	3.9% (1.6-7.7)	2,338 (828-4,953)	21,353 (8,617-42,372)
Ecuador	2019	0.6% (0.3-1.1)	9.5% (6.9-12.9)	4,933 (2,426-9,305)	79,791 (58,085-107,766)	0.4% (0.1-0.9)	4.7% (2.6-7.7)	3,315 (1,215-7,190)	38,249 (21,059-63,159)
Peru	1990	1.4% (0.6-2.7)	15.7% (10.8-22.1)	16,824 (7,571-31,237)	183,389 (125,430-258,035)	1.4% (0.5-3.1)	5.6% (2.3-11.4)	16,629 (6,064-36,086)	66,257 (26,619-133,595)
Peru	2019	1.3% (0.6-2.5)	15.2% (11.7-19.1)	20,121 (8,907-37,519)	230,092 (177,241-288,481)	1.2% (0.4-2.7)	5.5% (3.0-9.0)	16,022 (5,667-36,522)	73,986 (40,296-121,996)
Colombia	1990	1.3% (0.6-2.4)	35.2% (26.7-44.2)	22,392 (10,262-40,322)	587,664 (445,861-737,578)	1.1% (0.4-2.5)	18.4% (8.9-31.6)	19,039 (6,652-43,695)	318,494 (153,629-546,684)
Colombia	2019	1.4% (0.6-2.6)	14.6% (11.1-18.8)	28,485 (13,152-52,887)	297,572 (225,894-381,911)	1.2% (0.4-2.6)	8.1% (4.7-13.0)	22,428 (8,074-50,818)	158,787 (92,534-253,777)
Costa Rica	1990	0.4% (0.2-0.8)	25.3% (19.5-32.0)	616 (285-1,153)	37,323 (28,759-47,283)	0.4% (0.1-0.8)	22.8% (12.7-36.4)	550 (201-1,240)	33,303 (18,635-53,262)
Costa Rica	2019	0.4% (0.2-0.7)	12.5% (9.4-16.1)	710 (332-1,298)	22,667 (16,920-29,202)	0.4% (0.1-0.8)	9.8% (5.6-15.7)	660 (254-1,419)	17,717 (10,138-28,449)
El Salvador	1990	1.2% (0.5-2.2)	18.6% (13.0-25.0)	3,447 (1,541-6,341)	54,502 (38,159-73,151)	1.0% (0.4-2.2)	3.8% (1.5-8.0)	3,005 (1,080-6,711)	11,503 (4,481-24,176)

El Salvador	2019	1.2% (0.5-2.1)	24.0% (19.4-29.0)	3,456 (1,559-6,275)	70,462 (56,939-85,144)	1.0% (0.4-2.1)	9.5% (5.6-14.4)	2,858 (1,036-6,223)	27,551 (16,382-41,835)
Guatemala	1990	0.6% (0.3-1.1)	23.9% (17.4-31.7)	2,267 (1,031-4,546)	94,736 (69,041-125,686)	0.4% (0.2-1.0)	4.7% (2.1-9.3)	1,808 (658-4,074)	19,602 (8,646-38,333)
Guatemala	2019	0.6% (0.3-1.1)	20.2% (16.4-25.0)	5,673 (2,643-10,585)	195,097 (158,093-240,828)	0.4% (0.1-1.0)	11.6% (7.1-17.7)	4,197 (1,337-9,447)	108,488 (66,292-164,918)
Honduras	1990	0.5% (0.2-1.0)	26.9% (20.1-34.8)	1,223 (558-2,404)	67,312 (50,219-87,113)	0.5% (0.2-1.1)	4.6% (2.2-8.5)	1,311 (454-2,785)	11,681 (5,715-21,577)
Honduras	2019	0.6% (0.3-1.1)	18.5% (14.7-22.9)	2,945 (1,393-5,602)	98,495 (78,039-121,589)	0.6% (0.2-1.3)	9.5% (5.8-14.4)	3,131 (1,141-6,758)	50,632 (30,869-76,684)
Mexico	1990	0.8% (0.4-1.6)	42.5% (34.4-50.9)	40,173 (18,696-78,509)	2,077,804 (1,683,833-2,487,684)	0.6% (0.2-1.3)	16.9% (9.5-26.9)	32,109 (12,386-68,016)	853,343 (480,707-1,357,961)
Mexico	2019	0.8% (0.4-1.6)	18.7% (15.1-22.5)	45,181 (20,177-89,408)	1,027,116 (828,146-1,236,471)	0.7% (0.2-1.4)	9.6% (5.7-14.9)	36,162 (13,337-76,800)	519,798 (309,860-811,956)
Nicaragua	1990	1.8% (0.9-3.4)	38.2% (29.7-47.6)	3,829 (1,816-7,055)	80,322 (62,504-100,122)	1.3% (0.4-2.8)	11.3% (5.5-21.2)	2,818 (978-6,187)	24,497 (12,049-46,228)
Nicaragua	2019	1.9% (0.9-3.6)	26.2% (20.7-32.0)	5,984 (2,745-11,383)	83,859 (66,309-102,613)	1.3% (0.5-2.9)	12.2% (7.1-18.8)	4,053 (1,402-8,998)	37,452 (21,701-57,802)
Panama	1990	0.7% (0.3-1.4)	19.3% (14.1-26.1)	924 (416-1,786)	24,633 (18,034-33,344)	0.7% (0.3-1.5)	15.6% (7.9-26.5)	865 (320-1,892)	19,545 (9,861-33,233)
Panama	2019	0.7% (0.3-1.3)	11.4% (8.3-14.8)	1,282 (605-2,394)	20,375 (14,914-26,527)	0.8% (0.3-1.7)	6.6% (3.6-11.1)	1,297 (484-2,863)	11,334 (6,209-18,951)
Venezuela (Bolivarian Republic of)	1990	2.4% (1.1-4.4)	19.9% (14.2-26.7)	23,713 (11,337-44,288)	198,015 (141,506-266,389)	0.9% (0.3-1.8)	14.5% (7.1-24.5)	8,469 (3,053-17,665)	142,906 (70,136-241,635)
Venezuela (Bolivarian Republic of)	2019	2.4% (1.1-4.5)	12.4% (8.8-16.9)	27,289 (12,126-50,896)	141,810 (100,347-192,252)	0.9% (0.3-1.9)	8.9% (4.1-16.7)	9,523 (3,576-20,874)	99,765 (46,288-187,232)
Brazil	1990	0.6% (0.3-1.1)	26.8% (21.4-32.1)	43,382 (19,746-82,783)	2,031,313 (1,624,776-2,435,192)	0.4% (0.1-0.9)	15.1% (11.4-18.8)	30,732 (11,425-68,033)	1,161,424 (875,190-1,446,081)
Brazil	2019	0.9% (0.4-1.7)	6.2% (4.7-7.9)	76,916 (35,774-141,561)	518,721 (394,174-655,029)	0.5% (0.2-1.1)	4.5% (2.5-7.3)	39,532 (14,940-86,267)	363,001 (205,980-588,993)
Paraguay	1990	1.8% (0.8-3.2)	27.2% (20.2-35.3)	3,573 (1,604-6,487)	54,345 (40,400-70,572)	0.8% (0.3-1.7)	10.4% (4.6-19.5)	1,542 (568-3,363)	20,732 (9,241-38,993)
Paraguay	2019	1.8% (0.8-3.4)	13.9% (10.2-18.4)	5,907 (2,750-11,216)	45,982 (33,609-60,695)	0.7% (0.3-1.6)	6.9% (3.8-11.9)	2,300 (880-4,929)	21,801 (11,979-37,770)
Algeria	1990	5.2% (2.5-9.6)	22.5% (15.6-31.0)	73,243 (35,212-135,624)	318,670 (220,840-439,406)	0.5% (0.2-1.0)	2.4% (0.9-5.4)	6,484 (2,298-13,969)	33,141 (12,021-74,386)
Algeria	2019	5.9% (2.9-10.2)	23.1% (18.6-27.9)	91,387 (44,522-158,626)	358,086 (289,193-433,591)	0.5% (0.2-1.0)	2.1% (0.8-4.0)	6,815 (2,458-14,179)	30,832 (12,528-59,468)
Bahrain	1990	2.4% (1.1-4.5)	16.8% (12.9-21.2)	445 (203-826)	3,093 (2,387-3,904)	0.7% (0.3-1.7)	5.4% (2.9-9.3)	139 (50-315)	1,001 (535-1,739)
Bahrain	2019	2.5% (1.1-4.6)	32.2% (26.5-38.3)	1,084 (502-2,033)	14,247 (11,703-16,961)	0.7% (0.3-1.6)	10.6% (6.7-15.8)	306 (109-659)	4,440 (2,792-6,618)
Egypt	1990	0.7% (0.3-1.3)	13.0% (9.0-18.0)	20,880 (10,264-39,093)	384,724 (267,586-532,918)	0.3% (0.1-0.7)	2.3% (1.0-4.8)	9,062 (3,200-20,105)	64,098 (27,470-132,966)
Egypt	2019	0.7% (0.3-1.3)	16.2% (13.2-19.7)	32,554 (15,038-62,779)	780,203 (634,431-947,332)	0.4% (0.1-0.8)	2.8% (1.3-5.0)	15,871 (5,730-34,514)	123,103 (58,965-220,466)
Iran (Islamic Republic of)	1990	1.7% (0.8-3.2)	9.3% (6.3-13.5)	54,498 (25,593-101,267)	295,477 (198,484-425,411)	0.5% (0.2-1.0)	3.6% (1.4-7.7)	13,573 (5,097-30,068)	109,111 (43,055-231,734)
Iran (Islamic Republic of)	2019	1.7% (0.8-3.0)	11.9% (8.6-16.1)	46,244 (21,525-83,745)	332,699 (239,822-451,158)	0.4% (0.2-0.9)	4.9% (2.3-9.1)	11,415 (4,105-24,770)	130,430 (62,264-243,570)
Iraq	1990	1.1% (0.5-2.1)	21.5% (15.2-29.1)	11,192 (5,011-20,823)	212,591 (150,201-287,992)	0.5% (0.2-1.0)	4.7% (1.8-9.9)	4,430 (1,712-9,364)	44,660 (17,140-94,510)
Iraq	2019	1.1% (0.5-2.0)	25.5% (20.8-30.7)	24,032 (10,725-46,307)	576,932 (471,261-695,728)	0.5% (0.2-1.0)	5.8% (3.2-10.1)	9,918 (3,854-22,148)	124,191 (68,250-215,946)

Jordan	1990	3.0% (1.4-5.8)	32.4% (25.0-40.6)	7,579 (3,528-14,525)	80,488 (62,088-100,887)	0.7% (0.3-1.5)	12.8% (6.6-21.9)	1,565 (607-3,288)	28,112 (14,601-48,159)
Jordan	2019	2.9% (1.3-5.4)	32.9% (28.5-37.8)	18,271 (8,383-33,669)	204,951 (177,706-235,666)	0.6% (0.2-1.4)	14.9% (9.6-21.9)	3,597 (1,279-7,969)	84,274 (54,376-123,666)
Kuwait	1990	2.1% (1.0-3.9)	31.5% (23.8-40.1)	1,406 (649-2,569)	20,691 (15,597-26,300)	0.4% (0.2-1.0)	7.2% (3.2-13.4)	295 (103-662)	4,837 (2,190-9,020)
Kuwait	2019	2.2% (1.0-4.3)	35.8% (30.7-41.4)	2,639 (1,214-5,195)	43,426 (37,180-50,160)	0.5% (0.2-1.0)	13.9% (8.9-19.7)	514 (191-1,142)	15,801 (10,177-22,437)
Lebanon	1990	1.4% (0.7-2.7)	25.4% (18.9-32.3)	2,433 (1,139-4,531)	42,837 (31,869-54,534)	0.8% (0.3-1.7)	12.4% (6.3-21.1)	1,163 (416-2,544)	18,680 (9,480-31,983)
Lebanon	2019	1.4% (0.6-2.4)	35.2% (29.7-41.1)	2,462 (1,140-4,362)	63,578 (53,594-74,193)	0.7% (0.3-1.5)	13.3% (8.6-19.2)	1,123 (425-2,432)	21,289 (13,849-30,860)
Libya	1990	2.1% (1.0-4.1)	15.4% (10.4-21.4)	5,255 (2,513-9,981)	37,985 (25,581-52,547)	0.8% (0.3-1.7)	2.4% (1.0-5.1)	1,841 (659-4,021)	5,679 (2,261-11,930)
Libya	2019	2.2% (1.0-4.2)	16.1% (11.5-21.6)	6,538 (3,040-12,650)	48,300 (34,615-64,913)	0.8% (0.3-1.8)	2.8% (1.2-5.5)	2,248 (815-5,097)	8,043 (3,306-15,908)
Morocco	1990	2.5% (1.2-4.6)	14.8% (10.4-20.5)	33,304 (15,595-61,510)	199,266 (139,419-275,932)	0.9% (0.3-1.8)	2.3% (0.9-4.9)	11,544 (4,126-24,559)	31,227 (11,813-65,855)
Morocco	2019	2.6% (1.2-4.8)	10.7% (8.0-13.8)	40,640 (19,075-75,347)	169,720 (126,135-218,049)	0.9% (0.3-1.8)	2.6% (1.3-4.6)	13,358 (5,207-27,579)	39,735 (19,524-70,763)
Palestine	1990	3.2% (1.5-6.0)	16.4% (12.5-20.8)	3,644 (1,708-6,972)	18,954 (14,421-24,028)	1.1% (0.4-2.4)	1.9% (0.7-3.8)	1,248 (472-2,613)	2,057 (799-4,126)
Palestine	2019	3.4% (1.6-6.5)	20.0% (15.1-26.1)	9,004 (4,126-17,318)	53,104 (40,207-69,351)	1.2% (0.5-2.6)	4.4% (2.1-8.1)	3,028 (1,196-6,604)	11,281 (5,452-20,563)
Oman	1990	2.4% (1.1-4.6)	9.2% (6.6-12.8)	2,152 (992-4,035)	8,067 (5,784-11,238)	0.5% (0.2-1.2)	1.6% (0.6-3.2)	395 (136-891)	1,183 (450-2,365)
Oman	2019	2.2% (1.0-4.1)	9.5% (7.0-12.6)	2,920 (1,338-5,346)	12,376 (9,091-16,401)	0.5% (0.2-1.0)	2.4% (1.0-4.8)	593 (216-1,276)	3,075 (1,293-6,020)
Qatar	1990	3.4% (1.6-6.4)	18.4% (12.6-24.6)	540 (258-1,002)	2,897 (1,977-3,870)	0.8% (0.3-1.8)	9.8% (4.1-19.0)	94 (33-221)	1,224 (518-2,380)
Qatar	2019	3.4% (1.6-6.1)	26.9% (20.9-33.8)	2,176 (1,060-3,903)	17,313 (13,476-21,776)	0.6% (0.2-1.4)	11.6% (6.3-18.4)	272 (99-595)	4,952 (2,673-7,837)
Saudi Arabia	1990	1.9% (1.0-3.6)	8.4% (5.8-11.4)	17,481 (8,689-32,171)	75,449 (52,427-102,698)	0.5% (0.2-1.0)	1.8% (0.7-3.9)	3,511 (1,323-7,617)	14,205 (5,603-30,264)
Saudi Arabia	2019	1.8% (0.8-3.5)	11.9% (8.7-15.8)	26,906 (12,207-50,767)	173,976 (127,092-231,659)	0.5% (0.2-1.0)	3.7% (1.7-6.7)	5,407 (2,057-11,885)	43,023 (19,724-78,287)
Syrian Arab Republic	1990	0.9% (0.4-1.6)	25.1% (18.2-32.2)	6,351 (2,914-11,832)	184,415 (133,784-236,789)	0.7% (0.2-1.5)	11.0% (5.0-20.1)	4,716 (1,597-10,383)	77,750 (35,107-142,093)
Syrian Arab Republic	2019	1.5% (0.7-2.8)	22.0% (16.4-29.0)	13,547 (6,009-25,981)	202,496 (150,741-266,396)	0.9% (0.3-2.0)	8.7% (4.1-15.3)	8,165 (2,950-17,900)	79,210 (37,665-140,181)
Tunisia	1990	2.6% (1.1-4.9)	32.3% (24.3-40.8)	11,743 (5,197-22,058)	145,901 (110,068-184,330)	0.9% (0.3-2.1)	5.0% (2.2-9.8)	4,024 (1,466-8,989)	21,650 (9,402-42,527)
Tunisia	2019	2.2% (1.0-4.0)	25.4% (21.2-30.0)	9,013 (4,282-16,521)	105,189 (87,713-124,218)	0.8% (0.3-1.9)	3.7% (1.9-6.5)	3,289 (1,280-7,458)	14,545 (7,315-25,650)
Turkey	1990	1.0% (0.5-1.9)	39.8% (30.3-49.3)	34,038 (15,778-62,915)	1,299,828 (989,241-1,608,890)	0.5% (0.2-1.0)	13.4% (8.4-19.6)	14,470 (5,176-32,231)	419,908 (262,526-614,200)
Turkey	2019	0.9% (0.4-1.7)	41.5% (37.3-46.6)	28,939 (11,993-56,835)	1,391,599 (1,250,785-1,559,746)	0.4% (0.1-0.8)	28.3% (22.8-34.4)	11,569 (4,385-25,567)	899,020 (722,601-1,090,833)
United Arab Emirates	1990	2.8% (1.3-5.2)	10.5% (7.2-14.4)	1,780 (852-3,378)	6,783 (4,667-9,305)	0.9% (0.3-2.0)	2.6% (1.1-5.3)	491 (187-1,074)	1,393 (563-2,831)
United Arab Emirates	2019	2.8% (1.3-5.2)	15.7% (12.0-19.8)	4,966 (2,287-9,284)	27,903 (21,311-35,156)	0.9% (0.3-2.0)	5.3% (2.8-9.3)	1,546 (558-3,362)	8,794 (4,662-15,457)
Yemen	1990	5.8% (2.8-10.7)	12.2% (8.3-17.4)	39,480 (18,910-73,622)	83,621 (56,730-119,718)	2.2% (0.8-4.8)	2.9% (1.1-5.8)	14,026 (5,063-30,389)	18,300 (6,883-36,668)

Yemen	2019	5.2% (2.5-9.6)	15.6% (11.8-19.9)	90,353 (42,228-165,844)	268,125 (202,428-342,775)	2.0% (0.8-4.2)	5.0% (2.6-8.5)	33,157 (12,463-69,072)	81,519 (42,919-140,096)
Afghanistan	1990	7.2% (3.5-12.9)	3.4% (2.2-5.1)	50,721 (24,796-91,317)	24,109 (15,241-36,011)	0.4% (0.1-0.7)	0.8% (0.3-1.8)	2,491 (912-5,181)	5,684 (2,064-12,232)
Afghanistan	2019	7.9% (4.0-13.7)	11.8% (8.5-16.0)	177,341 (89,544-305,535)	263,562 (189,852-358,182)	0.3% (0.1-0.8)	2.0% (0.8-4.2)	7,243 (2,673-16,373)	41,992 (16,973-89,085)
Bangladesh	1990	9.0% (4.5-15.5)	20.3% (15.2-26.2)	511,981 (256,508-878,651)	1,153,446 (865,029-1,485,340)	5.1% (2.0-11.0)	1.0% (0.4-2.2)	302,784 (119,080-648,191)	60,206 (23,062-129,155)
Bangladesh	2019	10.0% (5.8-15.8)	14.2% (10.9-18.3)	741,177 (427,390-1,172,066)	1,048,204 (802,670-1,350,309)	5.1% (2.2-10.0)	1.1% (0.4-2.1)	402,545 (177,719-793,059)	83,927 (33,649-162,605)
Bhutan	1990	16.6% (8.7-27.3)	31.8% (23.4-41.6)	6,513 (3,425-10,762)	12,500 (9,227-16,361)	6.1% (2.5-12.6)	9.7% (4.3-19.3)	2,051 (848-4,204)	3,225 (1,438-6,448)
Bhutan	2019	24.0% (16.3-33.8)	36.6% (29.4-44.3)	8,560 (5,837-12,079)	13,065 (10,490-15,822)	11.5% (5.8-19.3)	13.2% (7.9-19.8)	3,978 (2,009-6,665)	4,573 (2,733-6,849)
India	1990	14.1% (7.3-24.3)	8.9% (6.6-11.9)	6,153,997 (3,182,159-10,607,681)	3,887,724 (2,865,143-5,194,949)	3.4% (1.3-7.1)	2.4% (1.0-4.7)	1,352,816 (532,861-2,830,243)	981,574 (394,449-1,884,826)
India	2019	12.4% (7.4-19.0)	7.4% (5.4-10.0)	8,710,309 (5,205,779-13,305,703)	5,215,734 (3,816,598-6,977,404)	3.0% (1.2-6.3)	2.2% (1.0-4.0)	1,916,220 (757,738-4,037,267)	1,385,332 (633,334-2,577,652)
Nepal	1990	15.8% (8.6-26.2)	17.3% (12.4-23.6)	150,009 (81,825-249,693)	164,225 (118,312-224,492)	2.5% (1.0-5.3)	5.0% (2.3-9.6)	24,512 (9,258-51,197)	48,650 (21,913-93,090)
Nepal	2019	15.4% (9.5-23.6)	12.6% (9.8-16.1)	250,806 (155,125-384,313)	205,060 (159,944-261,477)	2.2% (0.9-4.8)	2.3% (1.0-4.4)	36,959 (14,461-80,129)	38,668 (17,106-73,270)
Pakistan	1990	9.3% (4.7-16.7)	11.7% (7.9-16.6)	577,715 (292,948-1,031,138)	726,000 (492,183-1,027,543)	2.0% (0.7-4.4)	1.2% (0.5-2.6)	114,670 (39,984-248,026)	67,229 (25,512-147,286)
Pakistan	2019	8.6% (4.6-14.4)	6.8% (4.9-9.2)	1,097,358 (582,745-1,836,776)	869,295 (624,326-1,173,666)	2.0% (0.8-4.2)	1.2% (0.5-2.3)	240,284 (93,936-498,199)	138,586 (58,373-274,251)
Angola	1990	0.4% (0.2-0.7)	4.4% (2.8-6.7)	1,963 (927-3,703)	23,137 (14,666-35,325)	0.3% (0.1-0.6)	1.3% (0.5-2.9)	1,450 (537-3,280)	6,750 (2,473-15,175)
Angola	2019	0.4% (0.2-0.7)	3.5% (2.3-5.2)	5,736 (2,505-11,077)	53,208 (34,050-78,472)	0.3% (0.1-0.7)	1.1% (0.4-2.3)	4,400 (1,638-10,354)	16,755 (6,211-36,343)
Central African Republic	1990	0.5% (0.2-0.9)	10.2% (6.7-15.1)	629 (282-1,223)	13,966 (9,158-20,630)	0.4% (0.2-1.0)	2.1% (0.8-4.7)	621 (231-1,351)	2,916 (1,098-6,538)
Central African Republic	2019	0.5% (0.2-0.9)	6.7% (4.4-9.6)	1,346 (594-2,509)	19,240 (12,802-27,680)	0.4% (0.2-0.9)	1.6% (0.6-3.6)	1,277 (503-2,662)	4,832 (1,695-10,688)
Congo	1990	0.3% (0.1-0.5)	11.3% (7.5-15.6)	346 (161-677)	15,209 (10,140-21,130)	0.3% (0.1-0.6)	2.6% (1.1-5.2)	414 (149-880)	3,556 (1,523-7,260)
Congo	2019	0.3% (0.1-0.5)	12.8% (8.8-17.3)	658 (302-1,247)	32,124 (22,134-43,422)	0.3% (0.1-0.7)	4.0% (1.7-7.6)	770 (284-1,715)	10,342 (4,466-19,661)
Democratic Republic of the Congo	1990	0.5% (0.2-1.0)	5.1% (3.2-7.4)	10,261 (4,751-19,715)	101,127 (64,176-147,071)	0.5% (0.2-1.1)	0.5% (0.2-1.0)	9,812 (3,660-21,671)	9,008 (3,414-19,329)
Democratic Republic of the Congo	2019	0.5% (0.2-1.0)	4.5% (2.9-6.7)	24,884 (11,237-49,446)	217,708 (140,286-323,201)	0.5% (0.2-1.0)	0.5% (0.2-1.0)	21,556 (8,010-46,814)	22,321 (7,831-47,014)
Equatorial Guinea	1990	0.5% (0.2-0.9)	22.0% (15.4-30.6)	96 (41-178)	4,541 (3,178-6,312)	0.5% (0.2-1.0)	8.1% (3.3-16.9)	98 (36-222)	1,755 (724-3,665)
Equatorial Guinea	2019	0.5% (0.2-0.8)	23.1% (17.2-29.2)	485 (224-894)	24,725 (18,430-31,336)	0.4% (0.2-1.0)	8.4% (3.9-15.5)	330 (118-727)	6,294 (2,900-11,597)
Gabon	1990	0.5% (0.2-0.9)	11.8% (7.9-16.6)	234 (105-456)	5,739 (3,852-8,060)	0.5% (0.2-1.2)	5.3% (2.0-10.4)	260 (96-595)	2,755 (1,047-5,390)
Gabon	2019	0.5% (0.2-1.0)	16.1% (12.3-20.6)	430 (203-829)	13,677 (10,440-17,420)	0.5% (0.2-1.3)	8.2% (4.3-14.0)	503 (179-1,164)	7,578 (4,037-12,965)
Burundi	1990	0.7% (0.3-1.4)	7.2% (4.6-10.7)	1,954 (911-3,576)	18,741 (12,110-27,808)	1.0% (0.4-2.2)	1.6% (0.6-3.5)	2,792 (1,033-6,057)	4,418 (1,658-9,535)
Burundi	2019	0.6% (0.3-1.1)	3.0% (1.9-4.4)	3,638 (1,716-6,697)	18,228 (11,645-26,653)	0.9% (0.3-1.9)	0.9% (0.4-2.0)	5,786 (2,020-12,504)	5,958 (2,308-13,224)

Comoros	1990	1.5% (0.7-2.8)	16.8% (11.1-23.0)	376 (170-720)	4,328 (2,878-5,946)	1.4% (0.5-3.1)	5.8% (2.4-11.9)	356 (124-812)	1,483 (615-3,071)
Comoros	2019	1.5% (0.7-2.8)	15.5% (11.9-19.6)	544 (254-1,024)	5,608 (4,314-7,118)	1.4% (0.5-3.2)	6.2% (3.2-10.6)	514 (195-1,158)	2,198 (1,150-3,786)
Djibouti	1990	2.6% (1.2-4.8)	14.9% (9.9-20.9)	788 (367-1,474)	4,573 (3,041-6,422)	1.5% (0.6-3.3)	4.5% (1.8-9.3)	363 (137-810)	1,095 (444-2,259)
Djibouti	2019	2.6% (1.3-4.6)	15.8% (11.6-20.3)	1,526 (746-2,688)	9,252 (6,772-11,903)	1.6% (0.6-3.3)	6.3% (3.3-11.3)	708 (286-1,490)	2,889 (1,504-5,131)
Eritrea	1990	1.7% (0.8-3.2)	4.4% (2.8-6.5)	2,919 (1,324-5,361)	7,500 (4,670-10,962)	0.3% (0.1-0.7)	0.6% (0.2-1.4)	481 (172-1,161)	1,005 (353-2,279)
Eritrea	2019	1.4% (0.6-2.6)	3.4% (2.2-5.0)	5,426 (2,430-10,210)	13,133 (8,410-19,478)	0.3% (0.1-0.7)	0.5% (0.2-1.2)	1,186 (445-2,528)	2,044 (684-4,511)
Ethiopia	1990	0.6% (0.3-1.2)	2.1% (1.4-3.2)	15,426 (6,989-30,227)	55,091 (35,435-84,414)	0.3% (0.1-0.8)	0.5% (0.2-1.1)	8,367 (2,931-19,407)	12,181 (4,296-27,677)
Ethiopia	2019	0.6% (0.3-1.1)	2.1% (1.3-3.1)	36,599 (17,271-70,189)	131,960 (82,491-195,662)	0.3% (0.1-0.7)	0.4% (0.1-0.8)	18,920 (6,783-42,338)	23,539 (8,342-51,730)
Kenya	1990	0.8% (0.4-1.4)	11.1% (7.6-15.5)	9,901 (4,525-17,957)	142,974 (97,769-200,282)	0.7% (0.3-1.5)	3.5% (1.5-7.4)	9,119 (3,375-19,782)	45,983 (20,369-98,228)
Kenya	2019	0.8% (0.4-1.6)	6.2% (4.3-8.9)	24,401 (10,954-47,426)	184,950 (126,671-264,451)	0.7% (0.3-1.5)	2.2% (1.0-4.1)	20,965 (7,777-44,873)	63,816 (29,288-119,270)
Madagascar	1990	5.5% (2.6-10.1)	29.2% (21.4-37.9)	34,501 (16,302-63,291)	183,919 (134,677-238,268)	3.8% (1.4-8.0)	4.7% (1.8-9.9)	24,068 (9,142-51,262)	30,366 (11,684-63,565)
Madagascar	2019	4.6% (2.4-8.0)	17.3% (13.4-22.2)	68,646 (35,464-118,060)	255,804 (197,797-328,569)	3.4% (1.3-7.3)	4.2% (2.1-7.7)	50,295 (19,562-107,564)	61,915 (30,251-112,147)
Malawi	1990	0.7% (0.3-1.4)	5.3% (3.4-7.6)	3,569 (1,663-6,838)	25,361 (16,561-36,788)	0.8% (0.3-1.7)	1.5% (0.6-3.3)	4,128 (1,460-8,841)	7,671 (2,969-16,654)
Malawi	2019	0.7% (0.3-1.3)	5.9% (4.1-8.4)	7,944 (3,747-15,167)	66,357 (46,143-94,421)	0.8% (0.3-1.6)	2.6% (1.2-4.9)	8,810 (3,532-18,850)	30,191 (13,963-57,789)
Mauritius	1990	1.6% (0.7-2.9)	40.2% (31.4-49.8)	822 (370-1,479)	20,632 (16,118-25,554)	1.3% (0.5-2.9)	13.0% (6.0-22.9)	642 (240-1,440)	6,495 (3,020-11,457)
Mauritius	2019	1.7% (0.8-3.1)	36.6% (31.1-42.0)	799 (390-1,466)	17,244 (14,639-19,779)	1.4% (0.5-3.0)	15.7% (10.2-22.7)	664 (249-1,399)	7,261 (4,718-10,458)
Mozambique	1990	1.2% (0.5-2.4)	7.3% (4.7-10.7)	7,721 (3,391-14,830)	45,476 (29,563-67,225)	1.3% (0.5-2.9)	1.6% (0.6-3.6)	9,214 (3,326-20,642)	11,559 (4,545-25,318)
Mozambique	2019	1.4% (0.6-2.6)	6.1% (4.1-8.6)	20,968 (9,785-40,974)	95,123 (63,639-132,492)	1.3% (0.5-2.7)	2.5% (1.1-4.9)	22,031 (8,123-46,660)	43,239 (18,970-83,861)
Rwanda	1990	0.5% (0.2-0.9)	3.2% (1.9-4.9)	1,645 (784-3,184)	11,689 (7,046-17,905)	0.9% (0.4-2.0)	0.6% (0.2-1.3)	3,520 (1,354-7,644)	2,118 (719-5,018)
Rwanda	2019	0.4% (0.2-0.8)	3.1% (2.0-4.5)	2,821 (1,329-5,473)	21,645 (14,249-31,469)	0.9% (0.3-2.1)	0.6% (0.2-1.2)	6,367 (2,027-15,003)	3,932 (1,564-8,680)
Seychelles	1990	1.8% (0.8-3.6)	34.3% (26.0-43.9)	69 (31-133)	1,285 (973-1,641)	1.2% (0.5-2.7)	18.5% (9.3-31.7)	46 (17-100)	696 (348-1,189)
Seychelles	2019	1.7% (0.8-3.4)	29.6% (23.8-36.2)	62 (29-121)	1,054 (849-1,289)	1.0% (0.4-2.1)	15.0% (9.6-22.1)	34 (12-70)	508 (324-749)
Somalia	1990	0.9% (0.4-1.7)	8.1% (5.2-11.6)	3,775 (1,752-7,096)	34,291 (22,132-49,021)	0.7% (0.3-1.6)	3.4% (1.4-6.9)	2,734 (983-5,730)	12,656 (5,004-25,393)
Somalia	2019	0.9% (0.4-1.7)	6.4% (4.2-9.3)	10,429 (4,869-20,071)	76,422 (50,086-111,030)	0.8% (0.3-1.8)	2.7% (1.1-5.7)	8,437 (2,989-19,558)	30,020 (11,695-63,349)
United Republic of Tanzania	1990	0.6% (0.3-1.0)	6.5% (4.1-9.6)	7,764 (3,686-14,165)	89,063 (56,368-131,525)	0.5% (0.2-1.0)	2.0% (0.9-4.2)	6,764 (2,502-15,273)	30,109 (12,871-62,552)
United Republic of Tanzania	2019	0.6% (0.3-1.1)	4.8% (3.3-6.9)	17,980 (7,917-33,436)	142,199 (97,050-204,682)	0.5% (0.2-1.1)	1.9% (0.8-3.9)	15,402 (5,508-34,129)	59,586 (24,932-122,269)
Uganda	1990	0.8% (0.4-1.4)	8.0% (5.1-11.5)	7,236 (3,351-13,157)	72,423 (46,551-104,887)	0.7% (0.3-1.5)	2.7% (1.0-5.6)	6,720 (2,454-14,686)	26,077 (10,017-54,228)

Uganda	2019	0.8% (0.4-1.7)	7.5% (5.5-9.8)	20,564 (8,875-40,284)	182,272 (134,028-238,411)	0.7% (0.2-1.6)	5.0% (2.6-8.9)	16,783 (5,804-38,342)	121,045 (63,669-214,686)
Zambia	1990	0.8% (0.4-1.5)	8.9% (5.7-12.6)	3,562 (1,630-6,917)	40,508 (25,883-57,812)	1.0% (0.3-2.1)	3.2% (1.3-6.4)	4,788 (1,610-10,469)	15,440 (6,234-31,437)
Zambia	2019	0.9% (0.4-1.7)	15.7% (12.2-19.8)	8,877 (4,246-16,765)	157,088 (121,969-198,686)	1.2% (0.5-2.6)	9.1% (5.0-15.2)	12,556 (4,737-27,485)	96,174 (52,807-159,965)
Botswana	1990	1.6% (0.7-2.8)	20.0% (14.1-27.6)	1,127 (532-2,048)	14,370 (10,113-19,880)	6.5% (2.6-13.2)	6.9% (2.9-14.1)	5,080 (2,008-10,290)	5,429 (2,255-11,013)
Botswana	2019	1.6% (0.8-3.3)	22.5% (17.6-28.2)	1,768 (831-3,512)	24,135 (18,891-30,281)	6.9% (3.6-11.9)	8.1% (4.7-12.9)	7,351 (3,773-12,582)	8,635 (4,939-13,655)
Lesotho	1990	1.5% (0.7-2.8)	19.5% (13.9-26.1)	1,509 (711-2,725)	19,086 (13,591-25,555)	0.5% (0.2-1.1)	2.6% (1.0-5.3)	524 (196-1,114)	2,629 (1,043-5,334)
Lesotho	2019	1.4% (0.6-2.6)	19.1% (14.5-25.0)	1,505 (685-2,899)	21,146 (16,015-27,676)	0.5% (0.2-1.1)	2.6% (1.1-5.3)	516 (196-1,164)	2,843 (1,204-5,748)
Namibia	1990	1.2% (0.6-2.3)	18.7% (13.0-25.6)	960 (460-1,841)	14,814 (10,286-20,291)	0.9% (0.3-2.0)	9.7% (4.3-17.3)	725 (267-1,611)	7,887 (3,502-14,130)
Namibia	2019	1.1% (0.5-2.1)	14.8% (10.8-19.2)	1,313 (604-2,491)	17,575 (12,806-22,798)	0.8% (0.3-1.8)	11.7% (6.4-19.6)	1,012 (379-2,124)	14,224 (7,798-23,816)
South Africa	1990	3.0% (1.4-5.5)	24.7% (19.7-30.8)	59,041 (27,942-107,038)	480,467 (383,823-600,916)	2.5% (0.9-5.2)	12.9% (6.9-20.3)	51,405 (19,688-108,061)	267,751 (144,242-420,688)
South Africa	2019	2.1% (1.0-4.0)	20.9% (16.8-25.6)	49,410 (23,377-92,988)	484,262 (388,560-592,630)	2.2% (0.8-4.7)	16.4% (9.4-25.1)	49,357 (18,694-104,184)	365,305 (209,920-561,434)
Eswatini	1990	0.5% (0.2-0.9)	13.4% (10.2-17.0)	220 (98-415)	5,893 (4,501-7,493)	0.4% (0.2-0.9)	3.2% (1.4-6.1)	203 (78-431)	1,500 (680-2,893)
Eswatini	2019	0.5% (0.2-0.9)	7.5% (5.2-10.6)	288 (130-561)	4,665 (3,206-6,567)	0.4% (0.2-0.9)	2.6% (1.1-4.9)	255 (92-552)	1,541 (679-2,938)
Zimbabwe	1990	0.7% (0.3-1.3)	9.0% (5.8-13.4)	3,954 (1,721-7,629)	53,586 (34,467-79,629)	0.6% (0.2-1.3)	1.1% (0.4-2.4)	3,746 (1,396-8,014)	6,837 (2,594-14,476)
Zimbabwe	2019	0.7% (0.3-1.3)	12.3% (9.0-16.5)	5,696 (2,577-10,312)	100,361 (73,672-134,669)	0.8% (0.3-1.9)	4.6% (2.3-8.4)	6,869 (2,547-16,201)	38,852 (19,075-70,265)
Benin	1990	1.2% (0.6-2.3)	7.5% (4.9-11.2)	2,667 (1,257-4,918)	16,396 (10,678-24,347)	0.4% (0.2-0.9)	1.5% (0.5-3.2)	985 (349-2,076)	3,370 (1,236-7,370)
Benin	2019	1.3% (0.6-2.6)	4.4% (2.9-6.2)	8,572 (4,104-16,759)	28,736 (19,189-40,694)	0.4% (0.2-1.0)	1.0% (0.4-2.1)	2,877 (1,034-6,626)	6,709 (2,740-14,217)
Burkina Faso	1990	0.5% (0.2-1.0)	12.3% (8.2-17.8)	2,424 (1,089-4,500)	56,691 (37,556-81,774)	0.9% (0.3-2.0)	1.9% (0.7-4.0)	4,314 (1,527-9,626)	8,944 (3,197-19,082)
Burkina Faso	2019	0.5% (0.2-1.0)	8.7% (5.9-12.5)	6,287 (2,868-12,066)	102,109 (69,270-146,625)	0.9% (0.3-2.1)	1.3% (0.4-2.8)	10,853 (4,007-25,098)	15,089 (5,346-33,159)
Cameroon	1990	0.4% (0.2-0.8)	4.9% (3.1-7.3)	2,259 (1,008-4,281)	25,440 (16,214-37,678)	0.4% (0.2-0.9)	1.1% (0.4-2.3)	2,366 (873-4,994)	5,717 (2,142-12,541)
Cameroon	2019	0.5% (0.2-0.9)	5.0% (3.4-7.0)	7,451 (3,402-14,318)	79,866 (55,132-112,743)	0.5% (0.2-1.0)	1.8% (0.9-3.2)	7,493 (2,637-16,574)	28,264 (13,845-51,402)
Cabo Verde	1990	0.5% (0.2-0.9)	8.8% (5.7-12.7)	88 (42-166)	1,621 (1,057-2,357)	0.6% (0.2-1.3)	2.9% (1.1-6.3)	114 (43-241)	540 (204-1,166)
Cabo Verde	2019	0.5% (0.2-0.9)	4.6% (3.0-6.7)	125 (60-239)	1,148 (762-1,696)	0.6% (0.2-1.3)	2.0% (0.8-4.2)	143 (52-320)	500 (198-1,040)
Chad	1990	0.4% (0.2-0.7)	9.6% (6.2-14.0)	1,039 (477-1,922)	27,937 (18,043-40,526)	0.3% (0.1-0.7)	3.3% (1.4-6.7)	1,016 (373-2,291)	10,270 (4,293-20,548)
Chad	2019	0.4% (0.2-0.7)	6.6% (4.4-9.5)	3,125 (1,442-5,931)	57,670 (38,893-82,732)	0.3% (0.1-0.8)	2.5% (1.0-5.2)	3,050 (1,147-6,895)	22,611 (9,302-47,034)
Côte d'Ivoire	1990	0.3% (0.2-0.7)	12.6% (8.6-17.7)	2,077 (929-4,077)	75,856 (51,694-106,021)	0.5% (0.2-1.0)	3.5% (1.4-7.4)	2,860 (1,074-6,347)	21,353 (8,328-45,478)
Côte d'Ivoire	2019	0.4% (0.2-0.7)	16.2% (11.7-21.6)	4,832 (2,189-9,362)	221,247 (159,792-295,187)	0.5% (0.2-1.1)	4.5% (1.9-8.7)	6,007 (2,128-13,552)	56,845 (24,262-110,512)

Gambia	1990	0.8% (0.3-1.4)	16.9% (11.6-23.7)	372 (172-681)	8,291 (5,686-11,654)	0.4% (0.1-0.8)	2.6% (1.0-5.6)	180 (66-412)	1,317 (495-2,896)
Gambia	2019	0.8% (0.4-1.5)	15.3% (11.6-19.4)	1,016 (467-1,928)	19,377 (14,704-24,578)	0.4% (0.1-0.9)	2.9% (1.4-5.2)	495 (175-1,134)	3,793 (1,816-6,897)
Ghana	1990	0.4% (0.2-0.8)	4.6% (3.1-6.6)	3,459 (1,640-6,585)	36,060 (23,806-51,131)	0.5% (0.2-1.1)	1.5% (0.5-3.2)	3,769 (1,406-8,313)	11,121 (4,084-24,818)
Ghana	2019	0.5% (0.2-1.0)	5.1% (3.6-7.3)	8,446 (3,883-16,719)	84,056 (58,753-120,133)	0.6% (0.2-1.3)	1.7% (0.7-3.2)	9,744 (3,433-21,784)	27,874 (12,123-52,996)
Guinea	1990	0.5% (0.2-0.9)	17.2% (11.8-24.3)	1,465 (674-2,641)	48,667 (33,287-68,573)	0.4% (0.2-1.0)	1.4% (0.5-3.1)	1,294 (516-2,880)	4,003 (1,423-9,030)
Guinea	2019	0.5% (0.2-0.9)	15.6% (11.0-21.5)	3,245 (1,466-6,085)	99,799 (70,703-138,207)	0.5% (0.2-1.1)	1.6% (0.6-3.4)	3,199 (1,139-7,224)	10,714 (4,141-23,196)
Guinea-Bissau	1990	0.5% (0.2-1.0)	2.8% (1.8-4.2)	267 (127-498)	1,483 (926-2,187)	0.5% (0.2-1.1)	0.7% (0.2-1.5)	250 (93-580)	378 (127-800)
Guinea-Bissau	2019	0.5% (0.2-1.0)	3.2% (2.0-4.8)	529 (234-1,068)	3,224 (2,068-4,858)	0.5% (0.2-1.1)	0.8% (0.3-1.7)	497 (180-1,102)	824 (296-1,804)
Liberia	1990	0.4% (0.2-0.8)	3.3% (2.1-5.1)	393 (176-728)	3,056 (1,933-4,687)	0.3% (0.1-0.7)	1.5% (0.6-3.3)	323 (116-719)	1,621 (632-3,491)
Liberia	2019	0.4% (0.2-0.8)	3.7% (2.5-5.6)	1,079 (480-2,149)	9,850 (6,628-14,756)	0.3% (0.1-0.7)	3.8% (1.8-6.7)	830 (281-1,886)	10,044 (4,810-17,839)
Mali	1990	0.7% (0.3-1.3)	8.6% (5.7-12.3)	2,608 (1,234-5,052)	34,329 (23,002-49,375)	0.5% (0.2-1.0)	0.8% (0.3-1.7)	2,011 (715-4,239)	3,295 (1,220-7,112)
Mali	2019	0.7% (0.3-1.3)	13.3% (9.5-17.9)	8,052 (3,895-15,391)	162,088 (115,518-218,391)	0.5% (0.2-1.0)	1.2% (0.5-2.5)	5,827 (2,066-12,724)	14,979 (5,968-31,002)
Mauritania	1990	1.2% (0.6-2.2)	36.1% (27.6-45.1)	1,255 (590-2,311)	37,351 (28,599-46,653)	1.2% (0.4-2.7)	13.9% (7.0-23.7)	1,234 (456-2,854)	14,790 (7,464-25,199)
Mauritania	2019	1.4% (0.6-2.7)	17.7% (14.4-21.5)	3,091 (1,374-5,880)	38,820 (31,557-47,101)	1.3% (0.5-2.9)	10.7% (7.0-15.0)	3,007 (1,130-6,620)	24,615 (16,126-34,563)
Niger	1990	0.8% (0.4-1.5)	14.7% (10.1-20.4)	2,941 (1,367-5,610)	55,031 (37,745-76,563)	0.5% (0.2-1.1)	2.3% (1.0-4.5)	2,101 (768-4,663)	9,290 (3,964-18,381)
Niger	2019	0.8% (0.4-1.5)	12.2% (8.5-16.6)	9,699 (4,489-17,992)	151,023 (105,704-204,932)	0.6% (0.2-1.4)	2.3% (1.0-4.7)	7,633 (2,784-18,155)	30,472 (12,963-60,976)
Nigeria	1990	0.3% (0.2-0.7)	3.6% (2.4-5.3)	14,861 (7,075-29,711)	158,168 (105,737-231,277)	0.3% (0.1-0.5)	0.9% (0.4-2.0)	12,687 (4,854-26,654)	45,993 (18,545-101,384)
Nigeria	2019	0.3% (0.1-0.6)	2.0% (1.2-2.9)	37,141 (17,105-68,733)	226,255 (142,038-332,708)	0.3% (0.1-0.6)	0.5% (0.2-1.1)	31,704 (11,953-72,524)	63,025 (23,869-136,628)
Sao Tome and Principe	1990	0.3% (0.1-0.5)	1.0% (0.6-1.5)	17 (8-34)	65 (40-98)	0.3% (0.1-0.6)	0.4% (0.1-1.0)	18 (6-41)	28 (9-65)
Sao Tome and Principe	2019	0.3% (0.1-0.5)	1.1% (0.7-1.7)	29 (13-54)	126 (78-193)	0.3% (0.1-0.6)	0.5% (0.2-1.1)	30 (11-68)	55 (20-128)
Senegal	1990	0.4% (0.2-0.7)	24.2% (17.5-31.5)	1,426 (659-2,707)	92,515 (67,003-120,131)	0.6% (0.2-1.2)	3.5% (1.4-6.9)	2,421 (867-5,098)	14,269 (5,857-28,366)
Senegal	2019	0.3% (0.1-0.6)	9.9% (7.3-13.1)	2,649 (1,238-5,213)	86,463 (63,445-114,623)	0.5% (0.2-1.0)	2.1% (0.9-4.0)	3,874 (1,465-8,064)	17,012 (7,634-32,478)
Sierra Leone	1990	0.8% (0.3-1.6)	16.0% (10.8-22.3)	1,308 (572-2,556)	26,382 (17,811-36,715)	0.6% (0.3-1.4)	7.8% (3.3-15.5)	1,164 (452-2,545)	14,059 (5,959-28,026)
Sierra Leone	2019	0.9% (0.4-1.7)	14.4% (11.2-18.4)	4,096 (1,999-7,617)	65,532 (50,852-83,612)	0.7% (0.3-1.7)	7.0% (3.7-11.8)	3,318 (1,202-7,825)	33,098 (17,542-55,895)
Togo	1990	0.7% (0.3-1.4)	14.0% (9.5-19.0)	1,433 (661-2,652)	27,101 (18,481-36,808)	0.6% (0.2-1.3)	2.9% (1.1-6.5)	1,113 (413-2,521)	5,704 (2,213-12,731)
Togo	2019	0.7% (0.3-1.2)	7.5% (5.3-10.4)	2,805 (1,303-5,150)	31,341 (22,044-43,393)	0.5% (0.2-1.2)	1.9% (0.8-3.5)	2,054 (747-4,681)	7,470 (3,148-13,804)
American Samoa	1990	8.9% (4.3-16.1)	30.9% (22.5-40.7)	227 (110-410)	786 (573-1,036)	5.0% (2.0-10.6)	19.8% (9.0-35.3)	119 (46-251)	466 (213-833)

American Samoa	2019	9.0% (4.7-16.1)	30.0% (21.6-39.4)	281 (146-501)	933 (673-1,227)	5.4% (2.0-11.2)	18.7% (9.3-32.4)	159 (59-331)	553 (274-957)
Bermuda	1990	0.9% (0.4-1.7)	12.1% (8.0-17.7)	19 (9-35)	246 (162-359)	0.4% (0.2-0.9)	6.3% (2.3-12.9)	8 (3-17)	124 (46-255)
Bermuda	2019	1.2% (0.5-2.2)	12.6% (8.7-17.3)	18 (8-34)	195 (135-268)	0.5% (0.2-1.1)	6.0% (2.6-11.1)	7 (2-17)	93 (40-171)
Cook Islands	1990	9.1% (4.5-15.7)	37.4% (28.2-47.4)	96 (47-165)	394 (296-498)	5.7% (2.1-12.3)	48.3% (29.9-67.7)	55 (20-119)	470 (291-658)
Cook Islands	2019	8.1% (4.5-14.3)	33.0% (26.0-40.6)	54 (30-94)	218 (172-269)	5.1% (2.1-10.1)	45.6% (29.9-61.3)	36 (15-71)	323 (211-434)
Greenland	1990	4.2% (1.9-7.7)	52.3% (45.2-59.3)	81 (37-149)	1,014 (876-1,149)	0.4% (0.1-0.9)	59.5% (47.7-71.2)	7 (2-15)	1,027 (824-1,230)
Greenland	2019	4.1% (1.9-7.2)	47.5% (38.4-56.2)	73 (34-128)	848 (685-1,004)	0.5% (0.2-1.1)	57.1% (41.2-71.9)	9 (3-19)	1,029 (742-1,296)
Guam	1990	9.3% (4.6-16.7)	31.1% (23.0-40.3)	617 (304-1,102)	2,057 (1,519-2,668)	5.8% (2.1-13.0)	22.4% (11.5-37.7)	338 (122-754)	1,305 (669-2,192)
Guam	2019	14.0% (9.1-19.3)	25.8% (21.9-30.0)	962 (628-1,327)	1,780 (1,508-2,069)	9.0% (5.2-14.6)	14.7% (10.3-19.7)	571 (326-922)	928 (649-1,250)
Monaco	1990	0.2% (0.1-0.4)	30.7% (22.3-40.3)	1 (1-3)	207 (151-272)	0.2% (0.1-0.4)	28.5% (13.4-48.5)	1 (0-2)	183 (86-312)
Monaco	2019	0.2% (0.1-0.4)	22.2% (14.9-31.0)	2 (1-4)	209 (140-290)	0.2% (0.1-0.4)	21.2% (9.5-38.8)	2 (1-3)	188 (84-344)
Nauru	1990	0.9% (0.4-1.7)	47.3% (37.3-57.8)	4 (2-8)	236 (186-288)	0.6% (0.2-1.4)	41.8% (24.1-63.1)	3 (1-7)	205 (118-310)
Nauru	2019	0.8% (0.4-1.5)	44.6% (36.3-52.7)	5 (2-9)	259 (211-307)	0.5% (0.2-1.2)	35.6% (23.0-50.1)	3 (1-7)	196 (127-275)
Niue	1990	0.9% (0.4-1.7)	15.2% (10.1-21.2)	1 (0-2)	18 (12-25)	1.5% (0.5-3.3)	10.5% (4.3-21.1)	1 (1-3)	10 (4-20)
Niue	2019	1.0% (0.4-1.8)	13.6% (9.5-18.5)	1 (0-1)	10 (7-14)	1.5% (0.5-3.0)	9.7% (5.1-17.3)	1 (0-2)	6 (3-11)
Northern Mariana Islands	1990	23.4% (12.7-36.4)	43.4% (33.9-54.0)	438 (238-682)	813 (635-1,011)	17.1% (7.8-32.1)	46.2% (27.6-65.3)	373 (170-701)	1,009 (604-1,428)
Northern Mariana Islands	2019	27.8% (18.0-40.1)	31.5% (24.2-39.4)	616 (399-889)	698 (537-874)	21.6% (12.4-34.1)	21.9% (14.8-31.1)	444 (254-700)	449 (304-638)
Palau	1990	22.8% (12.6-36.7)	38.1% (29.2-47.3)	190 (105-306)	318 (243-395)	12.3% (4.9-24.4)	23.5% (12.0-37.5)	96 (38-189)	183 (93-292)
Palau	2019	30.6% (19.6-44.3)	46.7% (41.8-51.3)	189 (122-274)	289 (259-317)	20.8% (12.2-31.5)	34.0% (28.3-40.4)	116 (68-175)	189 (157-224)
Puerto Rico	1990	0.7% (0.3-1.3)	13.1% (9.4-17.7)	1,159 (545-2,263)	22,253 (15,975-30,136)	0.5% (0.2-1.0)	7.7% (3.9-13.7)	757 (255-1,584)	12,681 (6,417-22,652)
Puerto Rico	2019	0.8% (0.4-1.5)	9.0% (6.3-12.2)	985 (428-1,795)	10,545 (7,377-14,312)	0.4% (0.1-0.9)	4.0% (1.8-7.7)	486 (164-1,022)	4,566 (2,081-8,787)
Saint Kitts and Nevis	1990	0.6% (0.3-1.1)	5.9% (3.5-8.5)	12 (5-23)	126 (76-182)	0.3% (0.1-0.7)	2.5% (0.9-5.3)	7 (2-15)	53 (20-113)
Saint Kitts and Nevis	2019	0.7% (0.3-1.4)	5.8% (3.7-8.6)	16 (7-30)	129 (83-191)	0.4% (0.1-0.8)	2.6% (1.0-5.7)	8 (3-19)	57 (22-126)
San Marino	1990	0.2% (0.1-0.4)	30.2% (21.6-39.6)	2 (1-3)	285 (203-374)	0.1% (0.1-0.3)	30.1% (14.5-49.4)	1 (1-3)	290 (140-476)
San Marino	2019	0.2% (0.1-0.4)	17.7% (13.5-22.2)	2 (1-4)	182 (139-229)	0.1% (0.1-0.3)	13.8% (9.3-19.9)	1 (1-4)	141 (95-204)
Tokelau	1990	8.9% (4.2-16.3)	31.3% (22.4-41.5)	8 (4-14)	26 (19-35)	5.1% (1.9-10.7)	16.4% (6.8-32.1)	4 (1-7)	11 (5-22)
Tokelau	2019	9.0% (4.3-16.3)	30.3% (21.8-40.3)	6 (3-11)	20 (14-26)	5.4% (2.0-11.7)	15.7% (6.2-29.8)	3 (1-6)	9 (3-16)

Tuvalu	1990	9.0% (4.5-15.9)	20.9% (14.3-29.2)	36 (18-64)	84 (57-117)	5.1% (1.9-10.7)	5.7% (2.2-12.1)	20 (7-42)	23 (9-48)
Tuvalu	2019	9.1% (4.5-16.6)	31.7% (26.3-37.6)	56 (28-102)	196 (162-232)	5.2% (2.0-10.6)	14.0% (9.1-20.7)	28 (11-57)	76 (49-112)
United States Virgin Islands	1990	0.3% (0.1-0.5)	3.6% (2.3-5.3)	14 (6-26)	176 (113-261)	0.2% (0.1-0.4)	0.7% (0.3-1.6)	9 (3-20)	35 (13-78)
United States Virgin Islands	2019	0.4% (0.2-0.7)	2.7% (1.7-4.0)	12 (6-22)	90 (58-131)	0.2% (0.1-0.4)	0.6% (0.2-1.3)	6 (2-13)	18 (7-41)
South Sudan	1990	1.6% (0.8-3.1)	9.1% (6.0-13.0)	5,704 (2,650-10,874)	32,147 (21,020-45,953)	1.0% (0.4-2.3)	2.0% (0.7-4.4)	3,189 (1,136-7,231)	6,419 (2,100-13,942)
South Sudan	2019	1.7% (0.8-3.1)	8.0% (5.4-11.0)	9,714 (4,454-18,226)	46,749 (31,944-64,457)	1.0% (0.4-2.3)	1.9% (0.8-3.9)	5,566 (1,992-12,543)	10,233 (4,143-20,862)
Sudan	1990	5.8% (2.8-10.6)	12.2% (8.3-17.2)	62,704 (30,608-114,540)	132,226 (90,216-186,231)	0.4% (0.1-0.9)	2.5% (1.0-5.4)	4,067 (1,450-9,109)	26,731 (10,559-57,561)
Sudan	2019	6.4% (3.2-11.9)	9.3% (6.5-12.9)	148,185 (73,127-273,590)	214,740 (150,390-296,326)	0.4% (0.1-0.8)	2.8% (1.2-5.4)	7,553 (2,684-17,185)	60,226 (26,828-115,894)

Supplementary Table 4. Number (in thousands) of current chewing tobacco users in 1990 and 2019 for males and females aged 15 plus. Locations organized alphabetically.

	Females		Males	
**Location**	**1990**	**2019**	**1990**	**2019**
Afghanistan	13.2 (9.8-17.6)	35.2 (25.1-49.3)	250.2 (196.1-312.9)	1,217.1 (996.9-1,464.4)
Albania	4.6 (3.1-6.8)	4.0 (3.0-5.5)	14.0 (10.6-18.5)	12.0 (9.5-14.9)
Algeria	28.1 (20.0-38.6)	49.7 (38.1-63.8)	538.3 (436.0-657.4)	1,181.0 (965.3-1,435.4)
American Samoa	0.4 (0.2-0.5)	0.5 (0.3-0.7)	0.8 (0.6-1.1)	1.0 (0.8-1.3)
Andorra	0.0 (0.0-0.0)	0.1 (0.0-0.1)	0.0 (0.0-0.1)	0.1 (0.1-0.1)
Angola	8.5 (6.2-11.6)	26.0 (19.4-34.9)	16.4 (12.8-20.8)	44.7 (34.7-56.6)
Antigua and Barbuda	0.1 (0.1-0.1)	0.1 (0.1-0.2)	0.2 (0.1-0.3)	0.3 (0.2-0.4)
Argentina	30.5 (21.3-43.4)	42.2 (30.2-58.9)	18.0 (13.9-22.5)	25.5 (20.2-32.2)
Armenia	2.5 (1.9-3.5)	2.4 (1.8-3.1)	2.8 (2.2-3.6)	2.6 (2.1-3.3)
Australia	15.3 (11.5-20.9)	25.7 (19.8-33.3)	39.2 (31.5-48.2)	53.9 (44.3-65.7)
Austria	5.0 (3.8-6.5)	6.3 (4.9-8.2)	6.5 (5.3-7.9)	10.4 (8.5-12.9)
Azerbaijan	4.9 (3.5-6.8)	7.5 (5.6-9.9)	6.8 (5.2-8.9)	11.0 (8.6-14.0)
Bahamas	0.2 (0.1-0.2)	0.2 (0.2-0.3)	0.6 (0.4-0.8)	0.8 (0.6-1.1)
Bahrain	0.6 (0.4-0.8)	1.6 (1.2-2.2)	3.9 (3.0-5.0)	13.1 (10.2-16.7)
Bangladesh	6,080.5 (5,095.0-7,103.5)	13,541.1 (12,245.7-14,894.9)	6,119.4 (5,254.6-7,044.7)	12,106.1 (10,917.7-13,354.3)
Barbados	0.2 (0.2-0.3)	0.3 (0.2-0.4)	0.3 (0.2-0.5)	0.4 (0.3-0.6)
Belarus	9.8 (7.2-13.2)	8.4 (6.4-11.0)	10.7 (8.3-13.9)	8.8 (7.0-10.9)
Belgium	5.7 (4.4-7.4)	6.6 (5.1-8.5)	5.7 (4.6-7.0)	6.9 (5.6-8.5)
Belize	0.2 (0.1-0.3)	0.5 (0.3-0.7)	0.4 (0.3-0.6)	1.2 (0.9-1.7)
Benin	8.4 (6.5-11.0)	22.0 (16.8-28.7)	25.5 (20.8-30.6)	78.7 (64.2-97.7)
Bermuda	0.1 (0.0-0.1)	0.1 (0.1-0.1)	0.1 (0.1-0.1)	0.1 (0.1-0.1)
Bhutan	17.7 (14.1-21.6)	33.8 (27.9-39.9)	45.6 (38.0-53.5)	79.3 (70.1-88.5)
Bolivia (Plurinational State of)	14.1 (9.3-21.5)	25.2 (16.9-36.5)	22.7 (16.3-30.6)	41.9 (30.9-57.9)

Bosnia and Herzegovina	5.4 (4.0-7.5)	4.4 (3.3-5.7)	9.9 (7.3-13.2)	7.5 (6.0-9.5)
Botswana	24.0 (18.4-31.4)	53.0 (42.8-65.5)	3.6 (2.7-4.9)	7.6 (5.8-9.9)
Brazil	148.6 (110.7-198.8)	291.2 (224.7-374.9)	177.6 (140.0-231.2)	357.9 (283.8-442.1)
Brunei Darussalam	0.7 (0.5-1.0)	1.5 (1.1-2.1)	0.7 (0.5-0.9)	1.5 (1.2-1.8)
Bulgaria	12.0 (9.1-15.7)	13.8 (10.6-17.4)	20.8 (16.7-25.8)	24.4 (19.5-29.9)
Burkina Faso	78.7 (59.0-102.7)	201.0 (154.8-258.0)	44.5 (36.0-53.9)	108.8 (89.0-133.2)
Burundi	32.0 (23.9-43.2)	64.8 (48.5-85.0)	37.9 (30.4-46.1)	76.1 (61.5-92.7)
Cabo Verde	1.7 (1.3-2.1)	3.1 (2.4-4.1)	1.1 (0.9-1.3)	2.4 (1.9-2.9)
Cambodia	299.4 (249.8-354.9)	663.1 (569.7-767.4)	33.9 (27.5-41.3)	86.8 (70.3-105.7)
Cameroon	23.6 (18.1-29.8)	69.8 (53.7-89.6)	15.7 (12.7-19.3)	47.6 (38.5-58.5)
Canada	27.4 (20.8-35.8)	34.9 (26.8-44.8)	178.4 (144.3-220.4)	198.9 (162.4-241.6)
Central African Republic	5.8 (4.3-7.6)	10.4 (7.8-13.4)	3.6 (2.8-4.7)	7.4 (5.8-9.2)
Chad	16.3 (12.6-21.1)	36.8 (28.1-47.0)	15.1 (12.2-18.2)	37.5 (30.9-45.5)
Chile	10.9 (7.8-15.1)	15.6 (11.6-20.4)	12.6 (10.0-16.0)	15.0 (12.1-18.8)
China	984.3 (715.6-1,367.6)	1,276.7 (965.2-1,665.3)	3,325.4 (2,614.9-4,214.0)	5,142.6 (4,095.0-6,432.1)
Colombia	57.0 (38.9-86.5)	90.4 (65.2-125.2)	75.9 (55.6-99.7)	120.4 (92.0-155.7)
Comoros	4.9 (3.7-6.4)	9.9 (7.5-12.7)	6.0 (5.0-7.3)	13.0 (10.7-15.4)
Congo	4.9 (3.7-6.4)	12.4 (9.3-16.3)	2.5 (1.9-3.1)	7.0 (5.4-9.0)
Cook Islands	0.2 (0.1-0.2)	0.1 (0.1-0.2)	0.3 (0.3-0.4)	0.3 (0.2-0.3)
Costa Rica	2.1 (1.5-2.9)	3.8 (2.8-5.0)	2.5 (1.9-3.3)	4.2 (3.3-5.2)
Croatia	4.6 (3.3-6.4)	4.2 (3.1-5.6)	5.6 (4.3-7.4)	5.2 (4.3-6.6)
Cuba	11.5 (8.4-16.0)	12.6 (9.4-16.6)	22.0 (16.7-29.3)	22.0 (17.6-27.7)
Cyprus	0.4 (0.3-0.5)	0.8 (0.6-1.0)	0.4 (0.4-0.5)	0.9 (0.7-1.0)
Czechia	9.7 (7.2-12.5)	13.5 (10.3-17.1)	12.0 (9.7-14.9)	20.3 (16.7-25.0)
Côte d'Ivoire	19.7 (15.1-25.5)	47.6 (36.5-62.9)	13.0 (10.3-16.6)	31.4 (24.9-39.7)
Democratic People's Republic of Korea	15.0 (11.2-19.9)	20.8 (16.0-27.2)	12.8 (10.2-15.8)	20.0 (16.0-25.1)
Democratic Republic of the Congo	166.5 (125.8-215.6)	324.6 (247.0-425.7)	62.0 (49.0-77.5)	154.8 (120.7-193.9)

Denmark	3.4 (2.6-4.3)	4.3 (3.3-5.7)	5.1 (4.1-6.2)	7.3 (5.9-9.0)
Djibouti	1.3 (0.9-1.8)	4.0 (3.0-5.3)	2.4 (1.8-3.2)	6.5 (5.0-8.2)
Dominica	0.0 (0.0-0.1)	0.0 (0.0-0.1)	0.1 (0.1-0.1)	0.1 (0.1-0.1)
Dominican Republic	6.7 (4.9-9.2)	11.5 (8.7-15.4)	29.4 (22.0-38.7)	59.9 (46.5-75.8)
Ecuador	6.7 (4.6-9.9)	12.5 (9.2-17.3)	8.2 (5.9-11.7)	14.5 (10.9-19.5)
Egypt	37.8 (27.5-51.8)	71.2 (51.5-98.4)	132.3 (105.1-165.2)	235.3 (188.8-289.8)
El Salvador	8.4 (5.6-12.4)	11.3 (8.1-15.8)	10.6 (7.8-13.9)	13.4 (10.3-17.4)
Equatorial Guinea	0.9 (0.7-1.2)	2.6 (2.0-3.4)	0.5 (0.4-0.6)	2.2 (1.7-2.8)
Eritrea	2.1 (1.5-3.0)	5.4 (4.0-7.3)	22.3 (17.7-27.7)	51.3 (40.2-64.0)
Estonia	1.6 (1.2-2.1)	1.7 (1.3-2.2)	1.7 (1.3-2.3)	2.4 (1.9-3.0)
Eswatini	0.6 (0.4-0.9)	1.1 (0.8-1.5)	1.0 (0.8-1.3)	1.6 (1.3-2.1)
Ethiopia	51.8 (37.4-71.7)	110.9 (82.3-156.0)	227.2 (181.4-278.4)	498.2 (409.0-604.8)
Fiji	4.0 (2.9-5.7)	5.1 (3.7-6.9)	9.7 (7.4-12.6)	12.0 (9.5-15.0)
Finland	2.9 (2.3-3.8)	3.6 (2.8-4.6)	5.2 (4.1-6.4)	7.8 (6.2-9.5)
France	31.9 (23.9-41.0)	38.3 (29.5-49.1)	31.8 (25.8-39.1)	38.9 (31.6-47.0)
Gabon	1.3 (1.0-1.8)	2.8 (2.1-3.7)	0.8 (0.6-1.1)	1.6 (1.2-2.1)
Gambia	1.2 (0.9-1.5)	3.2 (2.4-4.2)	2.4 (1.9-3.0)	6.4 (5.0-8.1)
Georgia	4.6 (3.4-6.2)	3.1 (2.4-4.0)	8.2 (6.5-10.6)	5.6 (4.6-7.0)
Germany	50.3 (37.7-65.0)	60.2 (46.0-78.6)	54.7 (44.3-66.5)	81.7 (67.0-99.9)
Ghana	21.7 (16.5-28.0)	60.0 (45.6-79.4)	33.6 (27.1-41.7)	75.3 (60.3-92.6)
Greece	6.1 (4.7-7.9)	6.8 (5.2-8.8)	6.2 (5.1-7.6)	7.3 (5.9-8.9)
Greenland	0.1 (0.0-0.1)	0.1 (0.1-0.1)	0.8 (0.7-1.0)	0.8 (0.6-1.0)
Grenada	0.1 (0.0-0.1)	0.1 (0.1-0.1)	0.2 (0.1-0.2)	0.3 (0.2-0.4)
Guam	1.3 (0.9-1.9)	2.0 (1.6-2.6)	4.6 (3.6-5.9)	5.8 (4.8-6.8)
Guatemala	6.1 (4.2-8.8)	17.1 (12.0-23.9)	6.8 (5.0-9.2)	18.1 (13.6-24.3)
Guinea	12.6 (9.7-16.2)	23.9 (18.0-31.2)	12.9 (10.4-15.5)	23.7 (19.1-29.6)
Guinea-Bissau	1.9 (1.4-2.4)	3.7 (2.7-4.8)	1.9 (1.5-2.3)	3.7 (2.9-4.6)

Guyana	0.7 (0.5-1.1)	0.9 (0.6-1.3)	1.3 (1.0-1.7)	1.6 (1.2-2.0)
Haiti	7.1 (5.2-9.7)	17.0 (12.4-23.0)	7.6 (6.0-9.4)	16.2 (12.7-19.9)
Honduras	3.8 (2.7-5.5)	10.4 (7.4-15.0)	5.1 (4.0-6.6)	13.5 (10.7-17.3)
Hungary	7.6 (5.8-9.7)	7.9 (6.0-10.2)	8.5 (6.8-10.4)	8.7 (7.1-10.7)
Iceland	0.2 (0.2-0.3)	0.5 (0.4-0.6)	0.5 (0.4-0.6)	1.3 (1.1-1.7)
India	25,476.4 (20,830.3-31,076.0)	53,946.8 (46,425.9-61,972.3)	73,246.8 (63,713.5-83,998.7)	131,874.9 (119,300.1-145,160.2)
Indonesia	880.6 (667.6-1,134.9)	1,611.4 (1,219.6-2,089.7)	189.5 (149.5-234.9)	390.3 (315.1-475.5)
Iran (Islamic Republic of)	59.4 (42.7-82.9)	98.5 (74.0-131.7)	270.8 (210.5-341.7)	486.9 (379.5-615.3)
Iraq	12.6 (8.6-18.1)	33.4 (23.7-47.6)	36.4 (26.7-48.3)	93.5 (71.3-123.7)
Ireland	1.8 (1.3-2.3)	2.7 (2.1-3.5)	1.8 (1.5-2.3)	2.8 (2.3-3.4)
Israel	2.6 (2.0-3.4)	5.4 (4.1-7.0)	3.3 (2.6-4.1)	7.5 (6.1-9.2)
Italy	33.1 (26.0-42.5)	36.9 (28.2-48.3)	37.6 (30.4-46.1)	48.0 (39.1-59.1)
Jamaica	2.3 (1.6-3.3)	3.2 (2.3-4.3)	4.9 (3.6-6.8)	7.1 (5.4-9.2)
Japan	166.9 (125.6-219.1)	236.9 (180.3-301.2)	335.6 (267.6-414.5)	374.8 (304.5-455.2)
Jordan	4.8 (3.3-7.0)	14.1 (10.3-19.6)	25.2 (18.8-33.6)	85.6 (65.7-112.0)
Kazakhstan	11.6 (8.4-16.0)	12.7 (9.4-16.6)	44.0 (34.5-55.4)	55.9 (45.2-68.8)
Kenya	60.1 (44.1-79.6)	162.9 (121.6-215.8)	67.7 (54.1-85.4)	190.6 (151.4-236.5)
Kiribati	0.4 (0.2-0.5)	0.6 (0.4-0.9)	1.5 (1.1-1.9)	2.4 (1.9-3.0)
Kuwait	1.1 (0.7-1.5)	3.2 (2.4-4.4)	12.6 (9.8-16.2)	32.1 (25.1-41.1)
Kyrgyzstan	3.7 (2.7-5.0)	5.5 (4.1-7.3)	81.6 (65.5-100.1)	158.1 (130.5-188.9)
Laos	54.8 (42.6-69.3)	123.3 (97.4-151.8)	8.4 (6.4-10.8)	17.6 (13.7-22.3)
Latvia	2.5 (1.8-3.4)	1.9 (1.4-2.5)	2.2 (1.7-3.0)	1.7 (1.3-2.1)
Lebanon	5.1 (3.7-7.0)	7.5 (5.6-9.9)	15.2 (12.2-18.7)	24.7 (19.9-30.9)
Lesotho	2.7 (2.1-3.7)	3.6 (2.8-4.6)	7.3 (5.7-9.2)	9.9 (7.8-12.4)
Liberia	2.4 (1.8-3.0)	5.7 (4.4-7.5)	4.5 (3.6-5.5)	11.2 (8.7-14.1)
Libya	4.2 (2.8-6.5)	8.1 (5.8-11.2)	18.6 (14.4-24.3)	36.4 (28.3-46.1)
Lithuania	2.8 (2.1-3.6)	2.4 (1.8-3.1)	2.7 (2.1-3.4)	2.3 (1.8-2.8)

Luxembourg	0.2 (0.2-0.3)	0.4 (0.3-0.5)	0.2 (0.2-0.3)	0.4 (0.3-0.5)
Madagascar	197.3 (147.6-256.5)	504.8 (386.2-635.9)	455.0 (377.4-541.2)	1,197.2 (1,022.1-1,377.4)
Malawi	27.2 (20.9-35.8)	50.3 (38.7-66.9)	12.3 (9.5-16.5)	24.9 (18.9-32.8)
Malaysia	74.7 (56.4-98.9)	182.7 (139.1-235.6)	112.8 (89.4-140.9)	345.0 (276.4-421.2)
Maldives	1.4 (1.1-1.8)	4.7 (3.7-5.9)	2.6 (2.1-3.2)	11.0 (9.2-13.3)
Mali	24.7 (18.4-32.9)	57.3 (43.3-74.9)	48.5 (38.9-60.3)	121.5 (99.9-145.9)
Malta	0.2 (0.1-0.3)	0.3 (0.2-0.3)	0.2 (0.2-0.3)	0.3 (0.3-0.4)
Marshall Islands	0.5 (0.3-0.7)	0.9 (0.7-1.2)	1.5 (1.2-1.9)	2.4 (1.9-2.9)
Mauritania	5.2 (3.8-7.1)	11.4 (8.3-15.7)	5.3 (4.2-6.8)	11.8 (9.2-15.0)
Mauritius	10.2 (8.0-12.8)	17.8 (13.6-23.2)	5.7 (4.6-7.0)	9.8 (8.0-11.8)
Mexico	94.4 (63.4-137.8)	154.7 (113.6-208.8)	132.1 (98.0-176.2)	210.2 (165.0-263.7)
Micronesia (Federated States of)	1.9 (1.2-2.8)	2.2 (1.7-3.0)	3.3 (2.4-4.3)	3.9 (3.1-4.8)
Monaco	0.0 (0.0-0.0)	0.0 (0.0-0.0)	0.0 (0.0-0.0)	0.0 (0.0-0.0)
Mongolia	4.6 (2.9-7.4)	6.3 (4.3-9.0)	14.4 (10.2-20.2)	21.4 (16.0-27.8)
Montenegro	0.7 (0.5-1.0)	0.8 (0.6-1.1)	1.3 (1.0-1.8)	1.6 (1.2-2.1)
Morocco	38.9 (27.4-55.6)	55.3 (39.5-76.8)	145.1 (113.3-188.1)	229.4 (181.0-284.3)
Mozambique	85.1 (63.6-112.5)	158.8 (119.6-211.6)	35.8 (28.4-45.3)	77.5 (60.3-100.3)
Myanmar	711.5 (561.8-916.6)	1,351.2 (1,056.0-1,704.4)	1,687.3 (1,361.4-2,050.1)	2,704.3 (2,273.8-3,176.2)
Namibia	3.5 (2.6-4.6)	6.5 (5.1-8.6)	3.7 (2.9-4.8)	6.3 (4.9-8.0)
Nauru	0.0 (0.0-0.0)	0.0 (0.0-0.0)	0.0 (0.0-0.0)	0.0 (0.0-0.0)
Nepal	364.7 (287.4-452.5)	783.4 (642.7-940.4)	1,929.6 (1,689.3-2,186.5)	3,655.2 (3,367.5-3,972.8)
Netherlands	8.3 (6.2-10.8)	9.8 (7.6-12.6)	8.5 (6.9-10.4)	10.6 (8.6-12.9)
New Zealand	5.8 (4.1-8.0)	8.1 (6.1-10.8)	6.4 (5.2-7.8)	8.7 (7.1-10.6)
Nicaragua	6.9 (4.4-10.5)	12.9 (8.8-18.9)	9.8 (7.0-13.5)	19.5 (14.5-26.9)
Niger	19.3 (14.3-25.6)	62.9 (47.1-81.4)	42.2 (34.5-51.9)	122.3 (99.5-146.4)
Nigeria	75.3 (56.9-99.3)	186.6 (139.8-247.9)	119.4 (95.8-150.4)	233.2 (185.3-290.7)
Niue	0.0 (0.0-0.0)	0.0 (0.0-0.0)	0.0 (0.0-0.0)	0.0 (0.0-0.0)

North Macedonia	2.0 (1.4-2.7)	2.5 (1.9-3.3)	3.3 (2.5-4.3)	4.7 (3.7-5.9)
Northern Mariana Islands	0.9 (0.6-1.3)	0.8 (0.6-1.2)	1.6 (1.3-2.1)	1.5 (1.2-1.9)
Norway	5.1 (3.9-6.5)	10.9 (8.2-14.2)	9.5 (7.7-11.6)	28.2 (22.8-34.2)
Oman	1.1 (0.7-1.7)	2.4 (1.7-3.4)	9.9 (7.5-12.9)	30.1 (22.7-39.4)
Pakistan	1,215.6 (950.3-1,554.8)	2,615.5 (2,089.6-3,214.1)	4,555.2 (3,730.1-5,503.9)	9,580.1 (8,190.5-11,121.8)
Palau	1.0 (0.8-1.3)	1.5 (1.2-1.7)	1.3 (1.1-1.6)	2.0 (1.7-2.3)
Palestine	3.3 (2.2-4.8)	8.3 (5.7-12.5)	11.6 (8.6-15.6)	33.6 (25.3-44.9)
Panama	2.8 (2.0-4.0)	5.3 (3.9-7.3)	3.3 (2.6-4.3)	6.1 (4.7-7.7)
Papua New Guinea	64.8 (42.3-98.1)	153.0 (111.0-207.1)	118.7 (89.6-154.4)	283.8 (220.7-355.5)
Paraguay	10.7 (8.2-14.2)	23.1 (17.8-29.3)	18.1 (14.4-22.5)	38.9 (31.4-48.0)
Peru	53.2 (35.7-79.8)	69.2 (48.6-97.0)	57.9 (42.7-77.5)	83.1 (62.0-110.3)
Philippines	157.0 (122.2-201.1)	348.6 (266.3-443.0)	386.4 (309.2-480.5)	830.8 (673.5-1,008.2)
Poland	32.4 (24.5-43.9)	39.2 (30.0-50.5)	43.7 (34.6-54.8)	55.9 (45.1-68.9)
Portugal	5.6 (4.3-7.3)	6.7 (5.2-8.4)	5.4 (4.4-6.6)	6.6 (5.4-8.0)
Puerto Rico	3.9 (2.8-5.3)	4.3 (3.3-5.6)	5.2 (4.0-6.7)	5.5 (4.4-7.0)
Qatar	0.3 (0.2-0.4)	1.3 (0.9-1.8)	2.5 (1.9-3.3)	18.3 (13.4-24.1)
Republic of Korea	51.2 (38.2-70.1)	87.9 (66.2-114.0)	89.1 (70.4-111.1)	144.0 (116.4-178.1)
Republic of Moldova	3.8 (2.8-5.1)	3.3 (2.5-4.3)	4.3 (3.4-5.5)	3.7 (3.0-4.7)
Romania	19.1 (14.6-24.6)	18.9 (14.4-24.7)	32.2 (25.7-39.6)	37.0 (29.3-45.6)
Russian Federation	186.1 (136.3-254.0)	177.7 (134.4-230.4)	180.3 (135.6-244.1)	161.8 (125.5-204.1)
Rwanda	39.1 (29.8-51.2)	84.1 (65.0-106.9)	5.6 (4.2-7.5)	10.9 (8.3-14.3)
Saint Kitts and Nevis	0.0 (0.0-0.0)	0.0 (0.0-0.1)	0.0 (0.0-0.1)	0.1 (0.0-0.1)
Saint Lucia	0.1 (0.1-0.2)	0.2 (0.1-0.2)	0.3 (0.2-0.4)	0.5 (0.4-0.7)
Saint Vincent and the Grenadines	0.1 (0.1-0.1)	0.1 (0.1-0.1)	0.1 (0.1-0.2)	0.1 (0.1-0.2)
Samoa	0.4 (0.2-0.5)	0.5 (0.3-0.7)	0.9 (0.6-1.2)	1.1 (0.9-1.5)
San Marino	0.0 (0.0-0.0)	0.0 (0.0-0.0)	0.0 (0.0-0.0)	0.0 (0.0-0.0)
Sao Tome and Principe	0.1 (0.1-0.2)	0.3 (0.2-0.3)	0.1 (0.1-0.2)	0.3 (0.2-0.3)

Saudi Arabia	12.1 (8.5-17.4)	32.6 (23.8-44.8)	63.1 (47.3-82.3)	178.5 (137.5-225.1)
Senegal	10.9 (8.2-14.7)	23.0 (17.4-30.3)	5.2 (4.0-6.7)	10.4 (8.2-13.3)
Serbia	10.0 (7.4-13.6)	11.0 (8.3-14.5)	16.1 (12.4-20.6)	18.3 (14.3-23.1)
Seychelles	0.3 (0.3-0.5)	0.4 (0.3-0.6)	0.2 (0.2-0.3)	0.3 (0.3-0.4)
Sierra Leone	12.0 (9.3-15.3)	26.0 (20.1-33.4)	11.2 (9.0-14.0)	25.3 (20.3-31.5)
Singapore	3.9 (2.8-5.3)	10.8 (8.1-14.3)	7.8 (6.1-9.7)	17.4 (13.8-21.2)
Slovakia	3.6 (2.8-4.7)	4.3 (3.3-5.4)	4.1 (3.3-5.2)	5.2 (4.3-6.3)
Slovenia	1.7 (1.3-2.2)	2.1 (1.6-2.7)	2.3 (1.8-2.8)	3.2 (2.6-3.9)
Solomon Islands	2.5 (1.6-3.8)	5.2 (3.6-7.3)	3.8 (2.8-5.0)	7.6 (5.9-9.9)
Somalia	17.0 (12.6-22.7)	46.9 (34.0-63.2)	21.7 (17.0-27.2)	58.2 (45.2-73.5)
South Africa	250.7 (180.0-333.4)	405.3 (312.4-519.2)	167.3 (119.8-226.4)	178.6 (133.5-237.3)
South Sudan	14.2 (10.5-19.4)	25.6 (18.4-35.2)	24.2 (19.0-31.0)	34.7 (26.9-45.1)
Spain	21.4 (16.3-27.3)	27.6 (21.0-35.3)	21.0 (17.0-25.4)	28.1 (22.7-34.3)
Sri Lanka	251.1 (196.1-318.8)	500.5 (391.9-625.7)	666.5 (550.3-791.9)	1,132.4 (947.6-1,315.0)
Sudan	13.5 (9.5-19.6)	26.8 (19.2-38.1)	302.0 (231.9-386.7)	759.3 (600.8-950.7)
Suriname	0.4 (0.3-0.6)	0.7 (0.5-0.9)	1.3 (0.9-1.8)	1.8 (1.3-2.3)
Sweden	11.5 (8.6-15.2)	14.5 (10.7-18.9)	59.3 (48.1-72.5)	72.2 (58.5-88.7)
Switzerland	4.4 (3.3-5.7)	6.1 (4.7-8.0)	5.8 (4.7-7.2)	9.5 (7.7-11.5)
Syrian Arab Republic	10.3 (6.5-16.3)	18.9 (12.5-29.7)	14.0 (10.1-19.8)	28.4 (20.0-41.6)
Taiwan (Province of China)	21.5 (15.9-29.4)	27.5 (21.3-36.3)	48.0 (37.7-60.1)	59.1 (47.2-73.6)
Tajikistan	8.7 (6.4-12.0)	19.7 (14.7-25.9)	70.1 (55.3-88.2)	195.4 (154.9-240.7)
Thailand	373.7 (285.3-470.2)	897.2 (685.8-1,162.5)	186.7 (152.6-226.6)	365.9 (301.7-437.3)
Timor-Leste	4.1 (3.1-5.4)	8.3 (6.4-11.0)	2.4 (1.9-3.0)	5.0 (4.0-6.4)
Togo	7.5 (5.6-9.9)	19.9 (15.1-25.6)	6.5 (5.0-8.2)	15.6 (12.2-19.4)
Tokelau	0.0 (0.0-0.0)	0.0 (0.0-0.0)	0.0 (0.0-0.0)	0.0 (0.0-0.0)
Tonga	0.7 (0.5-1.1)	0.8 (0.5-1.2)	1.6 (1.1-2.1)	1.7 (1.3-2.3)
Trinidad and Tobago	1.3 (0.8-1.8)	1.5 (1.1-2.1)	2.6 (1.8-3.6)	2.7 (2.0-3.6)

Tunisia	28.9 (21.9-38.3)	47.7 (35.8-61.4)	116.6 (95.3-140.3)	170.0 (137.9-208.0)
Turkey	45.7 (32.0-65.4)	62.2 (46.2-82.9)	129.3 (98.9-171.4)	178.1 (139.1-225.4)
Turkmenistan	2.0 (1.4-2.7)	3.0 (2.2-4.0)	4.4 (3.3-5.7)	6.8 (5.3-8.8)
Tuvalu	0.1 (0.0-0.1)	0.1 (0.1-0.1)	0.1 (0.1-0.2)	0.2 (0.2-0.3)
Uganda	34.7 (26.2-46.0)	80.9 (60.4-107.2)	28.0 (21.7-36.7)	72.1 (54.7-95.5)
Ukraine	46.4 (35.5-60.8)	40.3 (30.6-51.8)	67.4 (53.7-87.2)	58.1 (46.1-72.8)
United Arab Emirates	1.8 (1.2-2.6)	7.5 (5.2-10.6)	17.5 (13.5-22.7)	108.9 (80.8-148.2)
United Kingdom	32.1 (24.7-41.0)	38.1 (29.9-48.7)	36.1 (29.5-43.9)	44.0 (35.6-53.5)
United Republic of Tanzania	42.7 (32.2-56.5)	104.5 (79.7-139.6)	47.2 (37.5-58.0)	109.2 (86.7-135.1)
United States Virgin Islands	0.1 (0.1-0.1)	0.1 (0.1-0.1)	0.1 (0.1-0.1)	0.1 (0.1-0.2)
United States of America	374.6 (285.4-479.3)	526.8 (400.5-682.5)	4,330.4 (3,820.2-4,923.5)	5,156.6 (4,269.9-6,145.9)
Uruguay	2.5 (1.9-3.3)	2.8 (2.2-3.7)	2.0 (1.6-2.4)	2.1 (1.7-2.6)
Uzbekistan	13.8 (9.7-19.5)	24.5 (17.9-33.4)	505.2 (382.5-639.7)	1,077.7 (877.4-1,319.3)
Vanuatu	1.1 (0.7-1.6)	2.3 (1.6-3.2)	2.1 (1.6-2.6)	4.3 (3.3-5.4)
Venezuela (Bolivarian Republic of)	31.3 (22.8-44.3)	54.1 (40.3-71.4)	127.8 (99.3-164.9)	219.0 (175.9-269.4)
Viet Nam	264.9 (200.0-341.7)	532.0 (404.8-691.2)	103.5 (83.3-130.0)	221.4 (177.7-272.3)
Yemen	161.3 (127.1-204.2)	351.6 (270.1-452.5)	409.5 (337.3-491.9)	925.6 (767.4-1,127.6)
Zambia	19.3 (14.1-26.2)	54.5 (40.9-74.4)	12.6 (9.6-16.5)	35.3 (27.6-45.7)
Zimbabwe	12.5 (8.9-17.8)	25.1 (18.6-35.3)	12.0 (9.0-16.4)	17.6 (13.1-23.1)

Supplementary Table 5. Age-standardized chewing tobacco prevalence in 2019 by region, for males and females separately.

Region	Males	Females
Andean Latin America	0.58% (0.48 to 0.72	0.46% (0.36 to 0.58
Australasia	0.54% (0.45 to 0.65	0.31% (0.24 to 0.4
Caribbean	0.73% (0.64 to 0.83	0.31% (0.27 to 0.35
Central Asia	4.45% (3.84 to 5.18	0.25% (0.22 to 0.28
Central Europe	0.47% (0.43 to 0.52	0.27% (0.24 to 0.31
Central Latin America	0.69% (0.62 to 0.79	0.38% (0.32 to 0.45
Central Sub-Saharan Africa	0.64% (0.54 to 0.74	1.24% (1 to 1.55
East Asia	0.84% (0.67 to 1.05	0.23% (0.17 to 0.32
Eastern Europe	0.35% (0.29 to 0.44	0.3% (0.23 to 0.39
Eastern Sub-Saharan Africa	2.51% (2.31 to 2.72	1.4% (1.27 to 1.54
High-income Asia Pacific	0.64% (0.55 to 0.74	0.43% (0.35 to 0.53
High-income North America	3.81% (3.11 to 4.56	0.37% (0.28 to 0.49
North Africa and Middle East	2.61% (2.43 to 2.8	0.46% (0.41 to 0.51
Oceania	6.8% (5.56 to 8.23	3.78% (2.91 to 4.89
South Asia	24.65% (22.81 to 26.69	12.13% (10.91 to 13.45
Southeast Asia	2.48% (2.27 to 2.69	2.59% (2.34 to 2.87
Southern Latin America	0.17% (0.15 to 0.2	0.24% (0.18 to 0.31
Southern Sub-Saharan Africa	0.77% (0.62 to 0.96	1.82% (1.49 to 2.24
Tropical Latin America	0.5% (0.4 to 0.61	0.35% (0.28 to 0.45
Western Europe	0.23% (0.21 to 0.24	0.15% (0.14 to 0.17
Western Sub-Saharan Africa	0.94% (0.88 to 1	0.8% (0.73 to 0.87

Authors' Contributions

Managing the estimation or publications process

Erin C Mullany, Christopher J L Murray, and Emmanuela Gakidou

Writing the first draft of the manuscript

Parkes J Kendrick, Marissa B Reitsma, Simon Hay and Emmanuela Gakidou.

Primary responsibility for applying analytical methods to produce estimates

Parkes J Kendrick, Marissa B Reitsma, Xiaochen Dai, Luisa Sorio Flor, Thomas Hsiao, Hayley D Tymeson, and Rachel L Updike.

Primary responsibility for seeking, cataloguing, extracting, or cleaning data; designing or coding figures and tables

Parkes J Kendrick, Marissa B Reitsma, Jason A Anderson, Nicholas D Arian, Xiaochen Dai, Rachel Feldman, Luisa Sorio Flor, Gabriela Fernanda Gil, Thomas Hsiao, Hayley D Tymeson, Rachel L Updike, and Joanna L Whisnant.

Providing data or critical feedback on data sources

Parkes J Kendrick, Amir Abdoli, Mohammad Abdollahi, E S Abhilash, Oladimeji M Adebayo, Bright Opoku Ahinkorah, Sohail Ahmad, Haroon Ahmed, Budi Aji, Chisom Joyqueenet Akunna, Fares Alahdab, Fahad Mashhour Alanezi, Turki M Alanzi, Khalid F Alhabib, Tilahun Ali, Sheikh Mohammad Alif, Syed Mohamed Aljunid, Saeed Amini, Hubert Amu, Catalina Liliana Andrei, Tudorel Andrei, Benny Antony, Davood Anvari, Jalal Arabloo, Wondwossen Niguse Asmare, Desta Debalkie Atnafu, Marcel Ausloos, Samad Azari, Atif Amin Baig, Maciej Banach, Till Winfried Bärnighausen, Sanjay Basu, Catherine P Benziger, Adam E Berman, Akshaya Srikanth Bhagavathula, Sadia Bibi, Dejana Braithwaite, Katrin Burkart, Sharath Burugina Nagaraja, Florentino Luciano Caetano dos Santos, Joao Mauricio Castaldelli-Maia, Dinh-Toi Chu, Vera Marisa Costa, Albertino Antonio Moura Damasceno, Giovanni Damiani, Lalit Dandona, Rakhi Dandona, Meseret Derbew Molla, Samath Dhamminda Dharmaratne, Meghnath Dhimal, Andem Effiong, Irina Filip, Luisa Sorio Flor, Masoud Foroutan, Mohamed M Gad, Birhan Gebresillassie Gebregiorgis, Lemma Getacher, Mansour Ghafourifard, Reza Ghanei Gheshlagh, Ahmad Ghashghaee, Nermin Ghith, Mahaveer Golechha, Sameer Vali Gopalani, Rafael Alves Guimarães, Yuming Guo, Rajat Das Gupta, Rajeev Gupta, Nima Hafezi-Nejad, Claudiu Herteliu, Kamal Hezam, Mehdi Hosseinzadeh, Mowafa Househ, Charles Ugochukwu Ibeneme, Segun Emmanuel Ibitoye, Seyed Sina Naghibi Irvani, Rakibul M Islam, Sheikh Mohammed Shariful Islam, Vardhmaan Jain, Mihajlo Jakovljevic, Sung-In Jang, Shubha Jayaram, Panniyammakal Jeemon, Jost B Jonas, Mikk Jürisson, Zubair Kabir, Leila R Kalankesh, André Karch, Salah Eddin Karimi, Kindie Mitiku Kebede, Bayew Kelkay, Yousef Saleh Khader, Gyu Ri Kim, Soewarta Kosen, Kewal Krishan, Nuworza Kugbey, G Anil Kumar, Dian Kusuma, Savita Lasrado, Doo Woong Lee, Yo Han Lee, Janni Leung, Shanshan Li, Wei Liu, Shilpashree Madhava Kunjathur, Deborah Carvalho Malta, Abdullah A Mamun, Borhan Mansouri, Mohammad Ali Mansournia, Manu Raj Mathur, Mohsen Mazidi, Carlo Eduardo Medina-Solís, Walter Mendoza, Ritesh G Menezes, Bartosz Miazgowski, Irmina Maria Michalek, GK Mini, Erkin M Mirrakhimov, Abdollah Mohammadian-Hafshejani, Shafiu Mohammed, Ali H Mokdad, Mariam Molokhia, Lorenzo Monasta, Tilahun Belete Mossie, Sumaira Mubarik, Mohsen Naghavi, Sreenivas Narasimha Swamy, Javad Nazari, Ruxandra Irina Negoi, Huong Lan Thi Nguyen, Chukwudi A Nnaji, Jean Jacques Noubiap, Felix Akpojene Ogbo, Ayodipupo Sikiru Oguntade, Andrew T Olagunju, Mayowa O Owolabi, Mahesh P A, Keyvan Pakshir, Demosthenes Panagiotakos,

Songhomitra Panda-Jonas, Eun-Cheol Park, Fatemeh Pashazadeh Kan, Shrikant Pawar, Hai Quang Pham, Khem Narayan Pokhrel, Amir Radfar, Vafa Rahimi-Movaghar, Mohammad Hifz Ur Rahman, Amir Masoud Rahmani, Pradhum Ram, Chhabi Lal Ranabhat, David Laith Rawaf, Salman Rawaf, Reza Rawassizadeh, Andre M N Renzaho, Leonardo Roever, Luca Ronfani, Gholamreza Roshandel, Ambuj Roy, Basema Saddik, Abdallah M Samy, Milena M Santric-Milicevic, Brijesh Sathian, Monika Sawhney, Ganesh Kumar Saya, Falk Schwendicke, Syed Mahboob Shah, Masood Ali Shaikh, Mohammed Shannawaz, Sara Sheikhbahaei, K M Shivakumar, Roman Shrestha, Soraya Siabani, Negussie Boti Sidemo, Ambrish Singh, Jasvinder A Singh, Virendra Singh, Valentin Yurievich Skryabin, Anna Aleksandrovna Skryabina, Ireneous N Soyiri, Chandrashekhar T Sreeramareddy, Rizwan Suliankatchi Abdulkader, Animut Tagele Tamiru, Musliu Adetola Tolani, Marcos Roberto Tovani-Palone, Bach Xuan Tran, Gebiyaw Wudie Tsegaye, Saif Ullah, Olalekan A Uthman, Narayanaswamy Venketasubramanian, Bay Vo, Giang Thu Vu, Yasir Waheed, Vahid Yazdi-Feyzabadi, Naohiro Yonemoto, Chuanhua Yu, and Yves Miel H Zuniga.

Developing methods or computational machinery

Parkes J Kendrick, Davood Anvari, Kurnia Dwi Artanti, Srikanta K Banerjee, Xiaochen Dai, Yuming Guo, Mowafa Househ, Kindie Mitiku Kebede, Shanshan Li, Borhan Mansouri, Abdollah Mohammadian-Hafshejani, Ali H Mokdad, Mohammad Ali Moni, Mohsen Naghavi, Mona Pathak, Chhabi Lal Ranabhat, Abdallah M Samy, Negussie Boti Sidemo, Bhaskar Thakur, and Mikhail Sergeevich Zastrozhin.

Providing critical feedback on methods or results

Parkes J Kendrick, Mohammad Abdollahi, Aidin Abedi, E S Abhilash, Victor Aboyans, Oladimeji M Adebayo, Shailesh M Advani, Bright Opoku Ahinkorah, Sohail Ahmad, Keivan Ahmadi, Haroon Ahmed, Budi Aji, Yonas Akalu, Chisom Joyqueenet Akunna, Fares Alahdab, Ziyad Al-Aly, Fahad Mashhour Alanezi, Turki M Alanzi, Khalid F Alhabib, Tilahun Ali, Sheikh Mohammad Alif, Vahid Alipour, Syed Mohamed Aljunid, Mahmoud A Alomari, Tarek Tawfik Amin, Hubert Amu, Robert Ancuceanu, Catalina Liliana Andrei, Tudorel Andrei, Alireza Ansari-Moghaddam, Benny Antony, Davood Anvari, Jalal Arabloo, Wondwossen Niguse Asmare, Marcel Ausloos, Asma Tahir Awan, Getinet Ayano, Getie Lake Aynalem, Samad Azari, Darshan B B, Ashish D Badiye, Atif Amin Baig, Maciej Banach, Srikanta K Banerjee, Suzanne Lyn Barker-Collo, Till Winfried Bärnighausen, Sanjay Basu, Mohsen Bayati, Shahrzad Bazargan-Hejazi, Tariku Tesfaye Bekuma, Derrick A Bennett, Isabela M Bensenor, Catherine P Benziger, Adam E Berman, Akshaya Srikanth Bhagavathula, Nikha Bhardwaj, Pankaj Bhardwaj, Krittika Bhattacharyya, Sadia Bibi, Ali Bijani, Antonio Biondi, Dejana Braithwaite, Hermann Brenner, Andre R Brunoni, Katrin Burkart, Sharath Burugina Nagaraja, Zahid A Butt, Florentino Luciano Caetano dos Santos, Josip Car, Giulia Carreras, Joao Mauricio Castaldelli-Maia, Jung-Chen Chang, Pankaj Chaturvedi, Onyema greg Chido-Amajuoyi, Dinh-Toi Chu, Sheng-Chia Chung, Liliana G Ciobanu, Vera Marisa Costa, Baye Dagnew, Xiaochen Dai, Giovanni Damiani, Lalit Dandona, Rakhi Dandona, Parnaz Daneshpajouhnejad, Jiregna Darega Gela, Meseret Derbew Molla, Abebaw Alemayehu Desta, Meghnath Dhimal, Arielle Wilder Eagan, Mohammad Ebrahimi Kalan, Kristina Edvardsson, Andem Effiong, Saman Esmaeilnejad, Ibtihal Fadhil, Emerito Jose A Faraon, Medhat Farwati, Farshad Farzadfar, Valery L Feigin, Irina Filip, Filippos Filippidis, Florian Fischer, Luisa Sorio Flor, Nataliya A Foigt, Morenike Oluwatoyin Folayan, Masoud Foroutan, Mohamed M Gad, Biniyam Sahiledengle Geberemariyam, Birhan Gebresillassie Gebregiorgis, Lemma Getacher, Abera Getachew Obsa, Ahmad Ghashghaee, Nermin Ghith, Paramjit Singh Gill, Ibrahim Abdelmageed Ginawi, Salime Goharinezhad, Mahaveer Golechha, Sameer Vali Gopalani, Michal Grivna, Avirup Guha, Yuming Guo, Rajat Das Gupta, Rajeev Gupta, Tarun Gupta, Nima Hafezi-Nejad, Mohammad Rifat Haider, Arief Hargono, Simon I Hay, Golnaz Heidari, Claudiu Herteliu, Thomas R Hird, Ramesh Holla, Mihaela Hostiuc,

Sorin Hostiuc, Mowafa Househ, Charles Ugochukwu Ibeneme, Segun Emmanuel Ibitoye, Irena M Ilic, Milena D Ilic, Leeberk Raja Inbaraj, Seyed Sina Naghibi Irvani, Jessica Y Islam, Rakibul M Islam, Sheikh Mohammed Shariful Islam, Farhad Islami, Ramaiah Itumalla, Jalil Jaafari, Vardhmaan Jain, Mihajlo Jakovljevic, Sung-In Jang, Shubha Jayaram, Panniyammakal Jeemon, Ravi Prakash Jha, Jost B Jonas, Mikk Jürisson, Ali Kabir, Zubair Kabir, Leila R Kalankesh, Tanuj Kanchan, Himal Kandel, Neeti Kapoor, André Karch, Salah Eddin Karimi, Kindie Mitiku Kebede, Bayew Kelkay, Yousef Saleh Khader, Ejaz Ahmad Khan, Maryam Khayamzadeh, Gyu Ri Kim, Ruth W Kimokoti, Mika Kivimäki, Sindhura Lakshmi Koulmane Laxminarayana, Ai Koyanagi, Kewal Krishan, Nuworza Kugbey, G Anil Kumar, Nithin Kumar, Om P Kurmi, Dian Kusuma, Savita Lasrado, Paolo Lauriola, Doo Woong Lee, Yo Han Lee, Janni Leung, Shanshan Li, Hualiang Lin, Wei Liu, Shilpashree Madhava Kunjathur, Azeem Majeed, Reza Malekzadeh, Deborah Carvalho Malta, Abdullah A Mamun, Borhan Mansouri, Mohammad Ali Mansournia, Santi Martini, Manu Raj Mathur, Prashant Mathur, Mohsen Mazidi, Martin McKee, Carlo Eduardo Medina-Solís, Suresh Mehata, Walter Mendoza, Ritesh G Menezes, Irmina Maria Michalek, Ted R Miller, GK Mini, Andreea Mirica, Erkin M Mirrakhimov, Hamed Mirzaei, Sanjeev Misra, Yousef Mohammad, Abdollah Mohammadian-Hafshejani, Shafiu Mohammed, Ali H Mokdad, Mariam Molokhia, Mohammad Ali Moni, Tilahun Belete Mossie, Sumaira Mubarik, Shankar Prasad Nagaraju, Mohsen Naghavi, Nitish Naik, Mahdi Nalini, Atta Abbas Naqvi, Sreenivas Narasimha Swamy, Muhammad Naveed, Javad Nazari, Ruxandra Irina Negoi, Sandhya Neupane Kandel, Huong Lan Thi Nguyen, Yeshambel T Nigatu, Chukwudi A Nnaji, Jean Jacques Noubiap, Christoph Nowak, Felix Akpojene Ogbo, Ayodipupo Sikiru Oguntade, Andrew T Olagunju, Mayowa O Owolabi, Mahesh P A, Adrian Pana, Demosthenes Panagiotakos, Songhomitra Panda-Jonas, Ashok Pandey, Utsav Parekh, Eun-Cheol Park, Eun-Kee Park, Fatemeh Pashazadeh Kan, Mona Pathak, Shrikant Pawar, Richard G Pestell, Hai Quang Pham, Khem Narayan Pokhrel, Akram Pourshams, Akila Prashant, Amir Radfar, Vafa Rahimi-Movaghar, Mohammad Hifz Ur Rahman, Muhammad Aziz Rahman, Pradhum Ram, Juwel Rana, Chhabi Lal Ranabhat, Priya Rathi, David Laith Rawaf, Salman Rawaf, Andre M N Renzaho, Aziz Rezapour, Mavra A Riaz, Leonardo Roever, Gholamreza Roshandel, Bedanta Roy, Basema Saddik, Sana Salehi, Abdallah M Samy, Juan Sanabria, Milena M Santric-Milicevic, Bruno Piassi Sao Jose, Brijesh Sathian, Monika Sawhney, Ganesh Kumar Saya, Falk Schwendicke, Abdul-Aziz Seidu, Nachimuthu Senthil Kumar, Sadaf G Sepanlou, Omid Shafaat, Syed Mahboob Shah, Masood Ali Shaikh, Mohammed Shannawaz, Kiomars Sharafi, Aziz Sheikh, Sara Sheikhbahaei, Mika Shigematsu, Rahman Shiri, Kawkab Shishani, K M Shivakumar, Siddharudha Shivalli, Roman Shrestha, Soraya Siabani, Negussie Boti Sidemo, João Pedro Silva, Ambrish Singh, Jasvinder A Singh, Dhirendra Narain Sinha, Valentin Yurievich Skryabin, Anna Aleksandrovna Skryabina, Ali Soroush, Ireneous N Soyiri, Chandrashekhar T Sreeramareddy, Paschalis Steiropoulos, Stefan Stortecky, Kurt Straif, Rizwan Suliankatchi Abdulkader, Gerhard Sulo, Eyayou Girma Tadesse, Animut Tagele Tamiru, Minale Tareke, Md Ismail Tareque, Ingan Ukur Tarigan, Bhaskar Thakur, Rekha Thapar, Musliu Adetola Tolani, Marcos Roberto Tovani-Palone, Bach Xuan Tran, Jaya Prasad Tripathy, Gebiyaw Wudie Tsegaye, Saif Ullah, Olalekan A Uthman, Marco Vacante, Constantine Vardavas, Narayanaswamy Venketasubramanian, Giang Thu Vu, Yasir Waheed, Yanzhong Wang, Andrea Werdecker, Joanna L Whisnant, Nuwan Darshana Wickramasinghe, Befikadu Legesse Wubishet, Vahid Yazdi-Feyzabadi, Yigizie Yeshaw, Mohammed Zewdu Yimmer, Naohiro Yonemoto, Zabihollah Yousefi, Chuanhua Yu, Hasan Yusefzadeh, Mohammad Zamani, Maryam Zamanian, Mikhail Sergeevich Zastrozhin, Anasthasia Zastrozhina, Jianrong Zhang, Zhi-Jiang Zhang, and Yves Miel H Zuniga.

Drafting the work or revising is critically for important intellectual content

Parkes J Kendrick, Mohsen Abbasi-Kangevari, Mohammad Abdollahi, Aidin Abedi, Oladimeji M Adebayo, Shailesh M Advani, Bright Opoku Ahinkorah, Haroon Ahmed, Budi Aji, Yonas Akalu, Fares Alahdab, Mahmoud A Alomari, Tarek Tawfik Amin, Hubert Amu, Robert Ancuceanu, Benny Antony, Jalal Arabloo, Monika Arora, Kurnia Dwi Artanti, Marcel Ausloos, Asma Tahir Awan, Darshan B B, Atif Amin Baig, Maciej Banach, Suzanne Lyn Barker-Collo, Till Winfried Bärnighausen, Hiba Jawdat Barqawi, Sanjay Basu, Tariku Tesfaye Bekuma, Derrick A Bennett, Isabela M Bensenor, Habib Benzian, Catherine P Benziger, Adam E Berman, Akshaya Srikanth Bhagavathula, Neeraj Bhala, Krittika Bhattacharyya, Sadia Bibi, Antonio Biondi, Dejana Braithwaite, Hermann Brenner, Andre R Brunoni, Sharath Burugina Nagaraja, Florentino Luciano Caetano dos Santos, Josip Car, Joao Mauricio Castaldelli-Maia, Maria Sofia Sofia Cattaruzza, Pankaj Chaturvedi, Simiao Chen, Onyema greg Chido-Amajuoyi, Dinh-Toi Chu, Liliana G Ciobanu, Vera Marisa Costa, Rosa A S Couto, Baye Dagnew, Albertino Antonio Moura Damasceno, Giovanni Damiani, Meseret Derbew Molla, Samath Dhamminda Dharmaratne, Meghnath Dhimal, Arielle Wilder Eagan, Mohammad Ebrahimi Kalan, Andem Effiong, Maha El Tantawi, Iffat Elbarazi, Saman Esmaeilnejad, Medhat Farwati, Mehdi Fazlzadeh, Irina Filip, Filippos Filippidis, Florian Fischer, Luisa Sorio Flor, Nataliya A Foigt, Morenike Oluwatoyin Folayan, Masoud Foroutan, Mohamed M Gad, Silvano Gallus, Biniyam Sahiledengle Geberemariyam, Birhan Gebresillassie Gebregiorgis, Lemma Getacher, Ahmad Ghashghaee, Nermin Ghith, Paramjit Singh Gill, Sameer Vali Gopalani, Giuseppe Gorini, Michal Grivna, Rafael Alves Guimarães, Rajat Das Gupta, Rajeev Gupta, Nima Hafezi-Nejad, Randah R Hamadeh, Graeme J Hankey, Simon I Hay, Golnaz Heidari, Claudiu Herteliu, Kamal Hezam, Thomas R Hird, Ramesh Holla, Mowafa Househ, Junjie Huang, Segun Emmanuel Ibitoye, Irena M Ilic, Milena D Ilic, Seyed Sina Naghibi Irvani, Jessica Y Islam, Rakibul M Islam, Sheikh Mohammed Shariful Islam, Farhad Islami, Hiroyasu Iso, Ramaiah Itumalla, Jalil Jaafari, Vardhmaan Jain, Mihajlo Jakovljevic, Sung-In Jang, Shubha Jayaram, Panniyammakal Jeemon, Ravi Prakash Jha, Jost B Jonas, Mikk Jürisson, Ali Kabir, Himal Kandel, André Karch, Kindie Mitiku Kebede, Ejaz Ahmad Khan, Gyu Ri Kim, Mika Kivimäki, Sindhura Lakshmi Koulmane Laxminarayana, Ai Koyanagi, Kewal Krishan, Nuworza Kugbey, Om P Kurmi, Dian Kusuma, Ben Lacey, Iván Landires, Savita Lasrado, Doo Woong Lee, Janni Leung, Hualiang Lin, Wei Liu, Alessandra Lugo, Shilpashree Madhava Kunjathur, Azeem Majeed, Afshin Maleki, Reza Malekzadeh, Deborah Carvalho Malta, Abdullah A Mamun, Narayana Manjunatha, Borhan Mansouri, Manu Raj Mathur, Mohsen Mazidi, Carlo Eduardo Medina-Solís, Walter Mendoza, Ritesh G Menezes, Irmina Maria Michalek, Ted R Miller, Yousef Mohammad, Abdollah Mohammadian-Hafshejani, Shafiu Mohammed, Ali H Mokdad, Mariam Molokhia, Lorenzo Monasta, Mohammad Ali Moni, Rahmatollah Moradzadeh, Shane Douglas Morrison, Tilahun Belete Mossie, Shankar Prasad Nagaraju, Mohsen Naghavi, Mahdi Nalini, Vinay Nangia, Sreenivas Narasimha Swamy, Sabina O Nduaguba, Ruxandra Irina Negoi, Sandhya Neupane Kandel, Huong Lan Thi Nguyen, Molly R Nixon, Jean Jacques Noubiap, Christoph Nowak, Virginia Nuñez-Samudio, Felix Akpojene Ogbo, In-Hwan Oh, Andrew T Olagunju, Mahesh P A, Demosthenes Panagiotakos, Songhomitra Panda-Jonas, Eun-Cheol Park, Fatemeh Pashazadeh Kan, Mona Pathak, Shrikant Pawar, Hai Quang Pham, Marina Pinheiro, Khem Narayan Pokhrel, Akila Prashant, Amir Radfar, Vafa Rahimi-Movaghar, Mohammad Hifz Ur Rahman, Pradhum Ram, Chhabi Lal Ranabhat, Priya Rathi, David Laith Rawaf, Salman Rawaf, Andre M N Renzaho, Mavra A Riaz, Leonardo Roever, Luca Ronfani, Bedanta Roy, Basema Saddik, Amirhossein Sahebkar, Hamideh Salimzadeh, Abdallah M Samy, Juan Sanabria, Milena M Santric-Milicevic, Ganesh Kumar Saya, Sadaf G Sepanlou, Syed Mahboob Shah, Sara Sheikhbahaei, Mika Shigematsu, Kawkab Shishani, K M Shivakumar, Siddharudha Shivalli, Negussie Boti Sidemo, Inga Dora Sigfusdottir, Rannveig Sigurvinsdottir, João Pedro

Silva, Jasvinder A Singh, Dhirendra Narain Sinha, Ali Soroush, Ireneous N Soyiri, Chandrashekhar T Sreeramareddy, Dan J Stein, Paschalis Steiropoulos, Stefan Stortecky, Kurt Straif, Johan Sundström, Takahiro Tabuchi, Eyayou Girma Tadesse, Animut Tagele Tamiru, Minale Tareke, Bhaskar Thakur, Kavumpurathu Raman Thankappan, Musliu Adetola Tolani, Marcos Roberto Tovani-Palone, Bach Xuan Tran, Jaya Prasad Tripathy, Saif Ullah, Brigid Unim, Marco Vacante, Constantine Vardavas, Narayanaswamy Venketasubramanian, Madhur Verma, Simone Vidale, Giang Thu Vu, Yasir Waheed, Yanzhong Wang, Andrea Werdecker, Nuwan Darshana Wickramasinghe, Befikadu Legesse Wubishet, Kazumasa Yamagishi, Yuichiro Yano, Vahid Yazdi-Feyzabadi, Yigizie Yeshaw, Naohiro Yonemoto, Ismaeel Yunusa, Muhammed Shahriar Zaman, Maryam Zamanian, Mikhail Sergeevich Zastrozhin, Anasthasia Zastrozhina, and Chenwen Zhong.

Extracting, cleaning, or cataloging data; designing or coding figures and tables

Parkes J Kendrick, Khalid F Alhabib, Saeed Amini, Hubert Amu, Jason A Anderson, Kurnia Dwi Artanti, Xiaochen Dai, Giovanni Damiani, Jalil Jaafari, Borhan Mansouri, Manu Raj Mathur, Ali H Mokdad, Mohsen Naghavi, Javad Nazari, Bedanta Roy, Abdallah M Samy, Ganesh Kumar Saya, Nachimuthu Senthil Kumar, Negussie Boti Sidemo, and Joanna L Whisnant.

Managing the overall research enterprise

Lalit Dandona, Simon I Hay, Ali H Mokdad, Erin C Mullany, Christopher J L Murray, Mohsen Naghavi, and Molly R Nixon.
